# pH-responsive manganese carboxymethyl chitosan hydrogel releases pesticide to promote rice tillering and suppress root-parasitic weeds

**DOI:** 10.1038/s41467-026-78320-3

**Published:** 2026-10-09

**Authors:** Ali Dai, Zhiguo Zheng, Ronghao Wu, Dengke Ma, Junyan Xu, You Mo, Liusheng Duan, Weiming Tan

**Affiliations:** 1 Engineering Research Centre of Plant Growth Regulators, Ministry of Education, College of Agronomy and Biotechnology, China Agricultural University, No. 2 Yuanmingyuan West Road, Haidian District, Beijing, China; 2 State Key Laboratory of Green Pesticide, Center for R&D of Fine Chemicals of Guizhou University, Guiyang, China

**Keywords:** Strigolactone, Plant signalling

## Abstract

Strigolactone (SL) signaling is an important agricultural target for controlling plant branching and parasitic weeds. Here, based on the SL inhibitor KK094, we describe the design of 32 indole derivatives, of which DK6 exhibits optimal activity. To enhance solubility and bioavailability, we describe a pH-responsive manganese-coordinated carboxymethyl chitosan hydrogel (CMCS-DK6), showing sustained release under alkaline conditions. We demonstrate CMCS-DK6 strongly inhibits germination of parasitic weeds, especially *Striga asiatica* (IC_50_ = 1.80 μM), while promoting rice tillering, reducing height, and increasing dry weight. Molecular simulations, along with microscale thermophoresis results, suggest that DK6 binds stably to SL receptors. Transcriptomics reveal DK6 suppresses SL signaling, modulates metabolic pathways, and interacts with hormone pathways to regulate tillering. This work offers a hydrogel delivery strategy and some potential mechanistic implications for improving crop architecture and weed control.

## Abstract (web summary)

Parasitic weeds can threaten crop production. Here the authors describe a pH-responsive hydrogel that releases a strigolactone inhibitor to promote rice tillering and suppress parasitic weed germination.

## Introduction

Strigolactones (SLs), a class of carotenoid-derived compounds exuded by plant roots, play a dual role in plant growth and ecological interactions^1,2^. On one hand, SLs induce seed germination of root parasitic weeds, leading to severe infestations in crops such as legumes (*Orobanche* spp.) and cereals like rice, sorghum, and maize (*Striga* spp.)^3–6^. These parasites cause substantial agricultural losses, estimated at approximately $1 billion annually in sub-Saharan Africa for *Striga* spp. and hundreds of millions for *Orobanche* spp., posing a significant threat to food security^7,8^. On the other hand, SLs function as a class of plant hormones that critically regulate plant architecture, primarily by inhibiting rice tillering through the D14-D3-D53 signaling pathway, thereby shaping plant form and potential yield^9,10^. This regulatory mechanism provides a foundational basis and key molecular targets for genetically or chemically manipulating the SL pathway to optimize crop architecture and enhance productivity.

Chemical intervention in the SL pathway represents a promising strategy for concurrently modulating rice tillering and controlling parasitic weeds. Early SL analogs (e.g., GR24, 2NOD, Nijmegen-1, SPL7) can induce parasitic seed germination^11–14^. However, their efficacy relies on host-derived nutrients, often failing to deplete the soil seed bank and potentially increasing infestation risk by enhancing weed recognition of hosts^13–15^. Furthermore, these compounds often suffer from poor stability, narrow application windows, and mimic endogenous SLs by binding the D14 receptor, consequently suppressing rice tillering and potentially compromising yield^15,16^. Research focus has thus shifted towards developing SL antagonists to achieve the dual objectives of promoting crop branching and suppressing parasitic weed germination. Notably, KK094 irreversibly binds D14, blocking SL signaling to promote rice tillering and inhibit *Striga* germination^17^. DL1 acts as a reversible competitive inhibitor, increasing branching in *Arabidopsis thaliana*^18^. SOP competitively binds *Sh*HTL7, a SL receptor in weeds, to disrupt parasitic signal perception without triggering germination^19^. Additional functional molecules like 2-MN, Triton X-100, and 7’-carba-4BD further support SL signaling research^20–22^. Despite this progress, current SL inhibitors are often hampered by poor aqueous solubility, rapid soil degradation, low utilization efficiency, and high production costs, limiting their field application.

To overcome these translational barriers, pesticide delivery systems based on functional polymeric materials have emerged. Among them, hydrogel technology is particularly attractive due to its unique three-dimensional (3D) network and highly tunable physicochemical properties^23,24^. As hydrophilic polymers, hydrogels exhibit high water retention, excellent biocompatibility, and biodegradability, and can respond to environmental stimuli (e.g., pH, temperature, enzymes) for smart regulation of pesticide release^24–27^. Their 3D network enables efficient encapsulation of active ingredients, while parameters like mesh size and crosslinking density can be precisely controlled to fine-tune release kinetics^28,29^. Compared to conventional formulations, hydrogel delivery systems can significantly prolong pesticide efficacy and enable on-demand release, thereby spatiotemporally enhancing utilization efficiency and mitigating environmental impact^30,31^. Consequently, smart hydrogels offer an innovative solution for improving pesticide efficiency and promoting sustainable agriculture.

Natural polysaccharide-based hydrogels have garnered significant attention in pesticide delivery due to their stimulus-responsiveness, biocompatibility, biodegradability, and abundant availability^32^. For instance, carboxymethyl cellulose hydrogels enable pH-responsive release of thiamethoxam and curcumin, while starch-based hydrogels loaded with carbendazim demonstrate pH-sensitive release and water retention^33–35^. Sodium alginate/cassava starch composite hydrogel microspheres exhibit redox/pH dual-responsive characteristics for controlled dinotefuran release, markedly extending efficacy^36^. Reported tragacanth gum polysaccharide-based and water hyacinth cellulose-based hydrogels are also biodegradable in soil^37,38^. Furthermore, materials like chitosan, cyclodextrin, and alginate are commonly used to construct hydrogel delivery systems^39–41^. Carboxymethyl chitosan (CMCS), a water-soluble chitosan derivative, retains favorable biocompatibility, biodegradability, and low toxicity while offering significantly improved solubility. Its abundant functional groups (–NH_2_, –COOH, –OH) provide multiple sites for functionalization and hydrogel formation^42,43^. Studies show that CMCS hydrogels can encapsulate spinosad, enabling pH/temperature dual-responsive release to overcome the photolability of biopesticides^44^, and load prothioconazole, combining pH-responsive release with nutrient supply for reduced application and increased yield^45^. Owing to their stimuli-responsiveness and biodegradability, CMCS hydrogels represent a highly promising functional carrier for controlled pesticide release.

Metal coordination-based stimuli-responsive delivery systems hold promise for smart agriculture. Recent studies report zinc-phenolic networks (e.g., CAFZn) for controlled release of plant growth regulators^46^, iron-tannic acid core–shell structures (Phelm) for smart seed coating^47^, and zeolitic imidazolate frameworks (ZIF-8) as nanocarriers for biostimulants and micronutrients while inhibiting *Striga* germination^48,49^. These systems enable abiotic stress response and seed nano-priming, promoting early development and stress tolerance. In this context, we constructed a Mn^2+^-crosslinked CMCS hydrogel using the essential micronutrient Mn^2+^ as a crosslinker. Although natural polysaccharide-based hydrogels are promising for pesticide delivery, their performance varies. Mn²⁺ coordination bonds reinforce polysaccharide hydrogels, overcoming burst release, low efficiency, rapid degradation, and poor mechanical strength of starch-based and alginate/cassava systems^35,36,50^. Under alkaline conditions, OH⁻ competitively disrupts coordination bonds, thereby enabling on-demand release—unlike conventional polysaccharide hydrogels, which have a narrow pH response and require chemical modification^45,51,52^. Mn^2+^ serves both as a crosslinker and a micronutrient, avoiding toxic crosslinkers and achieving delivery-nutrient synergy. The Mn^2+^-CMCS hydrogel seems to be low in cost, easy to prepare, fairly quick to respond, capable of sustained release, and reasonably multifunctional.

**Fig. 1:**
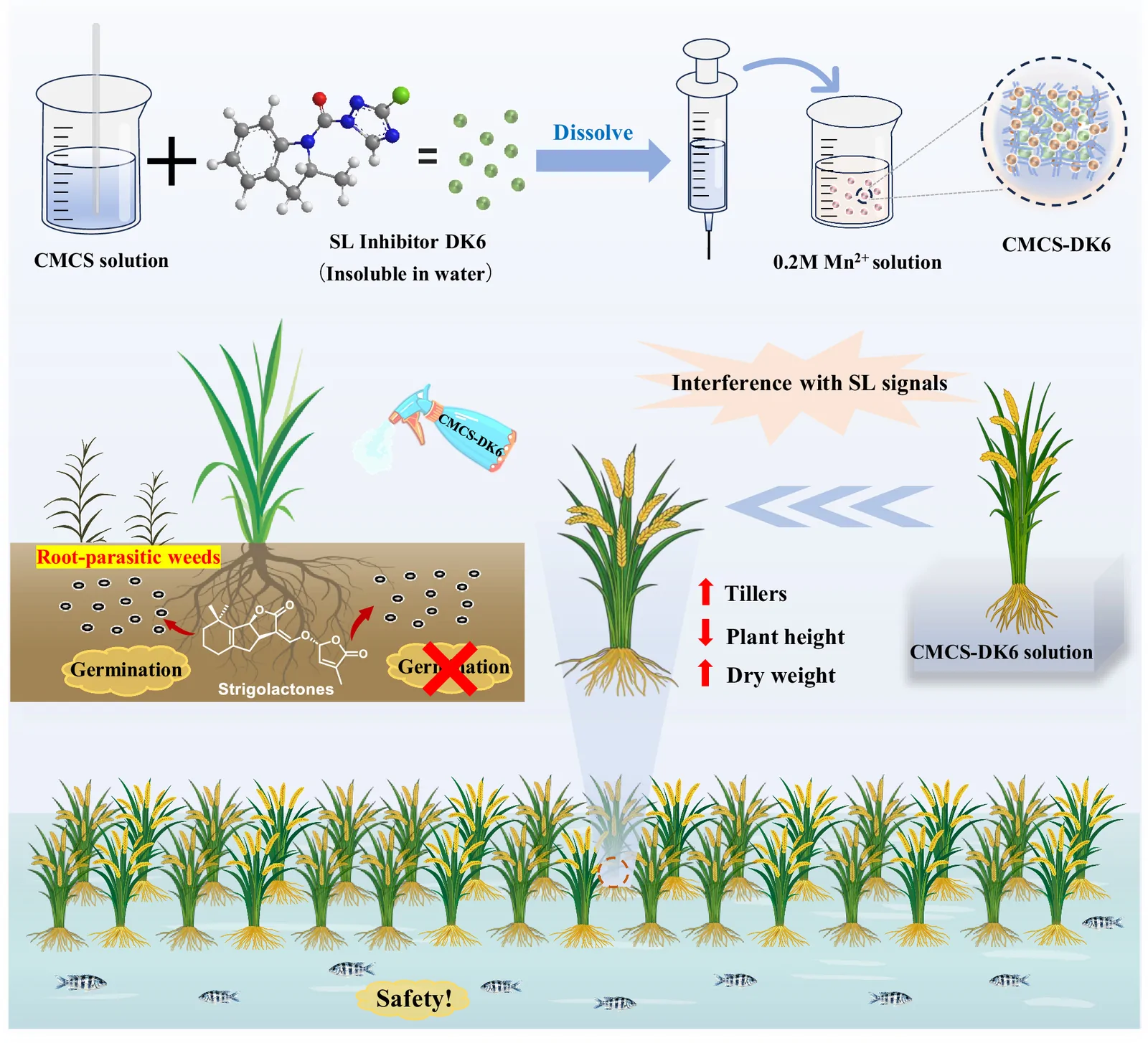
Preparation of a pH-responsive CMCS-DK6 hydrogel delivery system for synergistic rice tillering promotion and parasitic weed germination inhibition. Schematic depiction of the preparation process of the CMCS-DK6 hydrogel and its agricultural applications.

In summary, this study designed and synthesized a series of SL inhibitors, DK1-DK32, by preserving the critical triazole-urea pharmacophore in the conserved region while introducing diverse substituents in the non-conserved region. Systematic screening identified the triazole-based DK6 as the most active candidate, which was selected for supramolecular material construction. To improve its aqueous solubility and targeted delivery, a manganese-coordinated carboxymethyl chitosan (CMCS) hydrogel system was fabricated. The resulting CMCS-DK6 hydrogel enables pH-responsive release of DK6 specifically within the rhizosphere microenvironment. Biological assays demonstrated that this delivery system significantly suppresses *Striga asiatica (S. asiatica)* seed germination of the root parasitic weed Striga and enhances rice tillering, exhibiting a synergistic effect. Further computational modeling and transcriptome analysis revealed the underlying molecular mechanism responsible for the enhanced bioactivity of DK6 (Fig. 1). This Mn-coordinated CMCS hydrogel-mediated SL inhibitor delivery strategy offers an innovative approach to synchronously optimize crop architecture and achieve control of parasitic weeds, demonstrating significant potential for advancing agriculture.

## Results and discussion

### Design, synthesis, and identification of small molecule DK6

**Fig. 2:**
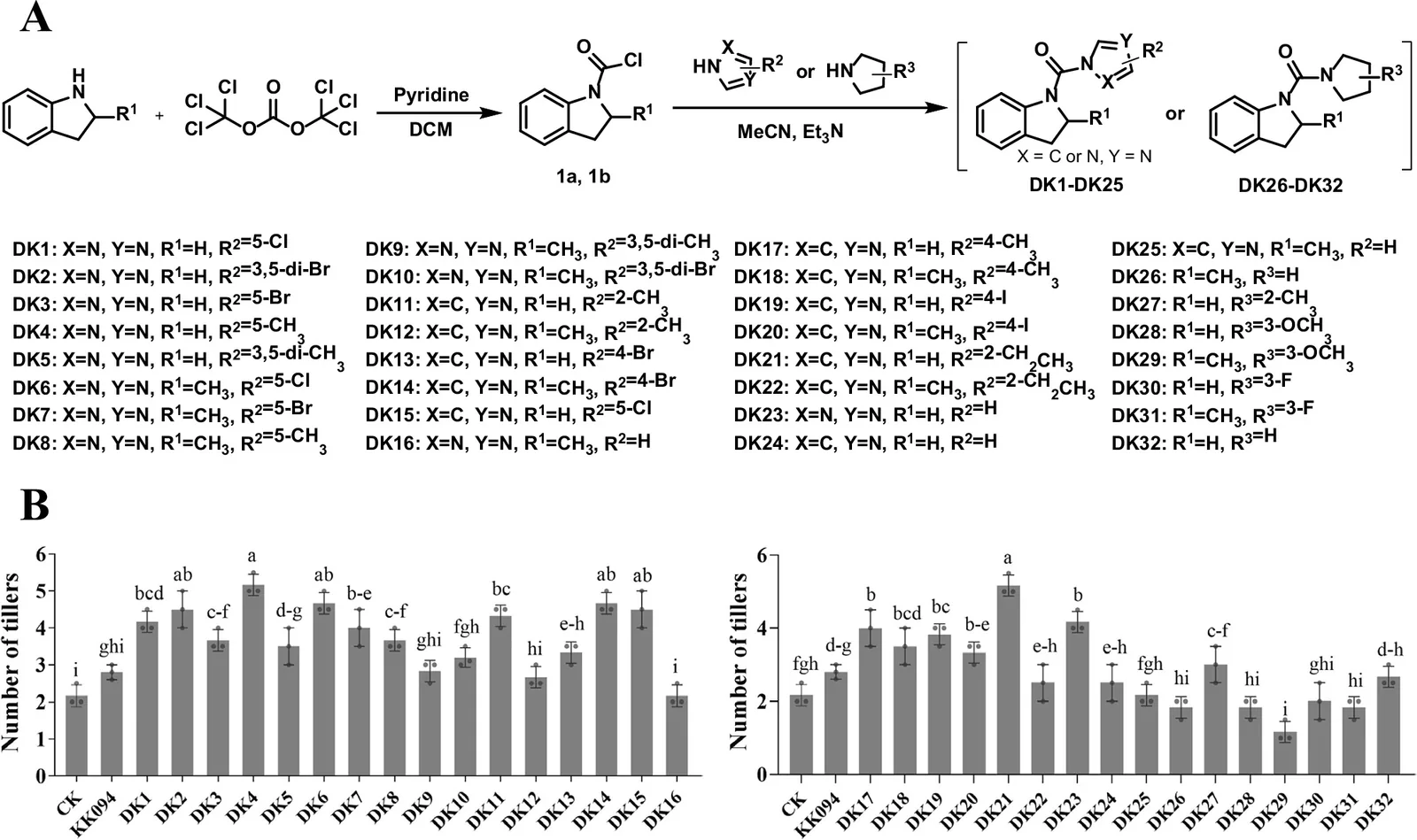
Synthesis and biological activity of the target small molecules DK1-DK32. **A** General synthetic procedure for DK1-DK32. **B** Tillering-promoting activity of DK1-DK32 on rice at a concentration of 10 µM. For **B**, error bars represent the mean ± standard deviation (SD), *n* = 3. Statistical significance among means was determined using one-way ANOVA followed by Tukey’s multiple comparison test. Different letters indicate statistically significant differences (*P* < 0.05). The experiment was repeated three times, showing similar results, with two samples per replicate. Source data are provided as a Source Data file.

Previous studies have reported that the triazole urea compound KK094, functioning as an SL antagonist, can covalently bind to the receptor D14, significantly inhibiting *Striga* germination and promoting rice tillering^17^. Indole derivatives exhibit plant growth regulation potential, attributed to the structural diversity of their core skeleton, their ability to mimic the active conformations of various endogenous hormones, specific interactions with multiple receptors, and favorable metabolic stability^53^. Furthermore, a majority of FDA-approved small-molecule drugs contain nitrogen-containing heterocycles, whose nitrogen atoms readily form hydrogen bonds with biological targets, making them prevalent features in drug candidates^54^. Leveraging these advantages, this study utilized indole as the core scaffold and employed privileged substructure hybridization with 1,2,4-triazole, imidazole, and tetrahydropyrrole rings to design and synthesize 32 small molecules, designated DK1-DK32 (Fig. 2A). The physicochemical properties, NMR, and HRMS data for all compounds are detailed in the Supplementary Information.

The germination inhibition of *S. asiatica* seeds by target compounds (80, 40, 20, 10, and 5 μM) co‑treated with 100 nM GR24 was evaluated, using KK094 as a positive control. The inhibition rates were determined, and the corresponding half-maximal inhibitory concentration (IC_50_) values were calculated. As summarized in Table 1, compounds incorporating a tetrahydropyrrole ring (DK26-DK32) generally exhibited poor inhibitory activity, with only DK26 (IC_50_ = 20.21 µM) showing activity comparable to KK094 (IC_50_ = 24.56 µM). Replacement of the tetrahydropyrrole ring with an imidazole ring also yielded limited success, with only DK16 (IC_50_ = 21.03 µM) and DK24 (IC_50_ = 23.54 µM) demonstrating good activity. Furthermore, the introduction of either electron-withdrawing or electron-donating substituents led to decreased activity. Subsequent replacement of the imidazole with a triazole structure enhanced the inhibitory potency, yielding DK23 (IC_50_ = 20.40 µM) and DK25 (IC_50_ = 17.17 µM). Building on the advantageous triazole scaffold, DK23 and DK25 were further optimized. Introduction of an electron-donating methyl group reduced activity, whereas substitution with Cl or Br atoms significantly enhanced the inhibitory effect. Among these, the Cl-containing derivative DK1 (IC_50_ = 4.18 µM) displayed prominent activity. Interestingly, introduction of a methyl group at the 2-position of the indole ring, resulting in DK6 (IC_50_ = 2.25 µM), further enhanced the activity, substantially outperforming KK094.

**Table 1:** IC_50_ values of target molecules DK1-DK32 on the seed germination of *S. asiatica*. ^*a*^

Compd.	Regression equation	R^2^	IC_50_ (μM)	Compd.	Regression equation	R^2^	IC_50_ (μM)
DK1	y = 3.3226x + 2.9349	0.9901	4.18 ± 0.28 r,s	DK18	y = 1.4700x + 3.0302	0.9405	21.88 ± 0.98 m-o
DK2	y = 2.6959x + 1.8152	0.9349	15.18 ± 0.24 q	DK19	y = 1.3283x + 2.6935	0.9959	54.50 ± 1.19 e,f
DK3	y = 2.9792x + 2.5030	0.9612	6.89 ± 0.47 r	DK20	y = 0.6426x + 3.6724	0.9458	116.44 ± 2.13 b
DK4	y = 1.1271x + 3.2731	0.9685	34.06 ± 0.84 k	DK21	y = 0.6888x + 3.7682	0.9883	61.44 ± 1.77 d
DK5	y = 1.0933x + 3.4050	0.9857	28.77 ± 0.68 l	DK22	y = 0.8434x + 3.5303	0.9697	55.30 ± 0.88 e
DK6	y = 3.6060x + 3.7159	0.9663	2.25 ± 0.16 s	DK23	y = 1.9258x + 2.4780	0.9225	20.40 ± 0.75 n-p
DK7	y = 3.2906x + 3.3013	0.9840	3.28 ± 0.24 s	DK24	y = 1.7166x + 2.6450	0.9411	23.54 ± 0.68 m,n
DK8	y = 1.1913x + 2.9521	0.9757	52.37 ± 1.03 e,f	DK25	y = 2.4774x + 4.5398	0.9190	17.17 ± 0.87 p,q
DK9	y = 0.8639x + 3.4995	0.9317	54.57 ± 0.64 e,f	DK26	y = 1.5703x + 2.9499	0.9671	20.21 ± 0.62 o,p
DK10	y = 0.7156x + 3.6181	0.9929	85.31 ± 1.63 c	DK27	y = 0.8440x + 3.6482	0.9902	39.97 ± 1.01 h,i
DK11	y = 0.6887x + 3.6771	0.9712	83.37 ± 1.25 c	DK28	y = 0.9423 + 3.3813	0.9631	52.21 ± 0.85 f
DK12	y = 0.4508x + 4.0431	0.9938	132.63 ± 1.25 a	DK29	y = 0.8763x + 3.6322	0.9881	36.38 ± 0.51 j,k
DK13	y = 0.6230x + 3.8905	0.9714	60.38 ± 1.64 d	DK30	y = 0.9730x + 3.4765	0.9924	36.80 ± 0.80 i-k
DK14	y = 1.1664x + 3.1540	0.9364	38.26 ± 1.18 h-j	DK31	y = 0.9063x + 3.4952	0.9981	45.75 ± 0.80 g
DK15	y = 2.1039x + 1.3276	0.9935	55.66 ± 1.31 e	DK32	y = 0.9511x + 3.4732	0.9110	40.30 ± 1.57 h
DK16	y = 1.6951x + 2.7577	0.9841	21.03 ± 0.62 n,o	KK094^b^	y = 1.1193x + 3.4439	0.9463	24.56 ± 0.77 m
DK17	y = 0.8112x + 3.4305	0.9534	86.08 ± 1.46 c				

^a^ The IC_50_ values, reflecting the effect of inhibiting weed seed germination, are shown as the mean ± standard deviation (SD), *n* = 3.

^b^ The SL inhibitor KK094 was used as a positive control. In assessing the inhibitory activity of test compounds on *S. asiatica* seed germination, treatment with 100 nM GR24 alone served as the control (resulting in 67.5% germination, defined as 0% inhibition). Test compounds were applied together with 100 nM GR24, and the inhibition rate was calculated as the relative reduction in germination percentage compared to the control. Except for DK1, DK6, and DK7 (0.3125-80 μM), all other compounds were tested at 5–80 μM. Statistical significance among means was determined using one-way ANOVA followed by Tukey’s multiple comparison test. Different letters indicate statistically significant differences (*P* < 0.05). The experiment was repeated three times, showing similar results (*n* = 3). Source data are provided as a Source Data file.

In rice tillering assays conducted at 10 µM, most compounds exhibited promising tillering-promoting activity (Fig. 2B). Analysis of their structure-activity relationship revealed that compounds featuring triazole or imidazole scaffolds generally demonstrated superior efficacy over tetrahydropyrrole derivatives, a trend consistent with their inhibitory effects on the germination of *S. asiatica*. Specifically, DK1 (*P* = 0.0023), DK2 (*P* = 0.0001), DK4 (*P* = 0.0001), DK7 (*P* = 0.0124) (triazole class), DK11 (*P* = 0.0004), and DK21 (*P* = 0.0001) (imidazole class) all promoted tillering more effectively than the positive control KK094. However, despite increasing tiller number, DK4 and DK21 also suppressed plant height and root length, which could be detrimental to normal plant development. Notably, DK6 (*P* = 0.0001), DK14 (*P *= 0.0001), and DK15 (*P* = 0.0001), which bear electron-withdrawing groups (Cl or Br), exhibited significantly enhanced tillering-promoting effects compared to KK094, with DK6 performing most prominently. Furthermore, DK6 also demonstrated a greater capacity to reduce plant height and increase plant dry weight relative to KK094, suggesting its potential application in rice dwarf breeding programs (Supplementary Figs. 1 and 2). Dose-response experiments confirmed that DK6 maintained tillering-promoting activity across a range of concentrations (Supplementary Fig. 3A). These results collectively validate the rationality of our molecular design strategy. Owing to its optimal combined performance in both inhibiting *S. asiatica* germination and promoting rice tillering, DK6 was selected as the lead candidate for the subsequent construction of supramolecular materials.

### Preparation of CMCS-DK6 hydrogel

**Fig. 3:**
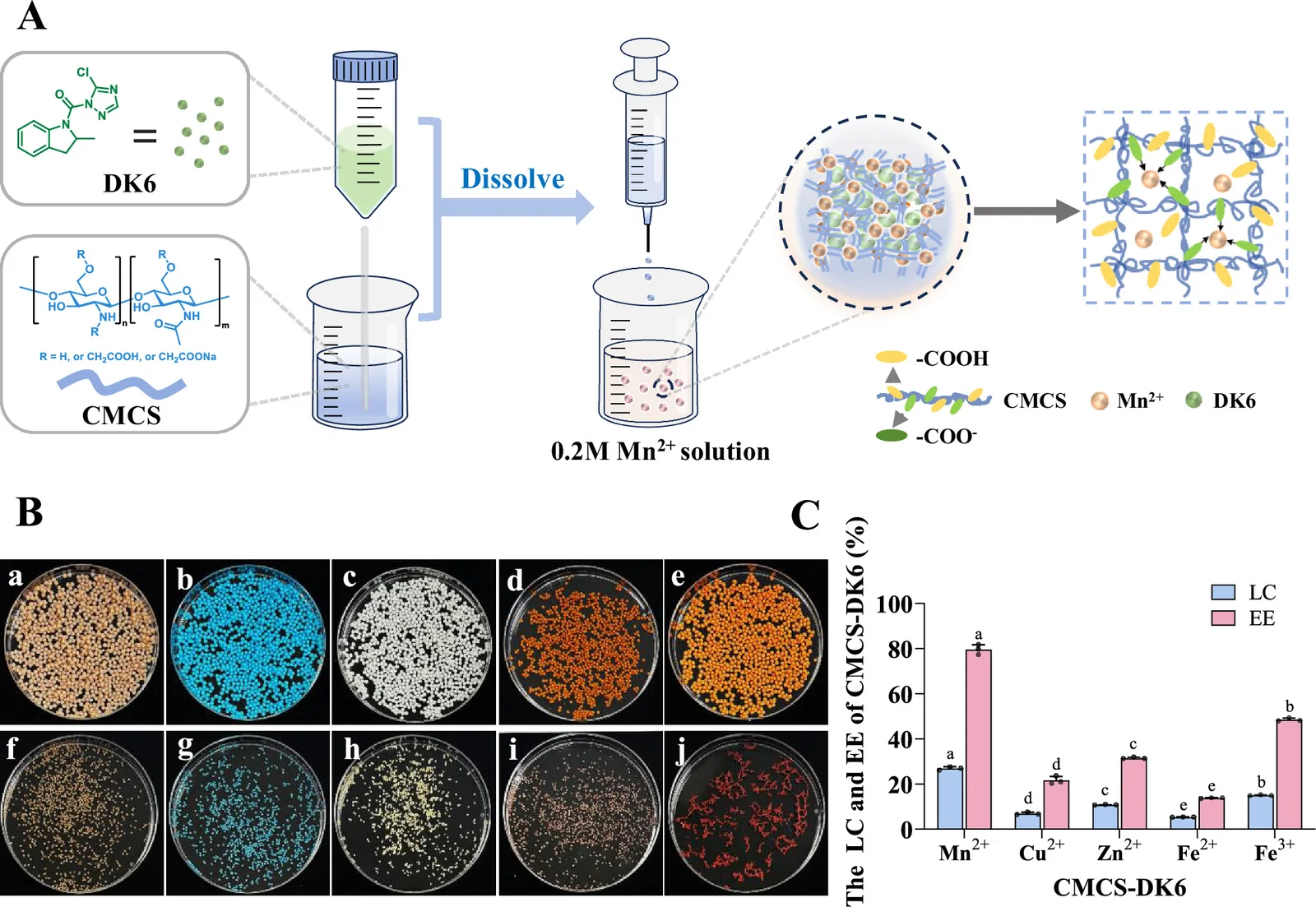
Preparation of the CMCS-DK6 Hydrogel. **A** Schematic diagram illustrating the preparation of the CMCS-DK6 hydrogel. **B** Photographs of DK6-loaded hydrogels before and after drying, cross-linked with different metal ions: Mn^2+^ (a, f), Cu^2+^ (b, g), Zn^2+^ (c, h), Fe^2+^ (d, i), Fe^3+^ (e, j). **C** Loading capacity (LC) and encapsulation efficiency (EE) of CMCS-DK6 hydrogels cross-linked with different metal ions. For **C**, error bars represent the mean ± standard deviation (SD). Statistical significance among means was determined using one-way ANOVA followed by Tukey’s multiple comparison test. Different letters indicate statistically significant differences (*P* < 0.05). The experiment was repeated three times, showing similar results (*n* = 3). Source data are provided as a Source Data file.

Recent studies using systems such as Zn-phenolic networks, Fe-tannic acid shells, and ZIF-8 have achieved controlled release of kinetin, seed coating, modulation of signaling molecules, and co-delivery of nutrients, demonstrating the potential of metal coordination chemistry in constructing tunable agricultural delivery platforms^46–49^. Building on this, we selected the essential plant micronutrient Mn^2+^ to coordinate with CMCS—a soluble, biocompatible, and degradable polymer—to encapsulate the hydrophobic compound DK6, forming a CMCS-DK6 hydrogel delivery system aimed at improving solubility, water absorption, and retention, thereby enhancing drug delivery performance (Fig. 3A).

The amino, carboxyl, and hydroxyl groups within CMCS molecules can form supramolecular hydrogels through chelation with metal ions. The core mechanism involves positively charged metal ions acting as cross-linkers, forming “ionic bridges” with the negatively charged -COO⁻ groups of CMCS, thereby constructing a three-dimensional network structure of the hydrogel^55^. Given that essential plant trace elements such as Zn^2+^, Cu^2+^, Mn^2+^, and Fe^2+^/Fe^3+^ can all chelate with CMCS to form hydrogels, these ions were selected as cross-linkers for the preparation of DK6-loaded CMCS hydrogels (Fig. 3A, B). Using Mn^2+^ as an example, the hydrogel preparation process is as follows: first, DK6 was dispersed in an aqueous CMCS solution via an emulsification method. The resulting emulsion was then loaded into a 10 mL syringe and added dropwise into an aqueous solution of metal ions, triggering an instantaneous ion crosslinking reaction. This reaction, based on coordination and ion exchange between CMCS and metal ions, leads to hydrogel formation. During the screening of suitable cross-linking ions, it was observed that Cu^2+^ and Fe^2+^ produced precipitates upon mixing with the DK6 solution, whereas the Zn^2+^, Mn^2+^, and Fe^3+^ systems remained homogeneous and clear (Supplementary Fig. 3B). This indicates that Cu^2+^ and Fe^2+^ are unsuitable for the preparation of this drug-loaded hydrogel, which is consistent with their correspondingly lowest drug loading capacity and encapsulation efficiency. Among the tested ions, the hydrogel cross-linked with Mn^2+^ demonstrated superior drug loading performance, with a drug loading capacity (LC) and encapsulation efficiency (EE) of 26.9% (*P* = 0.0001) and 79.6% (*P* = 0.0001), respectively (Fig. 3C). Nevertheless, there remains room for further improvement in encapsulation efficiency. Future efforts could focus on optimizing emulsification-crosslinking parameters to enhance the loading capacity for small molecules, as well as introducing co-crosslinking systems or multi-responsive architectures to improve the stability of the encapsulated payload, thereby achieving higher drug loading efficiency.

### Characterization of CMCS-DK6 hydrogel

**Fig. 4:**
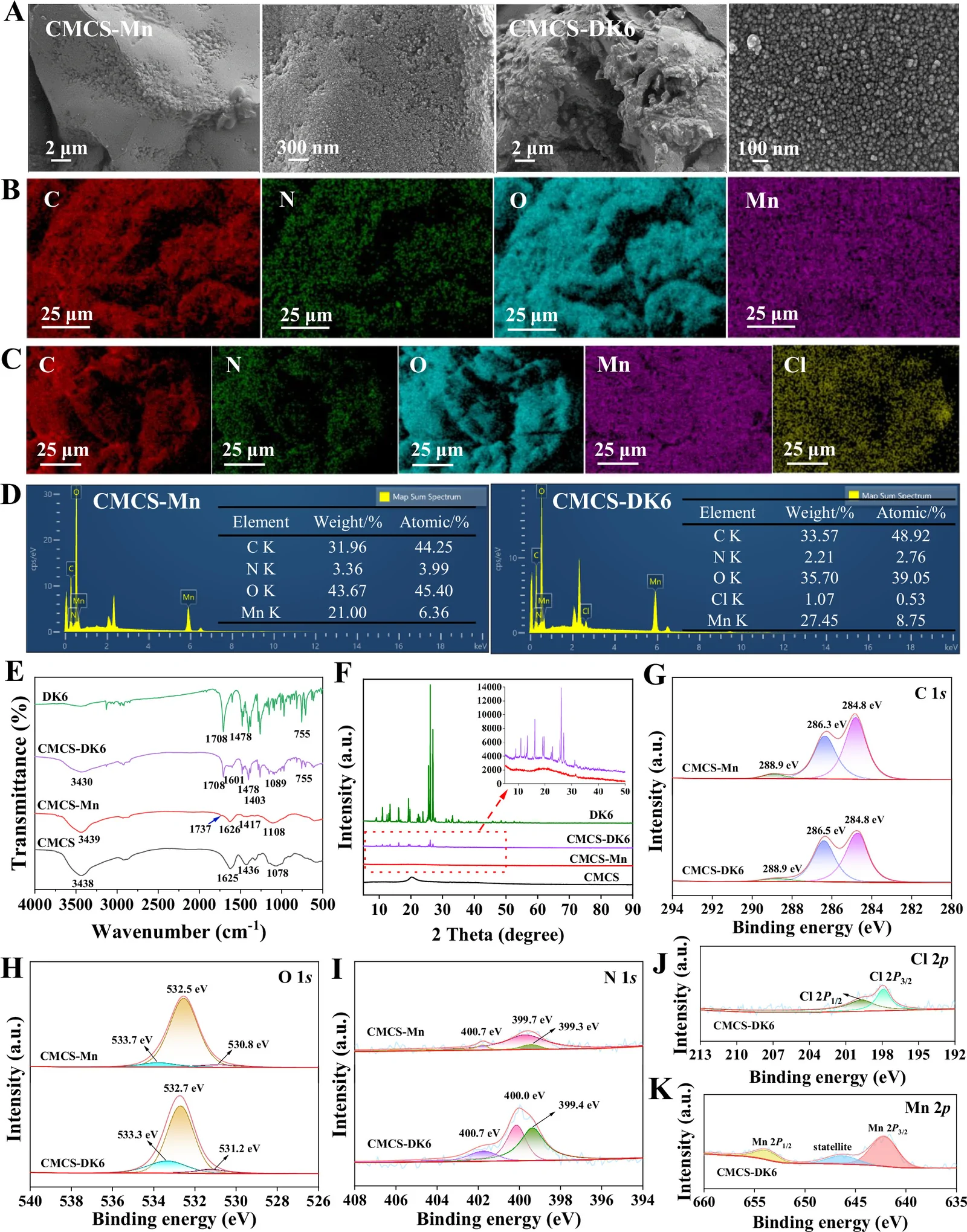
Characterization of the CMCS-DK6 Hydrogel. **A** SEM image. **B**, **C** elemental mapping. **D** EDS spectrum of CMCS-Mn and CMCS-DK6. **E** FT-IR spectra. **F** XRD patterns of CMCS, CMCS-Mn, CMCS-DK6, and DK6. **G–K** XPS spectra of CMCS-Mn and CMCS-DK6. Source data are provided as a Source Data file.

To confirm the successful preparation of hydrogel particles, a series of structural characterizations was performed. Compared with CMCS-Mn, the high-magnification Scanning electron microscopy (SEM) image of CMCS-DK6 hydrogel particles (Fig. 4A) revealed the presence of DK6 crystals on their surface. As shown in Fig. 4B–D, elemental mapping images further disclosed the uniform distribution of Mn and Cl elements within CMCS-DK6. Moreover, the EDS spectrum exhibited a characteristic Cl peak absent in CMCS-Mn. These results collectively confirm the successful loading of DK6 into the hydrogel network.

Fourier transform infrared (FT-IR) spectroscopic analysis (Fig. 4E) indicated that CMCS displays a strong and broad absorption band at 3438 cm^−1^, attributed to the stretching vibrations of O–H and N–H bonds^56^, while the stretching vibration absorption peak of C–OH is located at 1078 cm^−1^. In CMCS-Mn, the latter shifted to 1108 cm^−1^, suggesting potential interactions between –OH groups and Mn^2+^ ions as well as –COO⁻ groups. The asymmetric and symmetric stretching vibration peaks of –COOH in CMCS appear at 1625 cm^−1^ and 1436 cm^−1^, respectively^57^. After hydrogel formation, the characteristic absorption peak of –COO⁻ at 1436 cm^−1^ shifted to 1417 cm^−1^ and 1403 cm^−1^ in CMCS-Mn and CMCS-DK6, respectively, while the peak at 1625 cm^−1^ shifted to 1626 cm^−1^ and 1601 cm^−1^, respectively. These shifts suggest that, in addition to the coordination bonding between Mn^2+^ and –COO⁻, hydrogen bonding interactions might also exist between CMCS and DK6. This is plausible because CMCS contains numerous –OH groups, whose oxygen lone pair electrons can readily coordinate with metal ions, and the hydrogen atoms can form hydrogen bonds with –COO⁻ groups. Notably, a new peak emerged at 1737 cm^−1^ in CMCS-Mn, attributed to the asymmetric stretching vibration of –C = O, further confirming the ionic cross-linking between Mn^2+^ and the carboxyl groups of CMCS^58^. DK6 exhibits characteristic absorption peaks at 1708 cm^−1^ (ketone carbonyl stretching vibration), 1478 cm^−1^ (aromatic ring skeletal vibration), and 755 cm^−1^ (C–Cl stretching vibration). All these characteristic absorptions are clearly visible in the spectrum of CMCS-DK6, indicating the successful loading of DK6 into the hydrogel carrier.

Simultaneously, X-ray diffraction (XRD) was employed to characterize the crystalline behavior of CMCS, CMCS-Mn, DK6, and CMCS-DK6, with the results shown in Fig. 4F. DK6 exhibited distinct diffraction peaks at 2θ = 11.0°, 13.4°, 16.1°, 19.2°, 25.5°, 26.1°, and 26.9°, attributable to its high crystallinity. CMCS showed a sharp, strong diffraction peak at 2θ = 20.5°. However, in the CMCS-DK6 hydrogel, this characteristic diffraction peak significantly weakened or even disappeared, indicating a transition from a crystalline to a disordered amorphous structure. This reduction in crystallinity can be attributed to the potential hydrogen bonding interactions between CMCS and DK6 molecules, as well as the metal coordination bonds between Mn^2+^ and the –COO⁻ groups on the CMCS chains, which collectively disrupt the original ordered arrangement of the molecular chains.

To further investigate the chemical states and composition of the hydrogels, X-ray photoelectron spectroscopy (XPS) characterization was performed on the samples. The survey spectra showed all characteristic peaks, consistent with the EDS results, further confirming the successful loading of DK6. In the high-resolution C 1*s* spectrum (Fig. 4G), peaks at 284.8 eV and 288.9 eV are assigned to C–C and C = O bonds, respectively. After DK6 loading, the C–N peak shifted from 286.3 eV to 286.5 eV, indicating a change in the surrounding chemical environment^59^. In the O 1 *s* spectrum (Fig. 4H), the peaks for CMCS-Mn at 532.5 eV and 533.7 eV correspond to C–O and C = O, along with adsorbed oxygen species, respectively. These shifted to 532.7 eV and 533.3 eV in CMCS-DK6^59^. Additionally, the peak related to metal oxides at 530.8 eV also shifted, potentially associated with the coordination between Mn^2+^ and –COO⁻. The N 1*s* spectrum of CMCS-DK6 displayed three peaks at 399.4 eV, 400.0 eV, and 400.7 eV, which can be tentatively assigned to pyrrolic N, C–N, and C = N/amide N bond structures, respectively (Fig. 4I)^60^. Furthermore, the high-resolution Cl 2*p* spectrum clearly showed the doublet structure of Cl 2*p*_3/2_ (197.8 eV) and Cl 2*p*_1/2_ (199.7 eV) (Fig. 4J), further verifying the introduction of DK6^61^. In the Mn 2p spectrum (Fig. 4K), the binding energies for Mn 2*p*_3/2_ and Mn 2*p*_1/2_ are located at 642.2 eV and 653.8 eV, respectively, with a satellite vibration peak appearing at 646.3 eV, resulting from spin-orbit splitting^62^.

### pH-dependent swelling of CMCS-DK6 hydrogel

The swelling process of hydrogels is primarily governed by the combined effects of osmotic pressure and intermolecular interactions between polymer chains^63^. In this study, the hydrogel network formed by the cross-linking of CMCS with Mn^2+^ effectively regulated the swelling kinetics of the dried gel particles in an aqueous medium, exhibiting significant swelling differences under various pH conditions.

**Fig. 5:**
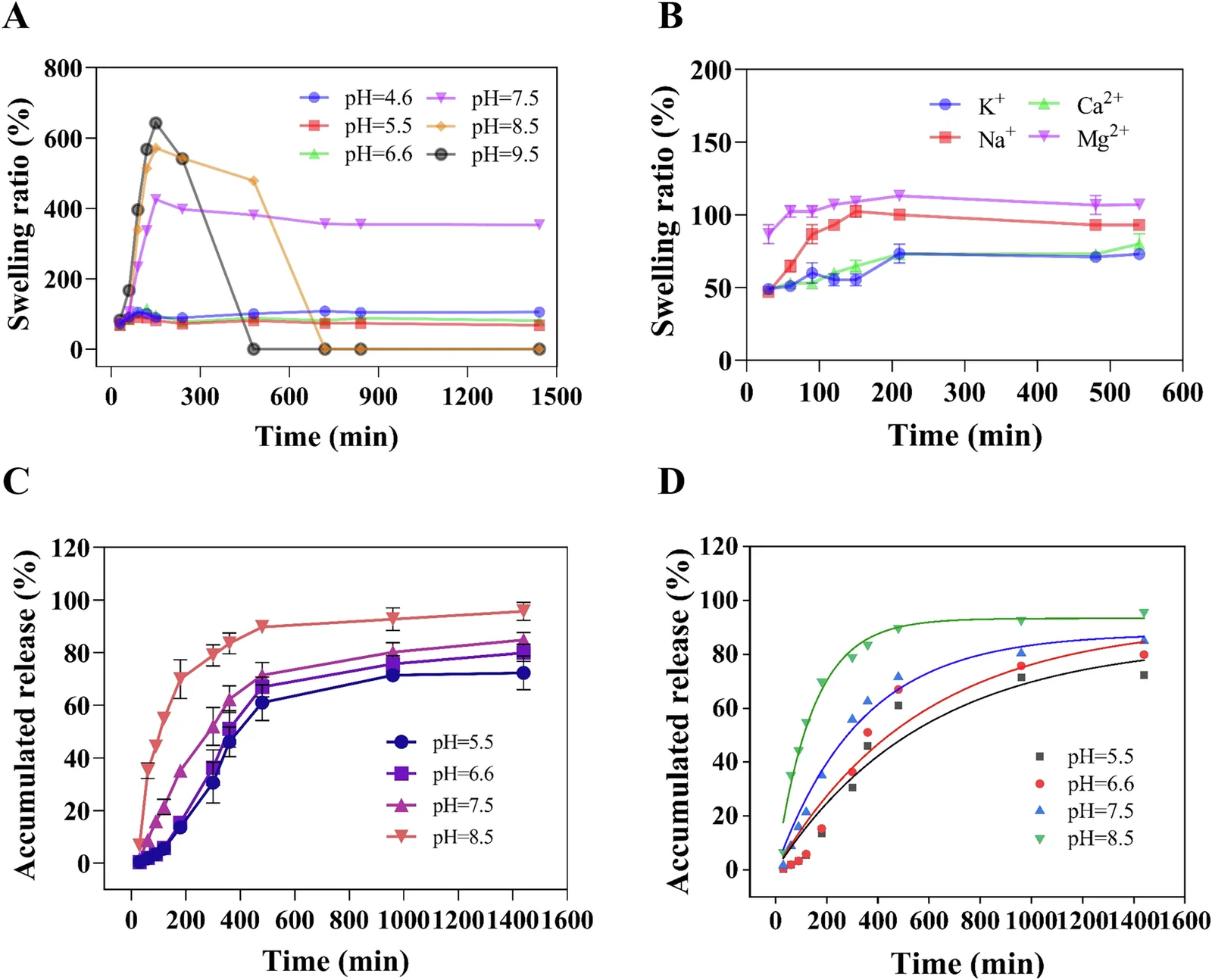
Swelling behavior and controlled release kinetics of the CMCS-DK6 hydrogel. **A** Swelling ratio of the CMCS-DK6 hydrogel at different pH values. **B** Swelling ratio of the CMCS-DK6 hydrogel in 0.02 M solutions of different ions. **C** Cumulative release profile of DK6 from the CMCS-DK6 hydrogel at different pH values. **D** Fitting of the DK6 release profiles from CMCS-DK6 at different pH values using a first-order kinetic model. For panels **A**–**C**, error bars represent the mean ± standard deviation (SD). The experiment was repeated three times, showing similar results (*n* = 3). Source data are provided as a Source Data file.

The swelling behavior of the CMCS-DK6 hydrogel under different pH conditions is shown in Fig. 5A. In acidic environments (pH = 3.6, 4.6, and 5.6), the hydrogel exhibited a low swelling ratio, and no significant changes in its macroscopic morphology were observed. In contrast, under alkaline conditions (pH = 8.5 and 9.5), the swelling reached a maximum at approximately 150 min, followed by a continuous process of swelling and structural disintegration, ultimately leading to the complete dissolution of the gel. The swelling states of the hydrogel at different time points can be visually observed in the corresponding digital photographs (Supplementary Fig. 4A). Furthermore, the swelling performance of the hydrogel in 0.02 M solutions of different inorganic salts (containing Ca^2+^, Na⁺, K⁺ or Mg^2+^) was investigated. Combining the results from Fig. 5B and Supplementary Fig. 4B, it can be seen that although the swelling degree increased slightly over time and eventually reached equilibrium, no significant differences were observed under the various ionic conditions. This indicates that, compared to the pH of the aqueous medium, the type of ions has a relatively limited influence on the gel’s swelling behavior, making the pH value of the medium the dominant factor regulating its swelling process.

The pH-responsive characteristics of the hydrogel originate from the presence of hydrophilic functional groups within its polymer chains, such as amino, carboxyl, and hydroxyl groups^64^. Under alkaline conditions, hydrogels containing anionic functional groups (–COO⁻ and –OH⁻) exhibit a significantly increased swelling ratio due to enhanced ionization, leading to strong electrostatic repulsion. Conversely, in acidic environments, hydrogels containing cationic groups (e.g., –NH^3+^) also demonstrate certain swelling behavior due to protonation^33^.

See Supplementary Figs. 4 and 5, the pH-responsive swelling behavior and its underlying mechanism reveal that in acidic environments, the –COOH groups are not fully ionized. Coordination bonds cause the polymer network of the hydrogel to contract, increasing the cross-linking density and consequently leading to a decreased swelling ratio. As the pH increases, –COOH groups gradually dissociate into –COO⁻. The increased number of anions enhances the electrostatic repulsion between chains, inducing network expansion and hydrogel swelling. Simultaneously, Mn^2+^ is strongly attracted to the –COO⁻ groups on the negatively charged CMCS chains, and its coordination effect also gradually strengthens, competing with the electrostatic repulsion. Under neutral conditions, a dynamic balance between these two opposing forces is likely reached, resulting in stabilized swelling behavior. Under alkaline conditions, –COOH groups undergo maximum ionization, and the hydrogel swelling reaches its maximum. For instance, at pH = 8.5 and 9.5, OH⁻ ions competitively inhibit the coordination stability between –COO⁻ and Mn^2+^, disrupting the cross-linked network. This process leads to an irreversible disintegration of the network after 150 min of swelling, manifested as concurrent swelling and structural rupture, ultimately resulting in the complete dissolution of the gel.

### Release behavior of CMCS-DK6 hydrogel delivery system

This study systematically evaluated the in vitro release behavior of CMCS-DK6 in PBS buffers at pH 5.5, 6.6, 7.5, and 8.5. As shown in Fig. 5C, DK6 release exhibited clear pH dependence, with both the release rate and cumulative release increasing at higher pH. At pH 8.5, the hydrogel released rapidly within 6 h, achieving a cumulative release of 95.7% at 24 h. The 6 h cumulative release rates at pH 7.5 and 8.5 were 62.4% and 83.5%, respectively, significantly higher than those at pH 6.6 (51.0%) and pH 5.5 (46.0%). Although the hydrogel network was denser under acidic conditions, restricting drug diffusion, the cumulative release at pH 5.5 still reached 72.3% at 24 h. With increasing pH, electrostatic repulsion between –COO⁻ groups intensified, leading to polymer network expansion and enhanced DK6 release.

To assess the release behavior of CMCS-DK6 in soils with varying pH, six paddy soils from Hunan, Jiangsu, Sichuan, Henan, Heilongjiang, and Liaoning were selected. As shown in Supplementary Fig. 6A, within 48 h, the cumulative release of CMCS-DK6 in these soils ranged from 63.5% to 86.1%, all reaching effective release levels. Compared with the PBS buffer system, buffering and adsorption effects in actual soil solutions attenuated the pH-dependent release profile. Furthermore, after growing rice for 45 days in the six soils supplemented with varying concentrations of CMCS-DK6, CMCS-DK6 promoted rice tillering to varying extents (Fig. 7, Supplementary, see the Supplementary Information for specific methods).

To further elucidate the release mechanism of DK6 from CMCS-DK6, the release profiles were fitted using Zero-order, First-order, Higuchi, and Ritger-Peppas models. As shown in Fig. 5D, Supplementary Fig. 6B, Supplementary Tables 1 and 2, the release kinetics of CMCS-DK6 hydrogels conformed better to the first-order model, primarily because the release rate was driven by the drug concentration gradient and decreased with residual drug content. Under alkaline conditions, hydrogel swelling facilitated drug diffusion, but the overall release remained diffusion-controlled. In PBS buffer, matrix erosion contributed to release at high pH, and the overall release behavior agreed well with the first-order model (*R*^2^ = 0.91–0.97). Similarly, in soil solutions, the release mechanism followed first-order kinetics (*R*^2^ = 0.94–0.99). These results confirm that the release rate is proportional to the residual drug amount, resulting from the synergistic effects of swelling, diffusion, and erosion.

In summary, CMCS-DK6 hydrogels exhibited pH-responsive behavior in PBS buffer, whereas in actual soil solutions, the regulatory effect of pH on release was weakened by buffering and adsorption. Nevertheless, the release process followed first-order kinetics in both media, indicating that the drug release rate is proportional to the residual drug amount, providing a theoretical basis for time-based prediction of release in field applications. Future studies should further quantify the influence of soil physicochemical properties on release kinetics to optimize the application strategy of hydrogels in real soil environments.

### Bioactivity

**Fig. 6:**
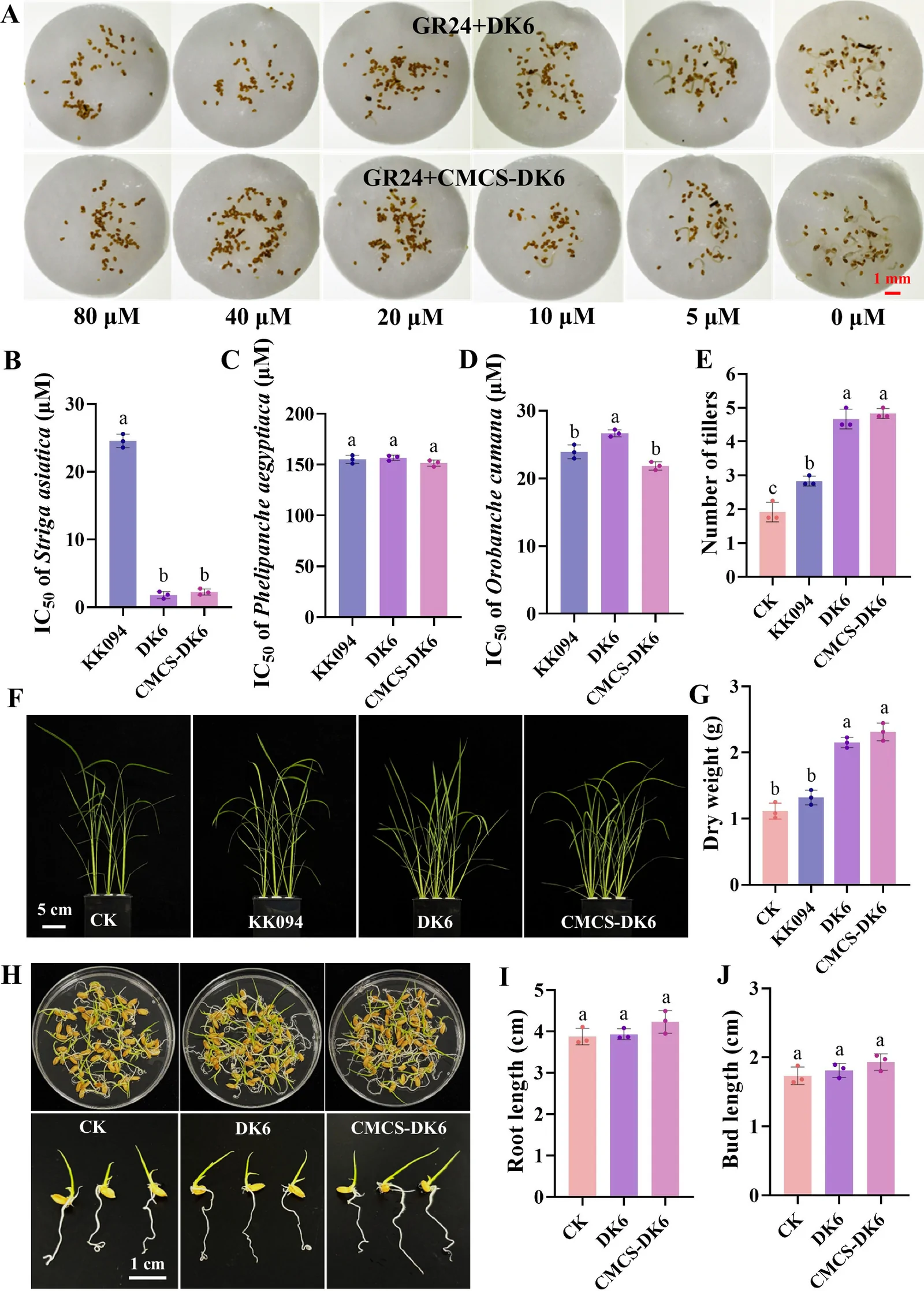
Inhibition of root parasitic weeds and promotion of rice tillering by CMCS-DK6 hydrogel. The germination rates induced by 100 nM GR24 alone for *Striga asiatica*, *Phelipanche aegyptiaca*, and *Orobanche cumana* were 67.5, 95.5 and 70.9%, respectively. When different concentrations of the blank hydrogel CMCS-Mn lacking DK6 (80, 40, 20, 10 and 5 μM) were co-applied with 100 nM GR24, the germination rates of *S. asiatica* (66.5–67.1%), *P. aegyptiaca* (93.1–94.0%) and *O. cumana* (69.3–69.8%) were comparable to those obtained with 100 nM GR24 treatment alone. Using the germination rate in the presence of 100 nM GR24 alone as the positive control (0% inhibition), the inhibition rate was calculated based on the relative reduction in germination observed in the “test compound + 100 nM GR24” treatment groups. Co-application of CMCS-DK6 hydrogel with 100 nM GR24 resulted in suppression of parasitic weed germination. **A** representative image showing inhibition of *S. asiatica* seed germination. The GR24 concentration was fixed at 100 nM, and the indicated concentrations (80, 40, 20, 10, 5 and 0 μM) correspond to DK6 or CMCS-DK6. IC_50_ values for germination inhibition of **B** *S. asiatica*, **C** *P. aegyptiaca*, and **D** *O. cumana* by the compounds KK094, DK6, and CMCS-DK6 in the presence of 100 nM GR24. The tested concentration range of DK6/CMCS-DK6 in *S. asiatica* is 0.3125-80 μM. Effects of 10 μM CMCS-DK6 hydrogel on rice (*Oryza sativa*), with an aqueous solution as the control (CK) and a DMSO content of <0.1%. **E**, **F** Promotion of rice tillering activity, **G** dry weight, **H** rice seed germination, **I** root length, and **J** shoot length. For panels **B**–**G**, **I** and **J**, error bars represent the mean ± standard deviation (SD). Statistical significance among means was determined using one-way ANOVA followed by Tukey’s multiple comparison test. Different letters indicate statistically significant differences (*P* < 0.05). The experiment was repeated three times, showing similar results (*n* = 3). Source data are provided as a Source Data file.

The inhibitory efficacy of the CMCS-DK6 hydrogel on root parasitic weed seed germination is shown in Fig. 6A–D. Compared to free DK6 (IC₅₀ = 2.25 µM, *P* = 0.0001), CMCS-DK6 exhibits a stronger inhibitory effect on *S. asiatica* germination (IC₅₀ = 1.80 µM, *P* = 0.0001), markedly outperforming KK094 (IC_50_ = 24.56 µM). Further results show that CMCS-DK6 inhibits the germination of *Phelipanche aegyptiaca* (*P. aegyptiaca*) and *Orobanche cumana* (*O. cumana*) with IC₅₀ values of 151.52 µM (*P* = 0.4117) and 21.85 µM (*P* = 0.0324), respectively, demonstrating comparable efficacy to KK094 (IC_50_ = 155.21 µM and 23.93 µM, respectively). The CMCS hydrogel matrix likely enhances DK6 bioactivity, potentially due to its excellent biocompatibility and sustained-release properties, which stabilize DK6 interaction with weed seeds and prolong its duration of action.

Concurrently, at 10 µM, CMCS-DK6 promotes rice tillering slightly more effectively than free DK6, with both surpassing KK094 in tillering promotion (*P* = 0.0001) and dry weight increase (*P* = 0.0001) (Fig. 6E–G). We speculate that the weakly acidic environment of rice growth may limit the hydrogel’s sustained-release profile, preventing the full realization of its efficacy advantage. Nevertheless, both CMCS-DK6 and DK6 combine plant height reduction with increased dry weight, providing a solid foundation for optimizing plant architecture and developing high-yielding, lodging-resistant rice cultivars. This CMCS-based hydrogel formulation offers a strategy for controlling root parasitic weeds and holds promise for mitigating crop damage in agricultural production.

Furthermore, to investigate their effects on early rice growth, rice seeds were cultivated in 10 μM solutions of DK6 and CMCS-DK6 for five days. As shown in Fig. 6H–J, no significant differences were observed in germination rate, root length (*P* = 0.1784, *P* = 0.2679), or shoot length (*P* = 0.1677, *P* = 0.4541) between the CMCS-DK6-treated group and the control CK or DK6-treated groups. These results indicate that, at this concentration, the CMCS-DK6 hydrogel does not exert any adverse effects on rice seedling growth and exhibits favorable biosafety.

### Analysis of *A. thaliana* Hypocotyl Elongation

**Fig. 7:**
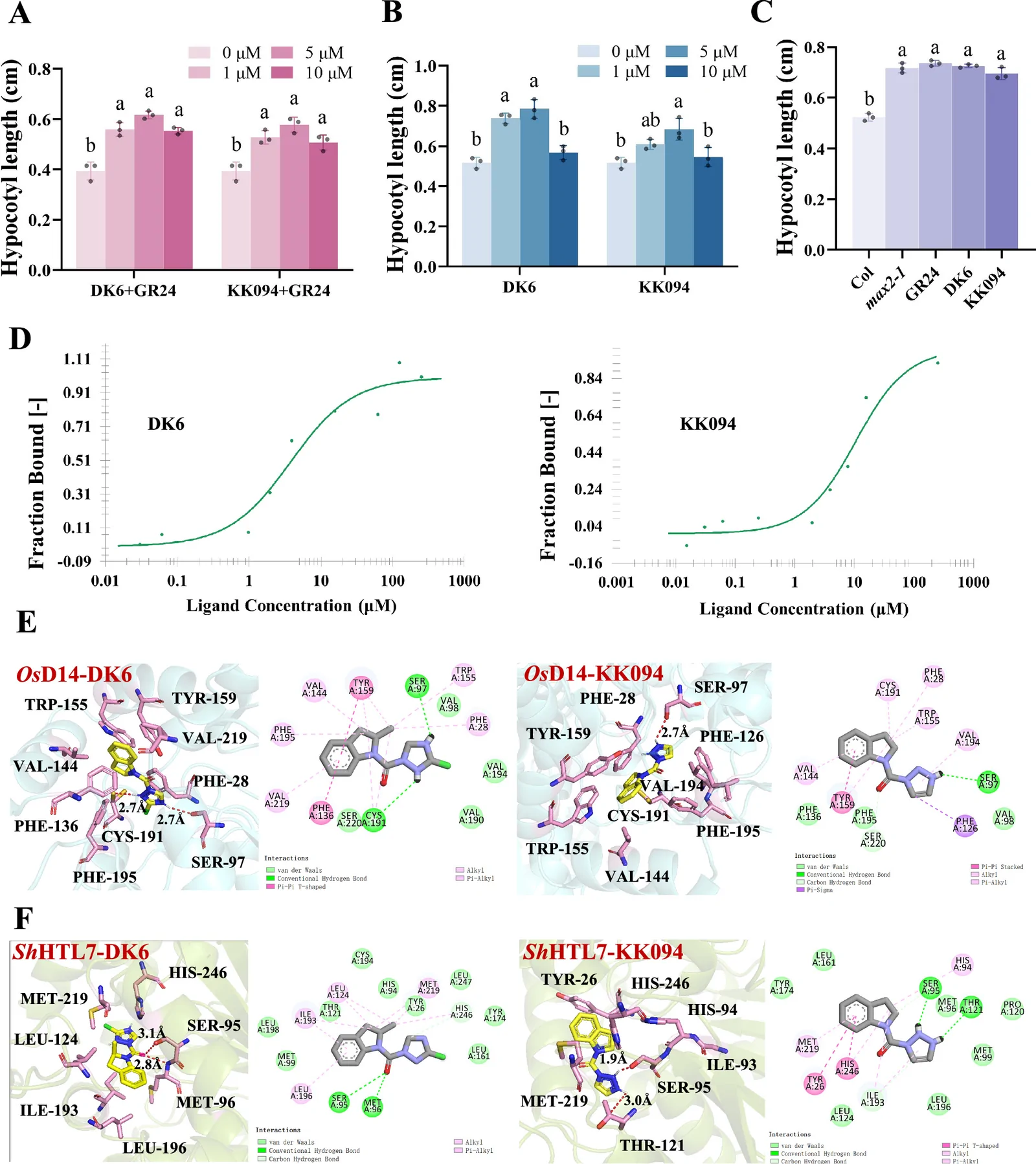
Biological activity and molecular interactions of DK6. Effect of DK6 on hypocotyl elongation in *A. thaliana*. **A** Wild-type *A. thaliana Col-0* treated with DK6. **B** Wild-type *A. thaliana Col-0* treated with DK6 in combination with 3 μM GR24. **C** SL signaling mutant *max2-1* treated with DK6. All data are presented as the mean ± SD from at least three independent replicates. **D** Microscale thermophoresis results of compounds DK6 and KK094 with *At*D14 (SL receptor in *A. thaliana*). Molecular docking of DK6/KK094 with the SL receptor proteins **E** *Os*D14 and **F** *Sh*HTL7. The ligand is shown in yellow, and key residues are in light purple. For panels **A**–**C**, error bars represent the mean ± standard deviation (SD). Statistical significance among means was determined using one-way ANOVA followed by Tukey’s multiple comparison test. Different letters indicate statistically significant differences (*P* < 0.05). The experiment was repeated three times, showing similar results (*n* = 3). Source data are provided as a Source Data file.

This study utilized *A. thaliana* (*Col-0*) and the SL signaling mutant *max2-1* to systematically evaluate the effect of DK6, the active component in CMCS-DK6 hydrogel, on hypocotyl elongation, with the aim of exploring its relationship with the SL signaling pathway. Based on the established mechanism that SL inhibits hypocotyl elongation in a MAX2-dependent manner^65^, we hypothesized that DK6 might function as an SL signaling inhibitor. As illustrated in Fig. 7A–C, in wild-type *A. thaliana*, DK6 treatment at various concentrations significantly promoted hypocotyl elongation, outperforming KK094. In the presence of GR24 (3 μM), both DK6 and KK094 effectively reversed the GR24-induced suppression of hypocotyl elongation, suggesting that DK6 may function by interfering with the SL signaling pathway^15^. The *max2-1* mutant exhibited longer hypocotyls and was insensitive to GR24. Consistent with expectations, DK6 did not further promote hypocotyl elongation in *max2-1*, a response pattern identical to that observed with KK094.

### Microscale thermophoresis analysis

The binding affinities of small molecules DK6 and KK094 to the *A. thaliana* SL receptor *At*D14 were determined using microscale thermophoresis (MST), as the rice SL receptor *Os*D14 and the *Striga* SL receptor *Sh*HTL7 were unavailable. As shown in Fig. 7D, the dissociation rate constant (K_d_) of DK6 for *At*D14 was 3.72 ± 1.509 μM, whereas that of KK094 was 10.55 ± 3.508 μM, indicating a higher affinity of DK6 for *At*D14. These results demonstrate that DK6 effectively interacts with *At*D14 with high binding affinity. Combining genetic evidence with MST binding assays, DK6 likely disrupts SL signaling through direct binding to the SL receptor *At*D14, thereby relieving hypocotyl elongation suppression in a MAX2-dependent manner. It should be noted that while MST analysis confirmed the direct binding of DK6 to *At*D14, direct binding assays with *Os*D14 and *Sh*HTL7 recombinant proteins were not performed in the current study due to the limited availability of these proteins. Given the high sequence and structural conservation of the SL receptor family, the binding affinity observed with *At*D14 supports the notion that DK6 likely interacts with other SL receptors as well. However, direct biochemical evidence for DK6 binding to *Os*D14 and *Sh*HTL7, as well as genetic evidence from corresponding knockout lines, will be essential to fully validate the molecular mechanism and will be pursued in future investigations.

### Molecular docking

Molecular docking is a valuable computational method for predicting ligand-receptor binding modes and elucidating key intermolecular interactions. This study focuses on the SL receptors—*Os*D14 in rice and *Sh*HTL7 in the parasitic weed *Striga*—systematically comparing the binding characteristics of ligands DK6 and KK094. In *Os*D14 (Fig. 7D), DK6 exhibits a binding energy of −9.5 kcal/mol, significantly lower than that of KK094 (−8.2 kcal/mol), indicating more stable binding and higher affinity. DK6 achieves effective polar anchoring via two hydrogen bonds (both 2.7 Å) formed between nitrogen atoms on its triazole ring and residues SER-97 and CYS-191. Furthermore, the ligand is fully embedded within a hydrophobic pocket composed of aromatic and aliphatic residues such as TRP-155, TYR-159, and VAL-144, along with residues from the receptor subunit (TRP-155, PHE-28), forming a dual hydrophobic layer that extensively encloses the ligand via van der Waals interactions, thereby minimizing conformational entropy changes. This stabilization is further enhanced by Pi-Alkyl interactions with PHE-136 and VAL-219. In contrast, although KK094, as a known active ligand, engages in Pi-Pi stacking with PHE-126 and Pi-Sigma interactions with TYR-159, and forms a hydrogen bond with SER-97 (2.7 Å) along with hydrophobic contacts with VAL-144 and PHE-195, these interactions are nevertheless less extensive than those observed for DK6. Consequently, the computed binding energy of DK6 is more favorable than that of KK094, indicating a relatively weaker binding affinity of KK094 in our docking analysis.

As shown in Fig. 7E, DK6 exhibits a binding energy of -8.0 kcal/mol to *Sh*HTL7. Its hydrogen bonds with SER-95 and MET-96 (bond lengths 3.1 Å and 2.8 Å, respectively) represent a distinctive polar interaction pattern for this receptor. Additionally, a hydrophobic pocket formed by aliphatic and sulfur-containing residues such as MET-219, LEU-124, and ILE-193, together with Pi-Alkyl interactions involving MET-96 and LEU-196, collectively enhances binding stability. KK094 engages in Pi-Pi T-shaped interactions with TYR-26 and π-cation interactions with HIS-246 and HIS-94, and also exhibits hydrogen bonding and hydrophobic complementarity, with a binding affinity of −7.8 kcal/mol. Overall, molecular docking predictions suggest that DK6 can form stable protein–ligand complexes with both *Os*D14 and *Sh*HTL7, providing a valuable reference for subsequent structure–activity relationship studies, though its actual binding affinity remains to be experimentally validated.

### Molecular dynamics simulations

**Fig. 8:**
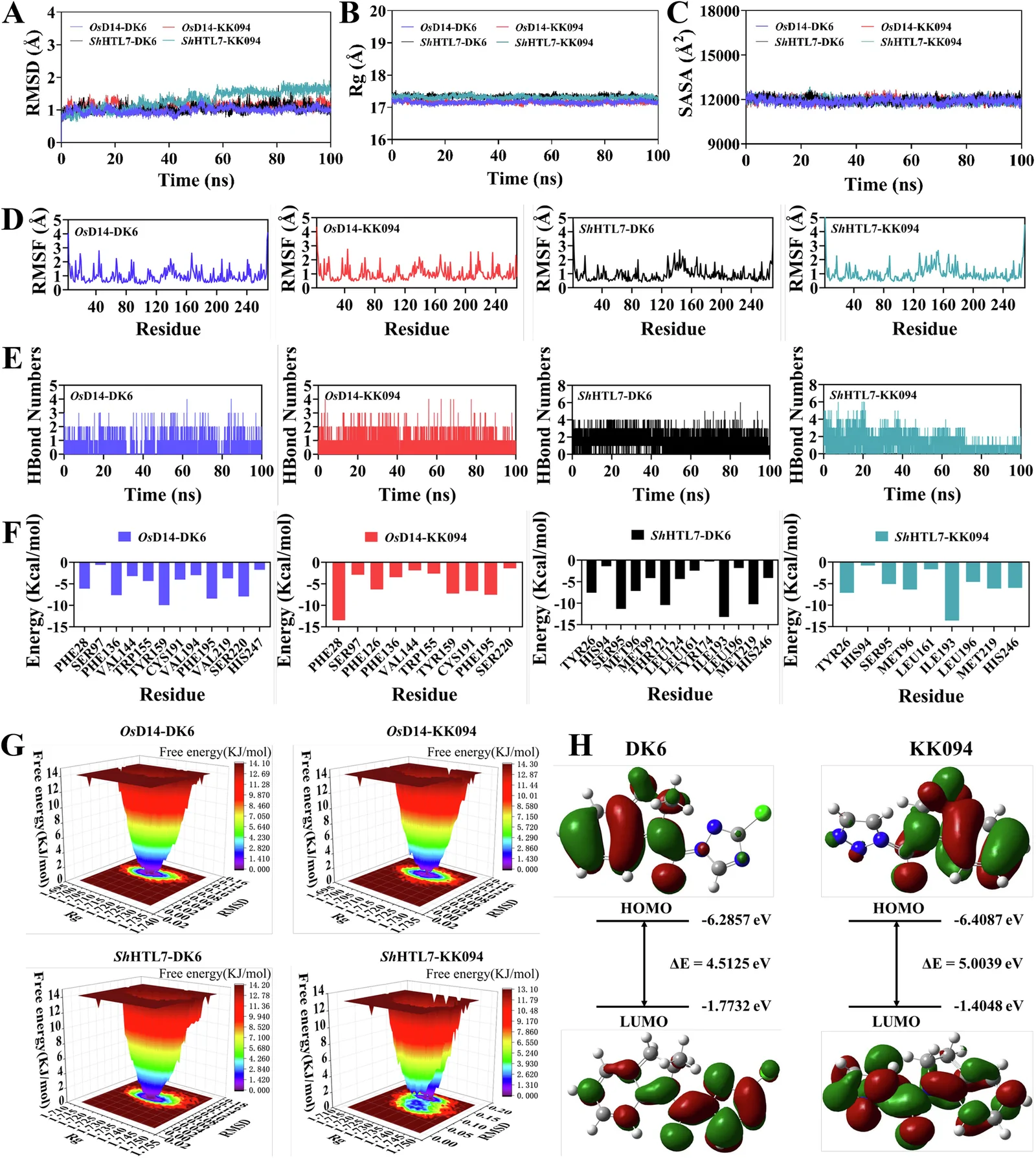
Molecular dynamics simulations and DFT calculations of DK6/KK094 (derived from CMCS‑DK6) in complex with the SL receptor proteins *Os*D14 and *Sh*HTL7. **A** root-mean-square deviation (RMSD), **B** radius of gyration (Rg), **C** solvent-accessible surface area (SASA), **D** root-mean-square fluctuation (RMSF) of the protein-ligand complexes over time. **E** Hydrogen bond numbers. **F** Binding free energy calculation per amino acid residue and **G** the free energy landscape (FEL). **H** DFT calculations for DK6/KK094. Source data are provided as a Source Data file.

Further molecular dynamics simulations were employed to predict the structural stability and binding affinity advantages of the DK6 complex within the *Os*D14 and *Sh*HTL7 protein systems (Fig. 8A–G). From the perspective of overall conformational stability, the root-mean-square deviation (RMSD) results from the simulations (Fig. 8A) revealed that the RMSD curves for the *Os*D14–DK6 and *Sh*HTL7–DK6 complexes rapidly converged and remained within a low, stable range throughout the 100 ns simulation time. In contrast, the RMSD values for *Os*D14–KK094 and *Sh*HTL7–KK094 not only converged more slowly but also exhibited significantly larger fluctuations. This indicates that the overall protein conformation underwent minimal drift after DK6 binding, providing a stable structural cornerstone for its high bioactivity. As shown in Fig. 8B, C, further analysis using the radius of gyration (Rg) and solvent-accessible surface area (SASA) demonstrated that the DK6 complexes possessed lower Rg values with minimal fluctuations, along with significantly reduced SASA values. Prediction results indicate a more compact structure for the DK6 complexes, where the hydrophobic pocket more effectively encapsulates and anchors the ligand molecule, thereby minimizing solvent interference with ligand–protein interactions and exhibiting the potential to enhance binding stability.

At the level of local dynamics, interaction strength, and thermodynamic stability, the root-mean-square fluctuation (RMSF) analysis of residues (Fig. 8D) revealed that key residues within the binding pocket (e.g., hydrophobic/polar residues involved in ligand recognition) in the DK6 complexes exhibited significantly lower fluctuations compared to the KK094 complexes. For instance, the RMSF values of binding pocket residues in *Os*D14–DK6 and *Sh*HTL7–DK6 were substantially lower than those in the corresponding KK094 systems, implying that DK6 enhances the rigidity of key residues, thereby facilitating sustained ligand–protein interactions. Hydrogen bond analysis (Fig. 8E) suggested that DK6 formed a moderately more stable hydrogen bond network with the proteins, showing a slight advantage over KK094. However, overall differences in hydrogen bonding between the two complexes were not pronounced. Binding free energy decomposition per residue (Fig. 8F) provided quantitative thermodynamic evidence, demonstrating more favorable (more negative) binding free energies for DK6 with key residues such as PHE and TYR in *Os*D14, indicating an energetically superior binding interaction. Free energy landscape (FEL) analysis (Fig. 8G) further indicated that the DK6 complexes occupied deeper and more concentrated low-energy wells, suggesting that their conformations are less prone to transition under physiological conditions and can sustainably maintain a high-activity binding state. In summary, prediction results indicate that the synergistic advantages across multiple dimensions—conformational stability, interaction strength, and thermodynamic stability—collectively contribute to the good binding affinity and potential bioactivity of DK6.

### Density functional theory

Based on density functional theory (DFT) calculations (Fig. 8H), the difference in biological activity between DK6 and KK094 primarily stems from their distinct frontier orbital characteristics. Compared to KK094, DK6 exhibits a higher highest occupied molecular orbital (HOMO) energy level (–6.2857 eV), with its electron density more localized over the aromatic region, indicating a stronger electron-donating capacity. Concurrently, DK6 possesses a lower lowest unoccupied molecular orbital (LUMO) energy level (–1.7732 eV), with its electron density more confined to the heteroatom-containing region, demonstrating superior electron-accepting ability. These properties render DK6 more active in electron transfer processes, thereby facilitating more effective interactions with biological targets such as proteins.

Furthermore, the HOMO–LUMO energy gap of DK6 (4.5125 eV) is smaller than that of KK094 (5.0039 eV). This narrower gap facilitates easier electron excitation, resulting in higher chemical reactivity, which in turn enhances the strength of interactions with biological targets. The aforementioned differences in electronic structure can be attributed to structural variations between the two compounds, including the degree of molecular conjugation and the nature of substituents. In summary, the combined advantages of DK6—namely, its narrower energy gap, favorable frontier orbital energy levels, and specific electron density distribution—endow it with enhanced chemical reactivity and a greater capacity for interacting with biomolecules, thereby accounting for its superior biological activity compared to KK094.

### Transcriptomics analysis

**Fig. 9:**
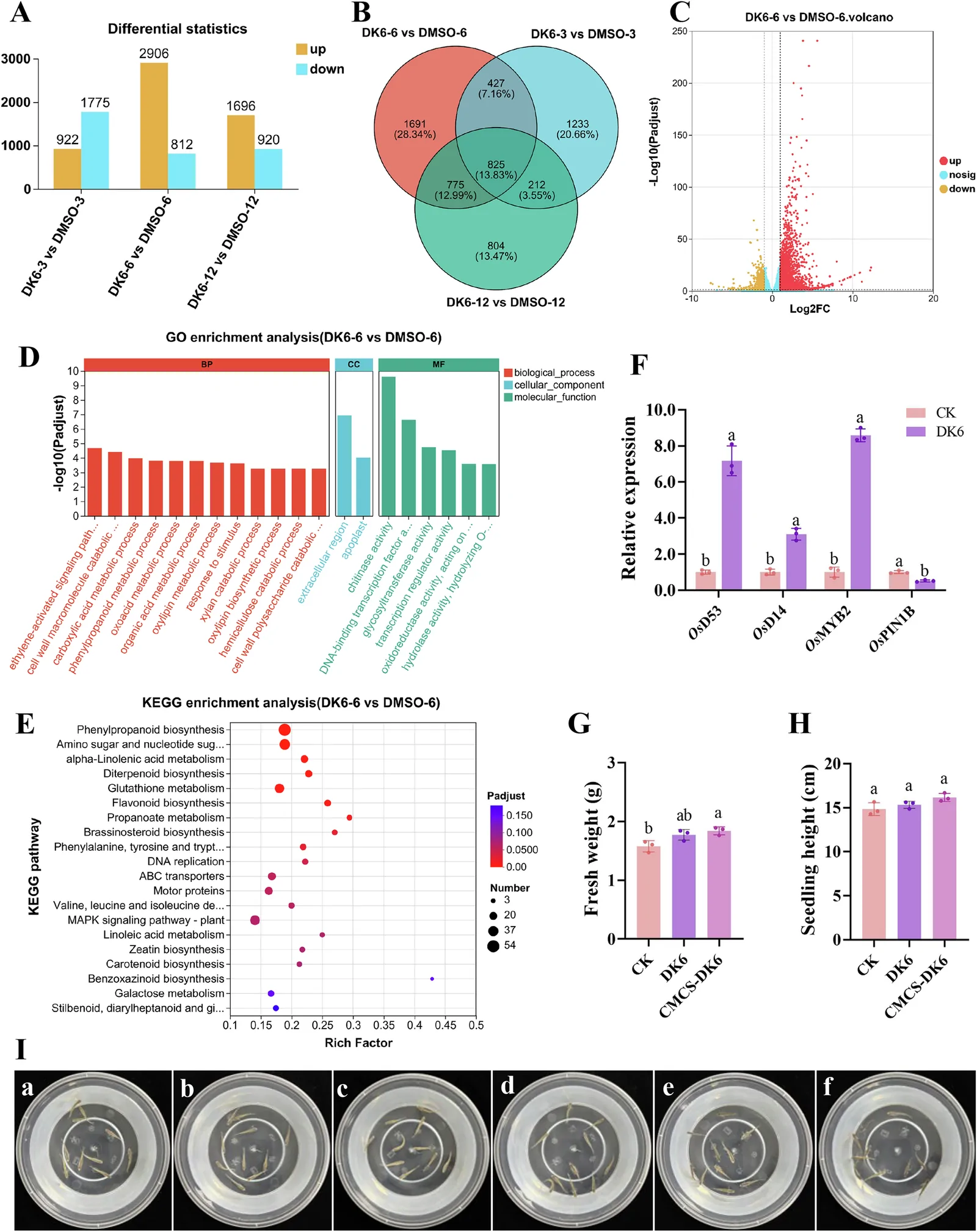
Transcriptomic analysis and key gene validation of DK6, and physiological effects and safety evaluation of the CMCS‑DK6 hydrogel. Transcriptomic analysis. **A** Statistics of differentially expressed genes (DEGs) and **B** Venn diagrams illustrating the differences between DK6 and DMSO control treatments at 3, 6, and 12 h. **C** Volcano plot of DEGs in the DK6 treatment group at 6 h. **D** GO and **E** KEGG enrichment analysis of the identified DEGs. **F** Gene expression analysis of relevant genes by RT-qPCR, with *Ubiquitin* as an internal control, the expression levels of *Os*D53 (*P* = 0.0002), *Os*D14 (*P* = 0.0006), *Os*PIN1B (*P* = 0.0001), and *Os*MYB2 (*P* = 0.0009) were all significantly altered in the DK6 treatment group. Nutritional function and safety of the CMCS-DK6 hydrogel. **G** Fresh weight and **H** seedling height of rice seeds on day 5 of germination after treatment with DK6 and CMCS-DK6 hydrogel. **I** Acute toxicity test of CMCS-Mn and CMCS-DK6 on zebrafish. Images of zebrafish captured after four days of exposure to aqueous solution (control, a), CMCS-Mn (b: 10 μg/mL, c: 15 μg/mL, d: 20 μg/mL), and CMCS-DK6 (e: 10 μg/mL, f: 15 μg/mL) in the acute toxicity assay. For panels **C**–**E**, Fisher’s exact test was used, and a corrected *P* < 0.05 was considered statistically significant. For panels **F**–**H**, error bars represent the mean ± standard deviation (SD). Statistical significance among means was determined using one-way ANOVA followed by Tukey’s multiple comparison test. Different letters indicate statistically significant differences (*P* < 0.05). The experiment was repeated three times, showing similar results (*n* = 3). Source data are provided as a Source Data file.

Differentially expressed genes (DEGs) were identified from rice samples treated with DK6, the active component of CMCS-DK6 hydrogel, versus DMSO controls, using thresholds of *p*-adjust <0.05 and |log_2_FC| ≥ 1. As summarized in Fig. 9A–C and Supplementary Data 1–3, a total of 2697 (922 up-regulated and 1775 down-regulated), 3718 (2906 up-regulated and 812 down-regulated), and 2616 (1696 up-regulated and 920 down-regulated) DEGs were detected at 3, 6, and 12 h after DK6 treatment, respectively. Venn diagram analysis further revealed 1233, 1691, and 804 unique DEGs across the three comparison sets (DK6-3 h vs DMSO-3 h, DK6-6 h vs DMSO-6 h, and DK6-12 h vs DMSO-12 h), along with 825 shared DEGs common to all time points. These results indicate that DK6 elicits dynamic transcriptomic changes in rice across multiple time points, with the most substantial alterations observed at 6 h post-treatment. This suggests that the 6-h time point may represent a critical window for DK6-mediated regulation of physiological processes such as tillering, warranting further investigation in subsequent analyses.

### GO enrichment and KEGG pathway analysis

As illustrated in Fig. 9D and Supplementary Data 4, functional annotation of DEGs in rice after 6-h treatment with compound DK6 revealed that Gene Ontology (GO) enrichment analysis showed, in the biological process (BP) category, DEGs were primarily enriched in various metabolic processes—such as cell wall macromolecule catabolic process, carboxylic acid metabolic process, and phenylpropanoid metabolic process—as well as response to stimulus, indicating that DK6 treatment significantly affected metabolic regulation and stress response mechanisms in the plants. In the cellular component (CC) category, genes were markedly enriched in the extracellular region and apoplast, suggesting substantial alterations in the structure or function of the cell periphery. Within the molecular function (MF) category, DEGs were predominantly associated with chitinase activity, DNA-binding transcription factor activity, glycosyltransferase/hydrolase activity, and transcription regulator activity, implicating roles in plant defense, regulation of gene expression, and macromolecular modification and degradation.

As shown in Fig. 9E and Supplementary Data 5, KEGG pathway analysis further indicated that DEGs were significantly enriched in pathways including phenylpropanoid biosynthesis, amino sugar and nucleotide sugar metabolism, and alpha-Linolenic acid metabolism. These pathways are largely related to secondary metabolite biosynthesis, carbohydrate metabolism, and hormone signal transduction, demonstrating that DK6 treatment extensively modulates secondary metabolism, energy metabolism, and signal transduction processes in rice at the transcriptional level. Together, these results suggest that DK6 may regulate multiple key metabolic and signaling pathways, thereby influencing rice tillering phenotype formation.

### Analysis of the SL pathway and genes related to rice tillering

Based on a multi-time-point transcriptome analysis, samples from the 6-h treatment group, which exhibited the most significant changes in differentially expressed genes, were selected for detailed investigation. At this time point, the expression of genes involved in SL biosynthesis and signaling, auxin efflux carriers, cytokinin synthase-encoding genes, as well as key regulatory factors closely associated with tillering and plant architecture establishment, were significantly altered. Further analysis revealed a specific gene regulatory network connecting SL signaling with other hormonal pathways, providing crucial molecular evidence for systematically elucidating the mechanism of action of DK6.

In the canonical SL signal transduction pathway, SL inhibits rice tillering by promoting the D14-D3 complex-mediated degradation of the D53 protein, thereby relieving the repression of downstream target genes^10,66^. Following treatment with the antagonist DK6, the SL signaling pathway is specifically inhibited, and the ubiquitination-mediated degradation of D53 is obstructed, leading to enhanced stability and accumulation of this protein within the cell. A strict negative feedback regulation mechanism exists within this signaling pathway. When DK6 antagonizes SL signaling, the plant misinterprets the internal SL level as insufficient, consequently lifting the transcriptional repression that SL normally imposes on its own biosynthesis and receptor expression. As an adaptive response, the plant modulates the expression of the receptor D14, key biosynthesis genes D10 and D17, and the signaling component gene D3, providing molecular-level confirmation of the pharmacological activity of DK6 as an inhibitor of the SL signaling pathway.

It is noteworthy that the transcription factor *Os*MYB2 has been found to interact with the *Os*ANT1 promoter and promote its expression^67^. Overexpression of *Os*MYB2 not only increases rice tiller number and yield, which is highly consistent with the upregulation of OsMYB2 observed after DK6 treatment. Concurrently, overexpression of *Os*JAC1 inhibits the elongation of coleoptile cells, resulting in a certain degree of plant height reduction^68^. Collectively, these findings elucidate the molecular basis underlying the multi-tillering and dwarf phenotype observed in rice following DK6 treatment.

Furthermore, our transcriptomic analysis suggests that DK6 may cooperatively regulate rice tillering with other hormone-related genes. Under normal conditions, SL signaling promotes the accumulation of the auxin efflux carrier PIN1 in vascular bundles, maintaining apical-dominant auxin export. In contrast, DK6 treatment reduces PIN1 expression and attenuates auxin-mediated suppression of lateral buds^65^. Additionally, given the known antagonistic interaction between SL and cytokinin in tillering regulation, DK6 treatment induces upregulation of IPT expression, potentially increasing cytokinin levels to directly stimulate tiller bud outgrowth^69^. Therefore, these expression changes are consistent with the expected effects of DK6 as an antagonist of the SL signaling pathway, providing transcriptome-level insights into its mechanism of action, although the detailed molecular mechanism requires further investigation

### RT-qPCR validation

To validate the reliability of the transcriptome sequencing results, we examined the expression levels of several key genes—including *Os*D53, *Os*D14, *Os*PIN1B, and *Os*MYB2—in rice treated with compound DK6 for 6 h using RT-qPCR. As shown in Fig. 9F, the expression trends of these genes were consistent with those observed in the transcriptome data, strongly supporting the credibility of the data. These results confirm that DK6 regulates key genes in the SL signaling pathway as well as genes involved in the hormone regulatory network.

### Nutritional function of hydrogel for rice growth

Based on the known function of manganese as a micronutrient in promoting seed germination, this study investigated the effect of CMCS-DK6 on rice growth (See Supplementary Information for details). As shown in Supplementary Fig. 8, with the gradual release of DK6 and Mn^2+^ from the hydrogel into the soil, rice seeds in the CMCS-DK6 treatment group germinated slightly earlier than those in the untreated group. The day of seed germination in each treatment group was recorded as Day 1. Compared with the control group (CK), the CMCS-DK6 treatment group exhibited increased seedling height (*P* = 0.0577) and fresh weight (*P* = 0.0213) on Day 5 (Fig. 9G, H). These results collectively suggest that the Mn^2+^-based CMCS-DK6 hydrogel may have potential as a crop nutrient supplement.

### Zebrafish acute toxicity analysis

To evaluate the environmental safety of the materials towards non-target organisms, acute toxicity tests were conducted using zebrafish. Zebrafish were exposed to CMCS-Mn and CMCS-DK6 solutions, and their status was observed after 96 h, with results presented in Fig. 9I. CMCS-Mn did not cause obvious acute toxicity at concentrations of 20, 15, and 10 μg/mL. For CMCS-DK6, the survival rate of zebrafish was 86.7% at 15 μg/mL, and no significant acute toxicity was observed at 10 μg/mL. According to the standard toxicity classification (LC_50_ > 10 μg/mL indicates low toxicity), the CMCS-Mn and CMCS-DK6 hydrogels exhibited limited toxicity and only mild effects upon acute exposure in zebrafish. However, if a comprehensive assessment of their environmental safety is required, further and more systematic ecotoxicological studies are still needed in the future.

In summary, this study successfully designed and synthesized a series of SL inhibitors, DK1-DK32. Among them, DK6 demonstrated optimal comprehensive efficacy in inhibiting *S. asiatica* seed germination and promoting rice tillering in bioactivity screenings. To further enhance its application potential, we developed a manganese ion-cross-linked CMCS-based pH-responsive hydrogel drug delivery system, which achieved pH-responsive controlled release of DK6 within the root zone. This system significantly improved the compound’s stability, duration of efficacy, and biocompatibility. The CMCS-DK6 hydrogel displayed higher release efficiency under alkaline conditions, making it suitable for the on-demand delivery of agrochemicals. Molecular docking and molecular dynamics simulations suggest that DK6 forms stable complexes with both *Os*D14 and *Sh*HTL7, providing a basis for future structure–activity relationship studies. Meanwhile, MST results showed that the experimental binding affinity of DK6 to the SL receptor *At*D14 is also higher than that of KK094. However, it should be noted that direct binding of DK6 to *Os*D14 and *Sh*HTL7 receptors requires further confirmation. Transcriptomics further confirmed at a systems level that DK6 specifically inhibits SL signal transduction, disrupts D53 protein degradation, and activates multiple pathways—including phenylpropanoid metabolism, carbohydrate metabolism, and multi-hormone networks (e.g., auxin and cytokinin)—collectively regulating rice tiller development and plant architecture. Taken together, we propose a polysaccharide-hydrogel-based strategy for SL inhibitor delivery. This system exhibits synergistic advantages in promoting crop tillering, reducing plant height, and controlling root parasitic weeds, thereby not only furnishing a chemical lead and delivery vehicle for the chemical intervention of SL signaling but also providing a feasible materials-based solution and theoretical foundation for advancing green agriculture.

## Methods

### Materials

The materials used in this study were obtained from the following sources: *Striga asiatica* was provided by the Institute of Genetics and Developmental Biology, Chinese Academy of Sciences (Beijing, China). *Phelipanche aegyptiaca* and *Orobanche cumana* were kindly gifted by Shihezi University (Xinjiang, China). The SL signaling mutant *max2-1* was supplied by the College of Biological Sciences, China Agricultural University (Beijing, China).

### Instruments and chemicals

Reagents and solvents were purchased from Shanghai Aladdin Biochemical Technology Co., Ltd. and Haohong Biological Co., Ltd., and used without further purification. Nuclear magnetic resonance (NMR) spectra (^1^H, ^13^C, and ^19^F NMR) were recorded on a Bruker AVANCE DPX500 spectrometer (Bruker Corp., Fallanden, Switzerland) using CDCl₃ as the solvent. Chemical shifts (δ) are reported in ppm, coupling constants (*J*) in Hz, and peak multiplicities are indicated as *s* = singlet, *d* = doublet, *t* = triplet, and *m* = multiplet. Melting points were determined using an X-4A micro melting point apparatus (Shanghai Precision Instrument Co., Ltd., China). High-resolution mass spectrometry (HRMS) was performed on a Thermo Fisher Q Exactive mass spectrometer.

### Synthesis of target compounds DK1-DK32

In a dried reaction vessel, a solution of triphosgene (3.12 mmol) and pyridine (6.79 mmol) in anhydrous dichloromethane was prepared. A solution of substituted dihydroindole (6.79 mmol) was added dropwise under an ice bath, and the mixture was stirred at low temperature for 30 min before being warmed to room temperature. After completion of the reaction, the mixture was washed with 0.1 M hydrochloric acid and extracted with dichloromethane. The crude product was purified by column chromatography (petroleum ether/ethyl acetate = 10:1) to afford intermediates 1a-1b. Intermediates 1a-1b (1.10 mmol) were slowly added to a solution of substituted five-membered heterocycle (1.16 mmol) and triethylamine (2.20 mmol) in anhydrous acetonitrile. Upon reaction completion, the crude product was purified by column chromatography (petroleum ether/ethyl acetate = 5:1) to yield target compounds DK1-DK32.

### Preparation of CMCS-DK6 hydrogel

The synthesis of CMCS-DK6 hydrogel microspheres was conducted under optimized conditions utilizing 4 wt% CMCS, an oil-to-water phase volume ratio of 1:10, 2 wt% Tween-80, and a 0.2 M Mn^2+^ solution. Specifically, 1.6 g of CMCS was dispersed in 40 mL of water to form the aqueous phase. The oil phase was prepared by dissolving 0.8 g of DK6 in 4 mL of dichloromethane containing 0.4 mL of Tween-80. Under vigorous stirring at 10,000 rpm, the oil phase was added dropwise into the aqueous phase, followed by emulsification for 5–10 min to form a stable emulsion. Subsequently, 10 mL of the resulting emulsion was transferred via a syringe and added dropwise into 30 mL of a 0.2 M metal ion solution (Zn^2+^, Cu^2+^, Mn^2+^, and Fe^2+^/Fe^3+^). The mixture was stirred at room temperature for 4–5 h to facilitate microsphere cross-linking and solidification. The obtained hydrogel particles were collected, washed thoroughly with deionized water, and dried in an oven at 55 °C for approximately 12 h, yielding the drug-loaded CMCS-DK6 hydrogel microspheres. Blank hydrogel microspheres without DK6 were prepared following an identical procedure.

### Characterization of CMCS-DK6 hydrogel

The morphology of the hydrogel, following sputter-coating with a thin gold layer, was examined using a scanning electron microscope (SEM, ZEISS GeminiSEM 300, Germany) operated at an accelerating voltage of 3 kV. Its elemental composition was analyzed using the attached energy-dispersive X-ray spectroscopy (EDS) system. Fourier transform infrared (FT-IR) spectra of the samples were acquired on a spectrometer (Thermo Fisher Scientific Nicolet iS20, USA) within the wavenumber range of 400–4000 cm⁻^1^. The crystallinity of the hydrogel was evaluated by XRD (Rigaku SmartLab SE, Japan) with a scanning range of 5–90° (2θ) at a rate of 2°/min. The chemical composition of the samples was analyzed *via* X-ray photoelectron spectroscopy (XPS), performed on a photoelectron spectrometer (Thermo Scientific K-Alpha, USA).

### Loading content and encapsulation efficiency

Accurately weighed 10 mg of CMCS-DK6 hydrogel microspheres were placed into a 10 mL volumetric flask, dissolved in dichloromethane, and diluted to volume. The solution was sonicated for 30 min and subsequently filtered through a 0.45 μm membrane. The concentration of DK6 in the resulting sample was quantified using high-performance liquid chromatography (HPLC, Agilent 1200 system). The chromatographic separation was performed on an Agilent Eclipse Plus C18 column (5 μm, 4.6 × 150 mm) maintained at 25 °C. The mobile phase, consisting of methanol and 0.2% aqueous formic acid solution (80:20, v/v), was delivered at a flow rate of 1.0 mL/min. Detection was carried out at a wavelength of 289 nm, with an injection volume of 5 μL^45^. The drug loading content (LC) and encapsulation efficiency (EE) were calculated according to Eqs. (1, 2), respectively:

1 $${LC}\,(\%)=\frac{{weight}\,{of}\,{DK}6\,{entrapped}\,{in}\,{hydrogel}\,{granules}}{{weight}\,{of}\,{hydrogel}\,{granules}}\times 100$$

2 $${EE}\,\left(\%\right)=\frac{{weight}\,{of}\,{DK}6\,{entrapped}\,{in}\,{hydrogel}\,{granules}}{{intitial}\,{weight}\,{of}\,{DK}6\,{employed}}\times 100$$

### Swelling studies

Hydrogels are polymeric materials characterized by a three-dimensional cross-linked network structure, which enables them to absorb substantial amounts of water and undergo significant swelling. To evaluate the water absorption and swelling behavior of the CMCS-DK6 hydrogel under specific conditions, a predetermined amount of the sample was placed in a culture dish and immersed in 15 mL of PBS solution at varying pH levels (4.6, 5.5, 6.6, 7.5, 8.5, and 9.5). At designated time intervals, the hydrogel was carefully removed, and any surface moisture was gently blotted with filter paper before weighing. The hydrogel was then returned to the original solution. The swelling ratio (*SR*) was calculated according to Eq. (3):

3 $${SR}\,\left(\%\right)=\frac{{W}_{s}-{W}_{d}}{{W}_{d}}\times 100$$

where *W*_*s*_ represents the mass of the swollen hydrogel and *W*_*d*_ denotes the mass of the dry hydrogel.

### In vitro release behavior

The drug release study was conducted using dried CMCS-DK6 hydrogel particles incubated in 30 mL of PBS release medium at different pH values (5.5, 6.6, 7.5, and 8.5). The experiment was performed in a constant-temperature shaker at 25 °C with agitation at 200 rpm. At predetermined time intervals, 1 mL of the release medium was withdrawn and replaced with an equal volume of fresh PBS at the same pH to maintain a constant total volume. The concentration of DK6 in the collected samples was determined by HPLC, and the cumulative release percentage, *R* (%), was calculated using Eq. (4):

4 $$R\left(\%\right)=\frac{{C}_{t}{V}_{0}+V{\sum }_{0}^{t-1}{C}_{t}}{{{{\rm{M}}}}}\times 100$$

where *R* represents the cumulative release of DK6; *V*_*0*_ and *V* denote the total volume of the release medium and the sample volume (1 mL), respectively; *C*_*t*_ is the concentration of DK6 at time *t*; and M is the total loading amount of DK6 in the hydrogel.

The release profiles were fitted using four kinetic models: the zero-order model, the first-order model, the Higuchi model, and the Ritger–Peppas model^70^, as described by Eq. (5–8), respectively:

5 $${{{\rm{Zero}}}}-{{{\rm{order\; model}}}}:\,{M}_{t}/{M}_{z}={kt}$$

6 $${{{\rm{First}}}}-{{{\rm{order\; model}}}}:\,{M}_{t}/{M}_{z}=1-{e}^{-{kt}}$$

7 $${{{\rm{Higuchi\; model}}}}:\,{M}_{t}/{M}_{z}={kt}^{1/2}$$

8 $${{{\rm{Ritger}}}}-{{{\rm{Peppas\; model}}}}:\,{M}_{t}/{M}_{z}={kt}^{n}$$

Here, *M*_*t*_*/Mz* is the cumulative percentage of DK6 released from the CMCS-DK6 hydrogel particles at time *t*; k is the release rate constant; and *n* is the diffusion exponent.

### Seed germination assay

Seeds of *Striga asiatica* (*S. asiatica*), *Phelipanche aegyptiaca* (*P. aegyptiaca*), and *Orobanche cumana* (*O. cumana*) were surface-sterilized with 1% NaClO solution for 2 min, followed by treatment with 75% ethanol for 2 min^15^. After rinsing 5–6 times with sterile water, the seeds were air-dried. The seeds were evenly distributed on glass fiber filter paper (Grade GF/D, Whatman, Cytiva, USA), with 40–80 seeds per plate, and placed in Petri dishes containing moistened filter paper. They were then incubated in the dark for 7 days: *S. asiatica* at 30 °C, and *P. aegyptiaca* and *O. cumana* at 25 °C. Subsequently, 80 μL of a mixed solution containing the test compounds at various concentrations (80, 40, 20, 10, and 5 μM, with DMSO concentration maintained below 0.1%, and for some compounds the range is 0.3125-80 μM) and 100 nM GR24 was added to each Petri dish. The dishes were then sealed and further incubated in darkness for 7 days. Germinated seeds were observed and counted under a stereomicroscope. The germination rate (*G*) and half-maximal inhibitory concentration (IC₅₀) were calculated using Equation (10):

9 $$G\,(\%)=({N}_{1}/{N}) \times \,100$$

where *G* represents the seed germination rate, *N*_*1*_ is the number of germinated seeds, and *N* is the total number of treated seeds.

### Rice tillering bioassay

Rice seeds (*Oryza sativa* L.) were surface-sterilized with 2% NaClO solution for 20 min, rinsed six times with sterile water, and then placed in Petri dishes containing moistened filter paper. The dishes were incubated in a growth chamber at 30 °C under a 16 h light/8 h dark photoperiod to promote germination. After germination, the seedlings were transferred to a hydroponic setup containing distilled water and cultivated for one week. Uniform seedlings were then selected and transferred to nutrient solutions supplemented with either the test compounds at various concentrations or an equivalent volume of DMSO (control). The plants were cultivated for an additional three weeks, with the nutrient solution being replaced every 3–4 days. After four weeks of total cultivation, the number of tillers with a length of at least 1 cm was recorded.

### Effects on Rice Seed Germination

Surface-sterilized rice seeds were distributed into Petri dishes and treated with solutions containing either 10 μM CMCS-DK6, 10 μM DK6, or no compound (control), with three replicates per treatment. The seeds were incubated in a growth chamber set at 30 °C with a 16/8-h light/dark cycle for 5 days, during which the solutions were replaced daily. For each replicate, 20–25 seeds were randomly selected to assess germination rates, and their root and shoot lengths were measured.

### A. thaliana hypocotyl elongation assay

Seeds of *A. thaliana* (*Col-0, max2-1*) were surface-sterilized in 1% NaClO solution for 15 min, rinsed 5–6 times with sterile water, and stratified at 4 °C in the dark for 3 days. The seeds were then evenly sown onto half-strength Murashige and Skoog (MS) solid medium containing 1% sucrose and 0.8% agar, with or without the test compounds. The Petri dishes were placed in a growth chamber and maintained at 22 °C under a 16 h light/8 h dark cycle for 7 days. After cultivation, hypocotyl lengths of the *A. thaliana* seedlings were measured and analyzed using ImageJ software. (https://imagej.nih.gov/ij/index.html)

### Microscale thermophoresis

The *At*D14 gene was amplified from *A. thaliana*, cloned into the pET28a vector, and transformed into E. coli DE3 pLysS. Following induction with IPTG, the recombinant *At*D14 protein was purified by Ni‑NTA affinity chromatography and verified by SDS‑PAGE (see Supplementary Information for details). Binding affinities of compounds DK6/KK094 to *At*D14 were determined using microscale thermophoresis (MST) on a Monolith NT.115 instrument (NanoTemper Technologies, Munich, Germany). *At*D14 protein was fluorescently labeled with the Monolith NT Protein Labeling Kit RED‑NHS (2nd Generation, NanoTemper). Test compounds were serially diluted and mixed with an equal volume of labeled *At*D14 at 25 °C in the dark for 5 min. Samples were loaded into standard treated capillaries and measured on the Monolith NT.115 according to the preset program, with three replicates. Data were analysed by nonlinear fitting using MO. Affinity Analysis software to obtain the dissociation rate constant (K_d_).

### Molecular docking

Molecular docking was employed to investigate the interactions between the ligands and the target protein and to predict their binding modes. The crystal structure of the SL receptor protein (PDB ID: 5DJ5^71^ or 7SNU^72^) was retrieved from the Protein Data Bank. Water molecules and co-crystallized ligands were removed, and hydrogen atoms were added using PyMOL. The small molecule ligands, DK6 and KK094, were subjected to energy minimization for optimization. A grid box was defined, and charges were assigned. Semi-flexible docking calculations were performed using AutoDock Vina to screen for the optimal binding conformation, followed by visualization and analysis.

### Molecular dynamics simulation

Molecular dynamics (MD) simulations were performed using GROMACS 2022^73^. The Amber14SB force field and the General Amber Force Field (GAFF2) were applied to the receptor protein and ligands, respectively. The system was solvated in a TIP3P water box, neutralized, and subjected to energy minimization. A 100 ns MD simulation was conducted under NPT ensemble conditions at 310 K with an integration time step of 1 fs. Long-range electrostatic interactions were treated using the Particle Mesh Ewald (PME) method. Using the backbone atoms of the full-length protein as the analysis target and the energy-minimized initial structure as the reference conformation, we calculated the root-mean-square deviation (RMSD), root-mean-square fluctuation (RMSF), hydrogen bonds, radius of gyration (Rg), and solvent-accessible surface area (SASA) from the simulation trajectory using GROMACS.

### Density functional theory

All density functional theory (DFT) calculations were performed using Gaussian 16^74^. Geometry optimizations and frequency analyses were carried out at the B3LYP level to verify that the obtained structures represent local minima on the potential energy surface without imaginary frequencies. Single-point energy calculations were then conducted based on the optimized structures, with solvation effects incorporated. The chemical reactivity was subsequently evaluated by analyzing frontier molecular orbital energy gaps and electrostatic potential distributions.

### Transcriptomic analysis

For transcriptomic analysis, tiller bases from rice plants treated with compound DK6 were collected as experimental materials. The samples were immediately frozen and stored at −80 °C until further processing. Library preparation and paired-end sequencing were performed on an Illumina platform by Shanghai Majorbio Bio-pharm Technology Co., Ltd. Differential expression analysis was conducted using the DESeq2 software. Genes meeting the criteria of |log_2_(Fold Change)| ≥ 1 and False Discovery Rate (FDR) < 0.05 were identified as differentially expressed genes (DEGs)^75^. To systematically elucidate the biological processes and metabolic pathways affected by DK6 treatment in rice, Gene Ontology (GO) functional annotation, encompassing the categories of Biological Process (BP), Cellular Component (CC), and Molecular Function (MF), as well as Kyoto Encyclopedia of Genes and Genomes (KEGG) pathway enrichment analysis, was performed on the identified DEGs.

### RT-qPCR analysis

Total RNA was extracted from rice tiller node tissues using the *SteadyPure* Plant RNA Rapid Extraction Kit (Accurate Biotechnology Co., Ltd, China), followed by reverse transcription and SYBR Green premix-based quantitative detection. RT-qPCR was performed on a Step One Plus system (Applied Biosystems) with *Ubiquitin* as the reference gene. Relative gene expression data were analyzed using the 2^⁻ΔΔCt^ method. Primer sequences are provided in Supplementary Table 3.

### Zebrafish acute toxicity assay

Acute toxicity was assessed using a static water bioassay with zebrafish under non-feeding conditions. Zebrafish (*n* = 10 per group) were exposed to aqueous solutions containing CMCS-DK6 (15 and 10 μg/mL) or CMCS-Mn (20μg/mL, 15 μg/mL and 10 μg/mL) for 96 h, after which survival was recorded. Deionized water was used as a control. Each treatment was performed in triplicate.

### Statistical analysis

Statistical analyses were performed using Origin 2022, GraphPad Prism 9.50, and Microsoft Excel 2021. Unless otherwise stated, all experiments were conducted with at least three biological replicates, and data are presented as mean ± standard deviation (SD). Statistical significance of differences between means was assessed using one‑way ANOVA followed by Tukey’s multiple comparison test *via* DPS software, with different letters indicating statistically significant differences (*P* < 0.05). Source data are provided as a Source Data file.

### Reporting summary

Further information on research design is available in the Nature Portfolio Reporting Summary linked to this article.

## Supplementary information

Supplementary Information

Description of Additional Supplementary Files

Supplementary Data 1

Supplementary Data 2

Supplementary Data 3

Supplementary Data 4

Supplementary Data 5

Reporting Summary

Transparent Peer Review file

## Source data

Source Data

## Data Availability

The authors declare that the data supporting the findings of this study are available within the paper and its supplementary information files. The raw transcriptomic data generated in this study have been deposited in the NCBI Sequence Read Archive (SRA) under accession code PRJNA1457576. The crystal structures of the SL receptor protein used for molecular docking in this study are available in the Protein Data Bank at https://www.rcsb.org/structure/5DJ5 (PDB code: 5DJ5^71^) and https://www.rcsb.org/structure/7SNU (PDB code: 7SNU^72^). Source data are provided with this paper.
