# Supplementary material for: pH-responsive manganese carboxymethyl chitosan hydrogel releases pesticide to promote rice tillering and suppress root-parasitic weeds
Source: Nat Commun. 2026 Oct 9;17:11467. doi: 10.1038/s41467-026-78320-3 (PMC13739920; doi:10.1038/s41467-026-78320-3)
Supplement: Supplementary file 1 [file 41467_2026_78320_MOESM1_ESM.pdf]

Supplementary

pH-Responsive Manganese Carboxymethyl Chitosan Hydrogel Releases Pesticide to Promote Rice Tillering and Suppress Root-Parasitic Weeds

Ali Dai[1], Zhiguo Zheng[2], Ronghao Wu[1], Dengke Ma[1], Junyan Xu[1], You Mo[1], Liusheng Duan[1], Weiming Tan[1]*

Affiliations:

[1]*Engineering Research Centre of Plant Growth Regulators, Ministry of Education, College of Agronomy and Biotechnology, China Agricultural University, No. 2 Yuanmingyuan West Road, Haidian District, Beijing 100193, China;*

[2]*State Key Laboratory of Green Pesticide, Center for R&D of Fine Chemicals of Guizhou University, Guiyang 550025, China.*

***CORRESPONDING AUTHOR FOOTNOTE**

*Authors to whom correspondence should be addressed;

E-mails: tanwm@cau.edu.cn.

Table of Contents

1. ^{1}H NMR, ^{13}C NMR, ^{19}F NMR and HRMS data for DK1-DK32 3

2. ^{1}H NMR, ^{13}C NMR, ^{19}F NMR and HRMS spectra 17

3. Effects of DK1-DK32 on rice plant height and dry weight 81

4. Rice tillering-promoting activity of DK6 at different concentrations 83

5. Digital photos of CMCS-DK6 swelling 84

6. pH-dependent swelling reasonable mechanism of CMCS-DK6 hydrogel 85

7. Cumulative release and kinetics of CMCS-DK6 hydrogel in different soils 85

8. Determination of rice growth in different soils 86

9. Rice growth in different soils 86

10. Kinetic fitting of cumulative release of CMCS-DK6 in different pH and soil 87

11. Construction and purification of *At*D14 protein expression vector 88

12. Hydrogel nutrient function 89

13. Photographs of rice with different treatment 89

0

1. ^{1}H NMR, ^{13}C NMR, ^{19}F NMR and HRMS data for DK1-DK32

(3-chloro-1*H*-1,2,4-triazol-1-yl)(indolin-1-yl)methanone (DK1)

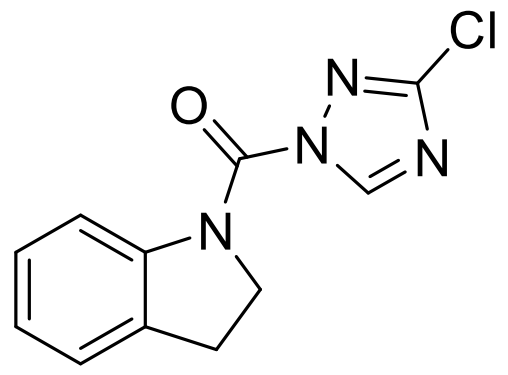

Yield 88%; White solid; m.p.141.3 – 142.5 °C.

^{1}H NMR (500 MHz, $CDCl_3$) δ 8.88 (s, 1H), 8.03 (d, J = 8.0 Hz, 1H), 7.29 – 7.25 (m, 2H), 7.14 (m, 1H), 4.58 (t, J = 8.3 Hz, 2H), 3.23 (t, J = 8.3 Hz, 2H).

^{13}C NMR (126 MHz, $CDCl_3$) δ 153.9, 147.5, 145.3, 141.8, 132.1, 127.8, 125.6, 125.1, 117.7, 51.4, 28.7.

HRMS (ESI) m/z Calcd for $C_{11}H_9ClN_4ONa$ $[M+Na]^+$ 271.03571; Found 271.03587.

(3,5-dibromo-1*H*-1,2,4-triazol-1-yl)(indolin-1-yl)methanone (DK2)

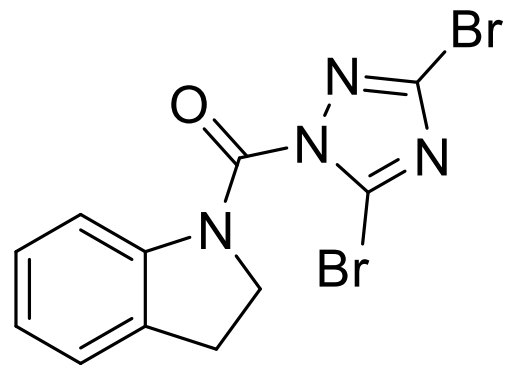

Yield 60%; White solid; m.p.113.5 – 115.0 °C.

^{1}H NMR (500 MHz, $CDCl_3$) δ 7.94 (dd, J = 84.2, 8.1 Hz, 1H), 7.31 – 7.20 (m, 2H), 7.12 (m, 1H), 4.25 – 4.21 (m, 2H), 3.22 (t, J = 8.4 Hz, 2H).

^{13}C NMR (126 MHz, $CDCl_3$) δ 144.5, 141.0, 132.4, 130.5, 128.0, 126.0, 125.2, 117.4, 116.4, 50.8, 28.2.

HRMS (ESI) m/z Calcd for $C_{11}H_8Br_2N_4ONa$ $[M+Na]^+$ 392.89570; Found 392.89541.

(3-bromo-1*H*-1,2,4-triazol-1-yl)(indolin-1-yl)methanone (DK3)

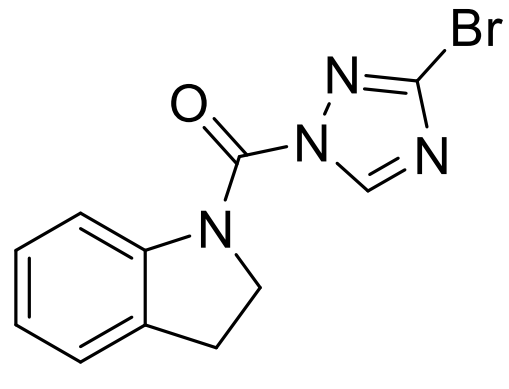

Yield 91%; White solid; m.p.141.9 – 143.3°C.

^{1}H NMR (500 MHz, $CDCl_3$) δ 8.85 (s, 1H), 8.03 (d, *J* = 8.0 Hz, 1H), 7.29 – 7.25 (m, 2H), 7.14 (m, 1H), 4.58 (t, *J* = 8.4 Hz, 2H), 3.23 (t, *J* = 8.3 Hz, 2H).

^{13}C NMR (126 MHz, $CDCl_3$) δ 147.7, 145.2, 141.8, 141.7, 132.2, 127.8, 125.6, 125.1, 117.7, 51.4, 28.7.

HRMS (ESI) m/z Calcd for $C_{11}H_{9}BrN_{4}ONa$ $[M+Na]^{+}$ 314.98519; Found 314.98521.

indolin-1-yl(3-methyl-1*H*-1,2,4-triazol-1-yl)methanone (DK4)

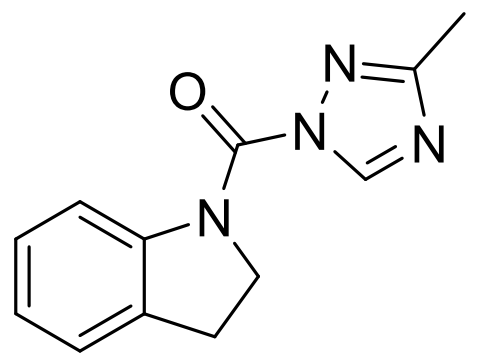

Yield 44%; White solid; m.p.90.4 – 91.5 °C.

^{1}H NMR (500 MHz, $CDCl_3$) δ 8.84 (s, 1H), 7.28 – 7.23 (m, 2H), 7.21 – 7.07 (m, 2H), 4.58 (t, *J* = 8.3 Hz, 2H), 3.21 (t, *J* = 8.3 Hz, 2H), 2.45 (s, 3H).

^{13}C NMR (126 MHz, $CDCl_3$) δ 162.1, 146.8, 142.2, 132.1, 127.7, 125.2, 125.0, 117.6, 115.2, 51.3, 28.7, 14.3.

HRMS (ESI) m/z Calcd for $C_{12}H_{12}N_{4}ONa$ $[M+Na]^{+}$ 251.09033; Found 251.08938.

(3,5-dimethyl-1*H*-1,2,4-triazol-1-yl)(indolin-1-yl)methanone (DK5)

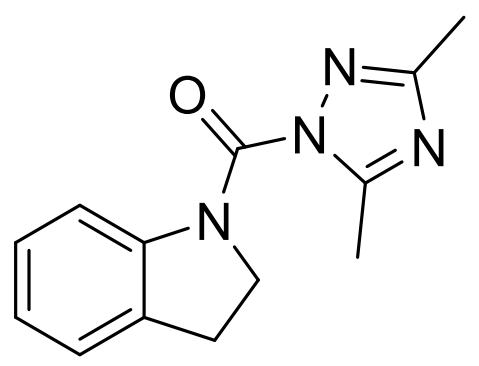

Yield 60%; Purple solid; m.p.112.3 – 113.5 °C.

^{1}H NMR (500 MHz, $CDCl_3$) δ 7.24 – 7.18 (m, 2H), 7.09 (m, 2H), 4.35 (t, *J* = 8.3 Hz, 2H), 3.17 (t, *J* = 8.3 Hz, 2H), 2.66 (s, 3H), 2.37 (s, 3H).

^{13}C NMR (126 MHz, $CDCl_3$) δ 156.1, 148.1, 143.2, 132.3, 127.7, 127.3, 125.1, 122.4, 115.2, 50.1, 28.4, 14.3, 14.0.

HRMS (ESI) m/z Calcd for $C_{13}H_{15}N_{4}O$ $[M+H]^{+}$ 243.12403; Found 243.12405.

(3-chloro-1*H*-1,2,4-triazol-1-yl)(2-methylindolin-1-yl)methanone (DK6)

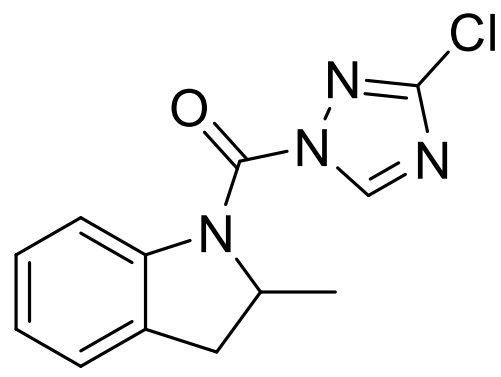

Yield 78%; White solid; m.p.75.9 – 77.6 °C.

^{1}H NMR (500 MHz, $CDCl_3$) δ 8.89 (s, 1H), 8.02 (s, 1H), 7.28 (m, 2H), 7.15 (m, 1H), 5.74 – 5.56 (m, 1H), 3.48 (dd, *J* = 15.6, 8.7 Hz, 1H), 2.82 – 2.70 (m, 1H), 1.28 (d, *J* = 6.4 Hz, 3H).

^{13}C NMR (126 MHz, $CDCl_3$) δ 154.1, 147.7, 145.2, 140.8, 131.3, 127.8, 125.7, 125.5, 118.3, 58.1, 36.6, 21.8.

HRMS (ESI) m/z Calcd for $C_{12}H_{11}ClN_4ONa$ $[M+Na]^+$ 285.05136; Found 285.05154.

(3-bromo-1*H*-1,2,4-triazol-1-yl)(2-methylindolin-1-yl)methanone (DK7)

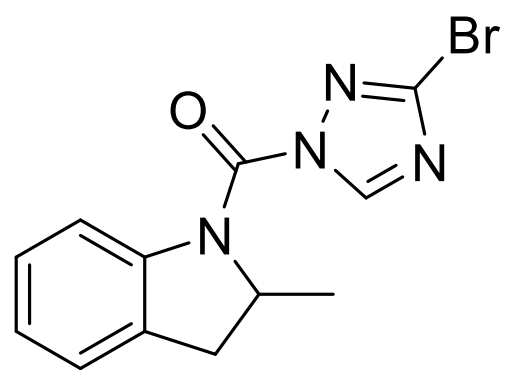

Yield 87%; White solid; m.p.96.6 – 98.2 °C.

^{1}H NMR (500 MHz, $CDCl_3$) δ 8.86 (s, 1H), 8.02 (s, 1H), 7.27 (d, *J* = 7.8 Hz, 2H), 7.15 (m, 1H), 5.66 (m, 1H), 3.48 (dd, *J* = 15.8, 8.8 Hz, 1H), 2.76 (dd, *J* = 15.7, 1.5 Hz, 1H), 1.27 (d, *J* = 6.4 Hz, 3H).

^{13}C NMR (126 MHz, $CDCl_3$) δ 147.9, 145.1, 142.0, 140.8, 131.3, 127.8, 125.7, 125.5, 118.3, 58.1, 36.6, 21.8.

HRMS (ESI) m/z Calcd for $C_{12}H_{11}BrN_4ONa$ $[M+Na]^+$ 329.00084; Found 329.00000.

(3-methyl-1*H*-1,2,4-triazol-1-yl)(2-methylindolin-1-yl)methanone (DK8)

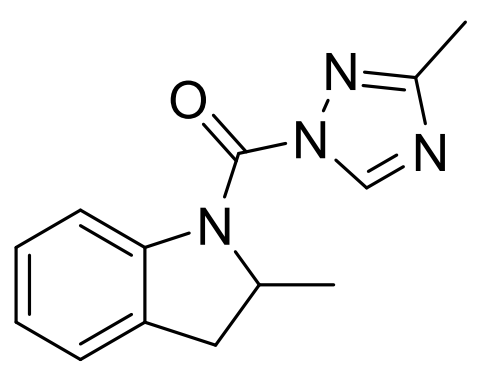

Yield 70%; Light yellow oily.

^{1}H NMR (500 MHz, $CDCl_3$) δ 8.84 (s, 1H), 7.99 (s, 1H), 7.25 (d, *J* = 7.1 Hz, 2H), 7.12 (m, *J* = 7.3 Hz, 1H), 5.72 (t, *J* = 7.4 Hz, 1H), 3.47 (dd, *J* = 15.7, 8.9 Hz, 1H), 2.77 – 2.68 (m, 2H), 2.46 (s, 3H), 1.24 (d, *J* = 6.6 Hz, 3H).

^{13}C NMR (126 MHz, $CDCl_3$) δ 162.3, 147.0, 146.5, 141.2, 131.2, 127.7, 125.4, 125.3, 118.1, 57.8, 36.5, 21.7, 14.3.

HRMS (ESI) m/z Calcd for $C_{13}H_{14}N_4ONa$ $[M+Na]^+$ 265.10598; Found 265.10593.

(3,5-dimethyl-1*H*-1,2,4-triazol-1-yl)(2-methylindolin-1-yl)methanone (DK9)

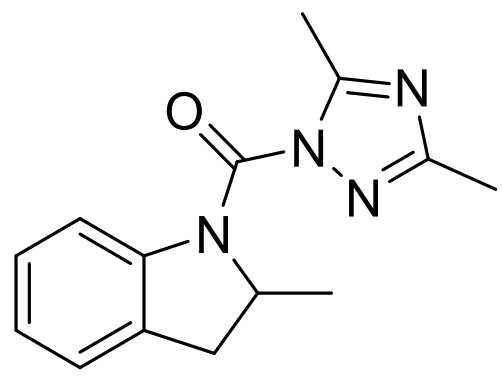

Yield 85%; Light yellow solid; m.p.64.1 – 65.7 °C.

^{1}H NMR (500 MHz, $CDCl_3$) δ 7.22 (d, *J* = 7.5 Hz, 2H), 7.09 (m, 1H), 5.26 (s, 1H), 3.46 (dd, *J* = 15.8, 9.1 Hz, 1H), 2.70 (dd, *J* = 15.9, 2.2 Hz, 1H), 2.63 (s, 3H), 2.38 (s, 1H), 2.37 (s, 3H), 1.16 (d, *J* = 6.3 Hz, 2H).

^{13}C NMR (126 MHz, $CDCl_3$) δ 159.9, 149.1, 147.8, 140.9, 131.1, 127.7, 125.4, 125.1, 117.3, 57.8, 36.3, 21.4, 14.0, 14.0.

HRMS (ESI) m/z Calcd for $C_{14}H_{17}N_4O$ $[M+H]^+$ 257.13968; Found 257.13980.

(3,5-dibromo-1*H*-1,2,4-triazol-1-yl)(2-methylindolin-1-yl)methanone (DK10)

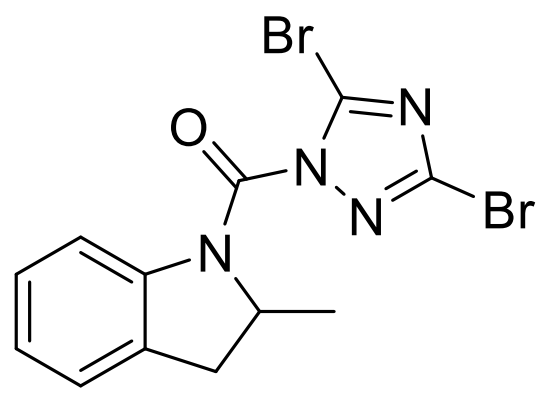

Yield 79%; Lilac solid; m.p.115.3 – 116.7 °C.

^{1}H NMR (500 MHz, $CDCl_3$) δ 8.10 – 8.04 (m, 1H), 7.28 (d, *J* = 14.6 Hz, 1H), 7.16 (d, *J* = 7.0 Hz, 1H), 5.02 – 4.79 (m, 1H), 3.50 (dd, *J* = 15.9, 9.0 Hz, 1H), 3.13 (dd, *J* = 7.4, 4.6 Hz, 1H), 2.73 (dd, *J* = 16.0, 2.1 Hz, 1H), 1.22 – 1.13 (m, 3H).

^{13}C NMR (126 MHz, $CDCl_3$) δ 144.3, 141.5, 140.0, 131.2, 128.1, 128.1, 126.2, 125.6, 117.8, 58.0, 36.3, 21.9.

HRMS (ESI) m/z Calcd for $C_{12}H_{10}Br_2N_4ONa$ $[M+Na]^+$ 406.91135; Found 406.91078.

indolin-1-yl(2-methyl-1*H*-imidazol-1-yl)methanone (DK11)

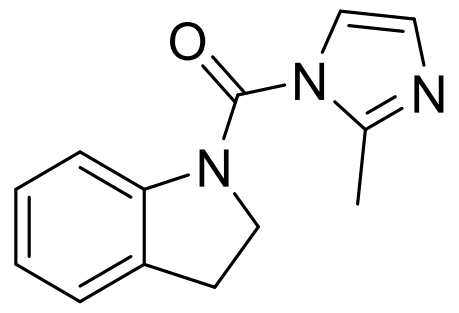

Yield 54%; Gray solid; m.p.112.2 – 113.4 °C.

^{1}H NMR (500 MHz, $CDCl_3$) δ 7.23 (d, *J* = 7.4 Hz, 1H), 7.2 – 7.0 (m, 3H), 7.0 (d, *J* = 1.6 Hz, 1H), 6.96 (d, *J* = 1.6 Hz, 1H), 4.06 (m, 2H), 3.20 – 3.13 (m, 2H), 2.47 (s, 3H).

^{13}C NMR (126 MHz, $CDCl_3$) δ 149.2, 145.5, 141.0, 132.4, 128.3, 127.9, 125.4, 125.1, 117.2, 115.9, 50.5, 27.9, 14.3.

HRMS (ESI) m/z Calcd for $C_{13}H_{14}N_3O$ $[M+H]^+$ 228.11313; Found 228.11355.

(2-methyl-1*H*-imidazol-1-yl)(2-methylindolin-1-yl)methanone (DK12)

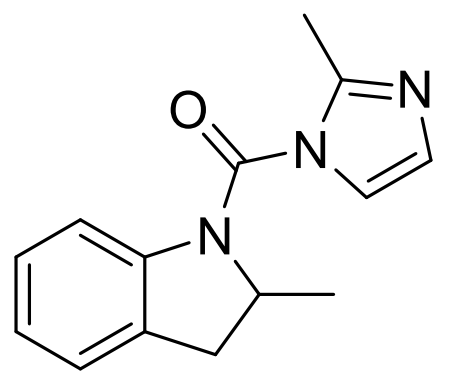

Yield 68%; Light yellow oily.

^{1}H NMR (500 MHz, $CDCl_3$) δ 7.23 – 7.20 (m, 1H), 7.08 – 7.01 (m, 3H), 7.00 – 6.90 (m, 2H), 4.70 (m, 1H), 3.42 (dd, *J* = 15.9, 8.9 Hz, 1H), 2.71 (dd, *J* = 15.9, 2.7 Hz, 1H), 2.42 (s, 3H), 1.32 (d, *J* = 6.4 Hz, 3H).

^{13}C NMR (126 MHz, $CDCl_3$) δ 148.6, 145.0, 139.8, 131.3, 128.4, 128.0, 125.9, 125.0, 117.5, 115.3, 58.2, 35.9, 20.3, 14.1.

HRMS (ESI) m/z Calcd for $C_{14}H_{16}N_3O$ $[M+H]^+$ 242.12878; Found 242.12875.

(4-bromo-1*H*-imidazol-1-yl)(indolin-1-yl)methanone (DK13)

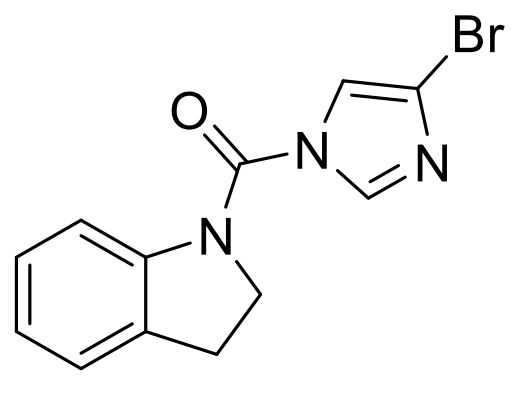

Yield 85%; White solid; m.p.136.8 – 137.6 °C.

^{1}H NMR (500 MHz, CDCl$_3$) δ 7.91 (d, *J* = 1.5 Hz, 1H), 7.45 (s, 1H), 7.33 (d, *J* = 1.6 Hz, 1H), 7.27 – 7.24 (m, 1H), 7.22 (m, 1H), 7.11 (m, 1H), 4.19 (t, *J* = 8.1 Hz, 2H), 3.21 (t, *J* = 8.1 Hz, 2H).

^{13}C NMR (126 MHz, CDCl$_3$) δ 147.0, 141.2, 136.7, 132.1, 127.9, 125.4, 125.4, 117.3, 116.9, 116.8, 51.3, 28.6.

HRMS (ESI) m/z Calcd for $C_{12}H_{11}BrN_3O$ [M+H]$^+$ 292.00800; Found 292.00810.

(4-bromo-1*H*-imidazol-1-yl)(2-methylindolin-1-yl)methanone (DK14)

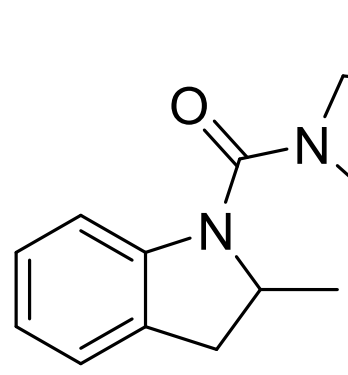

Yield 80%; Gray solid; m.p.54.4 – 56.0 °C.

^{1}H NMR (500 MHz, CDCl$_3$) δ 7.83 (d, *J* = 1.5 Hz, 1H), 7.28 – 7.24 (m, 2H), 7.13 (m, 1H), 7.07 (m, 1H), 6.86 (d, *J* = 7.9 Hz, 1H), 4.72 (m, 1H), 3.44 (dd, *J* = 15.8, 8.6 Hz, 1H), 2.73 (dd, *J* = 15.8, 2.7 Hz, 1H), 1.33 (d, *J* = 6.3 Hz, 3H).

^{13}C NMR (126 MHz, CDCl$_3$) δ 146.5, 139.8, 136.6, 135.3, 131.4, 127.9, 126.1, 125.2, 117.0, 115.8, 59.2, 36.2, 20.2.

HRMS (ESI) m/z Calcd for $C_{13}H_{12}BrN_3ONa$ [M+Na]$^+$ 328.00559; Found 328.00546.

(5-chloro-1*H*-imidazol-1-yl)(indolin-1-yl)methanone (DK15)

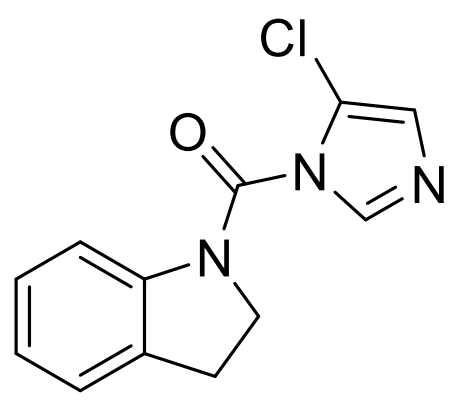

Yield 77%; White solid; m.p.118.9 – 120.2 °C.

^{1}H NMR (500 MHz, CDCl$_3$) δ 7.90 (s, 1H), 7.47 – 7.44 (m, 1H), 7.25 (dd, *J* = 4.8, 3.1 Hz, 2H), 7.22 (m, 1H), 7.10 (m, 1H), 4.19 (t, *J* = 8.1 Hz, 2H), 3.20 (t, *J* = 8.1 Hz, 2H).

^{13}C NMR (126 MHz, CDCl$_3$) δ 147.2, 141.2, 135.7, 132.1, 131.4, 127.9, 125.4, 125.4, 116.8, 113.5, 51.3, 28.6.

HRMS (ESI) m/z Calcd for $C_{12}H_{10}ClN_3ONa$ $[M+Na]^+$ 248.05851; Found 248.05786.

(1*H*-imidazol-1-yl)(2-methylindolin-1-yl)methanone (DK16)

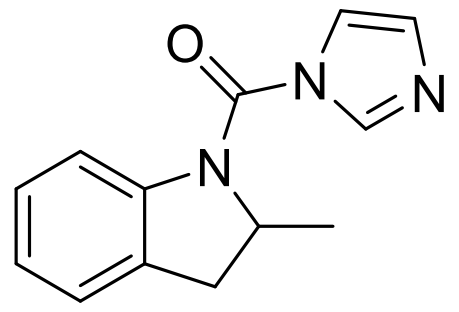

Yield 80%; Yellow oily.

^{1}H NMR (500 MHz, $CDCl_3$) δ 7.93 (s, 1H), 7.27 (m, 1H), 7.24 – 7.22 (m, 1H), 7.11 – 7.11 (m, 1H), 7.09 – 7.07 (m, 1H), 7.06 – 7.04 (m, 1H), 6.77 (d, *J* = 8.0 Hz, 1H), 4.73 (m, 1H), 3.43 (dd, *J* = 15.7, 8.6 Hz, 1H), 2.72 (dd, *J* = 15.7, 2.9 Hz, 1H), 1.33 (d, *J* = 6.3 Hz, 4H).

^{13}C NMR (126 MHz, $CDCl_3$) δ 147.9, 140.2, 136.7, 131.3, 130.0, 127.7, 125.9, 124.9, 117.7, 115.7, 59.0, 36.2, 20.1.

HRMS (ESI) m/z Calcd for $C_{13}H_{14}N_3O$ $[M+H]^+$ 228.11313; Found 228.11328.

indolin-1-yl(4-methyl-1*H*-imidazol-1-yl)methanone (DK17)

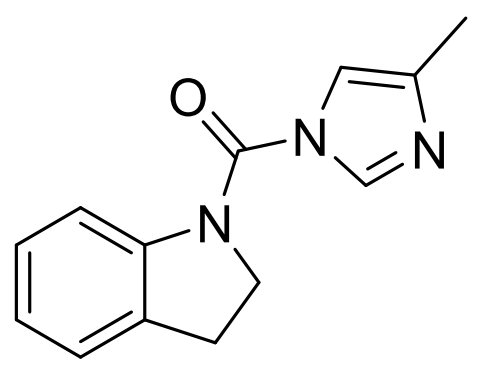

Yield 41%; White solid; m.p.105.5 – 107.2 °C.

^{1}H NMR (500 MHz, $CDCl_3$) δ 7.92 (d, *J* = 1.4 Hz, 1H), 7.40 (s, 1H), 7.24 (d, *J* = 7.5 Hz, 1H), 7.19 (m, 1H), 7.07 (dd, *J* = 8.1, 7.1 Hz, 1H), 7.05 (m, 1H), 4.18 (t, *J* = 8.1 Hz, 2H), 3.18 (t, *J* = 8.1 Hz, 2H), 2.25 (d, *J* = 1.2 Hz, 3H).

^{13}C NMR (126 MHz, $CDCl_3$) δ 148.5, 141.6, 139.3, 136.3, 132.1, 127.7, 125.2, 124.9, 116.6, 113.7, 51.2, 28.5, 13.7.

HRMS (ESI) m/z Calcd for $C_{13}H_{14}N_3O$ $[M+H]^+$ 228.11313; Found 228.11286.

(4-methyl-1*H*-imidazol-1-yl)(2-methylindolin-1-yl)methanone (DK18)

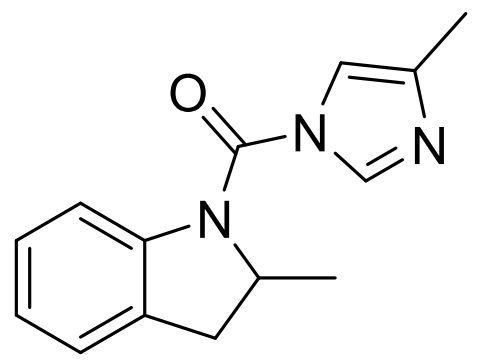

Yield 64%; Light yellow oily.

^{1}H NMR (500 MHz, $CDCl_3$) δ 7.83 (s, 1H), 7.22 (d, *J* = 7.3 Hz, 1H), 7.10 (m, 1H), 7.04 (d, *J* = 7.2 Hz, 1H), 6.97 (s, 1H), 6.84 (d, *J* = 8.0 Hz, 1H), 4.72 (m, 1H), 3.41 (dd, *J* = 15.7, 8.5 Hz, 1H), 2.71 (dd, *J* = 15.8, 3.1 Hz, 1H), 2.24 (s, 3H), 1.32 (d, *J* = 6.4 Hz, 3H).

^{13}C NMR (126 MHz, $CDCl_3$) δ 148.1, 139.3, 136.2, 131.2, 127.7, 125.9, 124.6, 118.6, 115.7, 113.8, 58.9, 36.2, 20.2, 13.7.

HRMS (ESI) m/z Calcd for $C_{14}H_{16}N_3O$ $[M+H]^+$ 242.12878; Found 242.12902.

indolin-1-yl(4-iodo-1*H*-imidazol-1-yl)methanone (DK19)

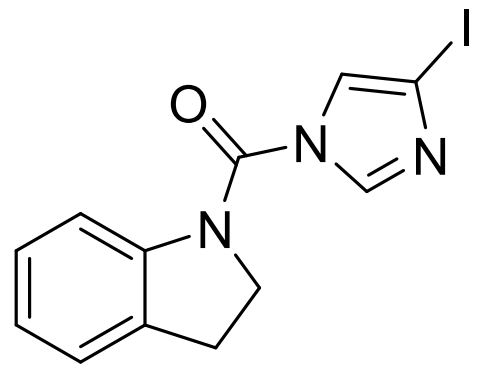

Yield 79%; White solid; m.p.148.1 – 149.3 °C.

^{1}H NMR (500 MHz, $CDCl_3$) δ 7.90 (s, 1H), 7.44 (s, 2H), 7.26 – 7.24 (m, 1H), 7.21 (m, 1H), 7.10 (m, 1H), 4.18 (t, *J* = 8.1 Hz, 2H), 3.20 (t, *J* = 8.1 Hz, 2H).

^{13}C NMR (126 MHz, $CDCl_3$) δ 146.9, 141.2, 138.2, 132.1, 127.9, 125.4, 125.4, 123.1, 116.8, 84.0, 51.2, 28.5.

HRMS (ESI) m/z Calcd for $C_{12}H_{11}IN_3O$ $[M+H]^+$ 339.99413; Found 339.99411.

(4-iodo-1*H*-imidazol-1-yl)(2-methylindolin-1-yl)methanone (DK20)

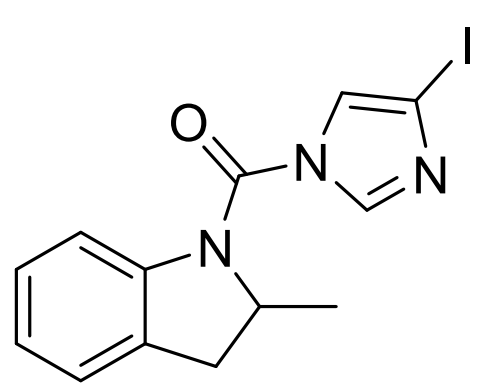

Yield 68%; White solid; m.p.96.9 – 98.2 °C.

^{1}H NMR (500 MHz, $CDCl_3$) δ 7.81 (d, *J* = 1.4 Hz, 1H), 7.37 (d, *J* = 1.4 Hz, 1H), 7.25 – 7.23 (m, 1H), 7.13 (m, 1H), 7.07 (m, 1H), 6.84 (d, *J* = 8.0 Hz, 1H), 4.71 (m, 1H), 3.43 (dd, *J* = 15.7, 8.6 Hz, 1H), 2.72 (dd, *J* = 15.7, 2.7 Hz, 1H), 1.32 (d, *J* = 6.3 Hz, 3H).

^{13}C NMR (126 MHz, $CDCl_3$) δ 146.4, 139.9, 138.1, 131.3, 127.9, 126.0, 125.2, 123.1, 115.8, 83.8, 59.1, 36.2, 20.2.

HRMS (ESI) m/z Calcd for $C_{13}H_{13}IN_3O$ $[M+H]^+$ 354.00978; Found 354.00969.

(4-ethyl-1*H*-imidazol-1-yl)(indolin-1-yl)methanone (DK21)

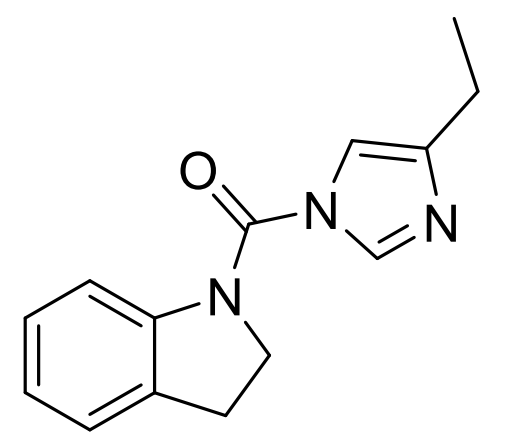

Yield 60%; Brown oily.

^{1}H NMR (500 MHz, $CDCl_3$) δ 7.22 (d, *J* = 7.3 Hz, 1H), 7.09 (m, 3H), 7.01 (d, *J* = 1.6 Hz, 1H), 6.98 (d, *J* = 1.6 Hz, 1H), 4.05 (t, *J* = 8.2 Hz, 2H), 3.16 (t, *J* = 8.3 Hz, 2H), 2.82 (q, *J* = 7.5 Hz, 2H), 1.29 (t, *J* = 7.6 Hz, 3H).

^{13}C NMR (126 MHz, $CDCl_3$) δ 150.3, 149.2, 141.0, 132.4, 128.3, 127.9, 125.4, 125.0, 117.1, 50.3, 27.9, 21.4, 12.3.

HRMS (ESI) m/z Calcd for $C_{14}H_{16}N_3O$ $[M+H]^+$ 242.12878; Found 242.12824.

(4-ethyl-1*H*-imidazol-1-yl)(2-methylindolin-1-yl)methanone (DK22)

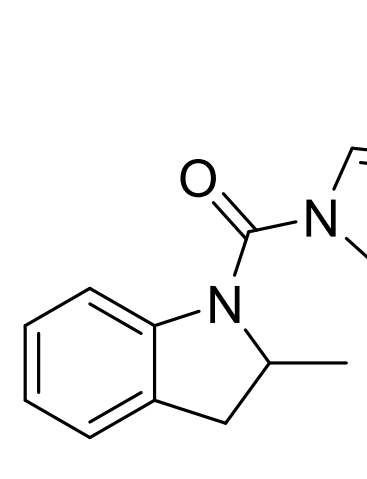

Yield 76%; Yellow oily.

^{1}H NMR (500 MHz, $CDCl_3$) δ 7.23 – 7.20 (m, 1H), 7.05 (dd, *J* = 7.0, 4.6 Hz, 2H), 7.00 – 6.95 (m, 2H), 6.90 – 6.82 (m, 1H), 4.70 (m, 1H), 2.80 (m, 2H), 2.74 – 2.60 (m, 2H), 1.33 (d, *J* = 6.5 Hz, 3H), 1.31 – 1.27 (m, 3H).

^{13}C NMR (126 MHz, $CDCl_3$) δ 149.9, 143.2, 128.3, 128.0, 127.3 (d, J = 27.6 Hz), 125.9, 124.9, 122.2, 117.4, 115.3, 58.2, 35.9, 21.3, 20.3, 12.2.

HRMS (ESI) m/z Calcd for $C_{15}H_{18}N_3O$ $[M+H]^+$ 256.14443; Found 256.14392.

indolin-1-yl(1*H*-1,2,4-triazol-1-yl)methanone (DK23)

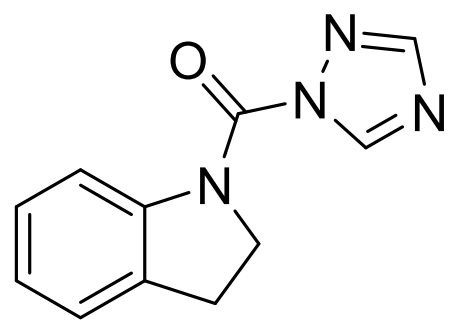

Yield 85%; White solid; m.p.93.8 – 95.3 °C.

^{1}H NMR (500 MHz, $CDCl_3$) δ 8.96 (s, 1H), 8.03 (s, 2H), 7.25 (dd, J = 7.6, 2.9 Hz, 2H), 7.12 (m, 1H), 4.57 (t, J = 8.3 Hz, 2H), 3.21 (t, J = 8.3 Hz, 2H).

^{13}C NMR (126 MHz, $CDCl_3$) δ 152.3, 146.5, 142.1, 132.1, 127.8, 125.4, 125.1, 117.6, 51.4, 28.7.

HRMS (ESI) m/z Calcd for $C_{11}H_{10}N_4ONa$ $[M+Na]^+$ 237.07468; Found 237.07471.

(1*H*-imidazol-1-yl)(indolin-1-yl)methanone (DK24)

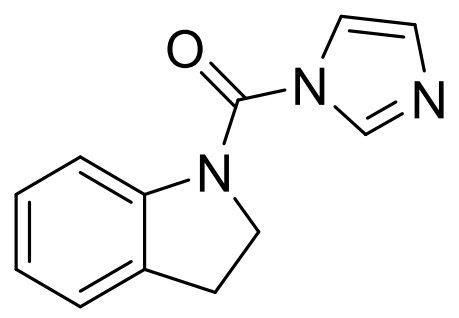

Yield 85%; Gray solid; m.p.67.5 – 69.3 °C.

^{1}H NMR (500 MHz, $CDCl_3$) δ 8.02 (s, 1H), 7.35 (d, J = 1.5 Hz, 1H), 7.24 – 7.20 (m, 1H), 7.18 (d, J = 7.7 Hz, 1H), 7.12 – 7.11 (m, 1H), 7.10 – 7.06 (m, 2H), 4.18 (t, J = 8.1 Hz, 2H), 3.18 (t, J = 8.1 Hz, 2H).

^{13}C NMR (126 MHz, $CDCl_3$) δ 148.3, 141.4, 136.8, 132.1, 130.0, 127.8, 125.3, 125.1, 122.5, 117.7, 51.2, 28.5.

HRMS (ESI) m/z Calcd for $C_{12}H_{12}N_3O$ $[M+H]^+$ 214.09748; Found 214.09699.

(2-methylindolin-1-yl)(1*H*-1,2,4-triazol-1-yl)methanone (DK25)

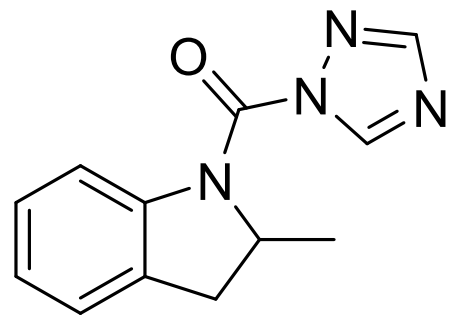

Yield 52%; White solid; m.p.81.8 – 82.6 °C.

^{1}H NMR (500 MHz, $CDCl_3$) δ 8.97 (s, 1H), 8.05 (s, 1H), 8.00 (s, 1H), 7.26 (d, *J* = 7.2 Hz, 2H), 7.13 (m, 1H), 5.68 (s, 1H), 3.47 (dd, *J* = 15.6, 8.9 Hz, 1H), 2.74 (dd, *J* = 15.8, 1.7 Hz, 1H), 1.24 (d, *J* = 6.2 Hz, 3H).

^{13}C NMR (126 MHz, $CDCl_3$) δ 152.5, 146.7, 146.3, 141.1, 131.3, 127.8, 125.5, 125.4, 118.2, 58.0, 36.6, 21.7.

HRMS (ESI) m/z Calcd for $C_{11}H_{12}N_4ONa$ $[M+Na]^+$ 251.09033; Found 251.09065.

(2-methylindolin-1-yl)(pyrrolidin-1-yl)methanone (DK26)

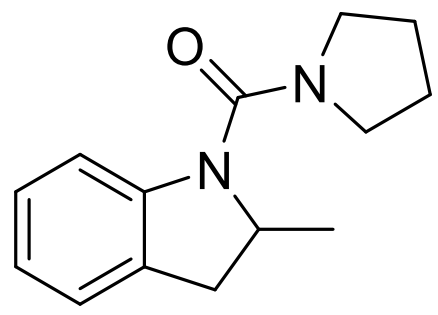

Yield 95%; White solid; m.p.62.3 – 63.9 °C.

^{1}H NMR (500 MHz, $CDCl_3$) δ 7.12 – 7.06 (m, 2H), 6.85 – 6.80 (m, 2H), 4.45 (m, 1H), 3.49 – 3.39 (m, 4H), 3.18 (dd, *J* = 15.2, 8.6 Hz, 1H), 2.64 (dd, *J* = 15.4, 7.1 Hz, 1H), 1.92 – 1.83 (m, 4H), 1.33 (d, *J* = 6.2 Hz, 3H).

^{13}C NMR (126 MHz, $CDCl_3$) δ 157.9, 144.3, 130.1, 127.0, 124.9, 120.9, 112.8, 57.7, 47.6, 47.6, 36.7, 25.5, 25.5, 20.1.

HRMS (ESI) m/z Calcd for $C_{14}H_{19}N_2O$ $[M+H]^+$ 231.14919; Found 231.14895.

indolin-1-yl(2-methylpyrrolidin-1-yl)methanone (DK27)

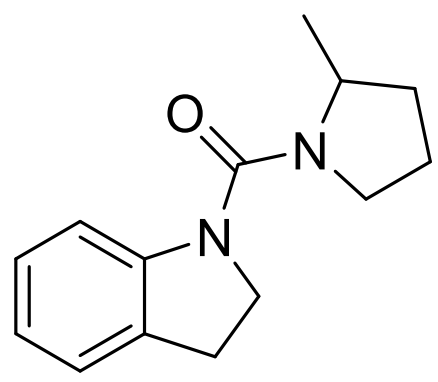

Yield 83%; Yellow oily.

^{1}H NMR (500 MHz, $CDCl_3$) δ 7.14 (d, *J* = 7.4 Hz, 1H), 7.12 – 7.10 (m, 2H), 6.86 (m, 1H), 4.13 (m, 1H), 3.97 (m, 1H), 3.83 (m, 1H), 3.46 (m, 1H), 3.32 (m, 1H), 3.14 – 3.09 (m, 1H), 2.97 (m, 1H), 2.14 (m, 1H), 1.89 (m, 1H), 1.80 – 1.71 (m, 1H), 1.54 – 1.47 (m, 1H), 1.24 (d, *J* = 5.9 Hz, 3H).

^{13}C NMR (126 MHz, $CDCl_3$) δ 158.6, 144.3, 131.5, 127.1, 124.8, 121.5, 114.5, 54.3, 50.0, 49.6, 33.7, 28.5, 25.4, 20.7.

HRMS (ESI) m/z Calcd for $C_{14}H_{19}N_2O$ $[M+H]^+$ 231.14919; Found 231.14914.

indolin-1-yl(3-methoxypyrrolidin-1-yl)methanone (DK28)

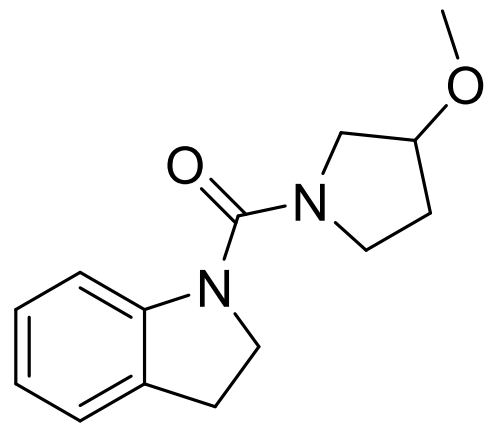

Yield 80%; Light yellow oily.

^{1}H NMR (500 MHz, $CDCl_3$) δ 7.14 (d, *J* = 7.5 Hz, 1H), 7.12 (d, *J* = 3.7 Hz, 2H), 6.87 (m, 1H), 3.99 – 3.93 (m, 2H), 3.93 – 3.88 (m, 1H), 3.64 – 3.56 (m, 2H), 3.50 – 3.45 (m, 2H), 3.33 (s, 3H), 3.11 – 3.00 (m, 2H), 2.07 – 2.02 (m, 1H), 1.97 – 1.91 (m, 1H).

^{13}C NMR (126 MHz, $CDCl_3$) δ 158.7, 144.2, 131.4, 127.1, 124.8, 121.6, 114.8, 79.4, 56.7, 52.5, 50.1, 45.9, 30.8, 28.6.

HRMS (ESI) m/z Calcd for $C_{14}H_{19}N_2O_2$ $[M+H]^+$ 247.14410; Found 247.14367.

(3-methoxypyrrolidin-1-yl)(2-methylindolin-1-yl)methanone (DK29)

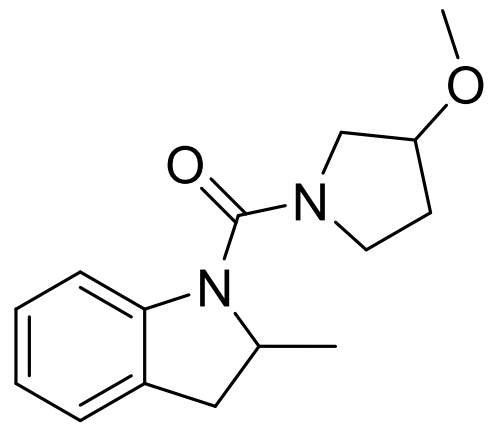

Yield 83%; Yellow oily.

^{1}H NMR (500 MHz, $CDCl_3$) δ 7.13 – 7.08 (m, 2H), 6.88 – 6.83 (m, 1H), 6.82 (d, *J* = 7.5 Hz, 1H), 4.45 (m, 1H), 3.97 (d, *J* = 4.3 Hz, 1H), 3.63 (m, 1H), 3.59 – 3.51 (m, 2H), 3.46 (m, 1H), 3.33 (d, *J* = 3.4 Hz, 3H), 3.18 (dd, *J* = 15.2, 8.6 Hz, 1H), 2.63 (m, 1H),

2.09 – 1.91 (m, 2H), 1.34 (dd, *J* = 6.2, 2.9 Hz, 3H).

^{13}C NMR (126 MHz, $CDCl_3$) δ 157.9, 144.4, 130.5, 127.2, 125.1, 121.0, 112.7, 79.3, 58.1, 56.6, 52.2, 45.6, 36.8, 30.9, 20.0.

HRMS (ESI) m/z Calcd for $C_{15}H_{21}N_2O_2$ $[M+H]^+$ 261.15975; Found 261.15938.

(3-fluoropyrrolidin-1-yl)(indolin-1-yl)methanone (DK30)

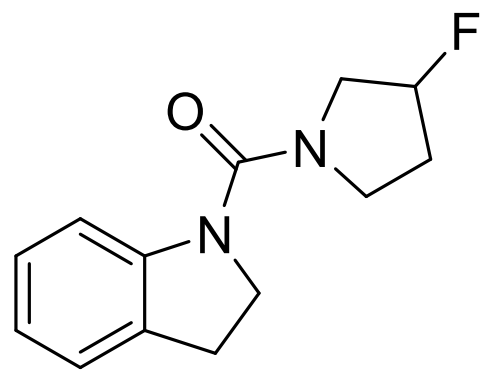

Yield 87%; Yellow oily.

^{1}H NMR (500 MHz, $CDCl_3$) δ 7.15 (d, *J* = 7.5 Hz, 1H), 7.13 (d, *J* = 4.2 Hz, 2H), 6.90 – 6.87 (m, 1H), 3.97 (m, 1H), 3.90 (m, 1H), 3.84 – 3.74 (m, 1H), 3.73 – 3.67 (m, 2H), 3.67 – 3.54 (m, 2H), 3.13 – 3.07 (m, 1H), 3.01 (m, 1H), 2.30 – 2.22 (m, 1H), 2.09 – 1.95 (m, 1H).

^{13}C NMR (126 MHz, $CDCl_3$) δ 158.5, 144.0, 131.5, 127.2, 124.8, 121.9, 114.8, 91.8, 54.2, 50.1, 45.8, 32.4, 28.6.

^{19}F NMR (471 MHz, $CDCl_3$) δ -177.86.

HRMS (ESI) m/z Calcd for $C_{13}H_{15}FN_2O$ $[M+H]^+$ 235.12411; Found 235.12441.

(3-fluoropyrrolidin-1-yl)(2-methylindolin-1-yl)methanone (DK31)

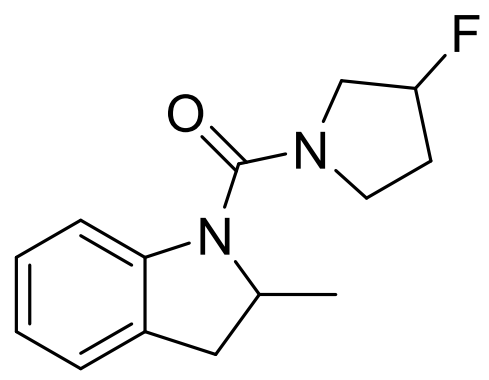

Yield 76%; Yellow oily.

^{1}H NMR (500 MHz, $CDCl_3$) δ 7.12 (dq, *J* = 14.8, 7.6 Hz, 2H), 6.91 – 6.79 (m, 2H), 5.33 – 5.19 (m, 1H), 4.51 – 4.42 (m, 1H), 3.84 – 3.73 (m, 1H), 3.73 – 3.60 (m, 2H), 3.58 – 3.53 (m, 1H), 3.20 (dd, *J* = 15.4, 8.5 Hz, 1H), 2.74 – 2.55 (m, 1H), 2.29 – 2.19 (m, 1H), 2.10 – 1.94 (m, 1H), 1.35 (t, *J* = 6.3 Hz, 3H).

^{13}C NMR (126 MHz, $CDCl_3$) δ 157.8, 144.3, 129.9, 127.3, 125.3, 121.2, 112.7, 92.0, 57.6, 54.0, 45.4, 36.8, 32.2, 20.0.

^{19}F NMR (471 MHz, $CDCl_3$) δ -177.80.

HRMS (ESI) m/z Calcd for $C_{14}H_{18}FN_2O$ $[M+H]^+$ 249.13976; Found 249.13929.

indolin-1-yl(pyrrolidin-1-yl)methanone (DK32)

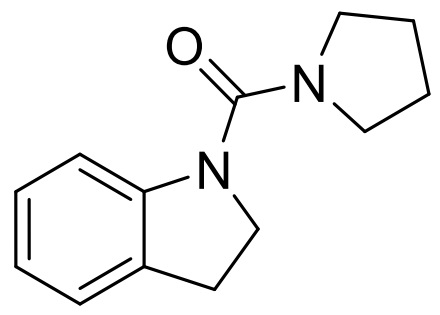

Yield 92%; White solid; m.p.77.7 – 79.0 °C.

^{1}H NMR (500 MHz, $CDCl_3$) δ 7.14 – 7.11 (m, 3H), 6.86 (m, 1H), 3.91 (t, *J* = 8.3 Hz, 2H), 3.46 – 3.42 (m, 4H), 3.04 (t, *J* = 8.3 Hz, 2H), 1.90 – 1.86 (m, 4H).

^{13}C NMR (126 MHz, $CDCl_3$) δ 158.7, 144.5, 131.3, 127.0, 124.7, 121.5, 114.8, 50.1, 47.9, 47.9, 28.7, 25.6, 25.6.

HRMS (ESI) m/z Calcd for $C_{13}H_{17}N_2O$ $[M+H]^+$ 217.13354; Found 217.13298.

2. ^{1}H NMR, ^{13}C NMR, ^{19}F NMR and HRMS spectra

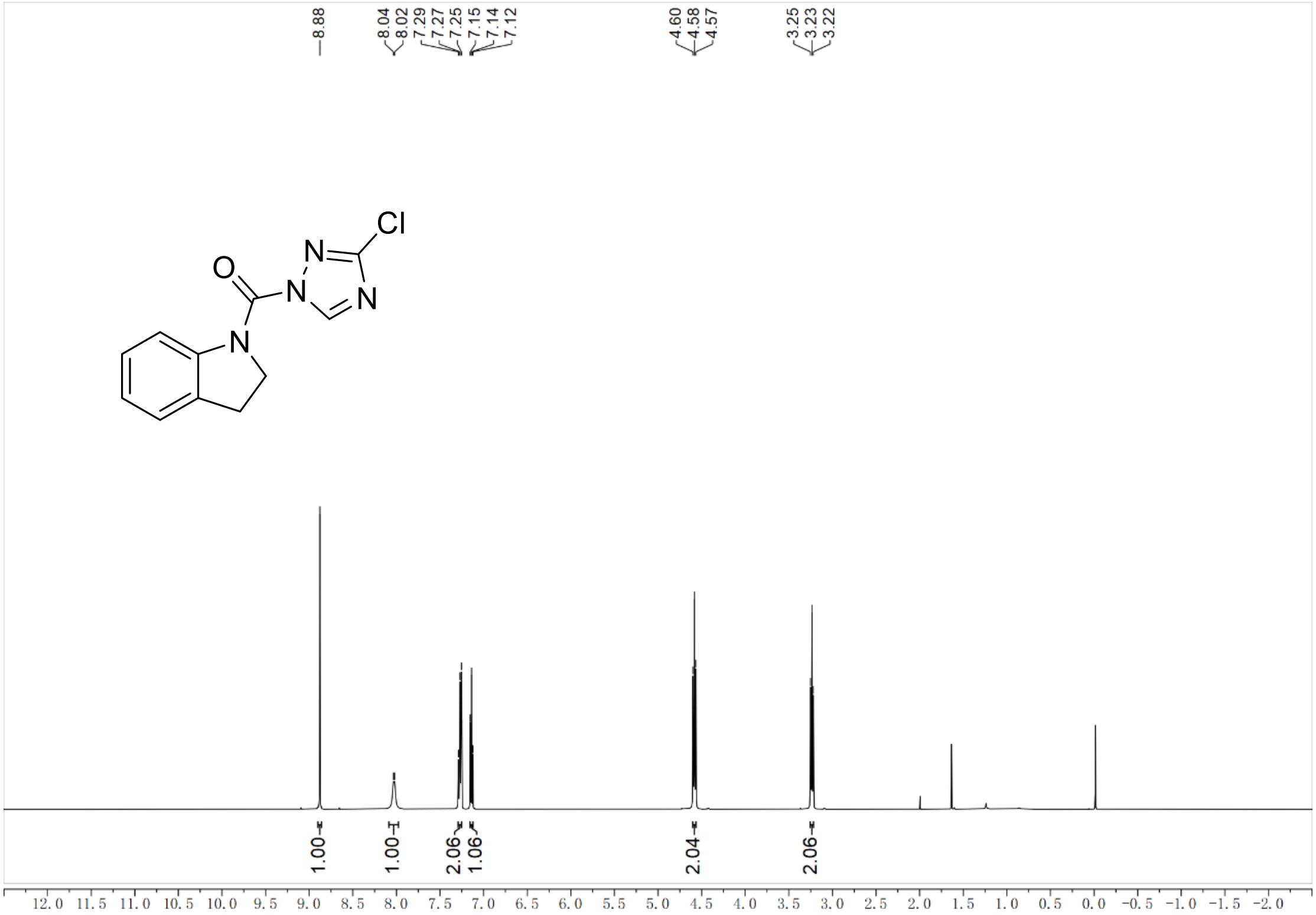

^{1}H NMR spectrum of compound DK1

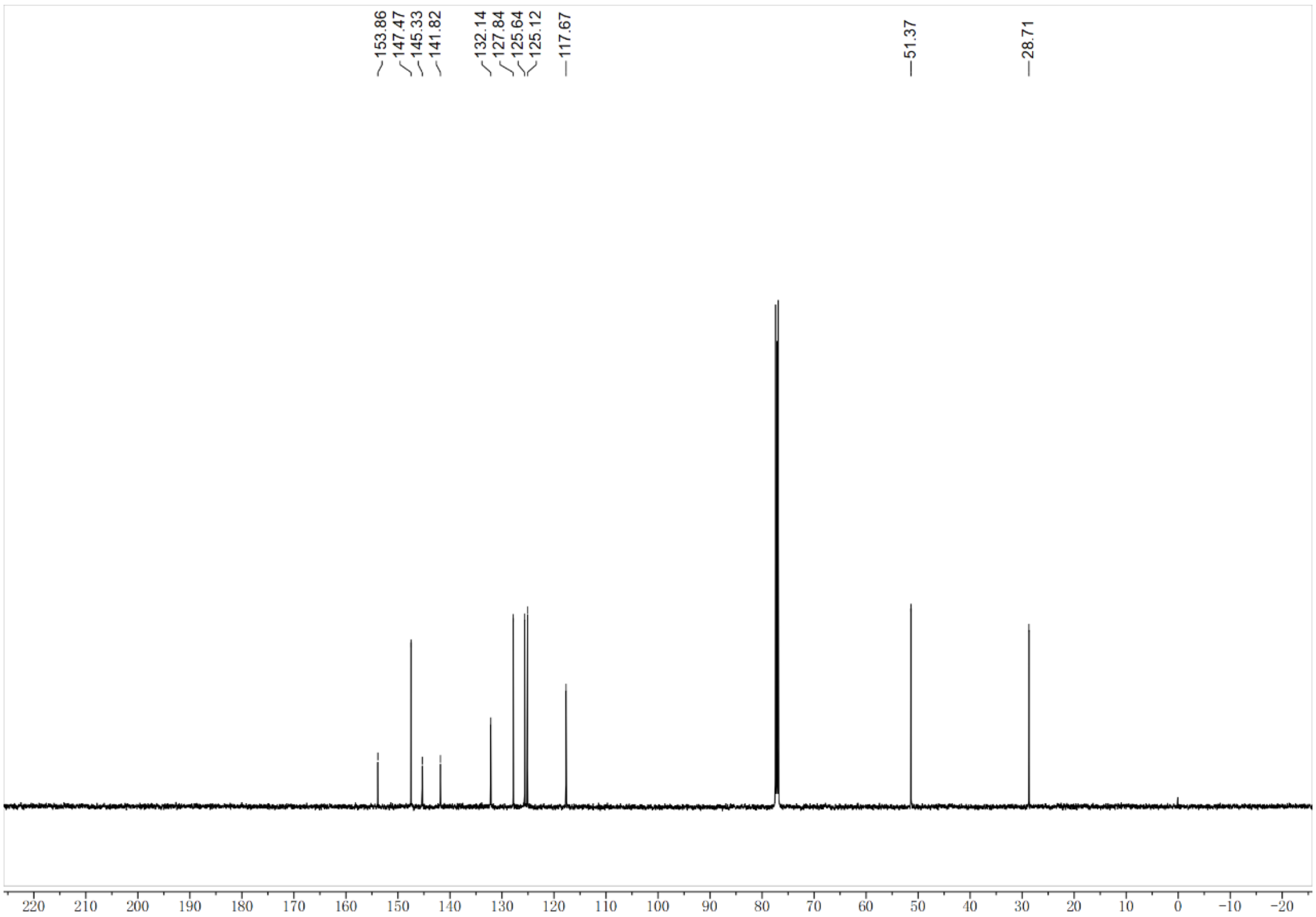

^{13}C NMR spectrum of compound DK1

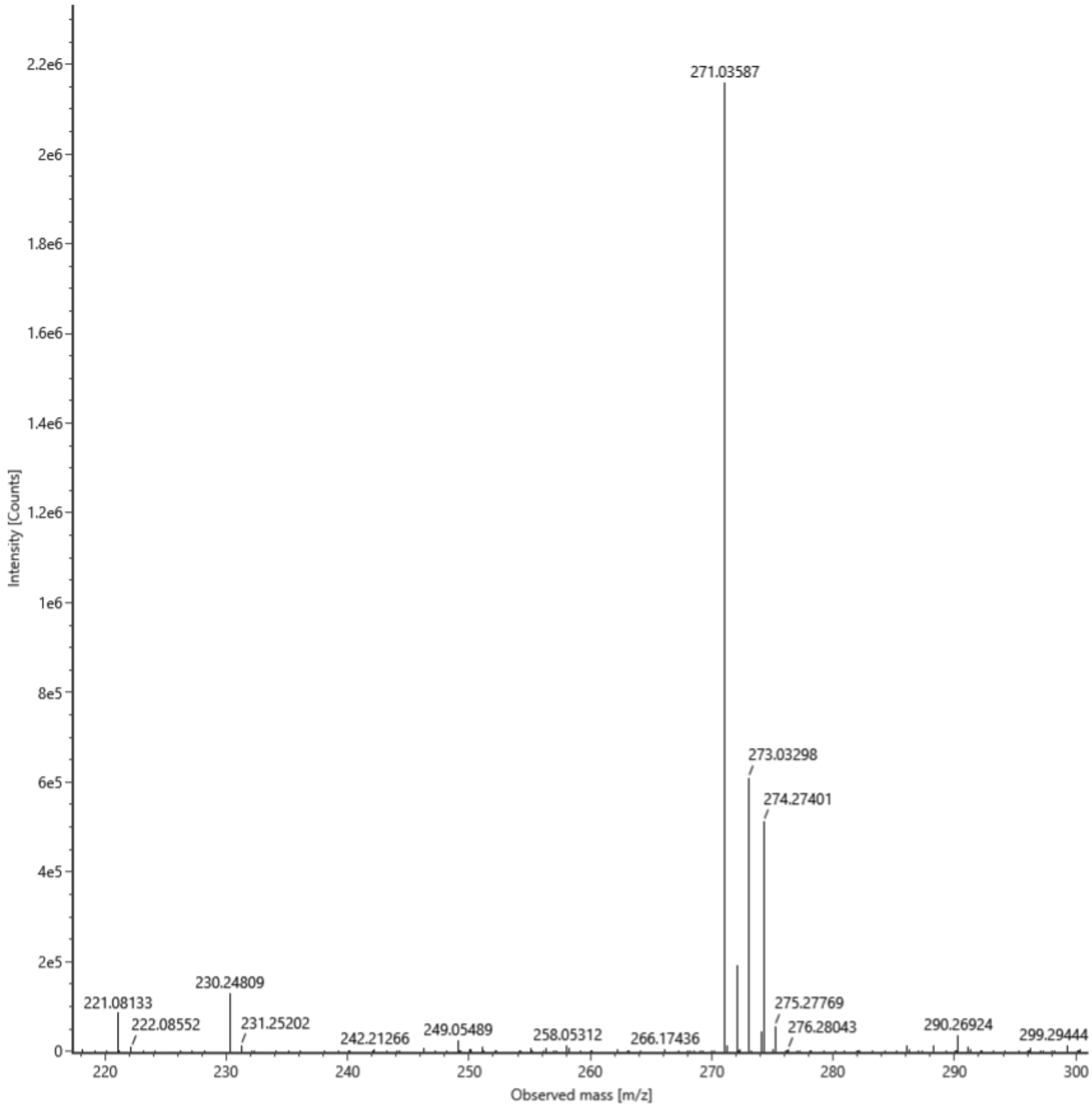

HRMS Spectrum of compound DK1

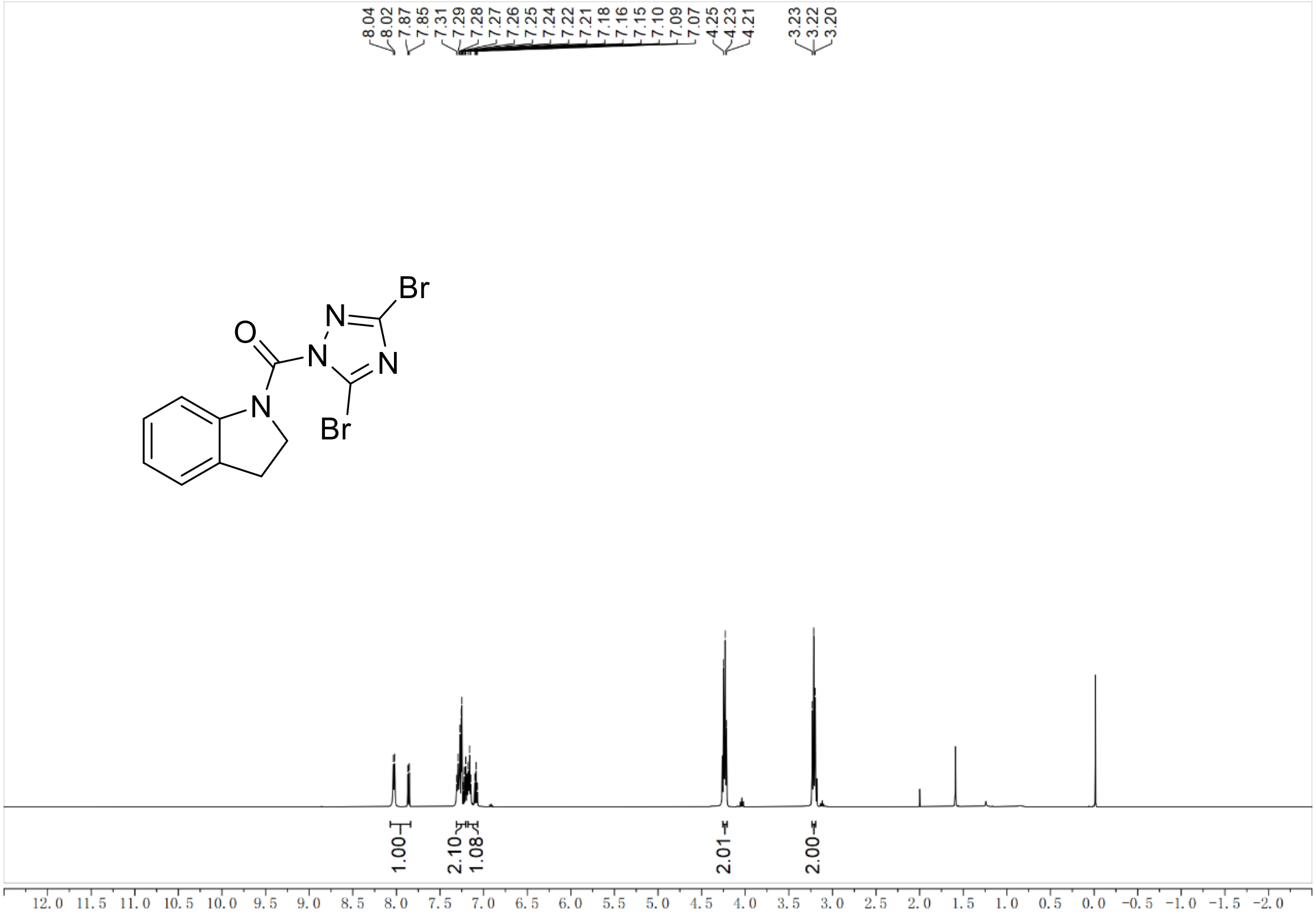

^{1}H NMR spectrum of compound DK2

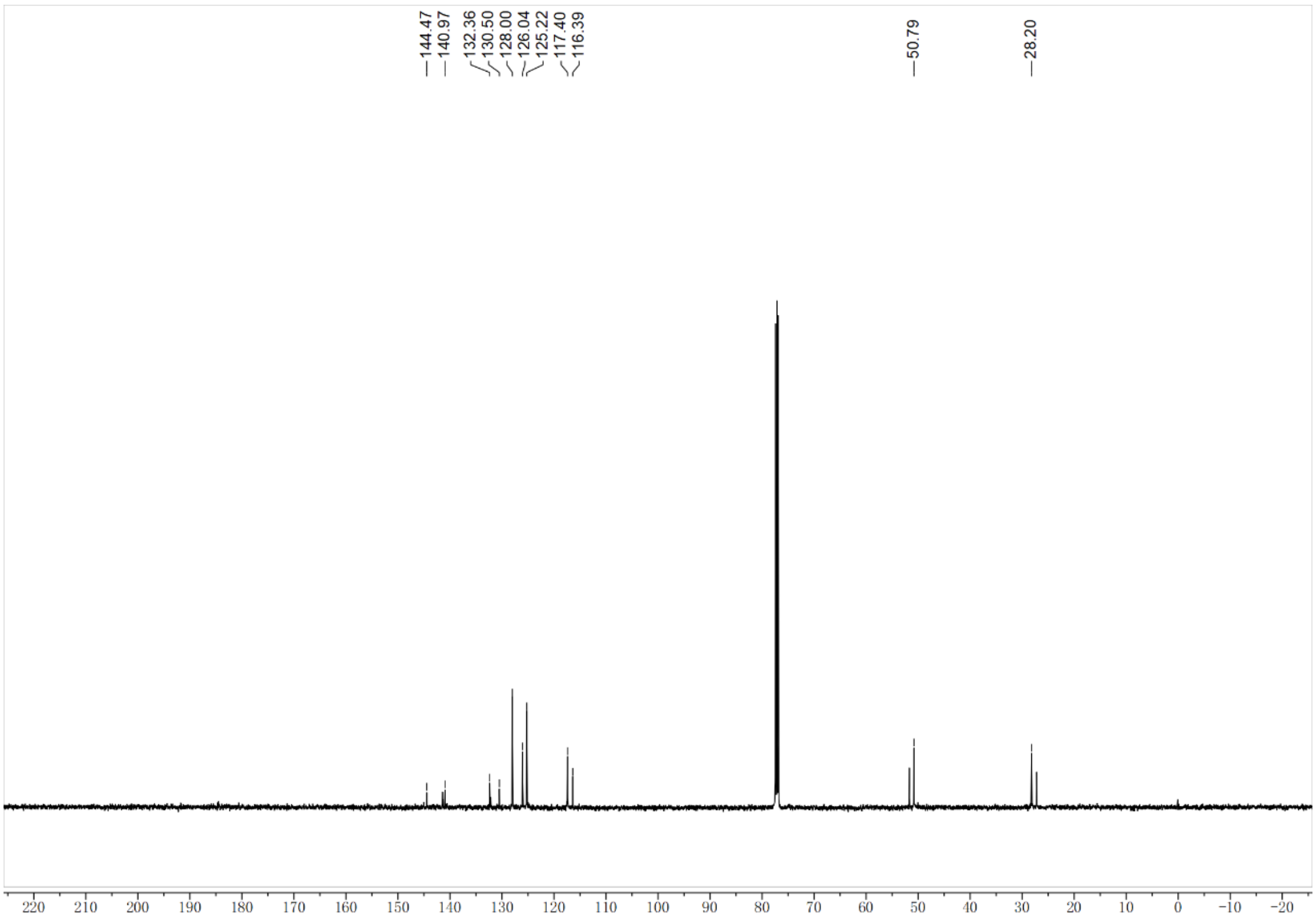

^{13}C NMR spectrum of compound DK2

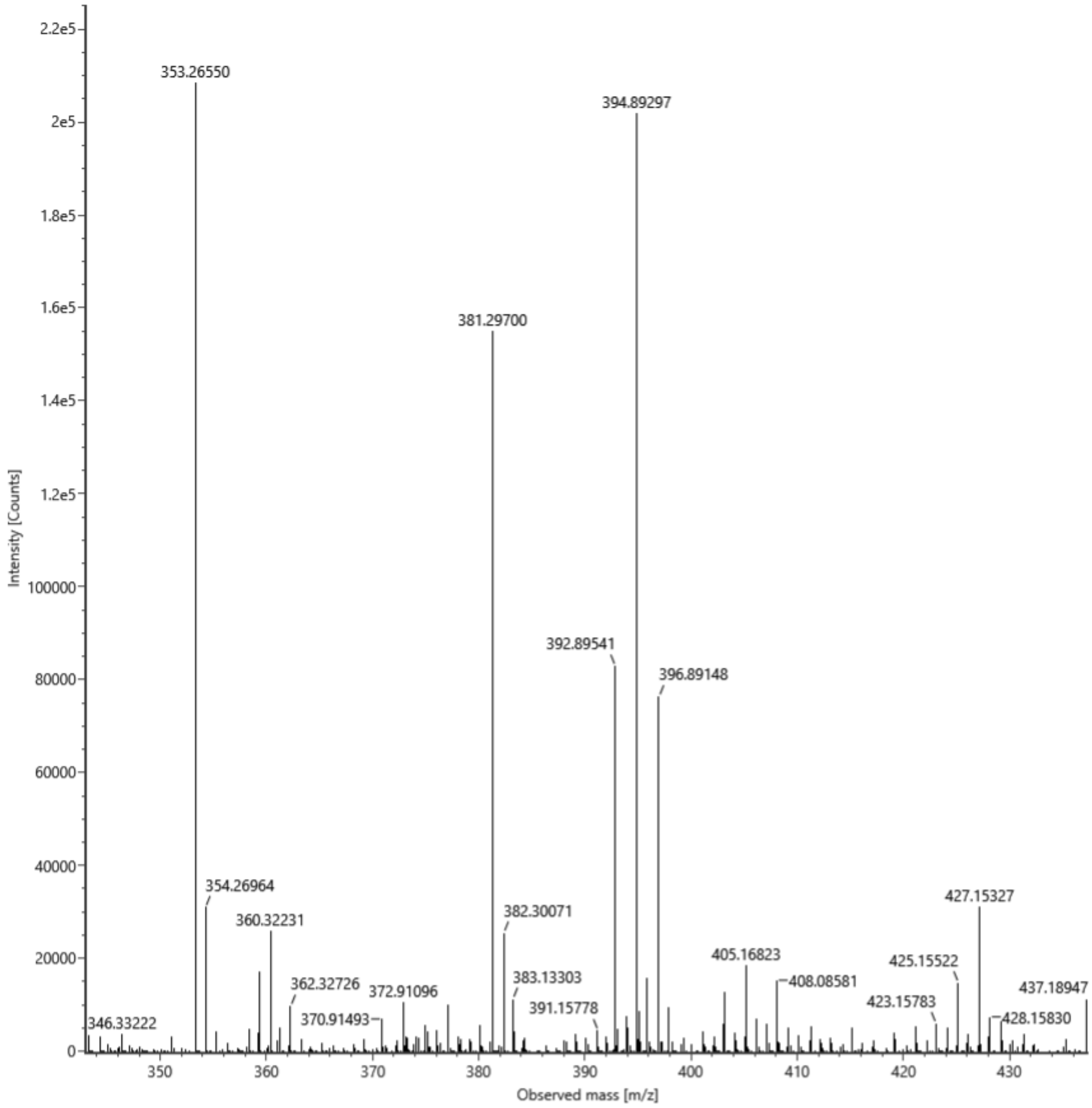

HRMS Spectrum of compound DK2

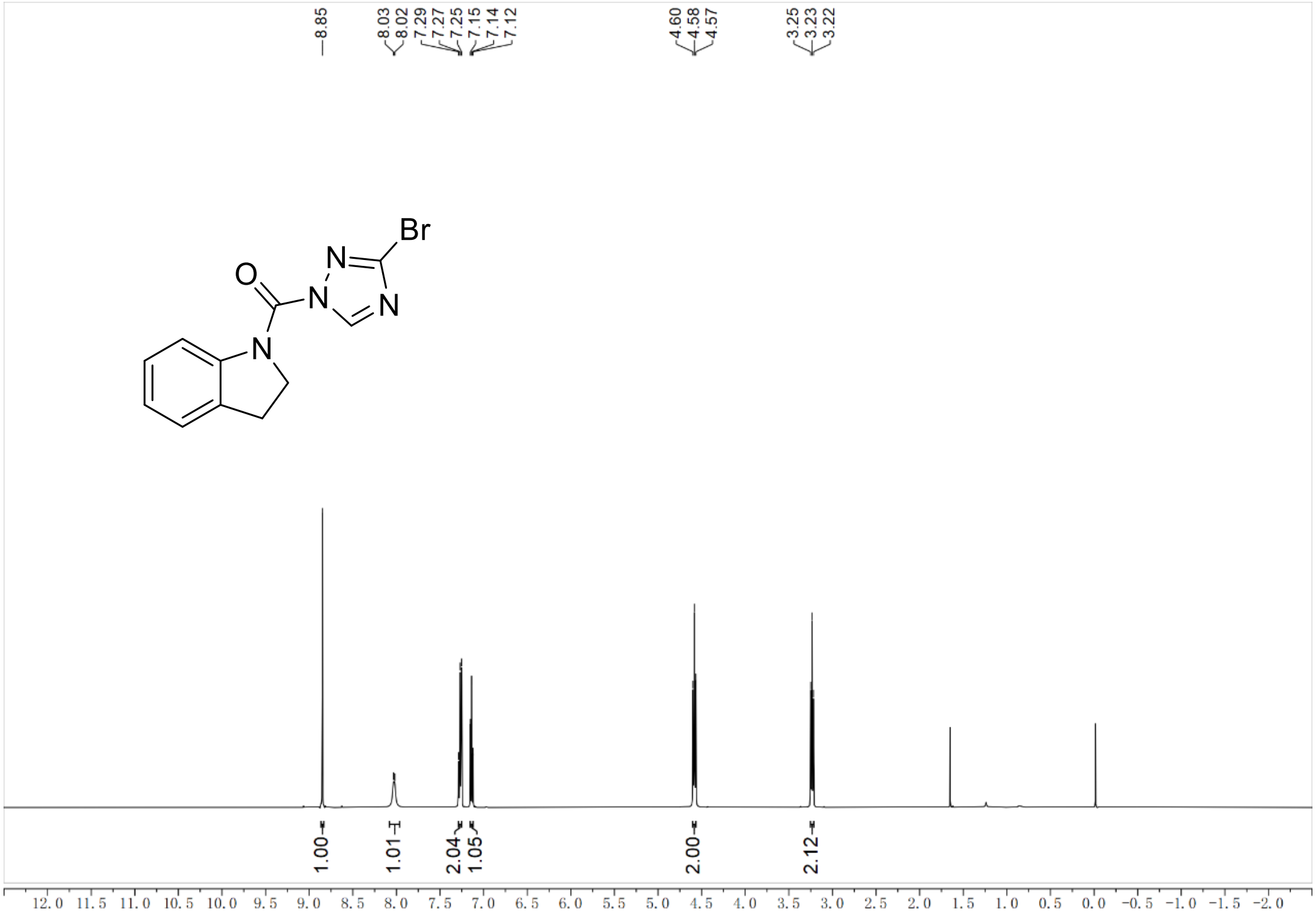

^{1}H NMR spectrum of compound DK3

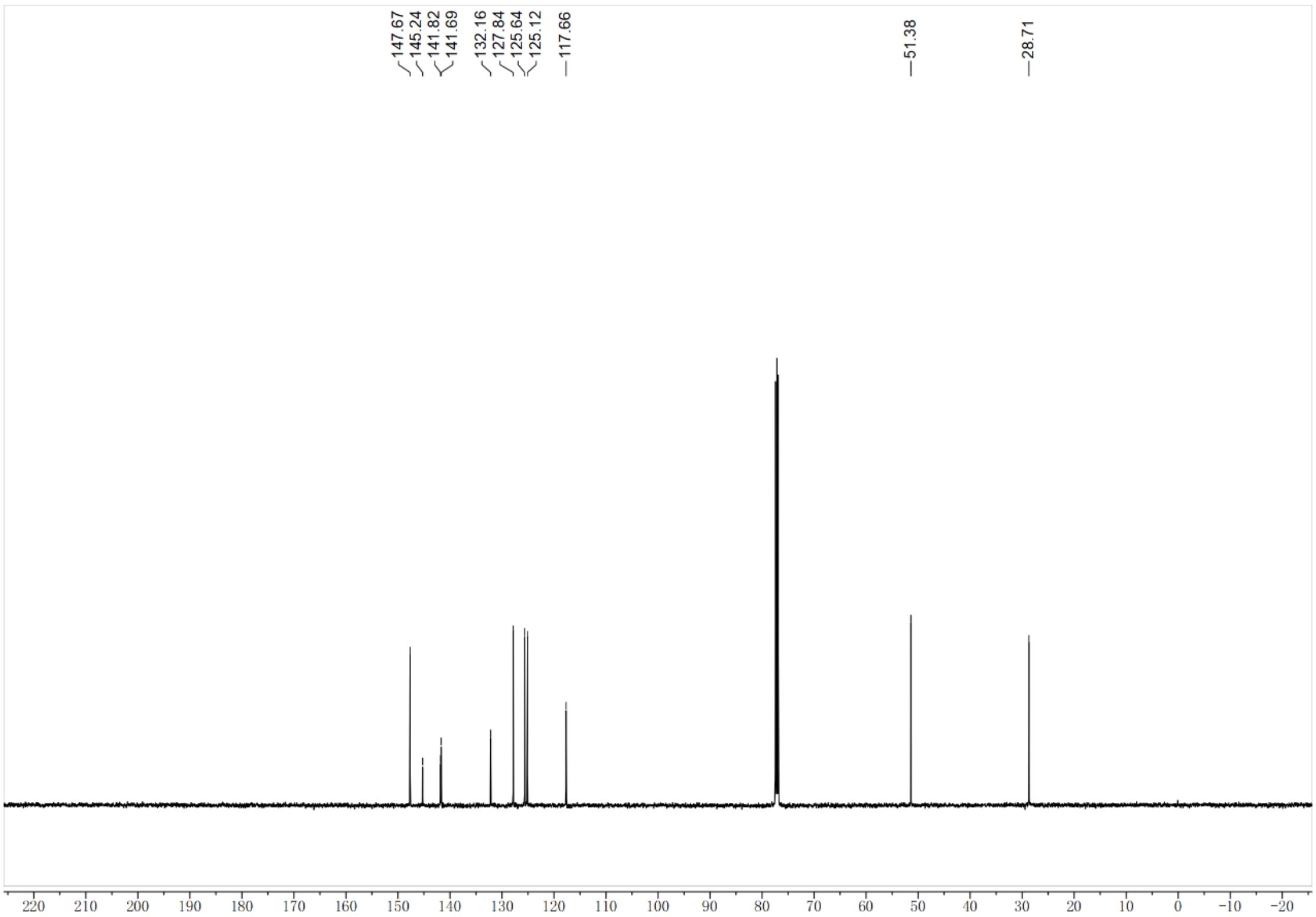

^{13}C NMR spectrum of compound DK3

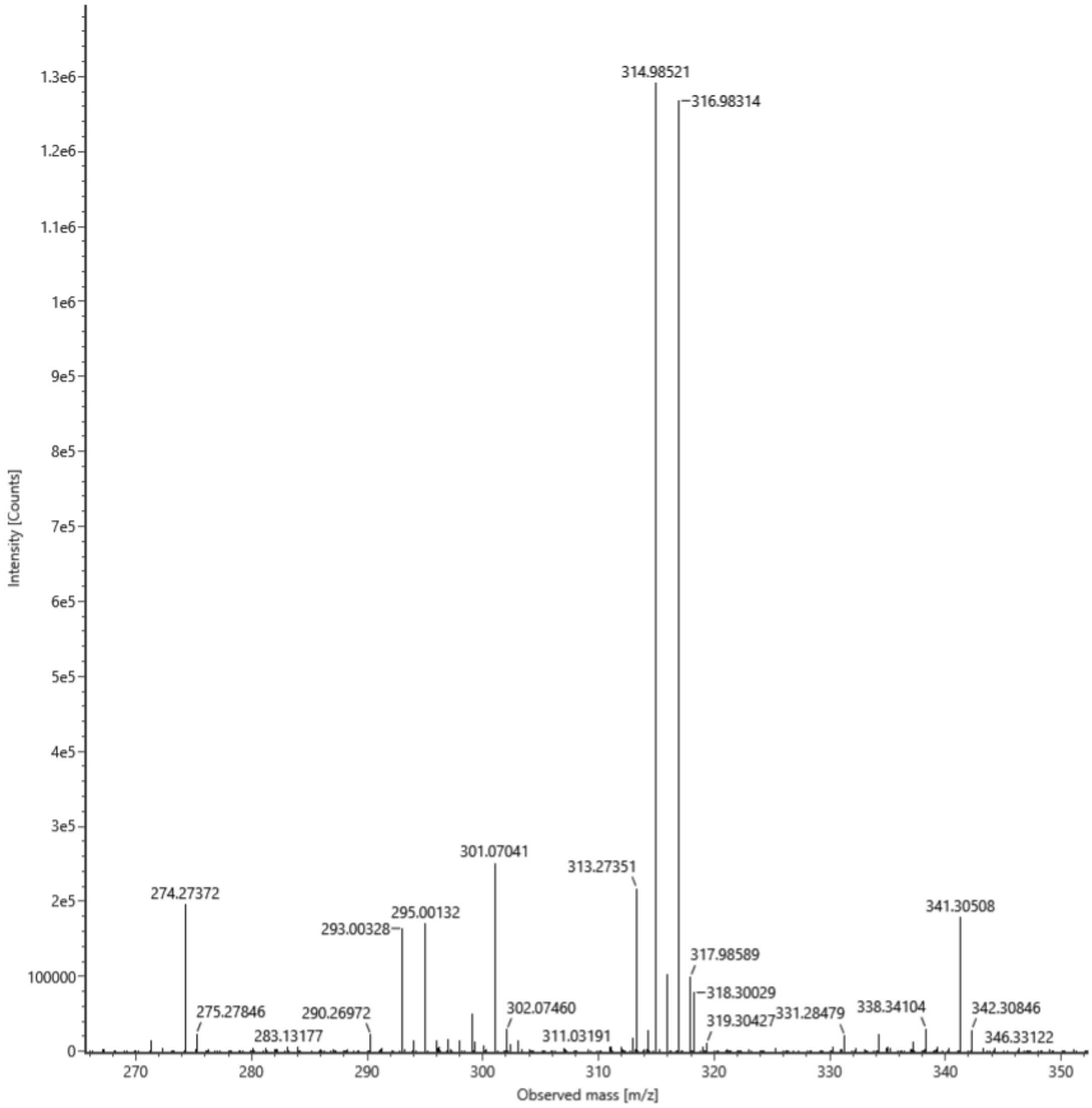

HRMS Spectrum of compound DK3

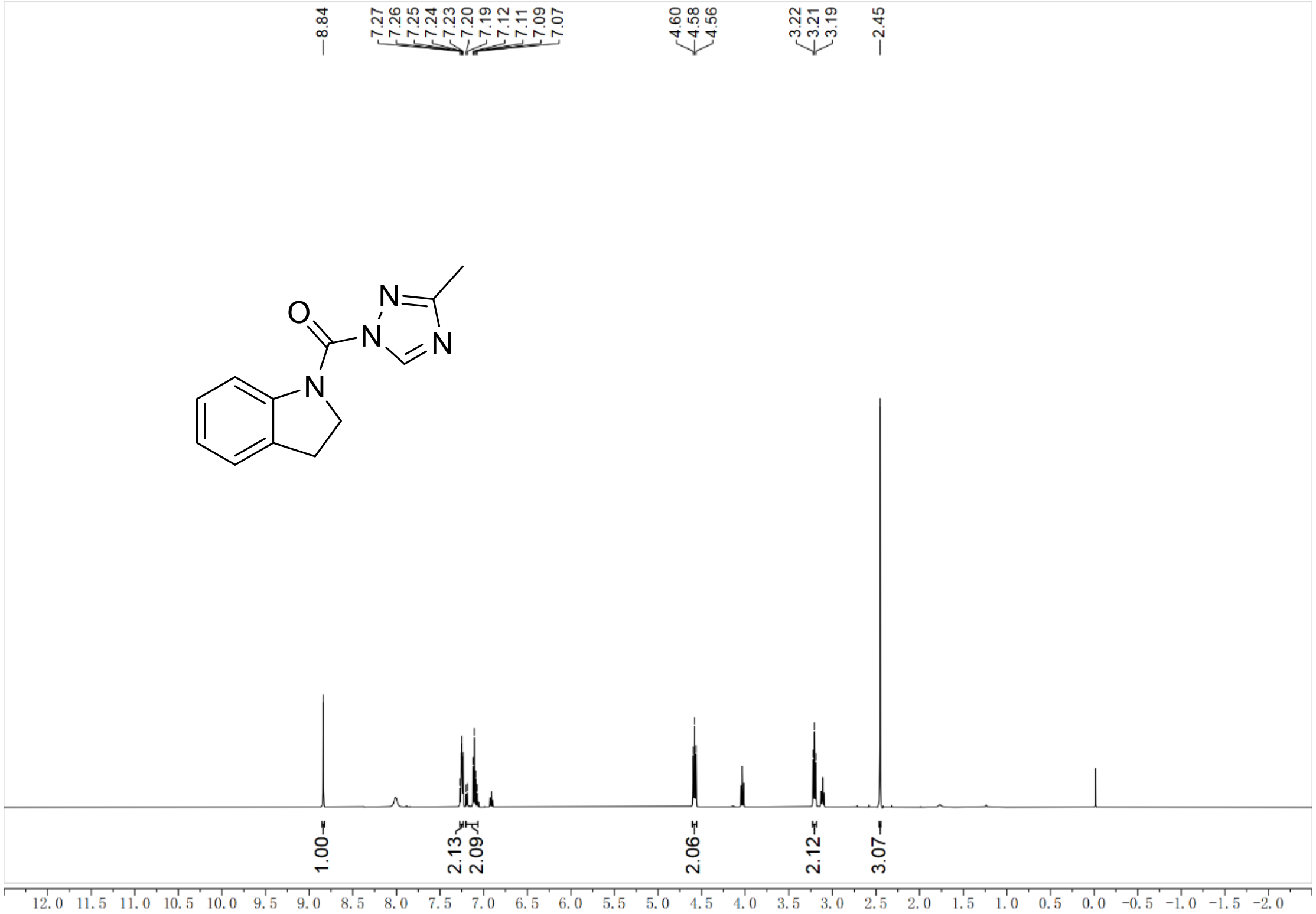

^1H NMR spectrum of compound DK4

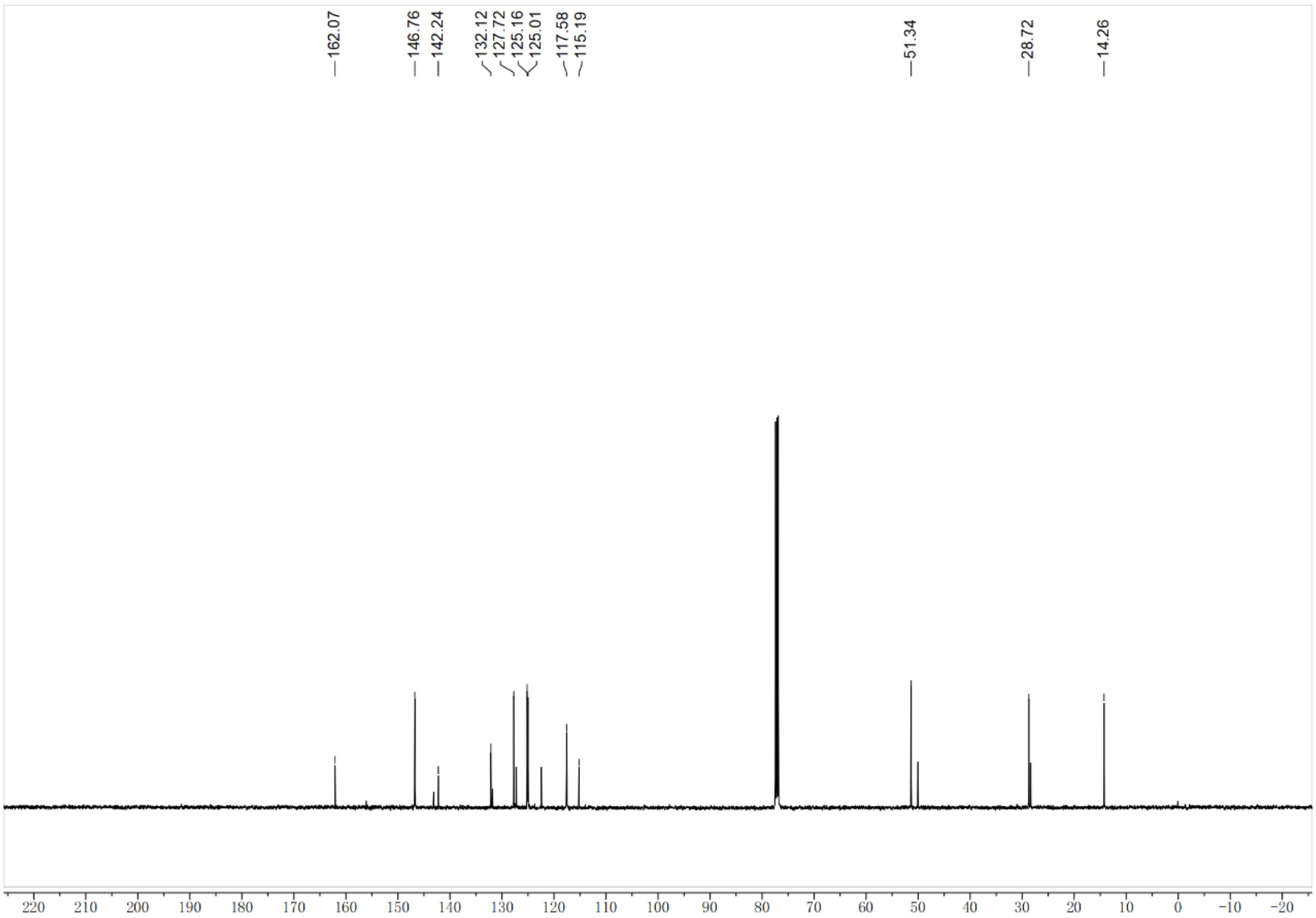

^{13}C NMR spectrum of compound DK4

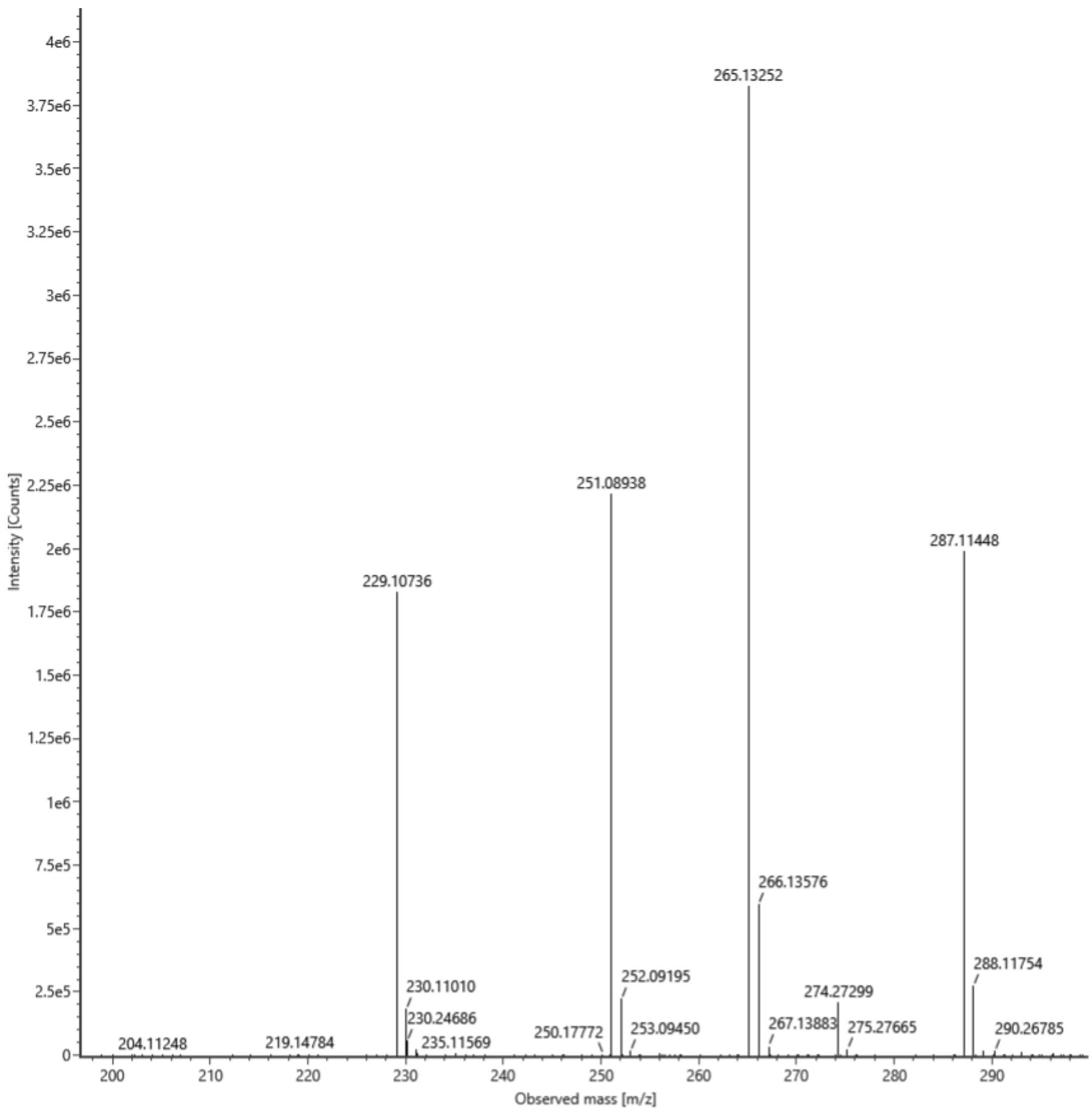

HRMS Spectrum of compound DK4

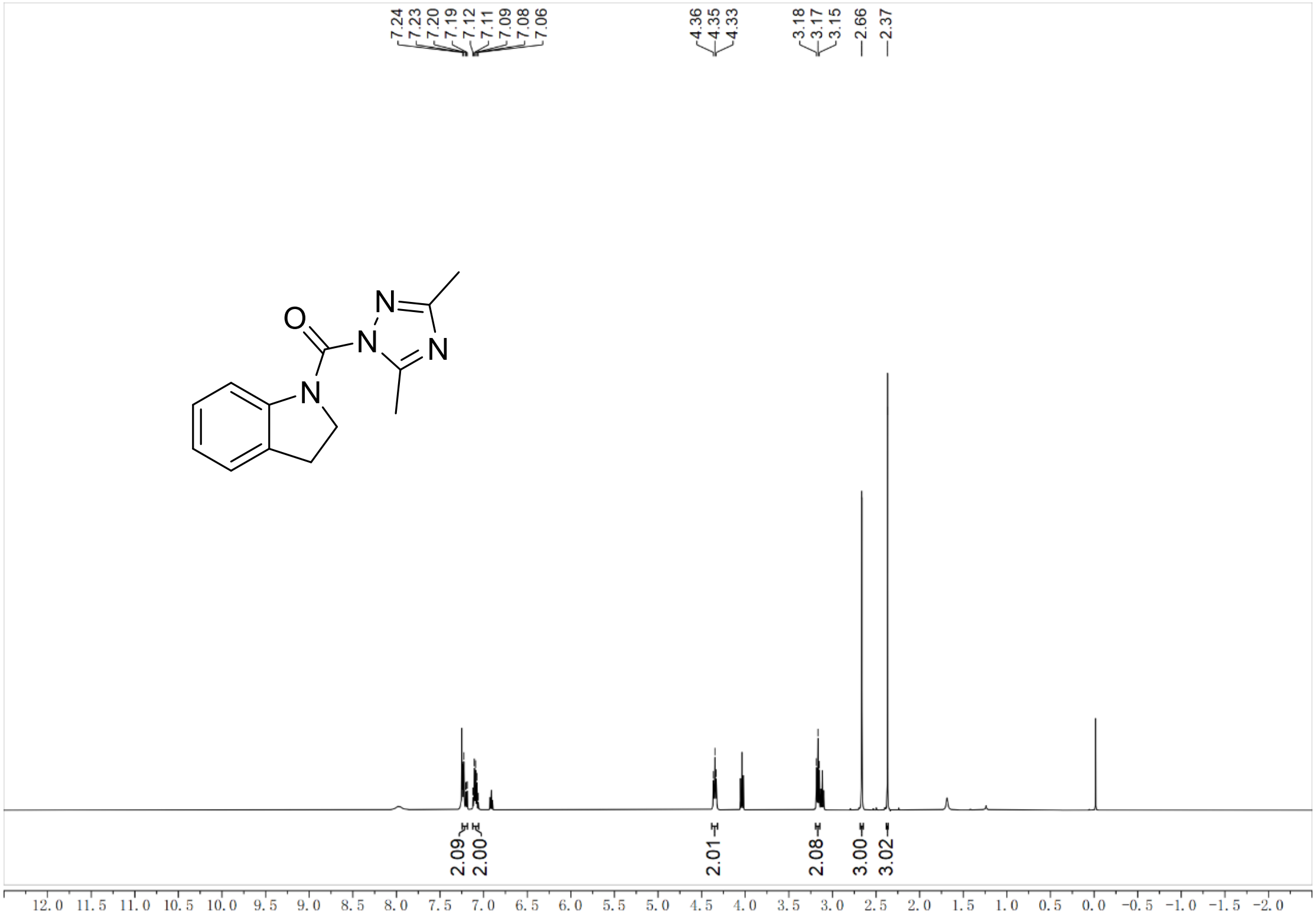

^{1}H NMR spectrum of compound DK5

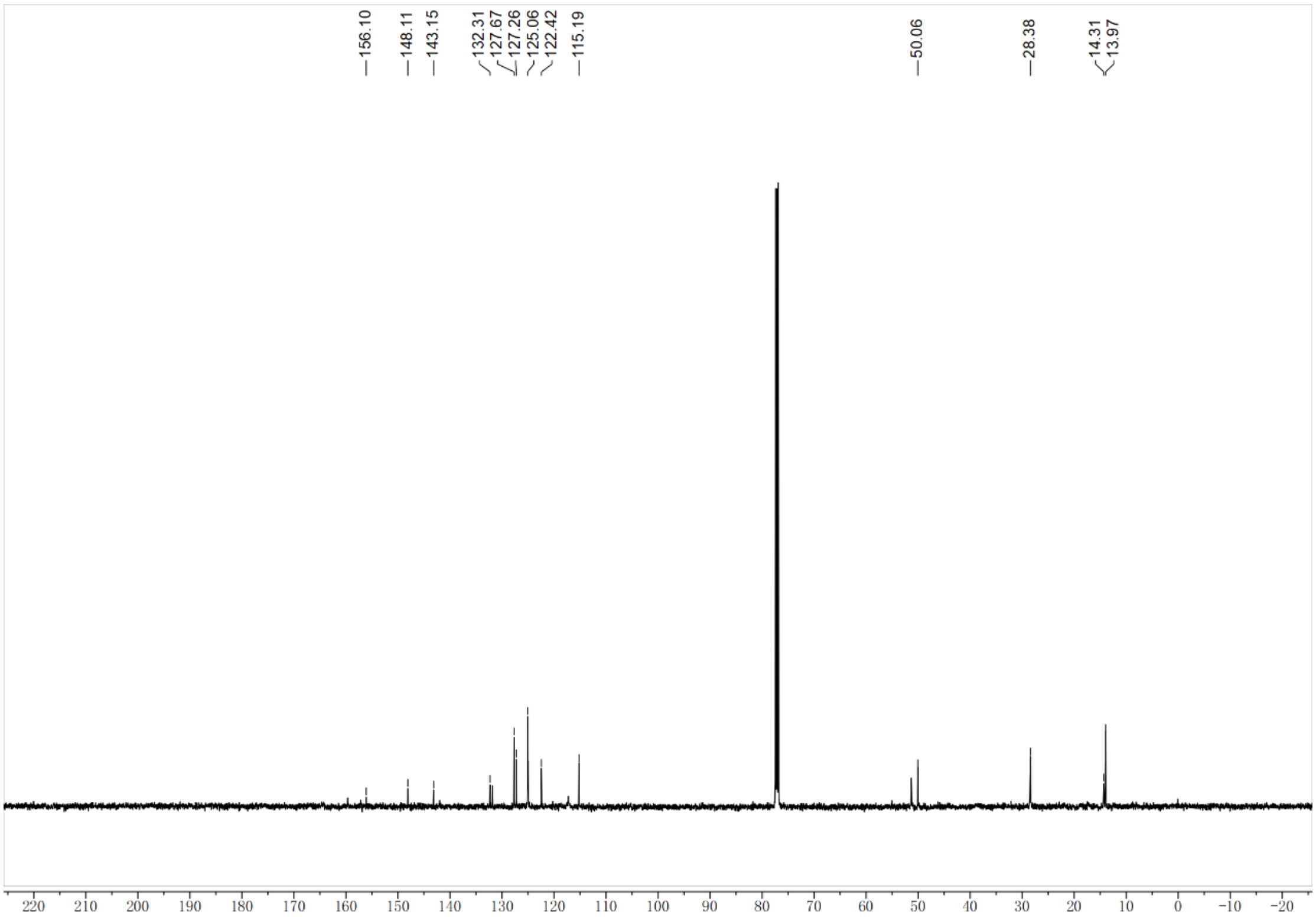

^{13}C NMR spectrum of compound DK5

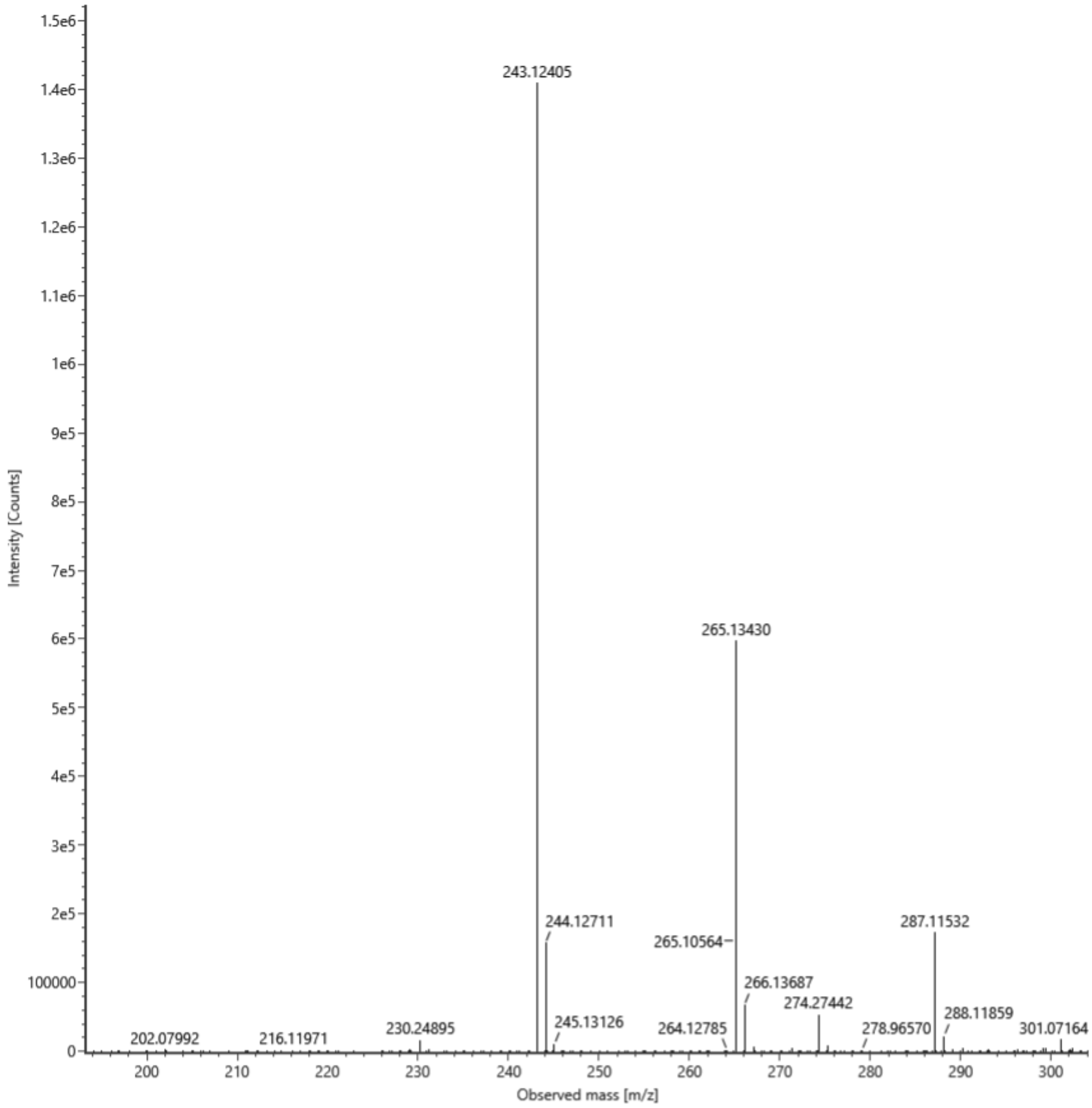

HRMS Spectrum of compound DK5

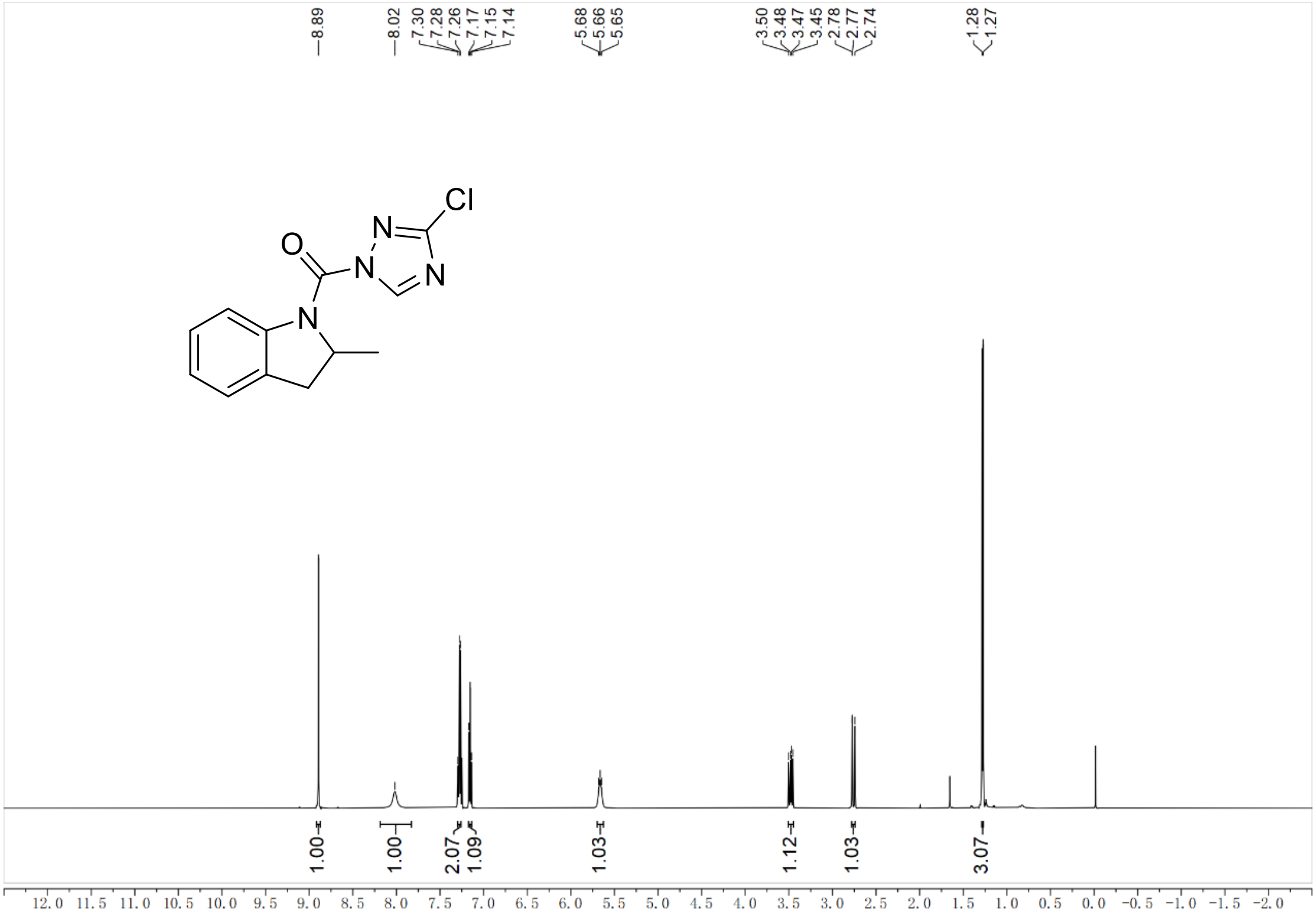

^{1}H NMR spectrum of compound DK6

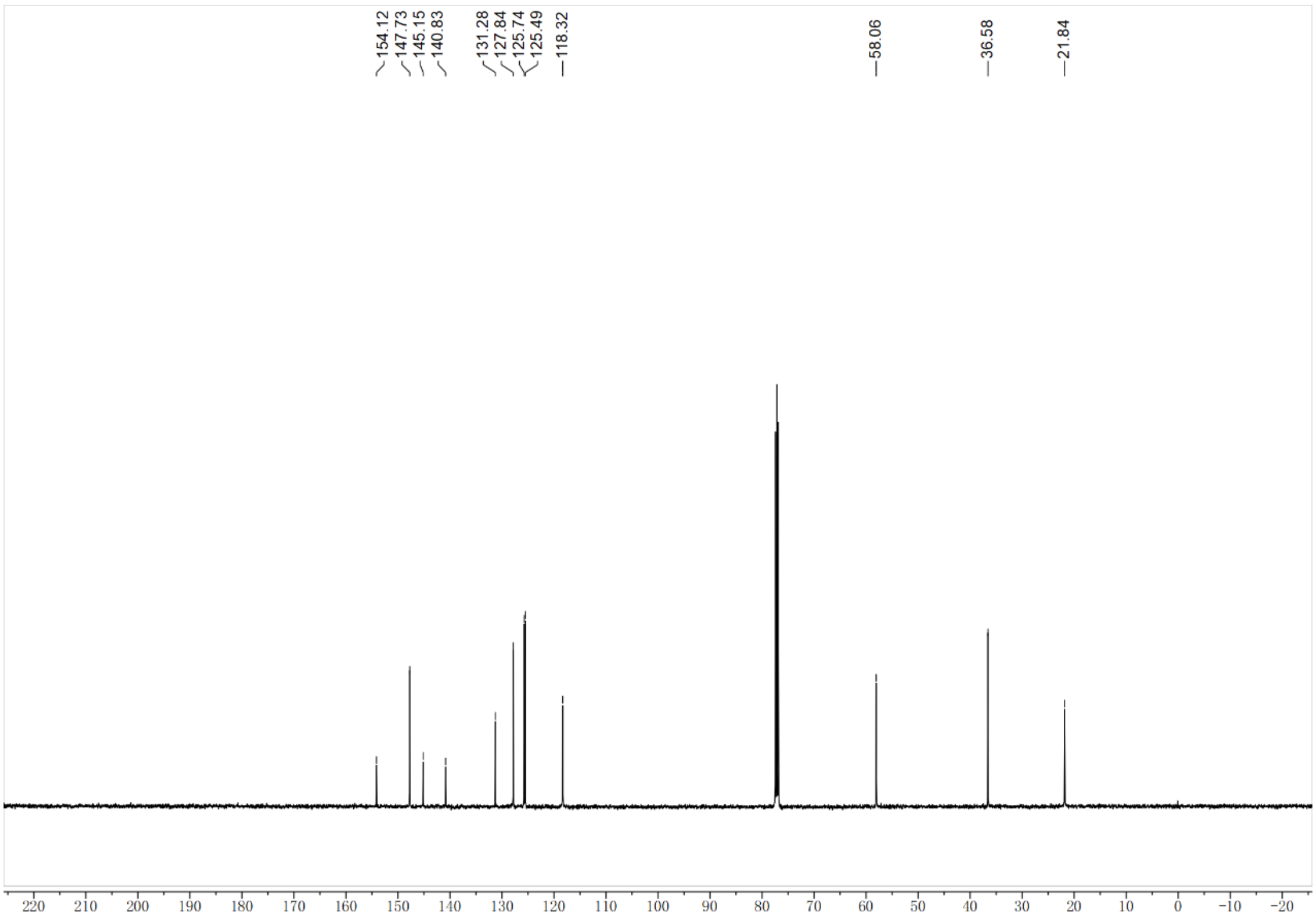

^{13}C NMR spectrum of compound DK6

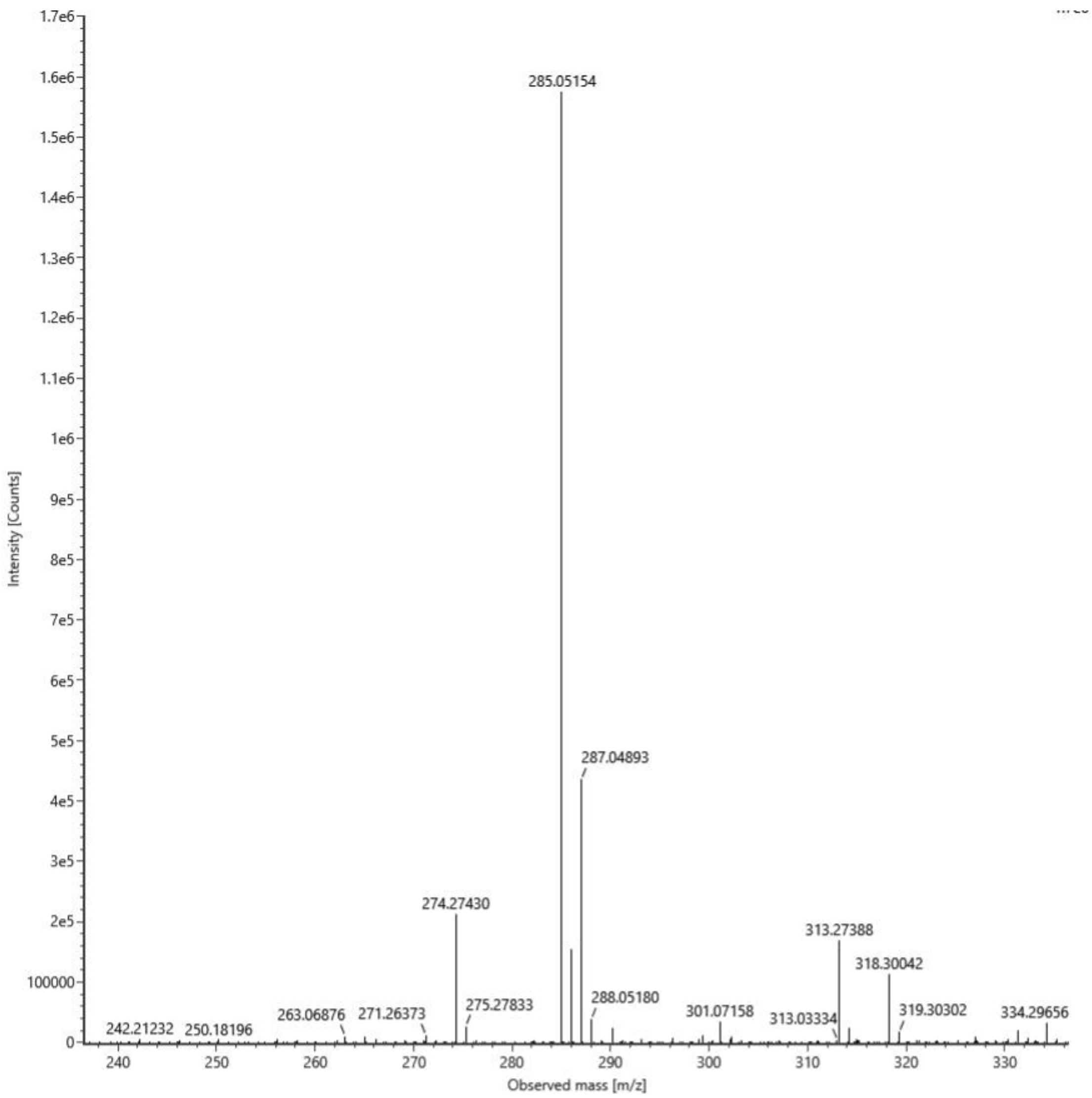

HRMS Spectrum of compound DK6

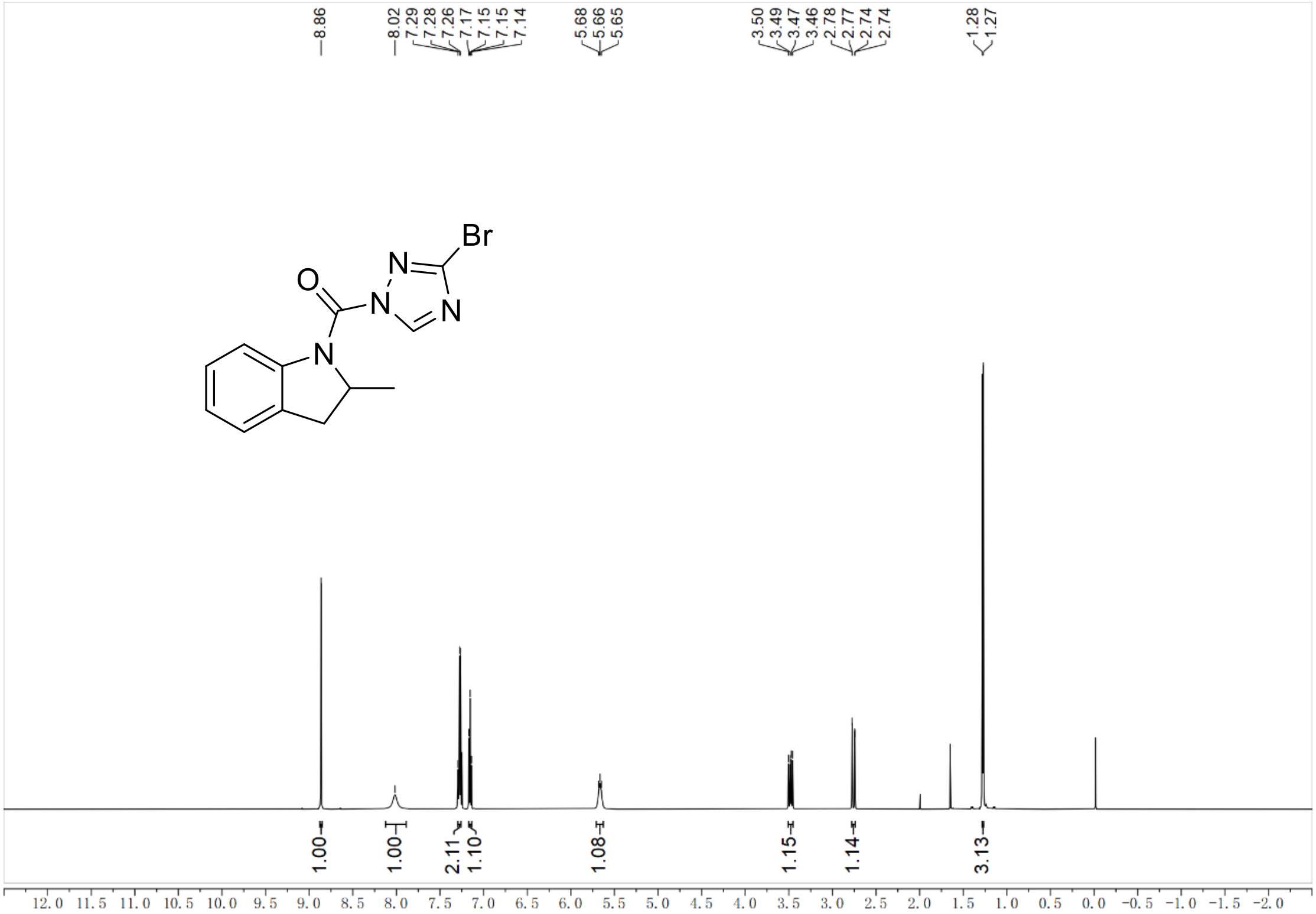

^{1}H NMR spectrum of compound DK7

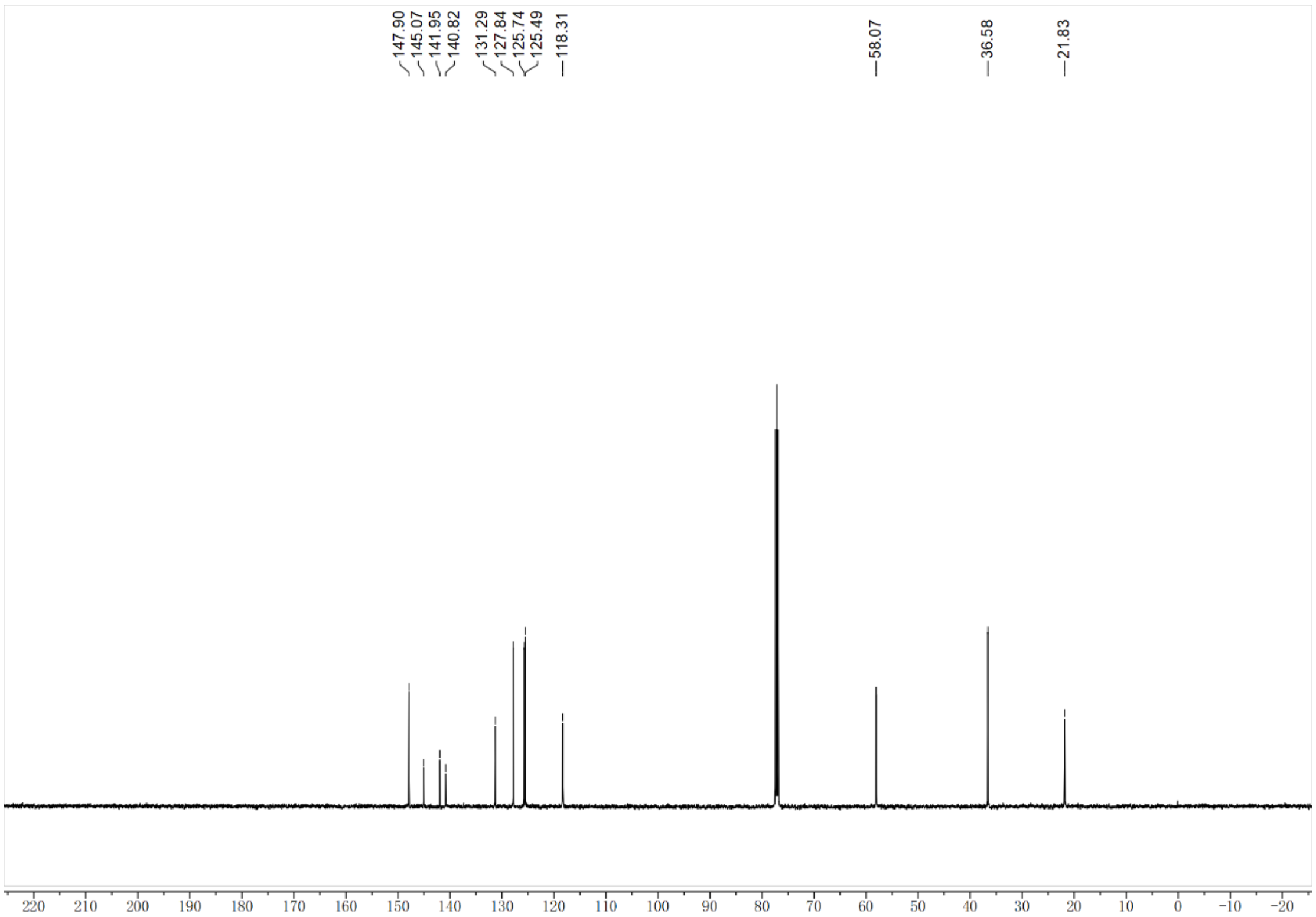

^{13}C NMR spectrum of compound DK7

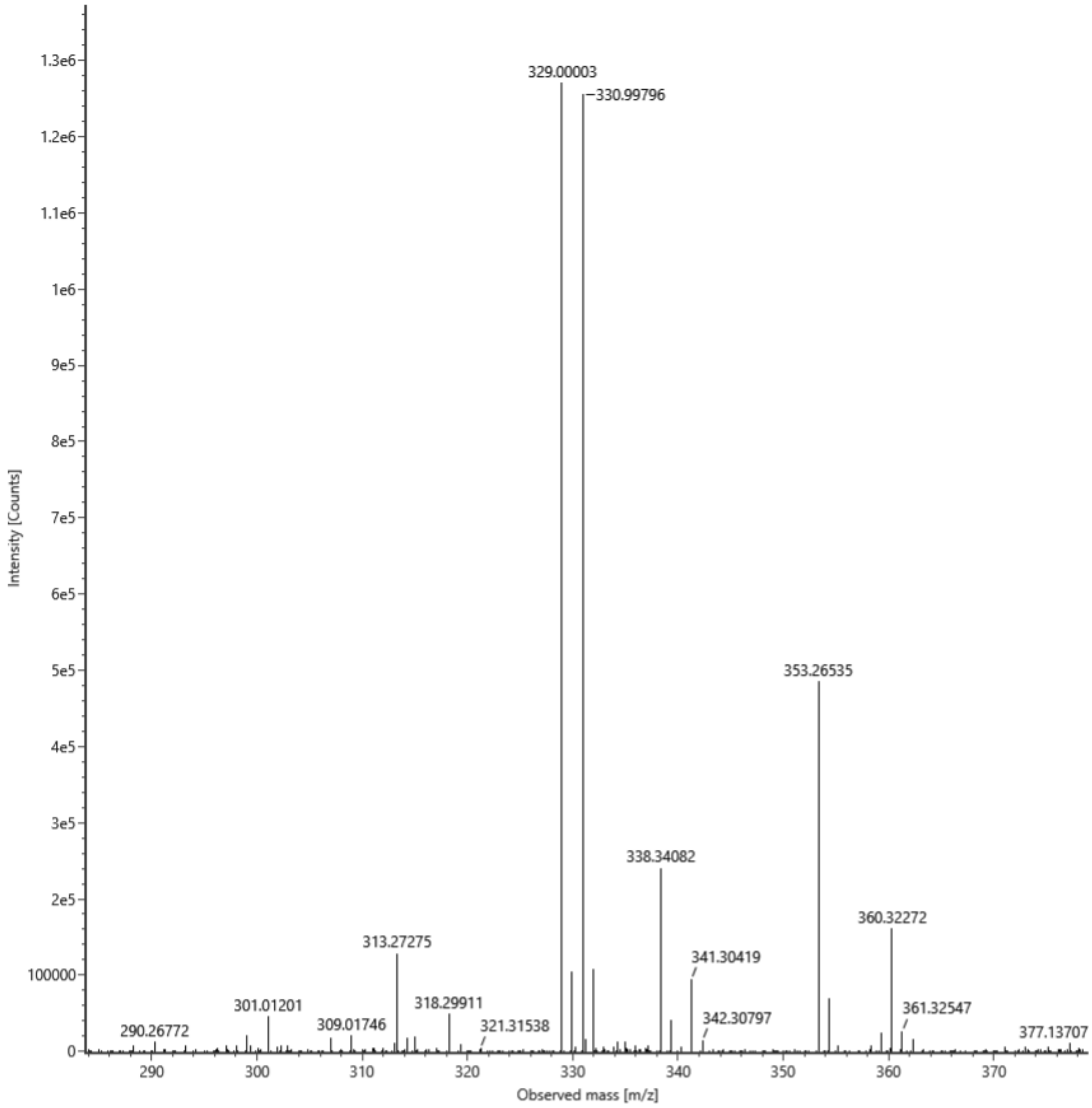

HRMS Spectrum of compound DK7

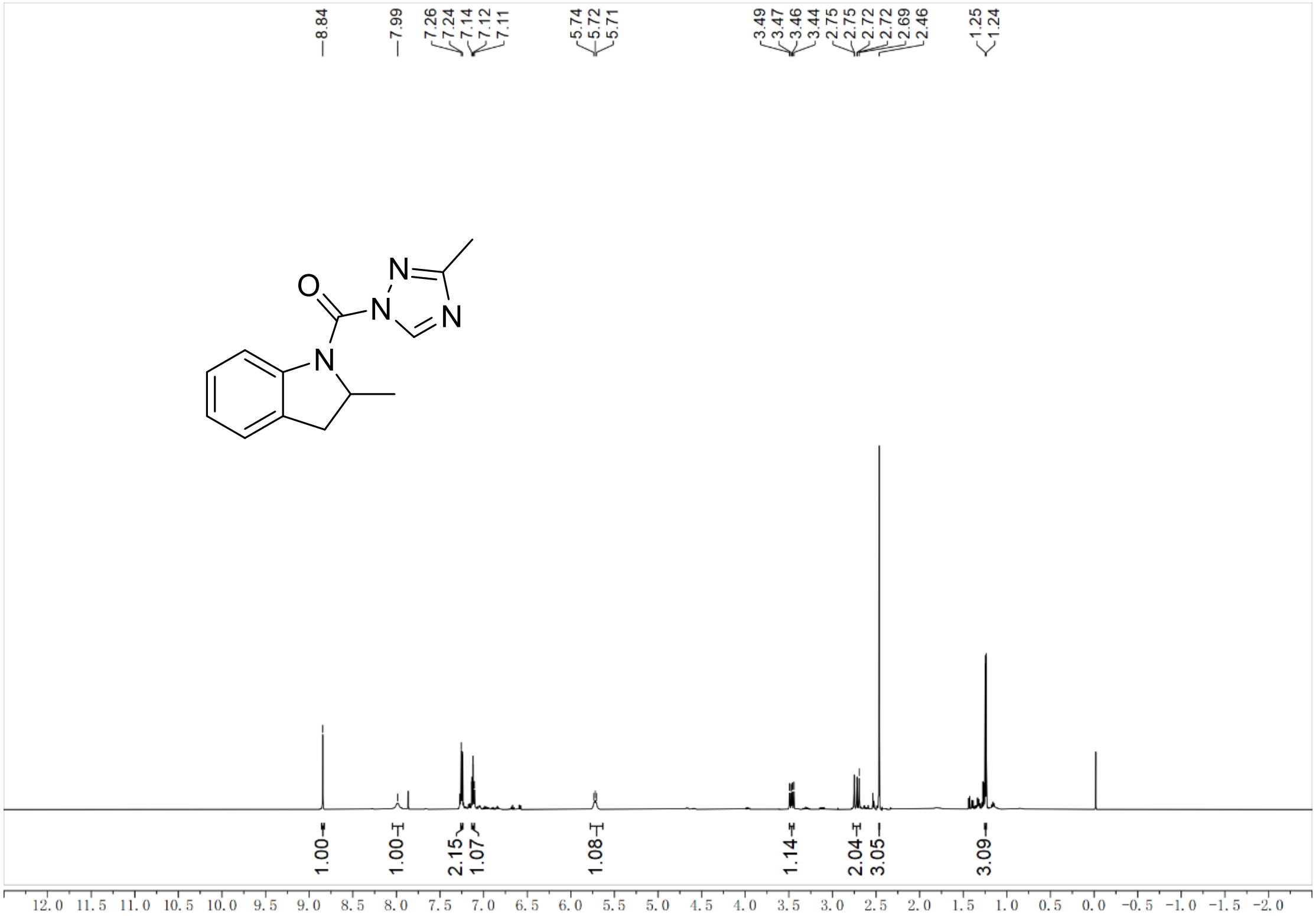

^{1}H NMR spectrum of compound DK8

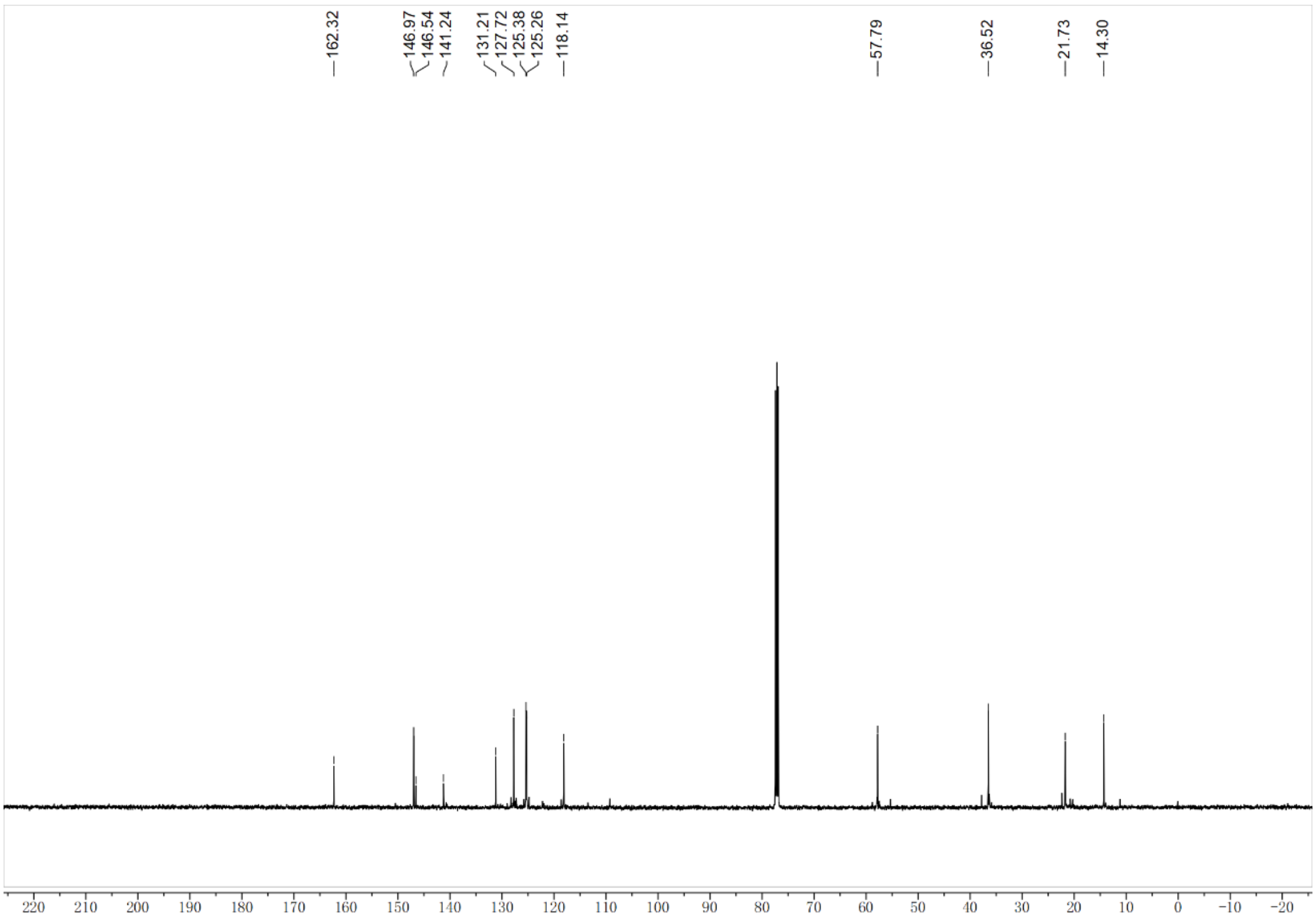

^{13}C NMR spectrum of compound DK8

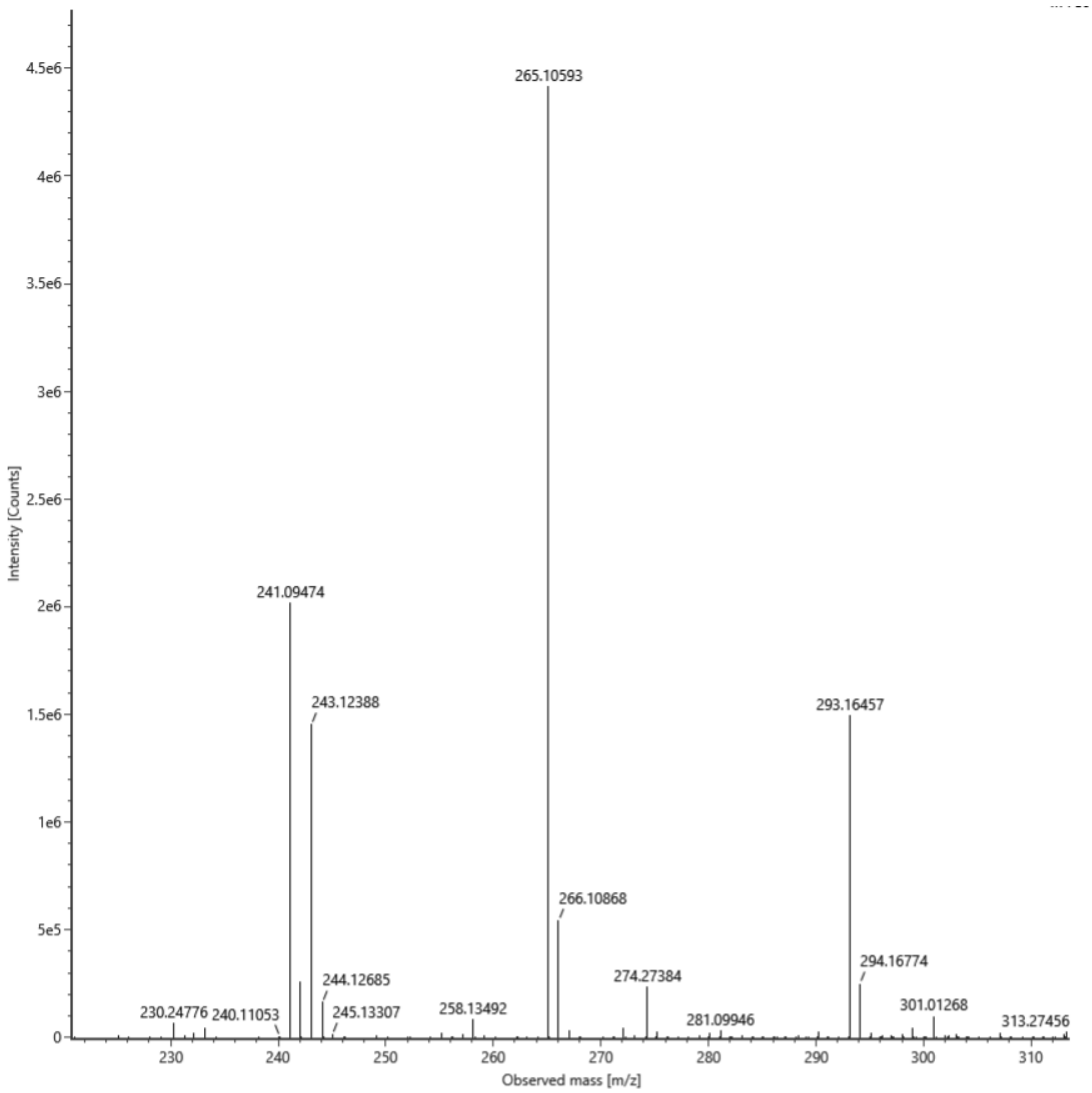

HRMS Spectrum of compound DK8

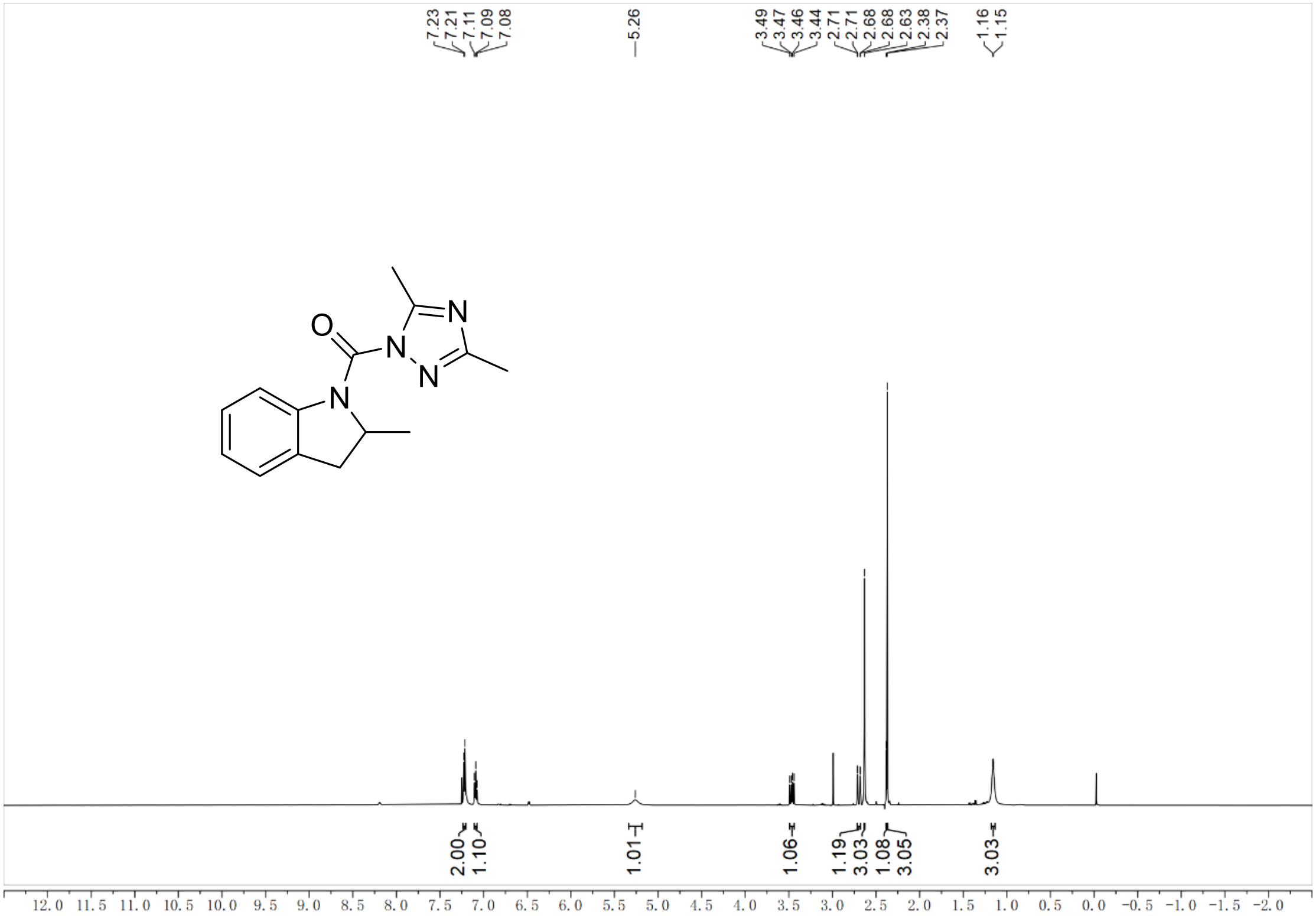

^{1}H NMR spectrum of compound DK9

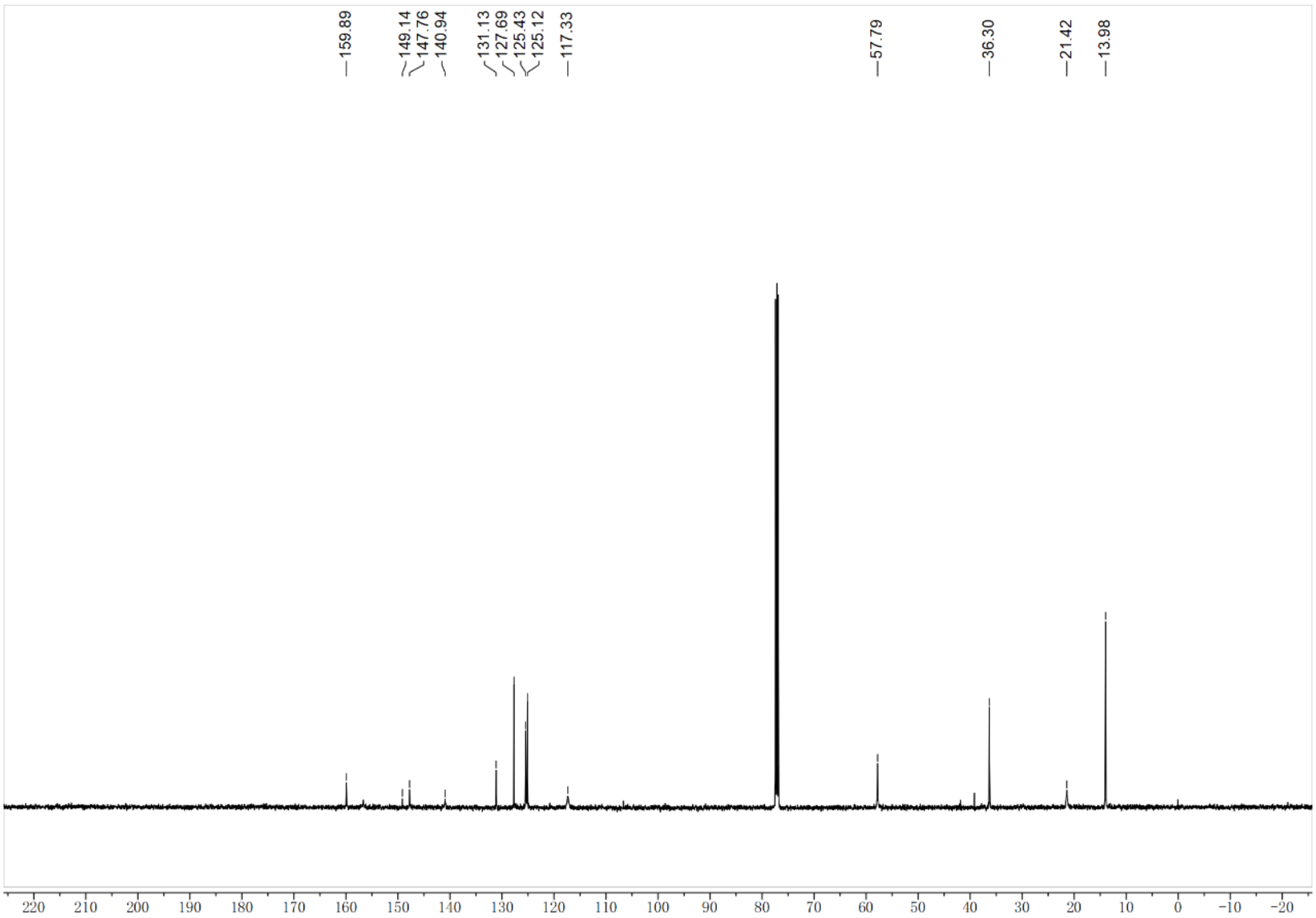

^{13}C NMR spectrum of compound DK9

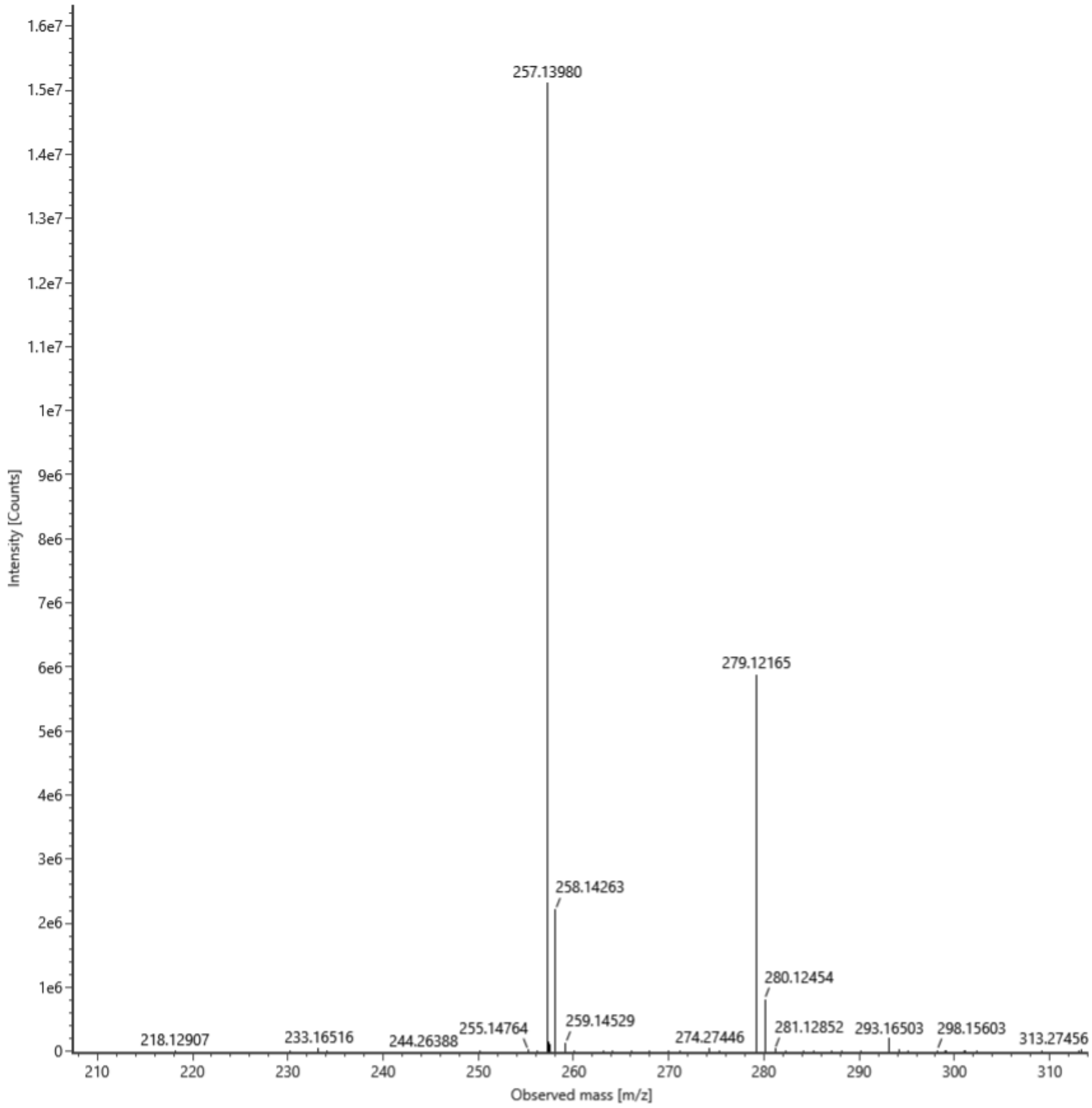

HRMS Spectrum of compound DK9

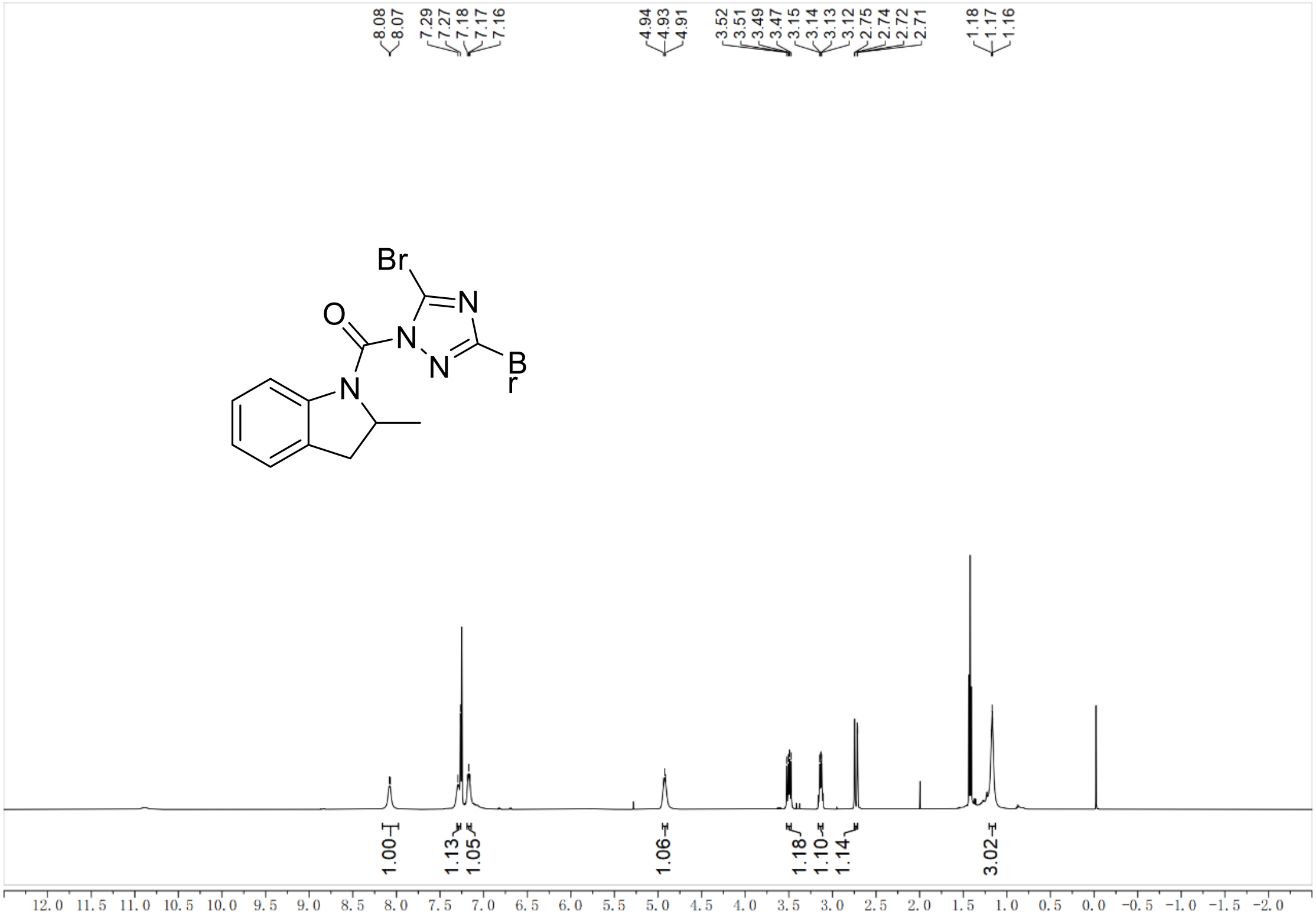

^{1}H NMR spectrum of compound DK10

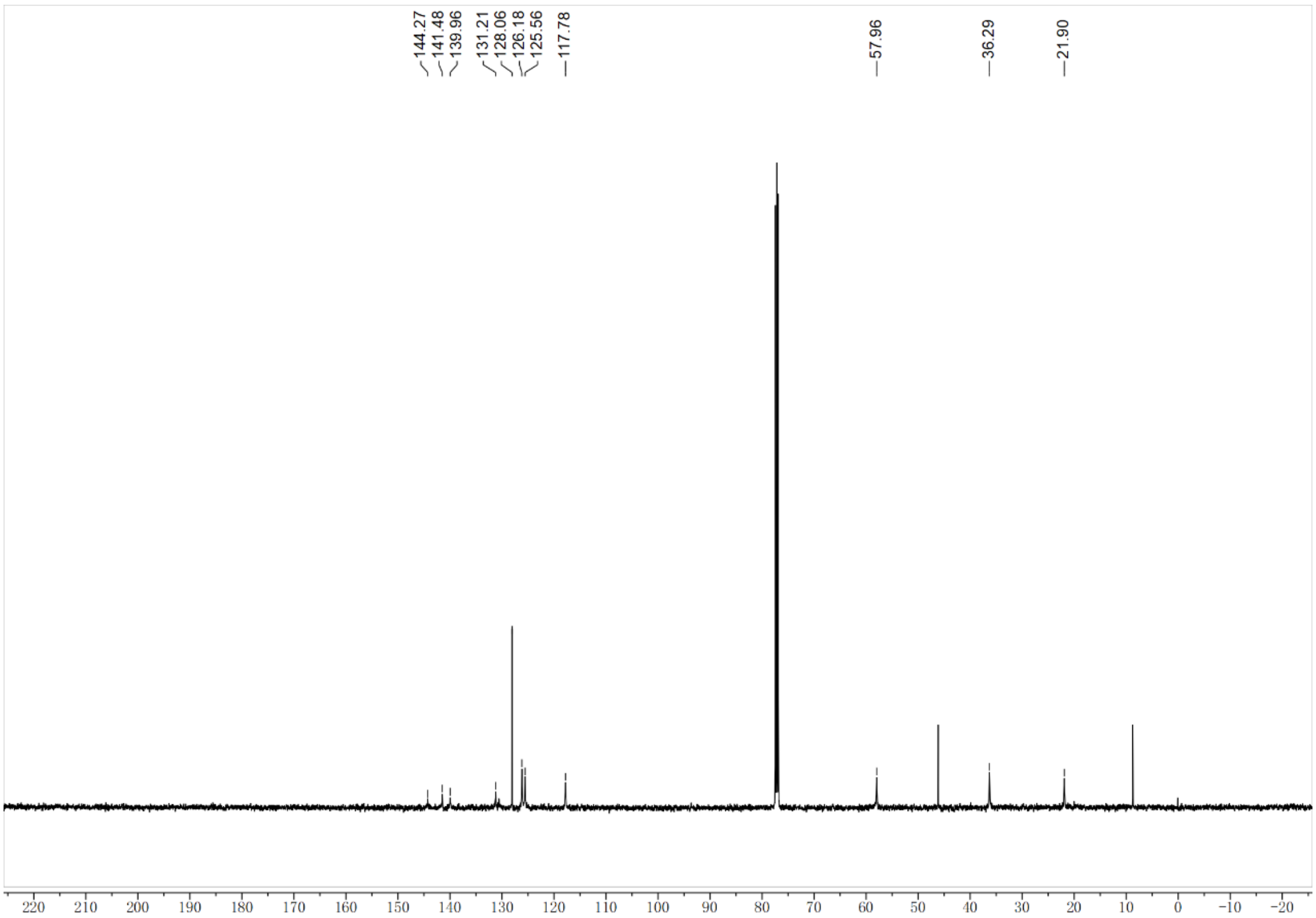

^{13}C NMR spectrum of compound DK10

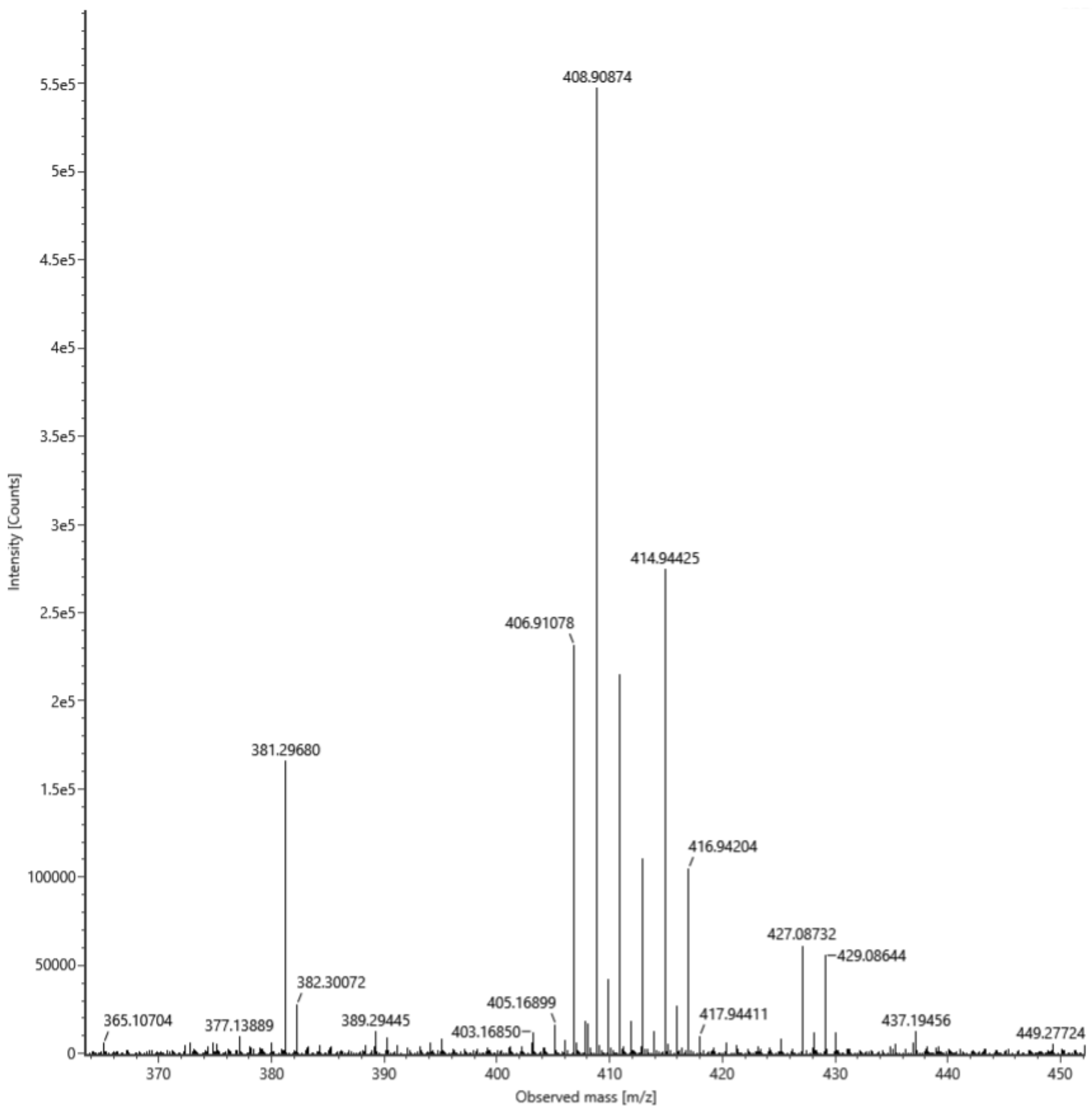

HRMS Spectrum of compound DK10

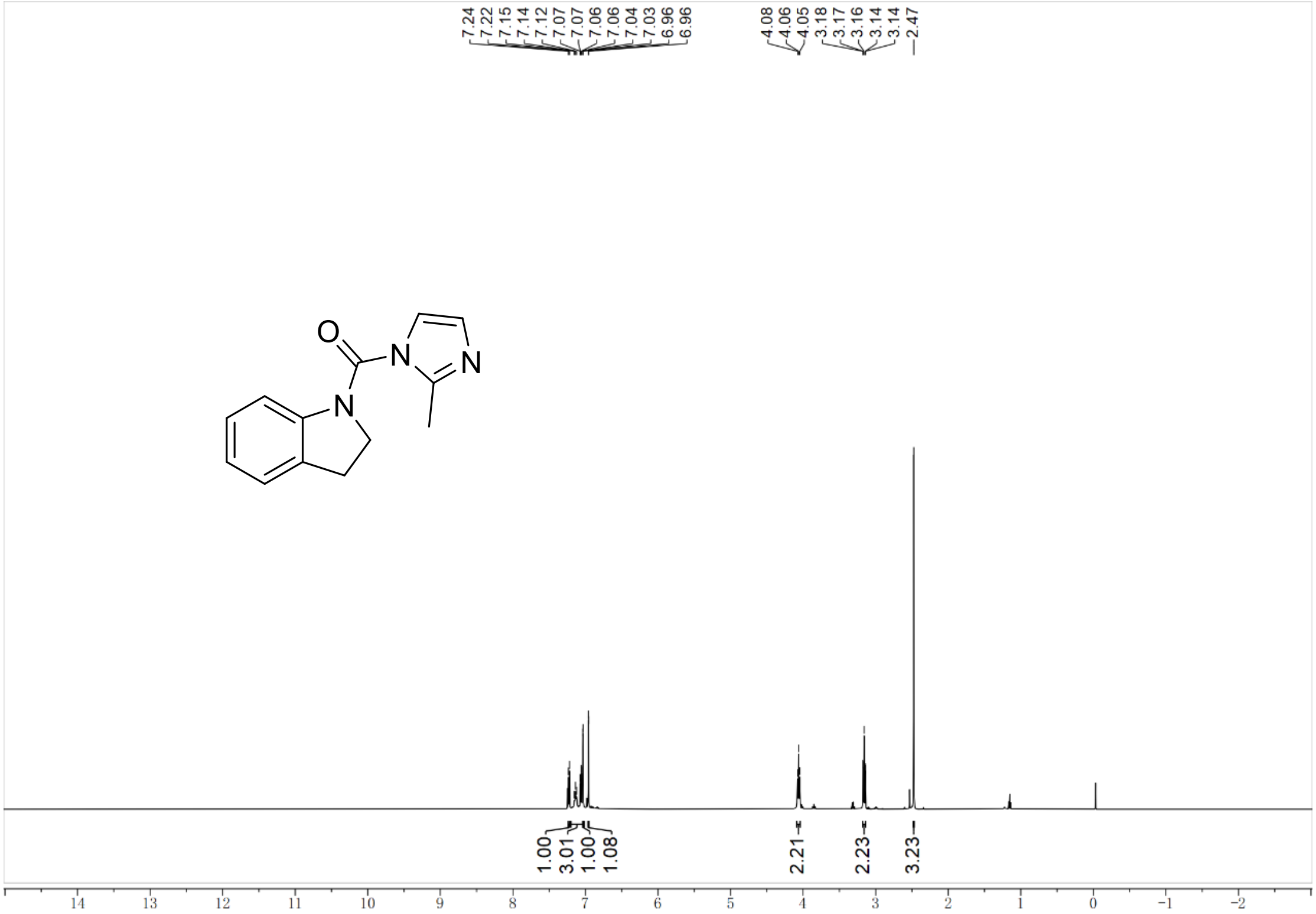

^{1}H NMR spectrum of compound DK11

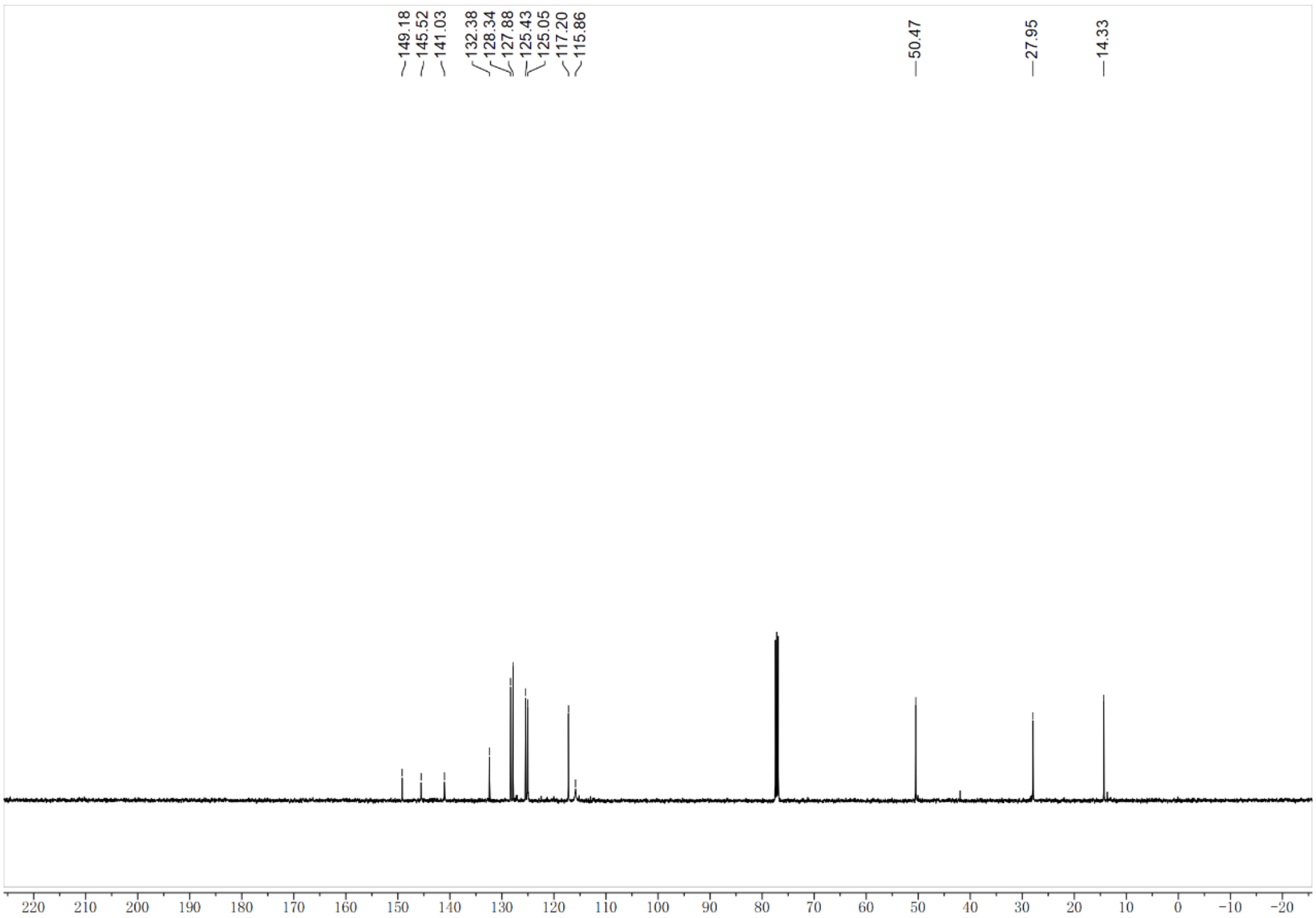

^{13}C NMR spectrum of compound DK11

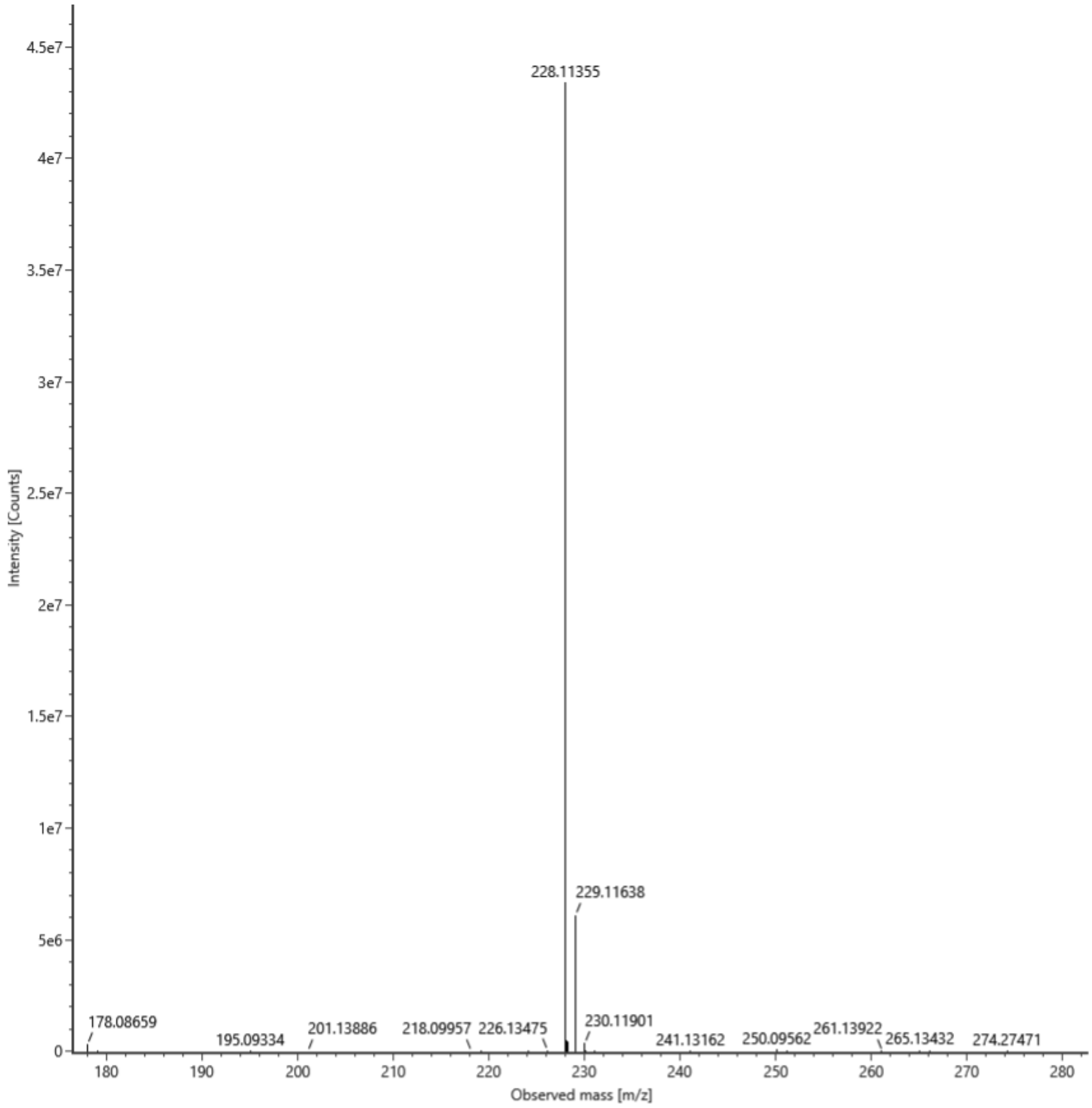

HRMS Spectrum of compound DK11

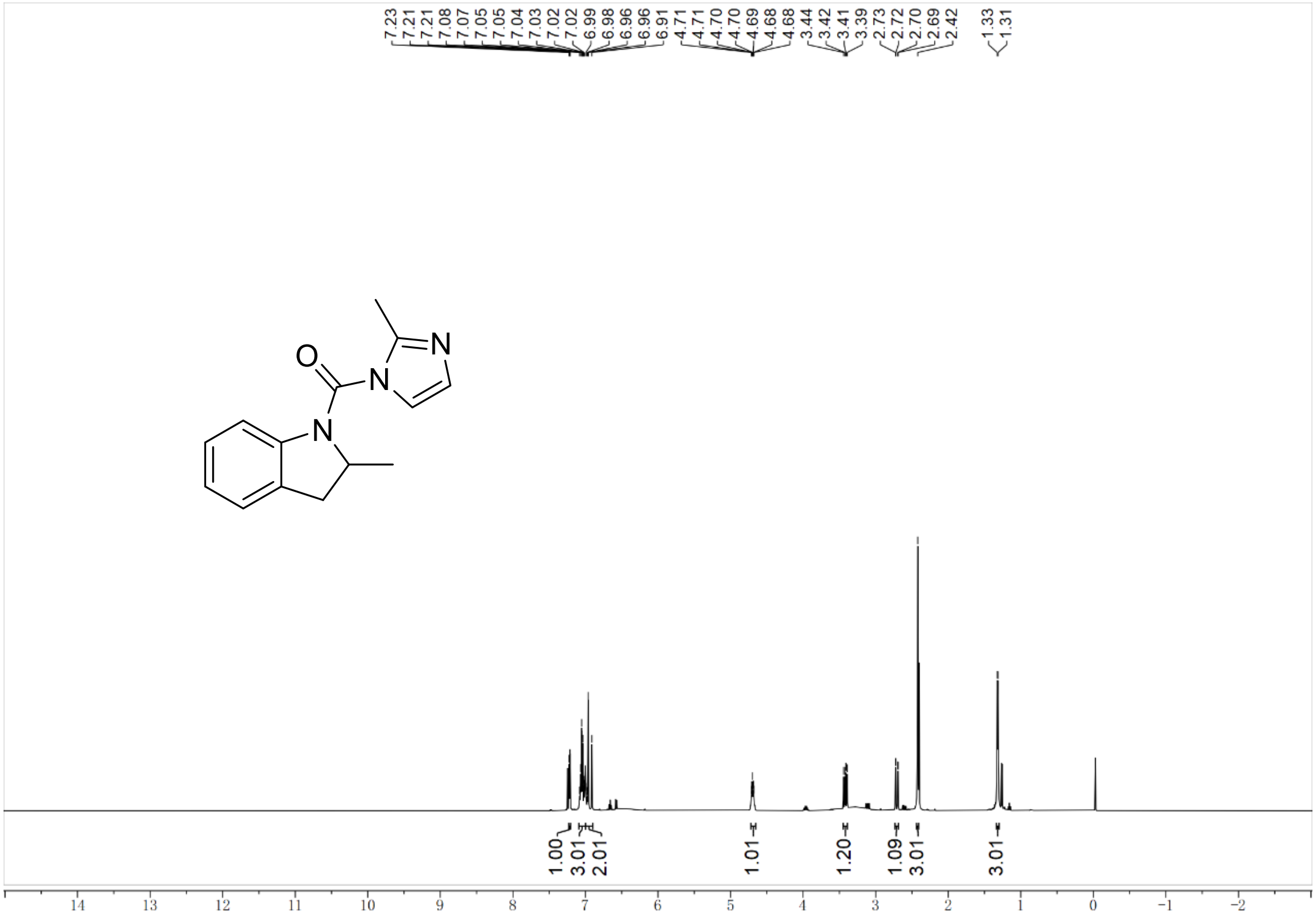

^{1}H NMR spectrum of compound DK12

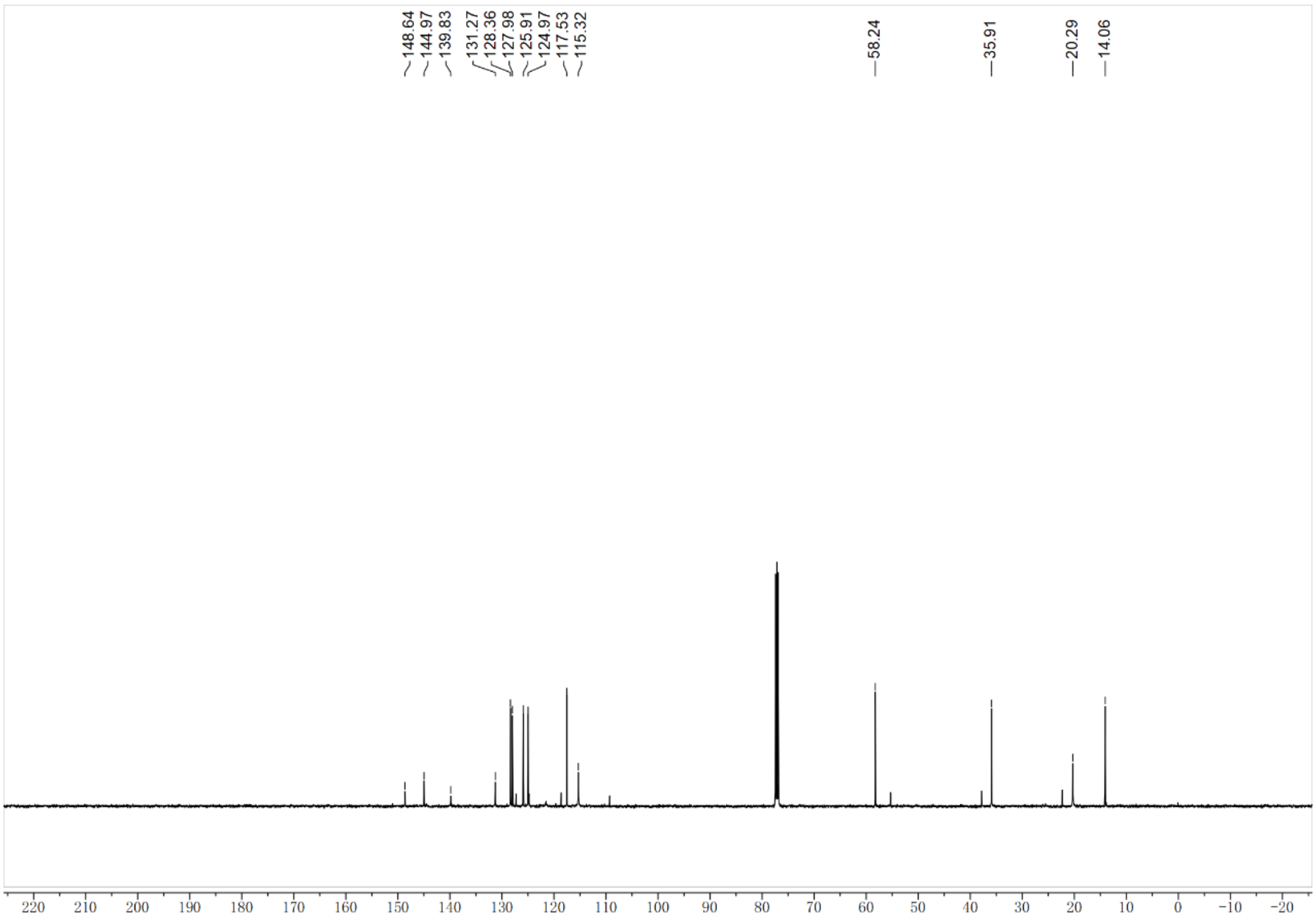

^{13}C NMR spectrum of compound DK12

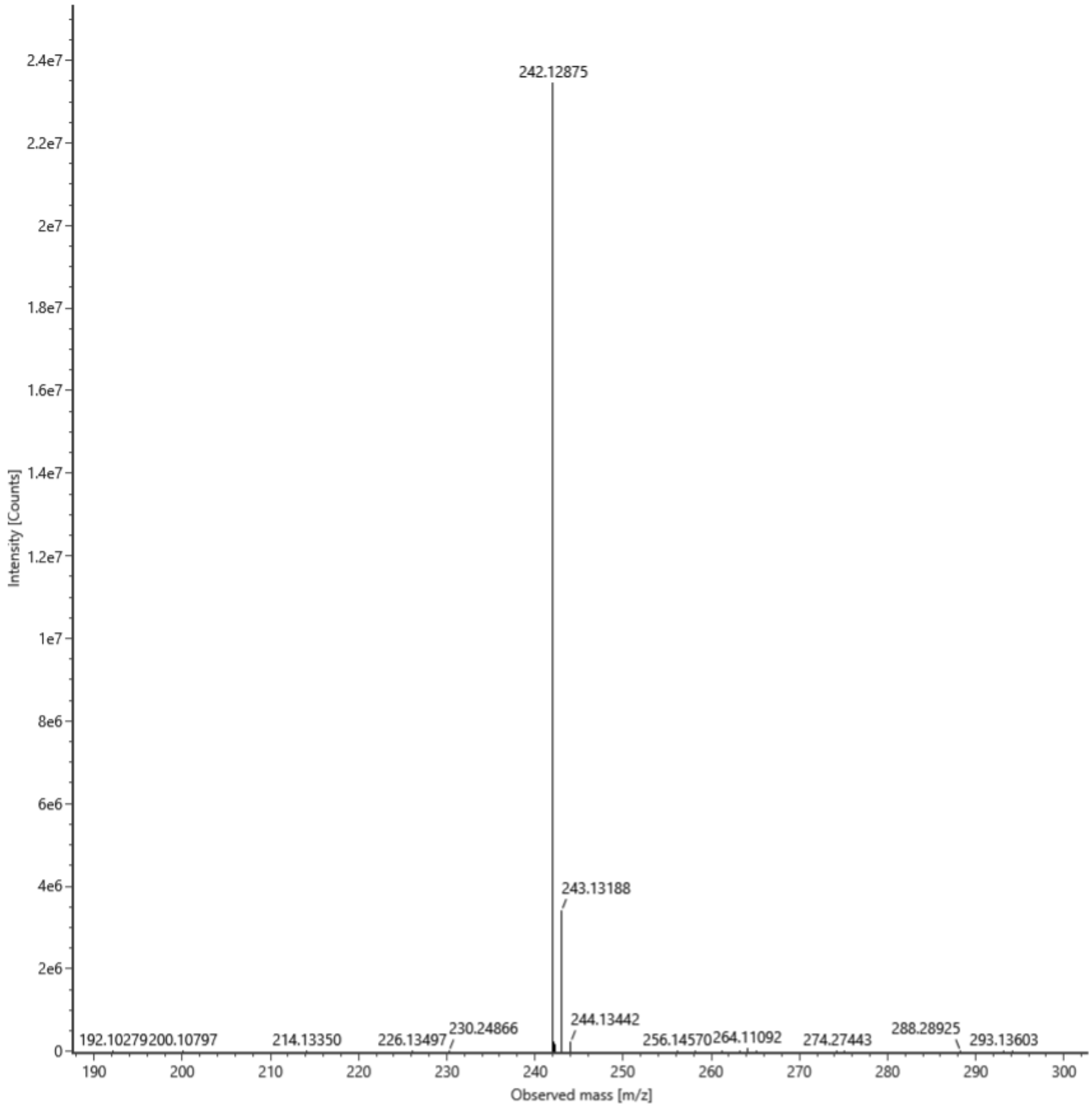

HRMS Spectrum of compound DK12

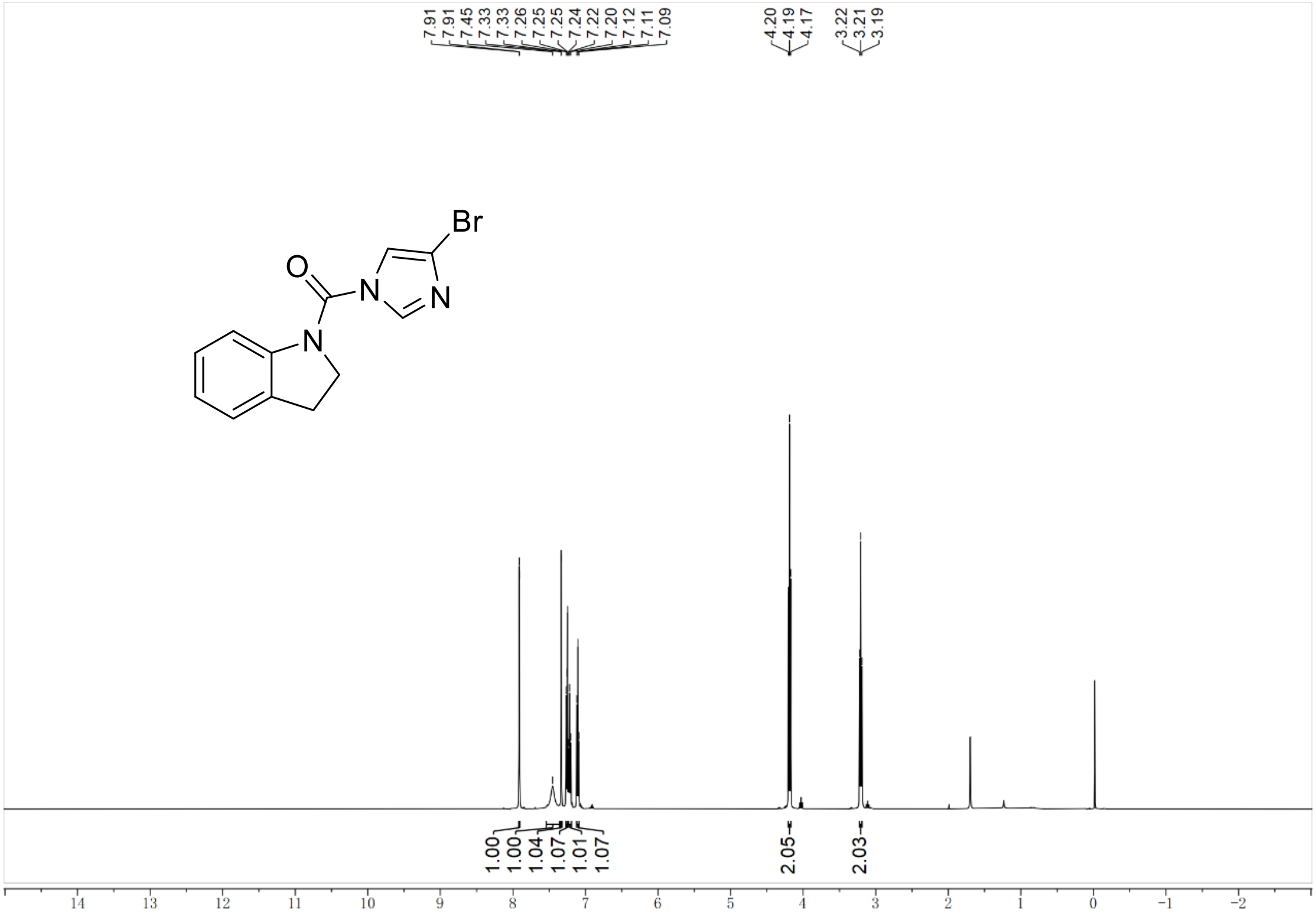

^{1}H NMR spectrum of compound DK13

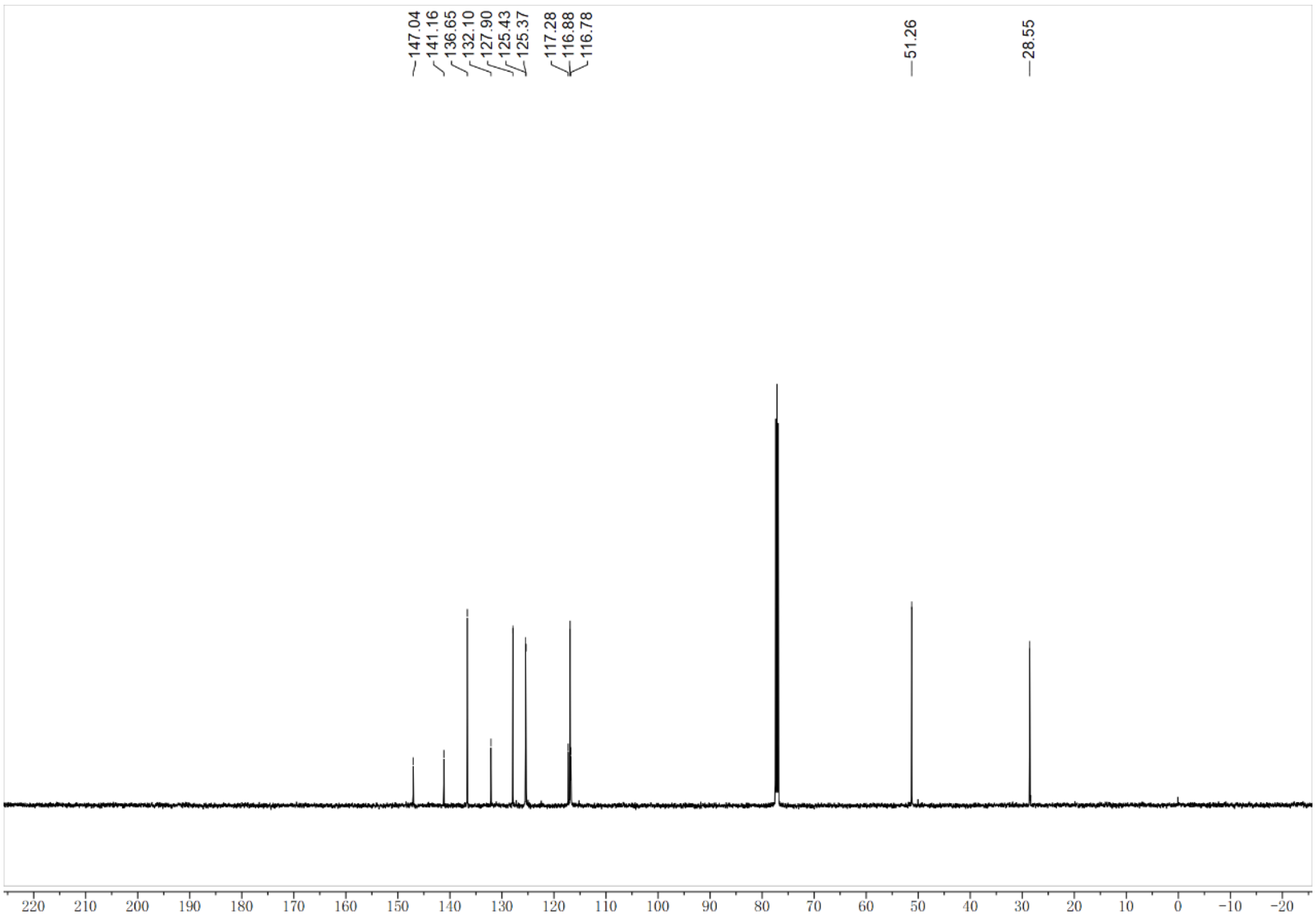

^{13}C NMR spectrum of compound DK13

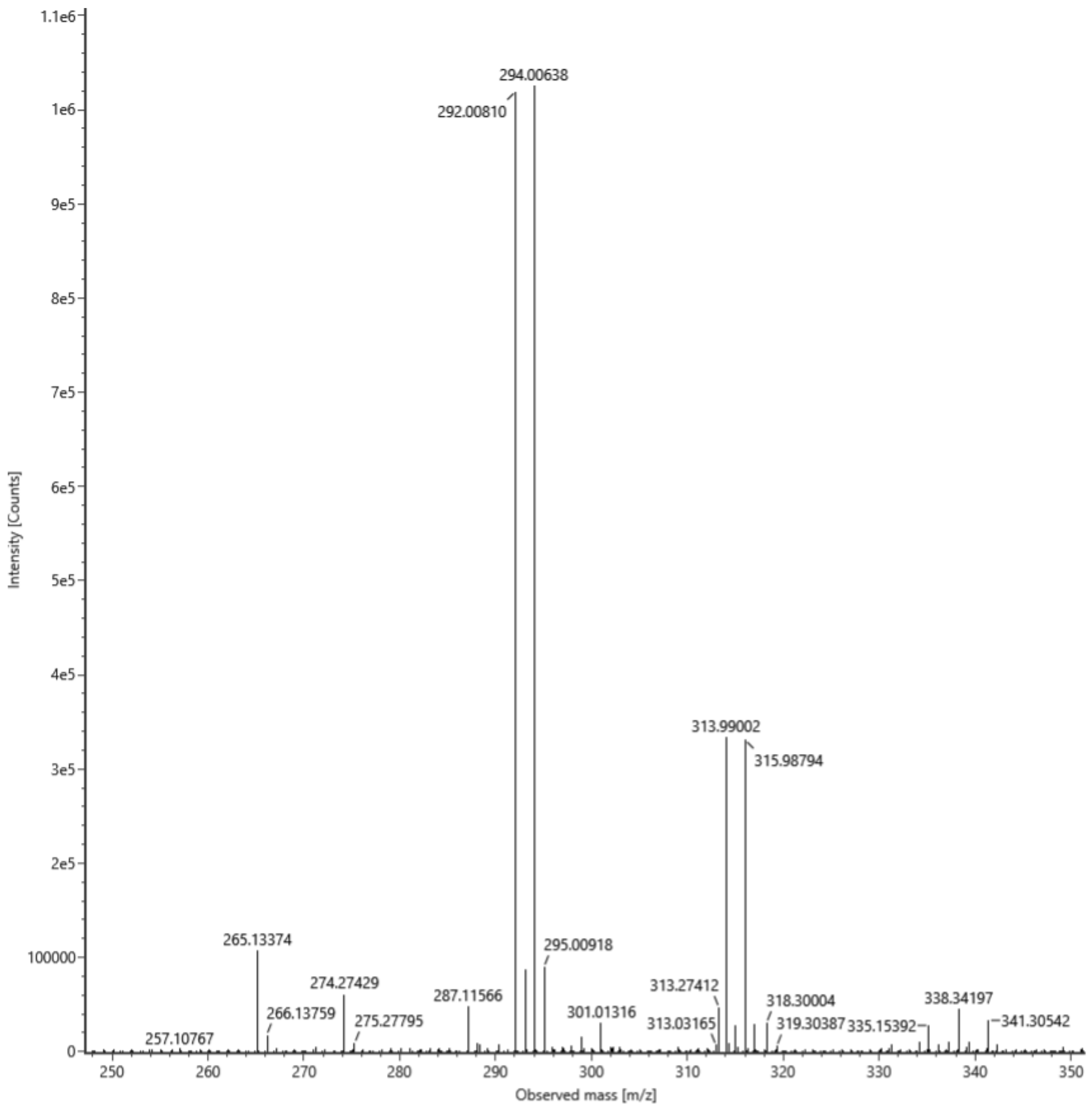

HRMS Spectrum of compound DK13

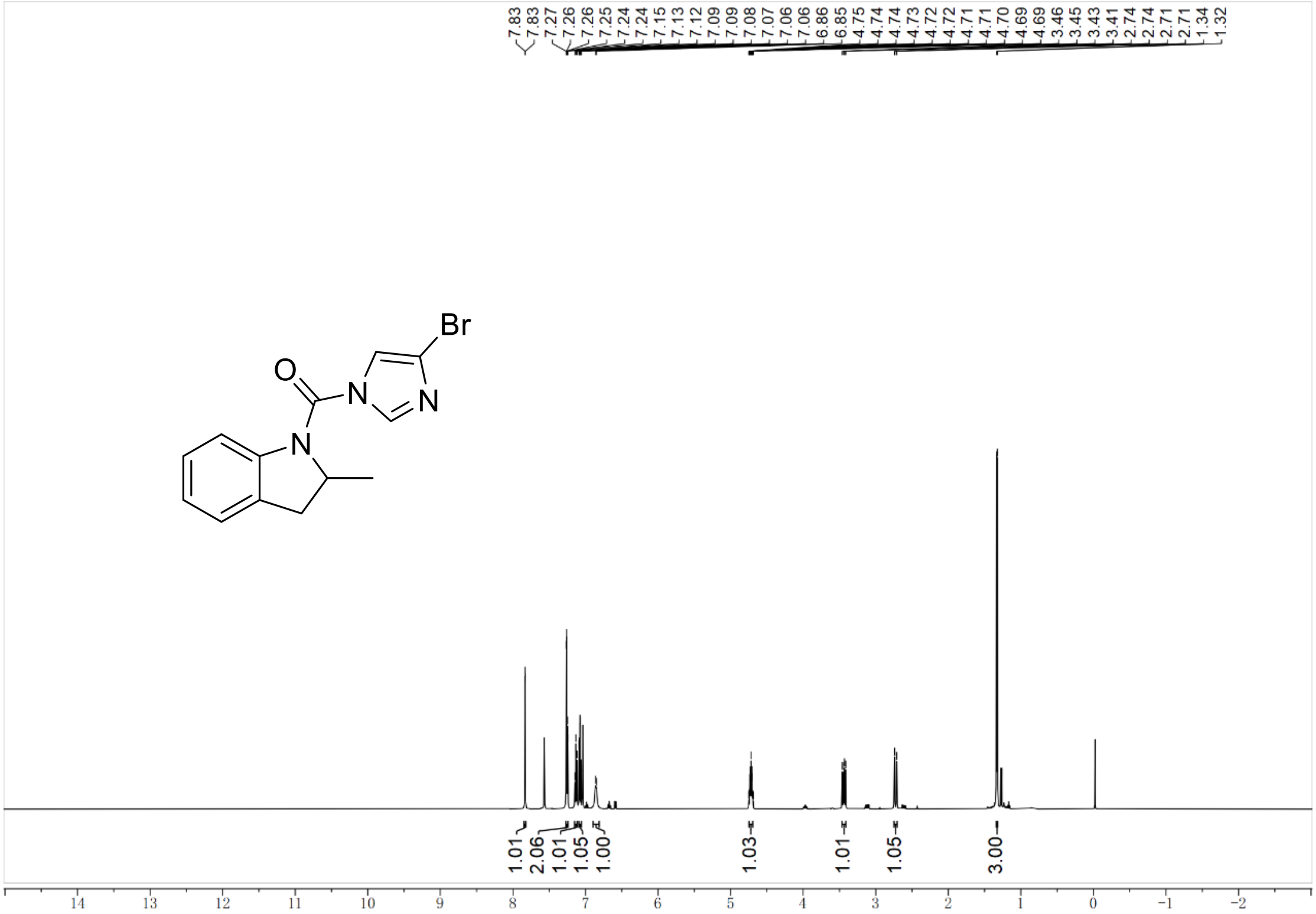

^{1}H NMR spectrum of compound DK14

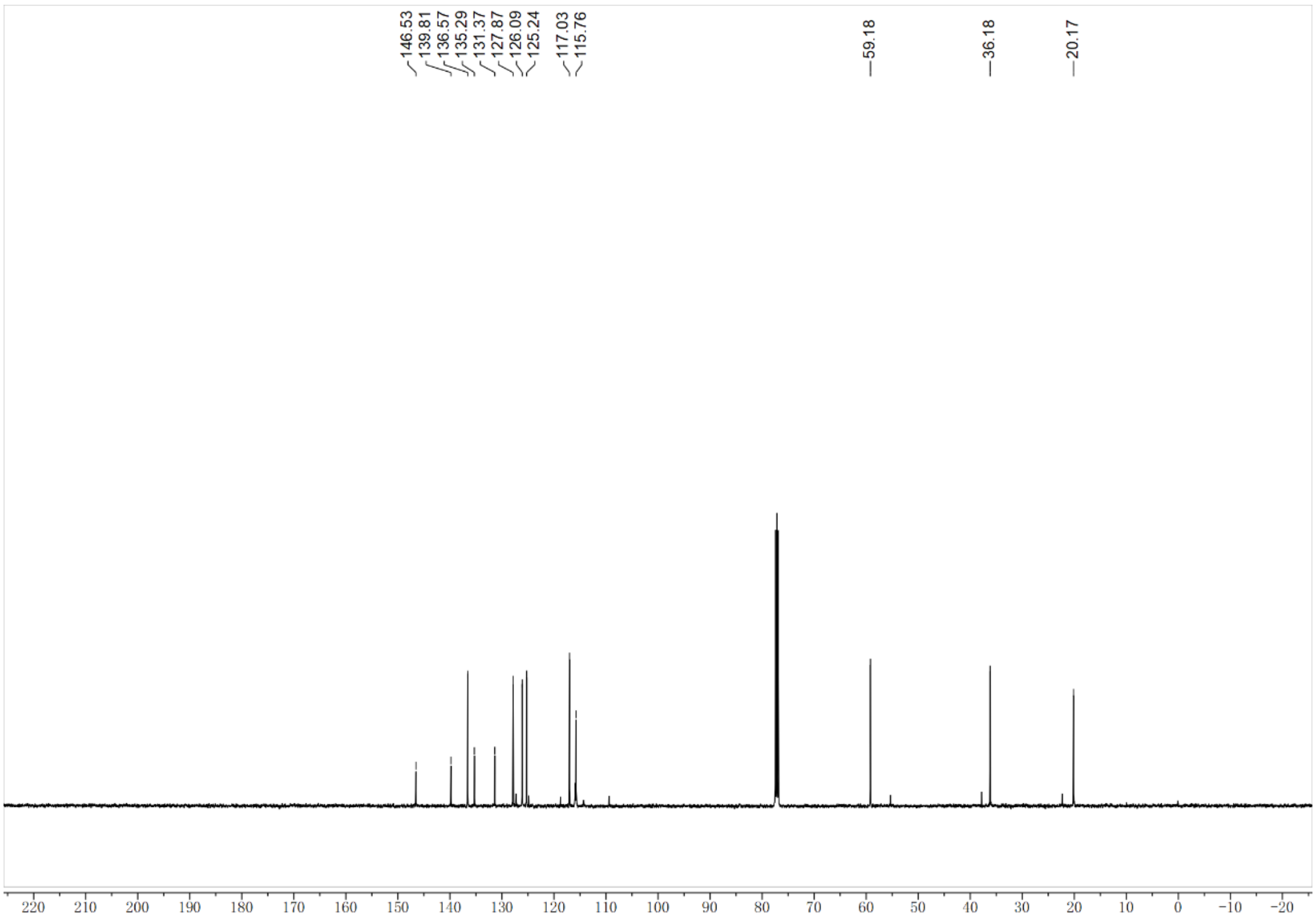

^{13}C NMR spectrum of compound DK14

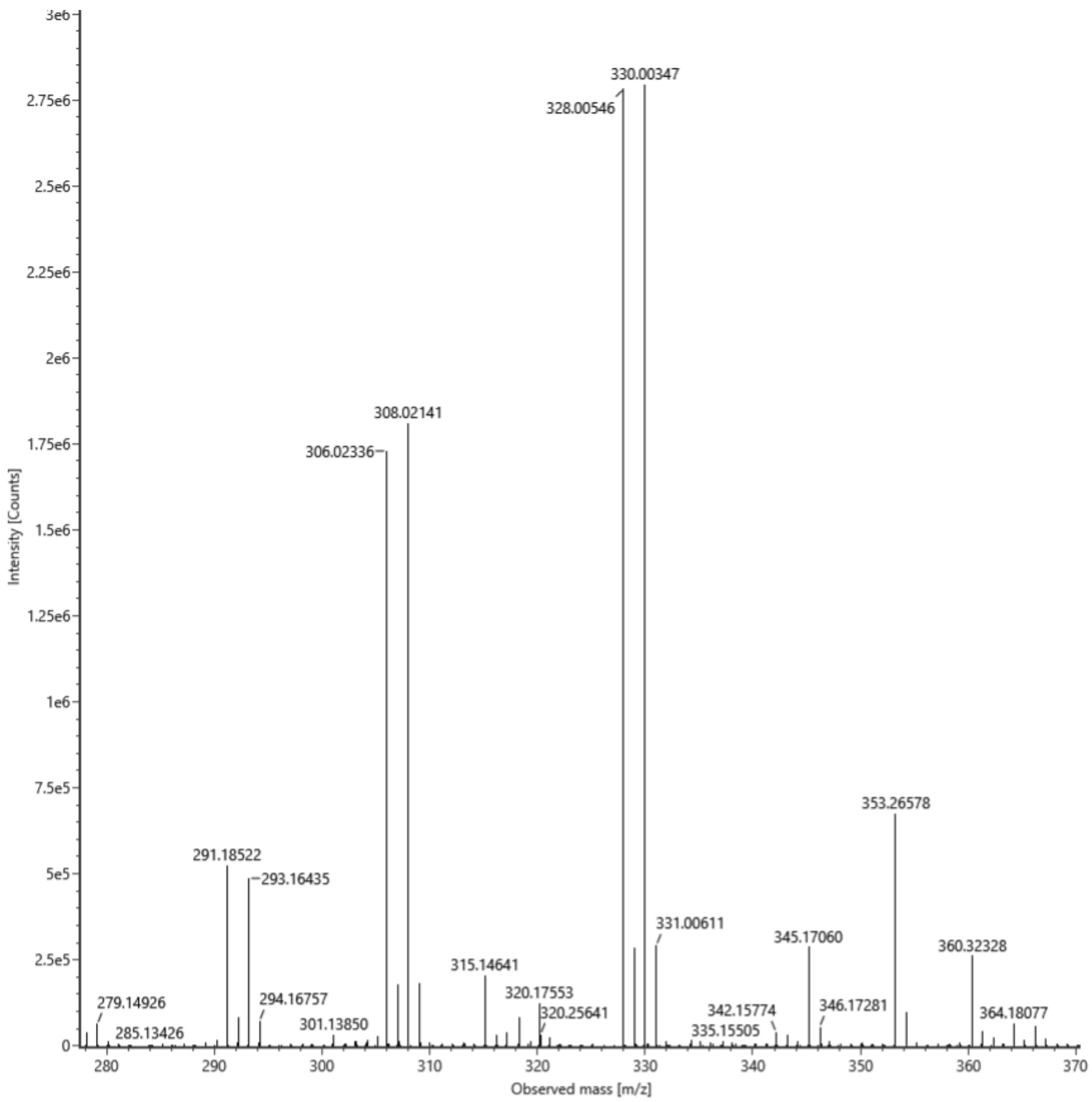

HRMS Spectrum of compound DK14

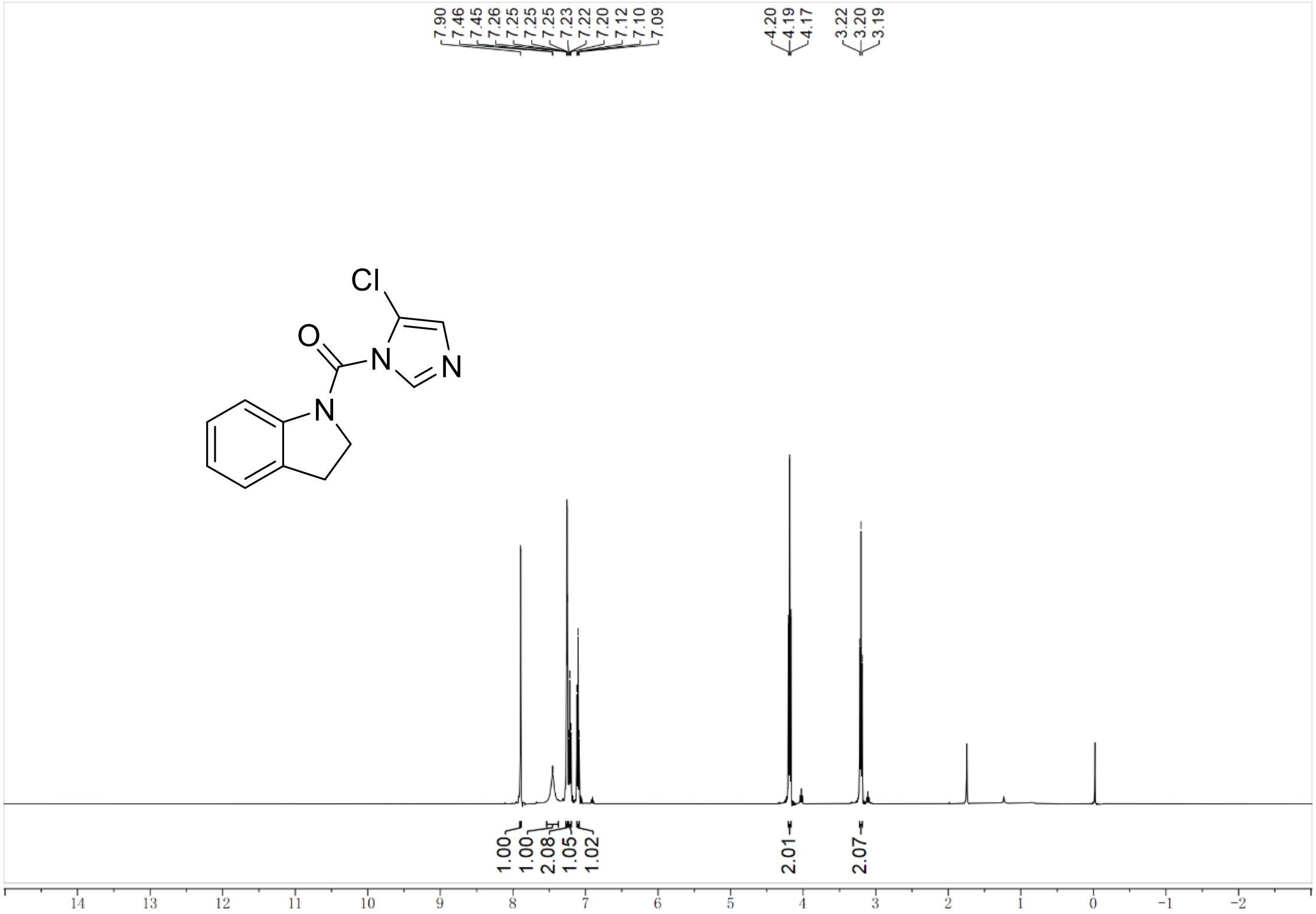

^{1}H NMR spectrum of compound DK15

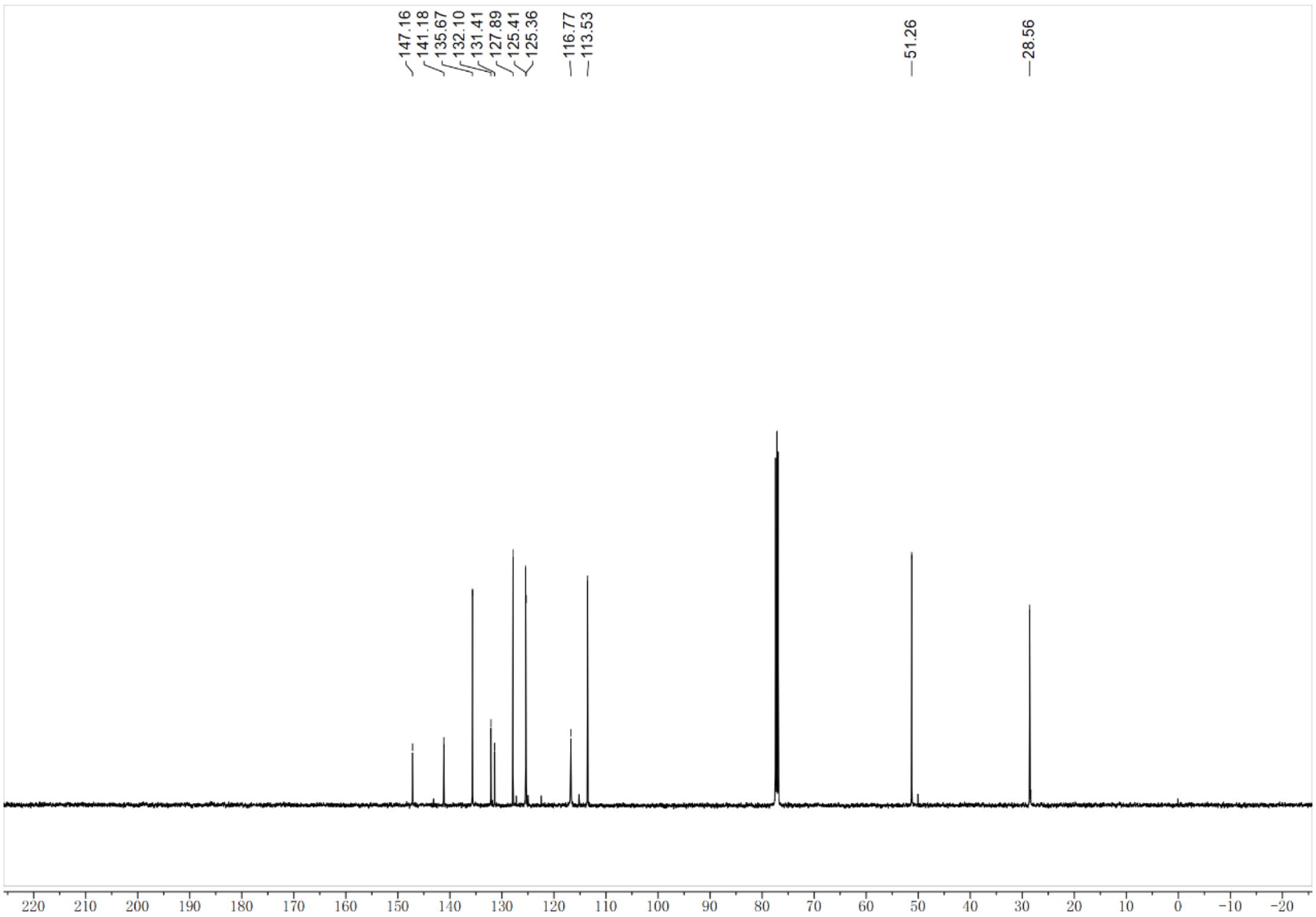

^{13}C NMR spectrum of compound DK15

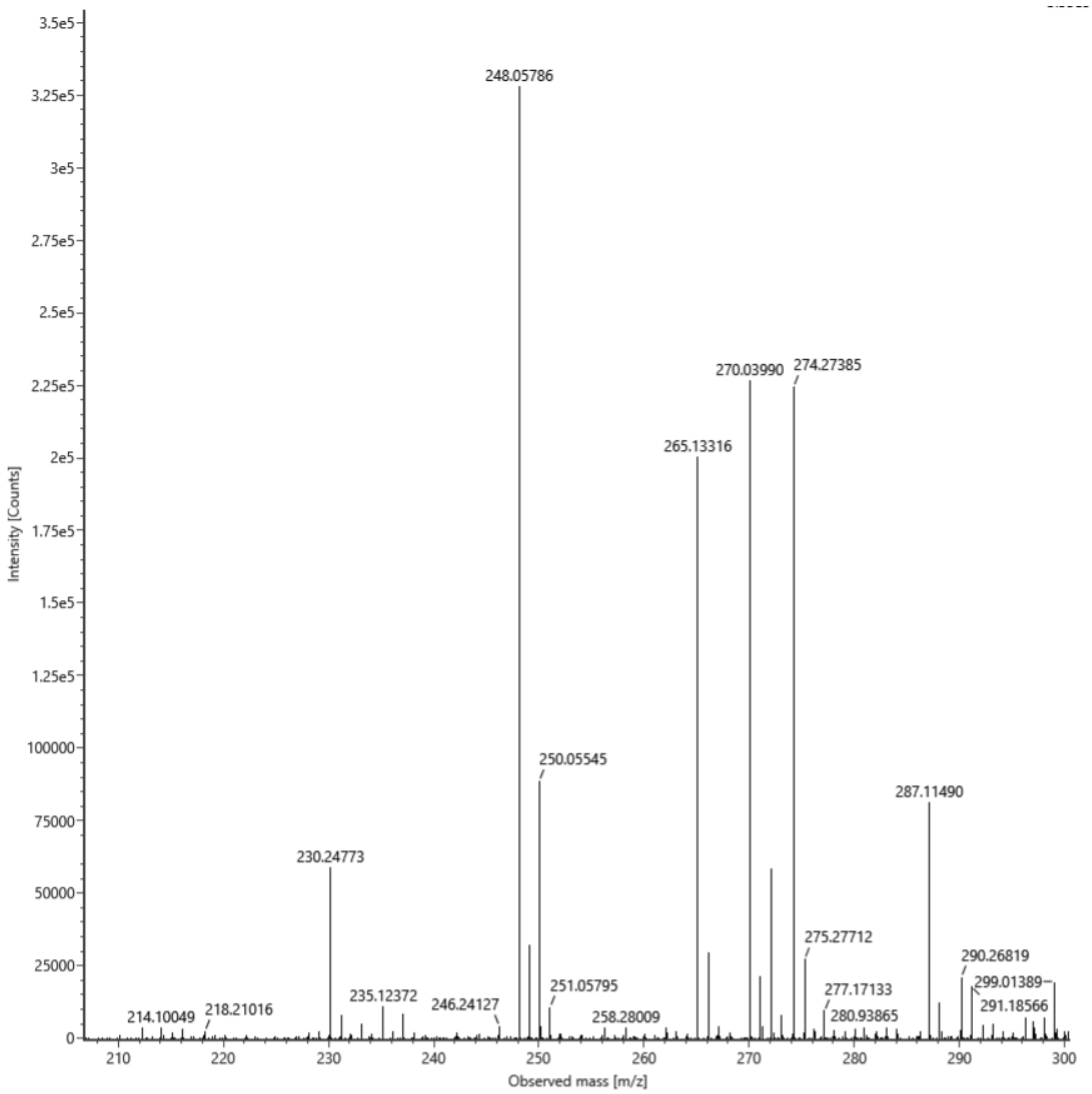

HRMS Spectrum of compound DK15

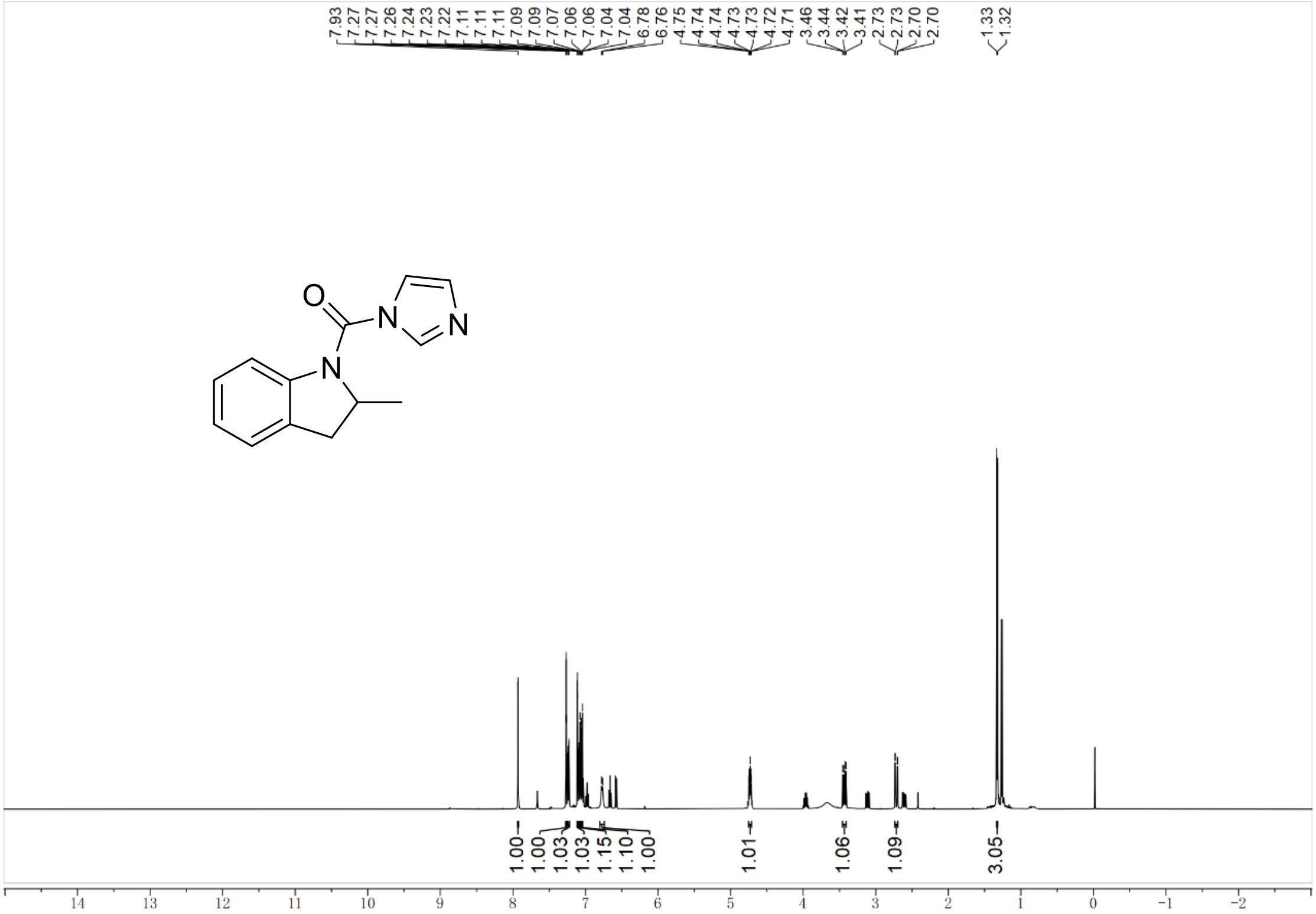

^{1}H NMR spectrum of compound DK16

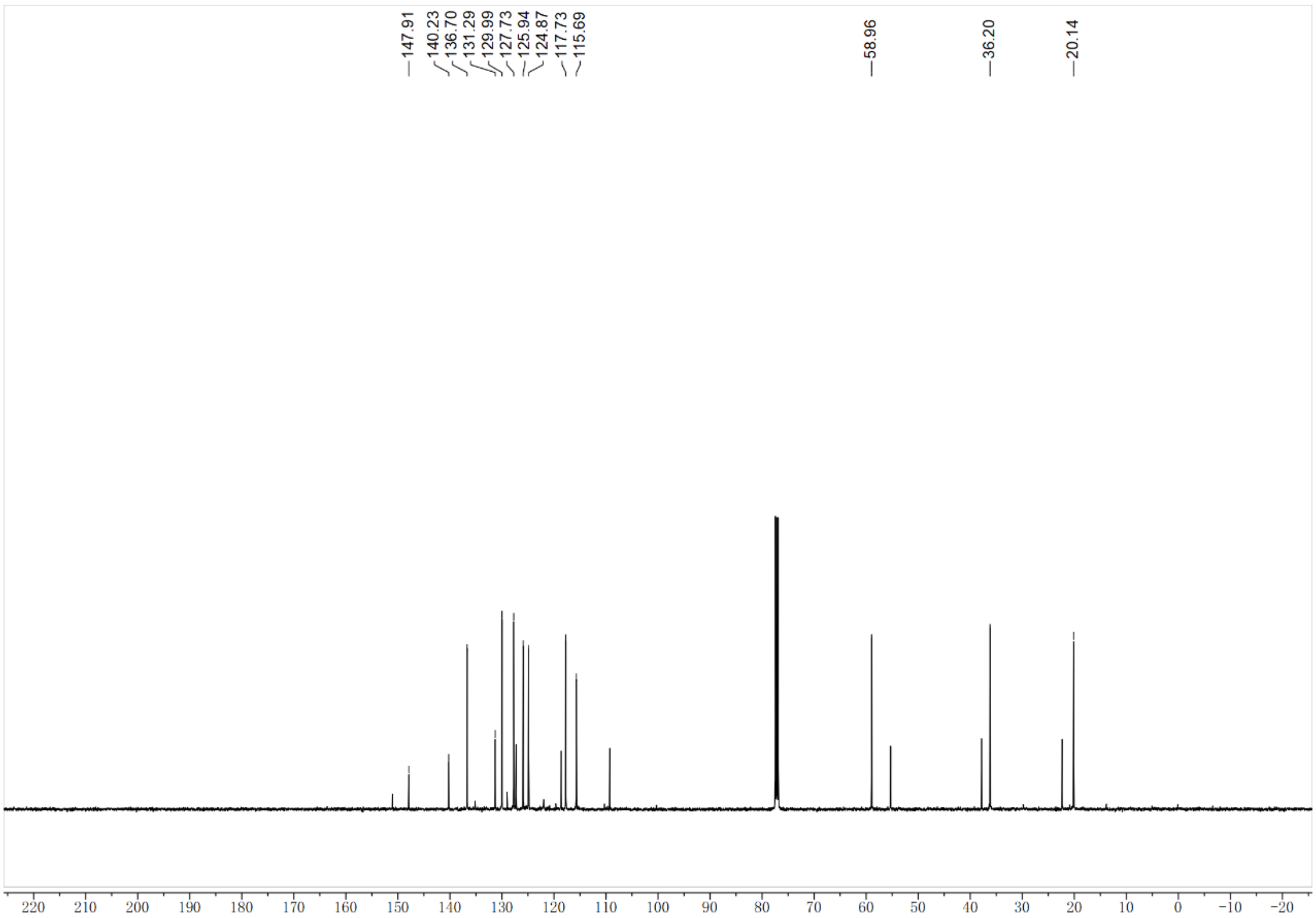

^{13}C NMR spectrum of compound DK16

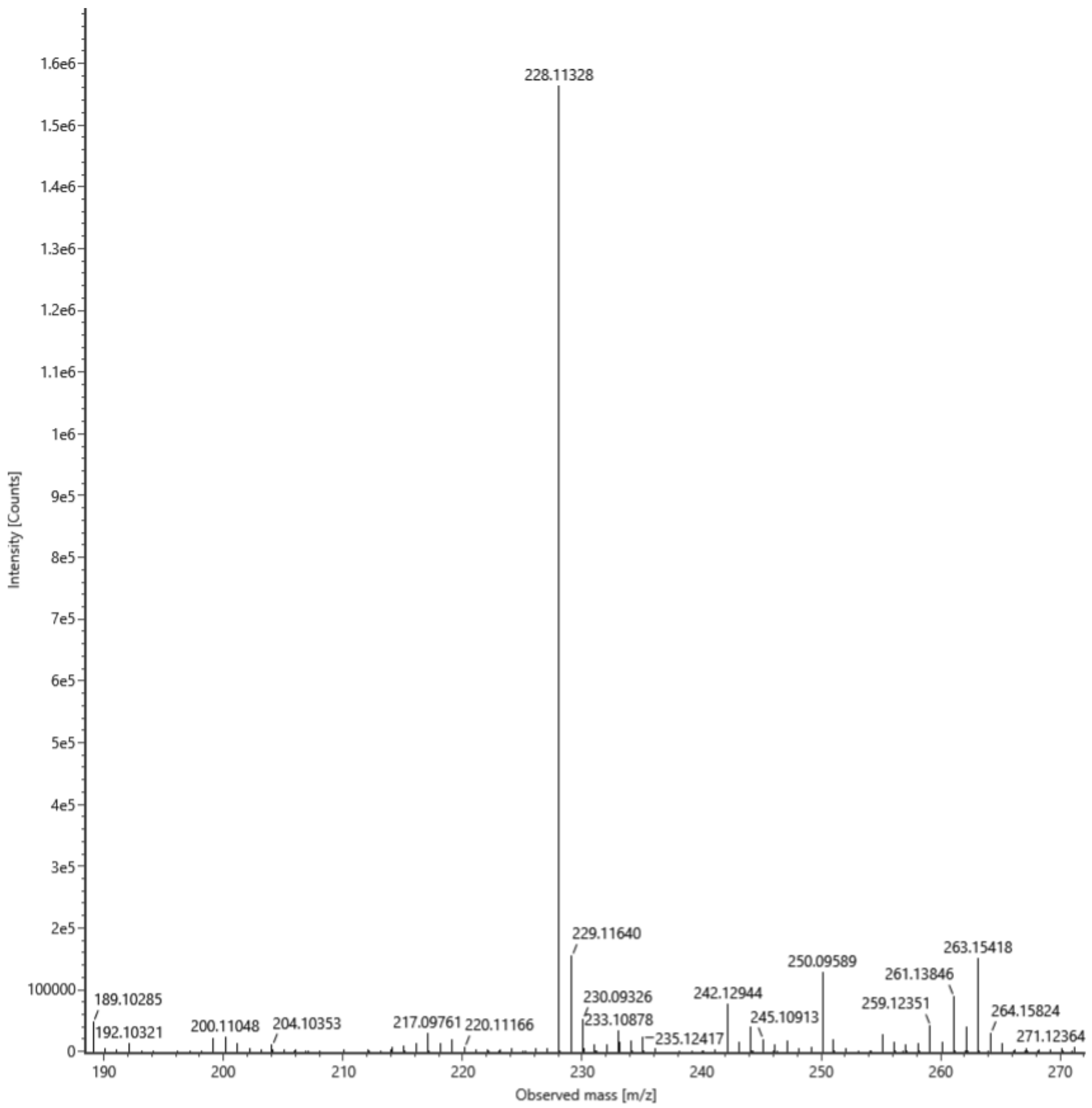

HRMS Spectrum of compound DK16

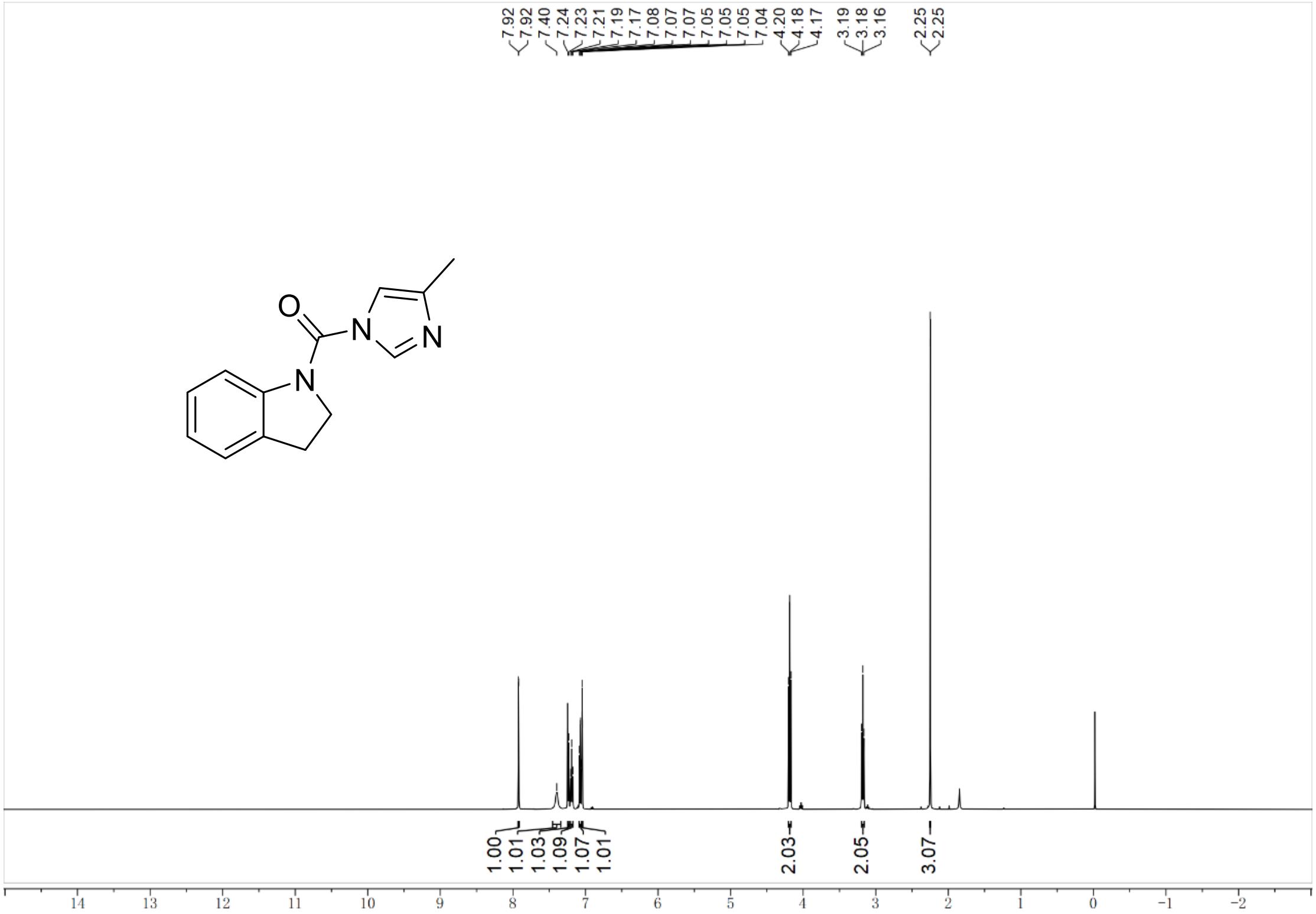

^{1}H NMR spectrum of compound DK17

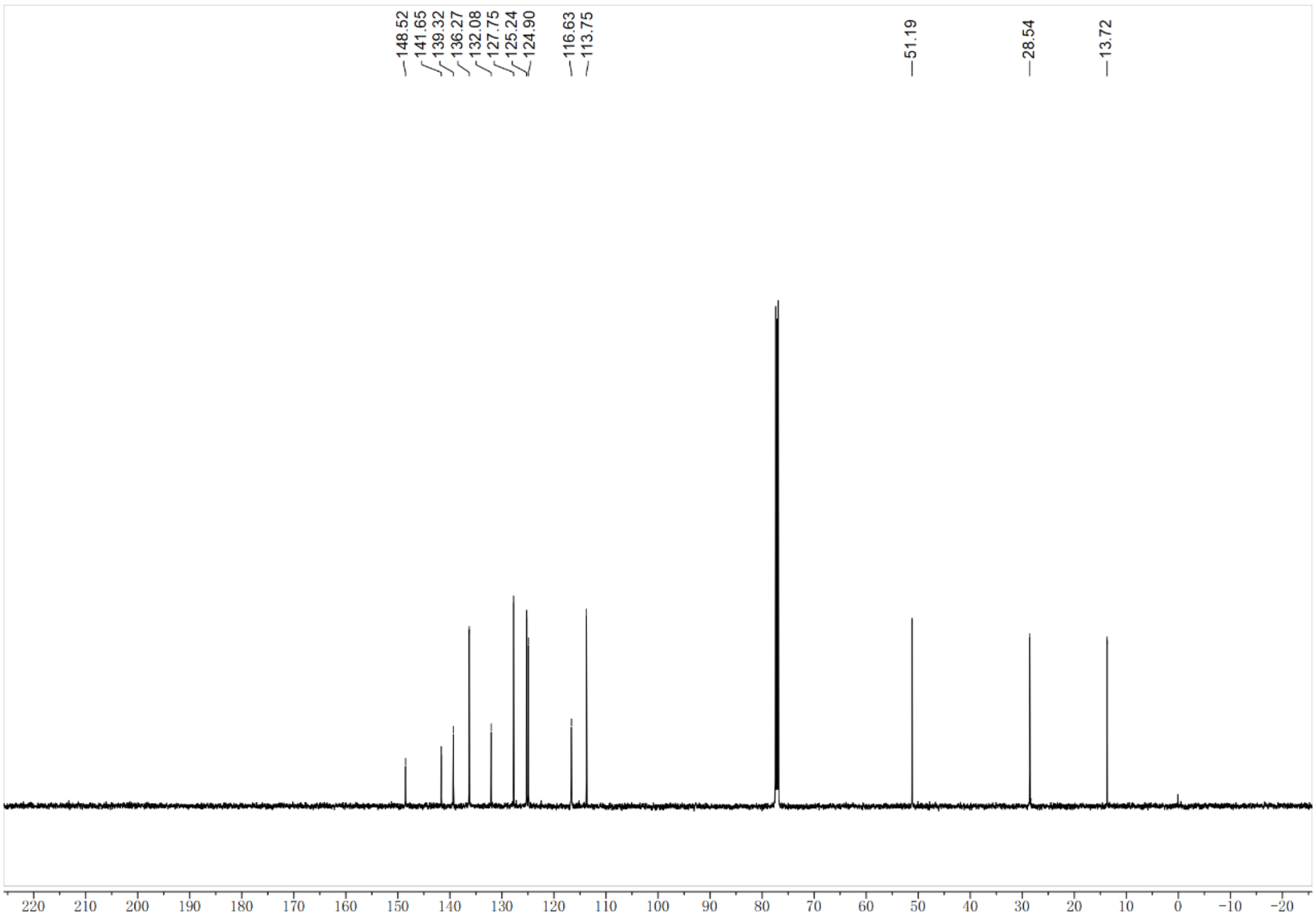

^{13}C NMR spectrum of compound DK17

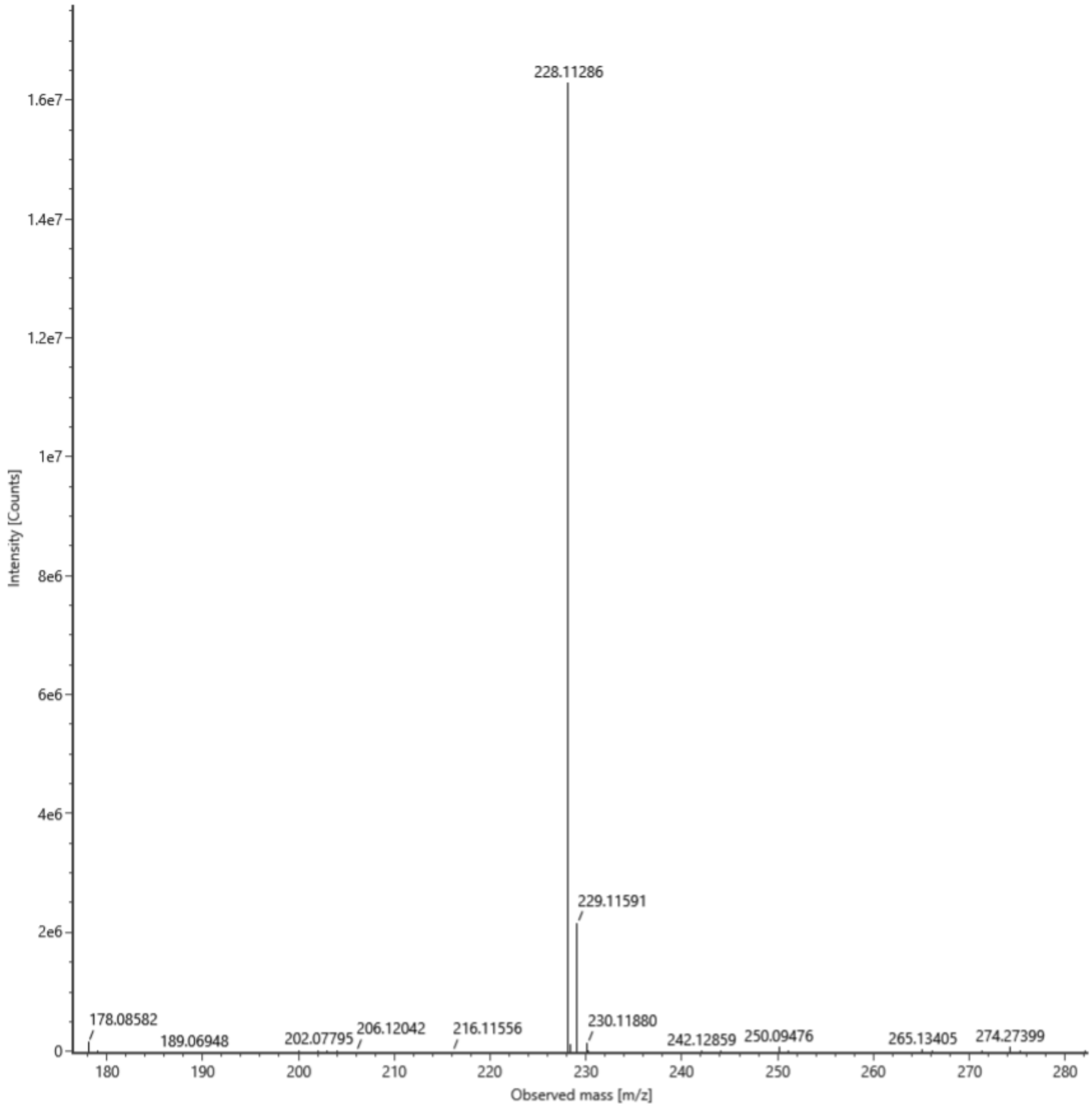

HRMS Spectrum of compound DK17

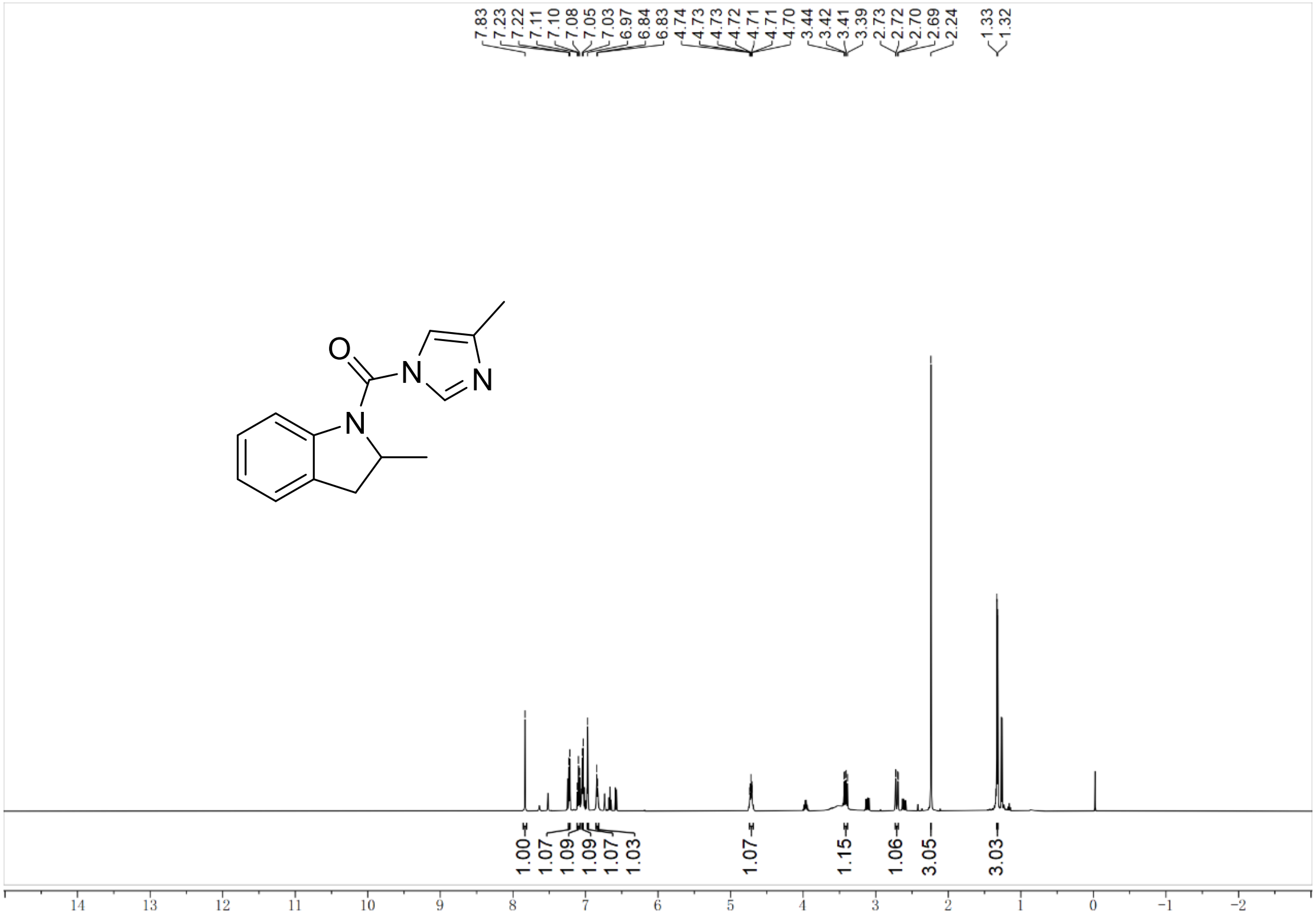

^{1}H NMR spectrum of compound DK18

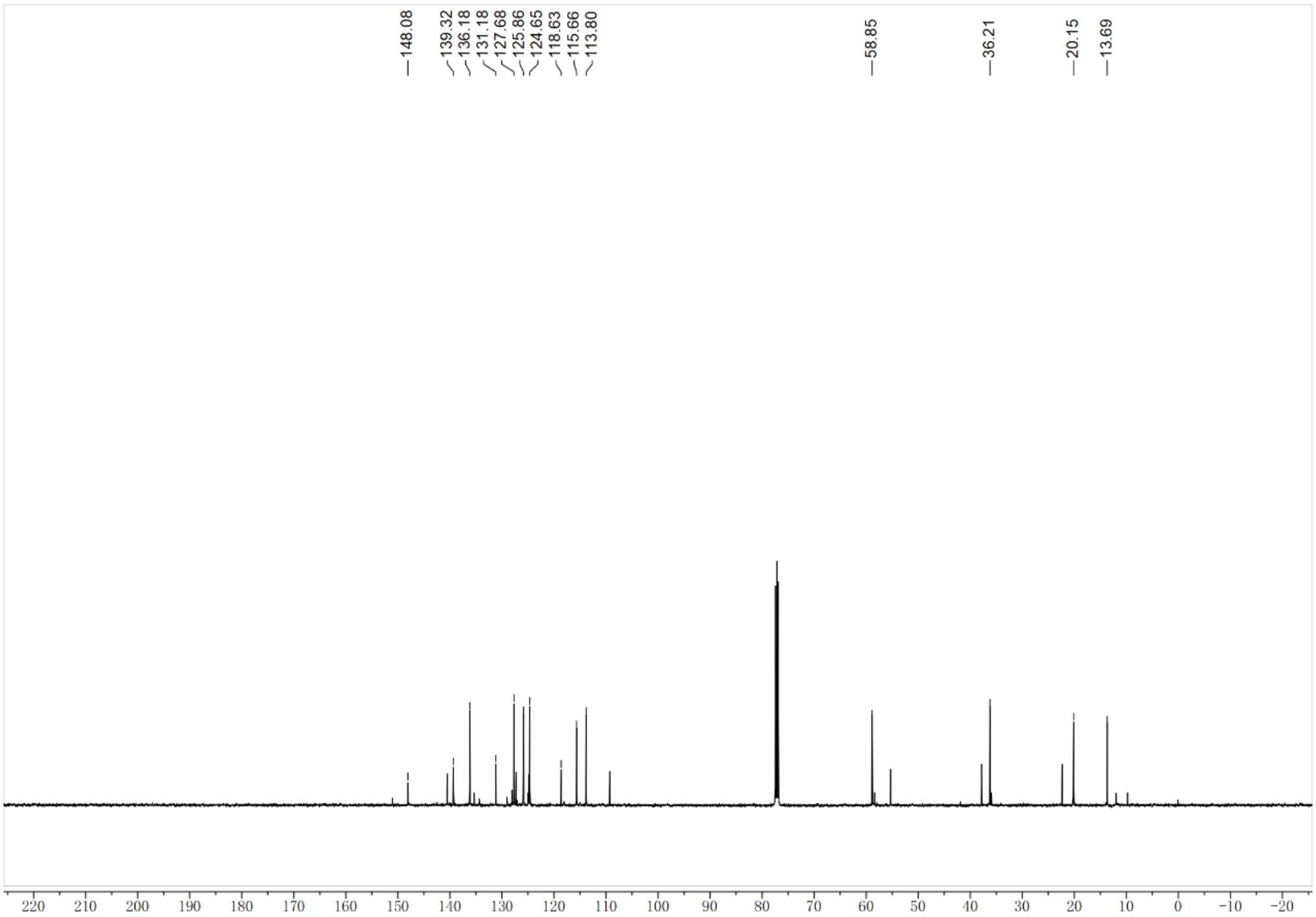

^{13}C NMR spectrum of compound DK18

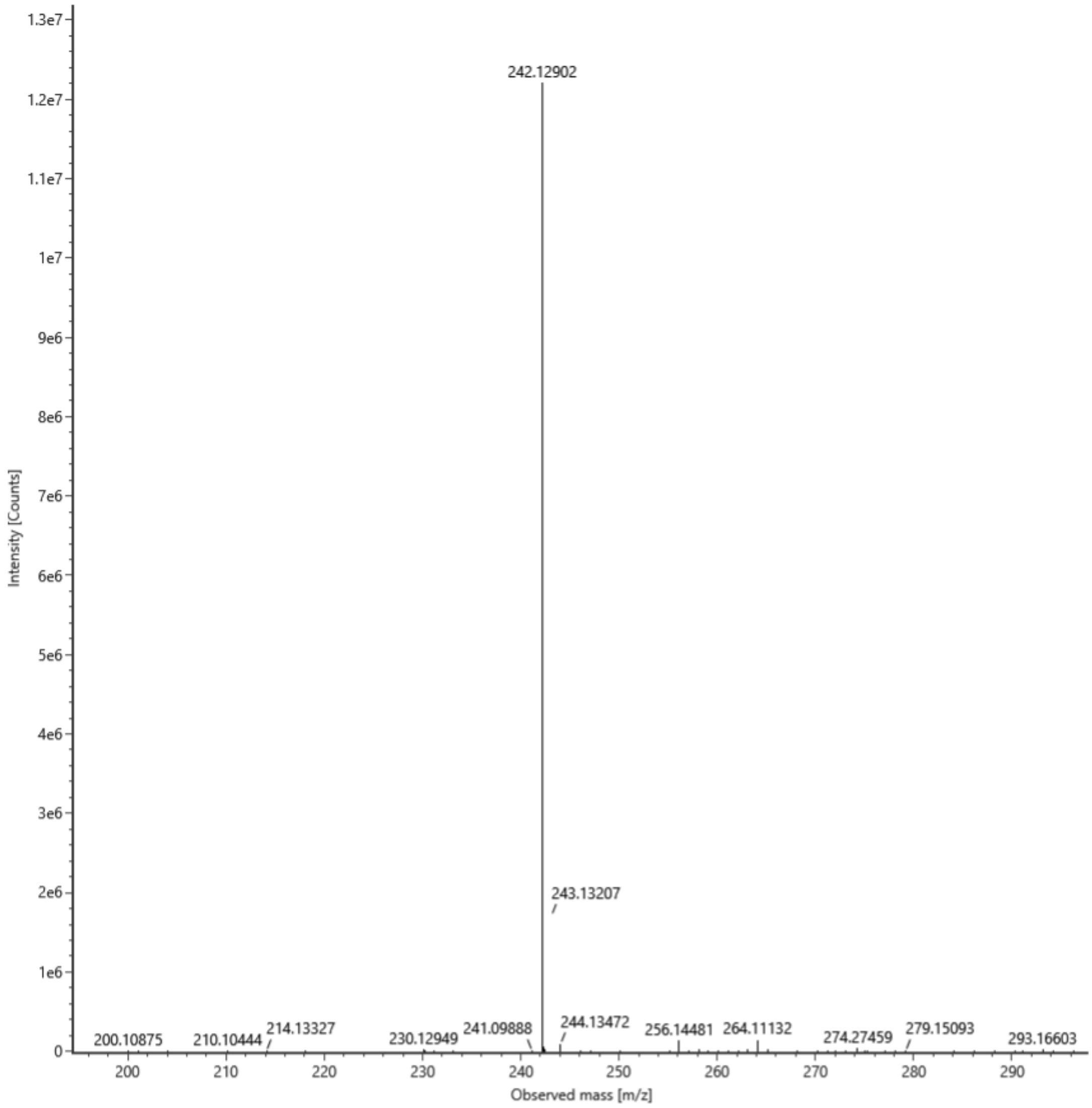

HRMS Spectrum of compound DK18

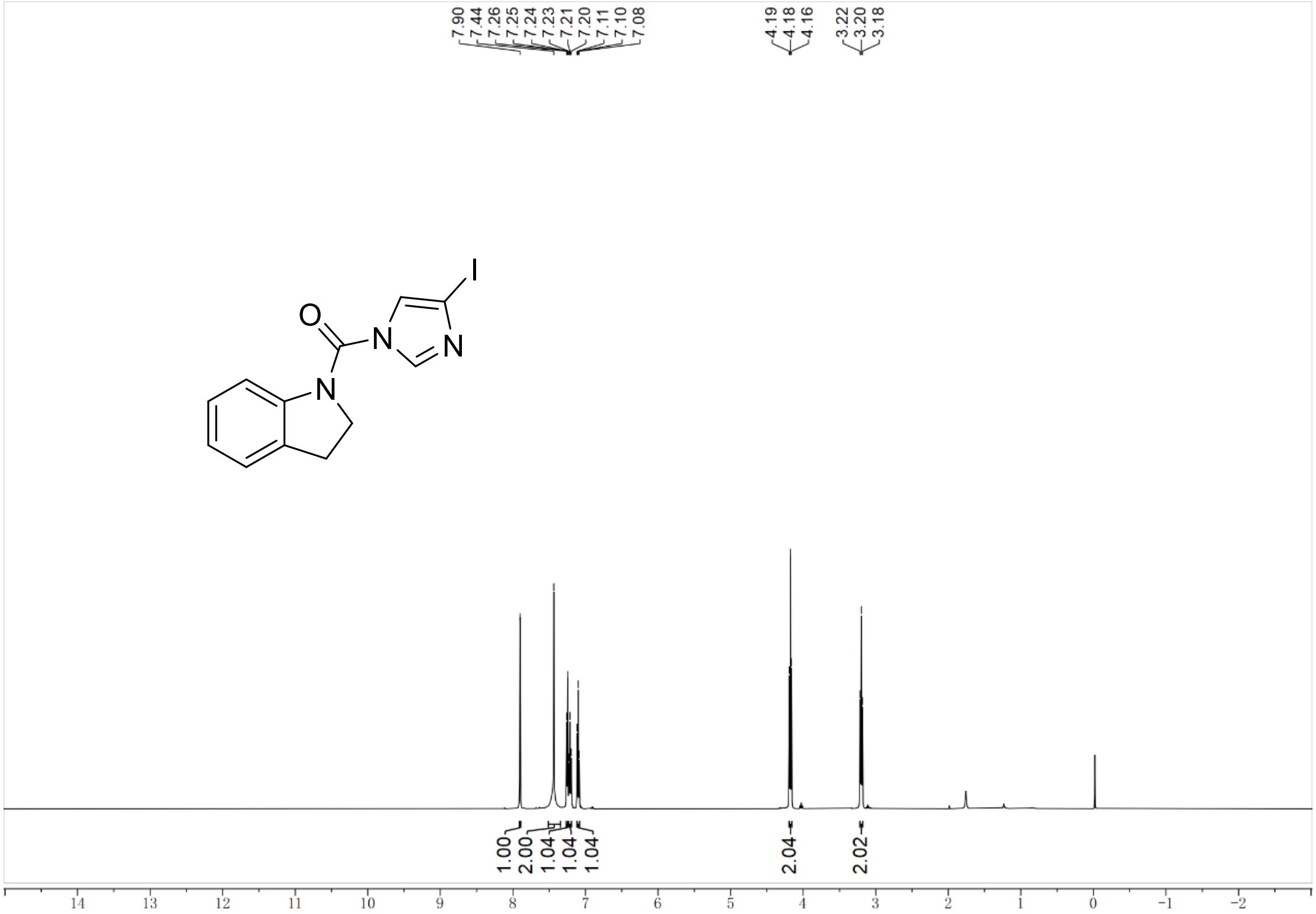

^{1}H NMR spectrum of compound DK19

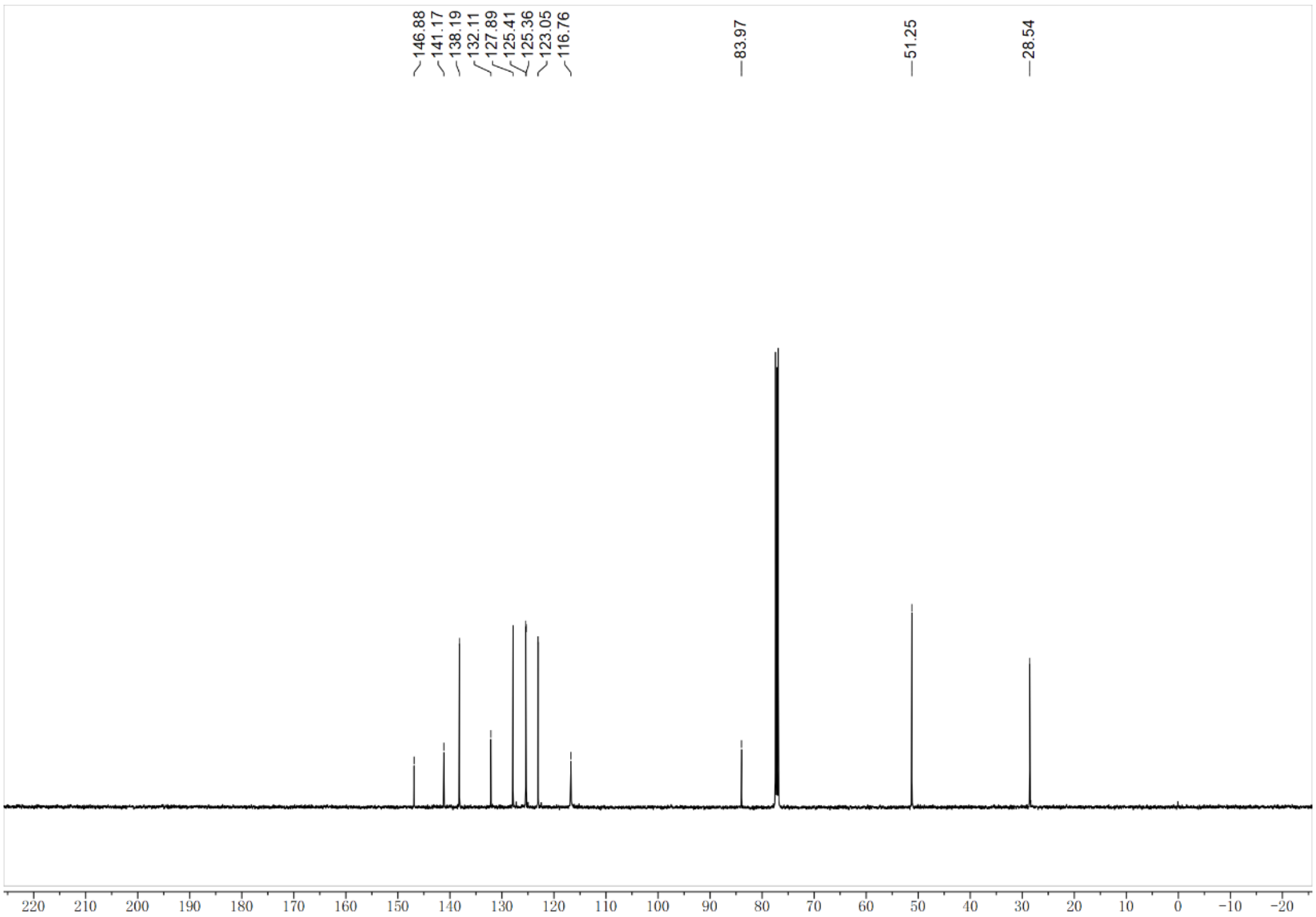

^{13}C NMR spectrum of compound DK19

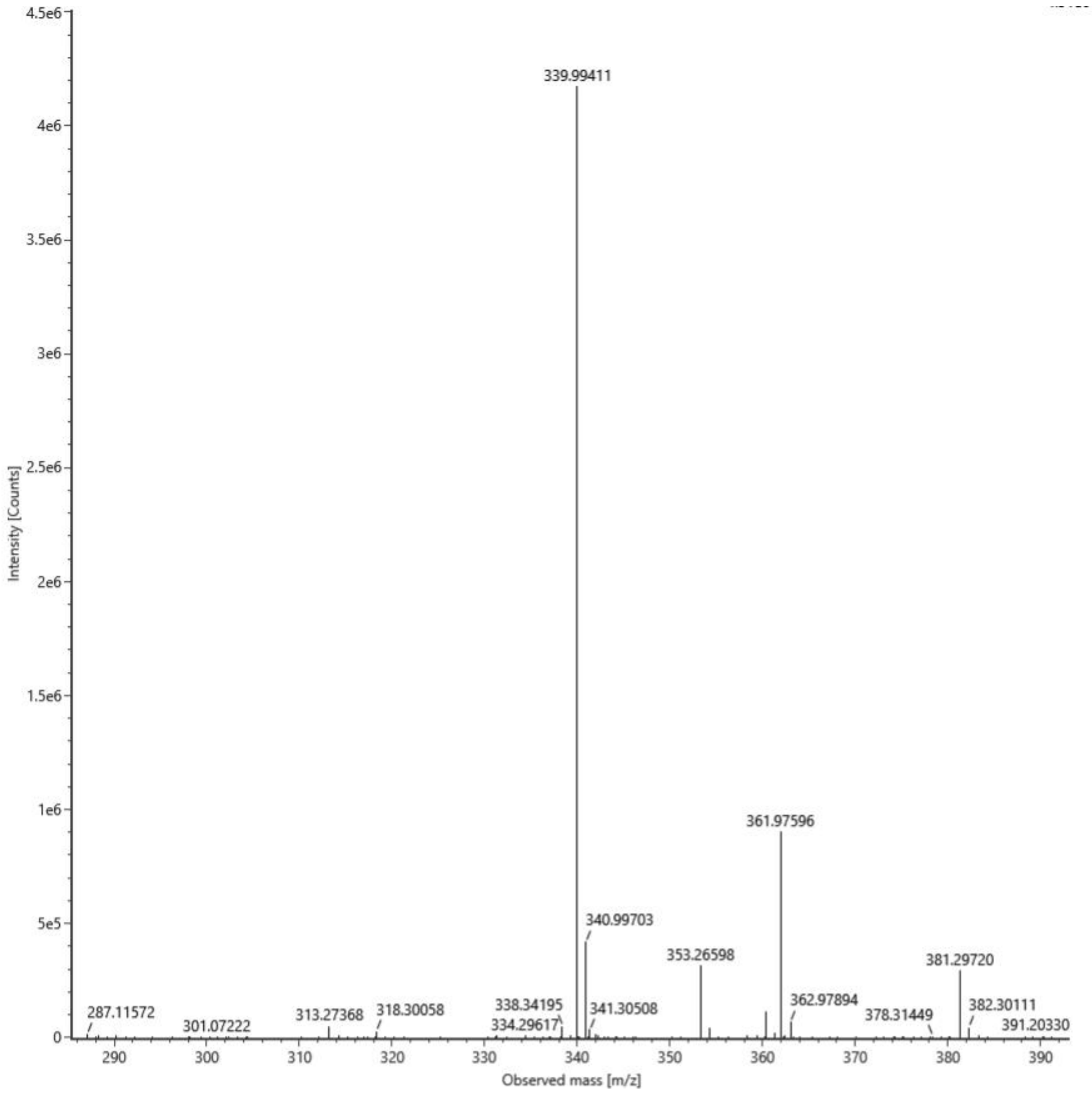

HRMS Spectrum of compound DK19

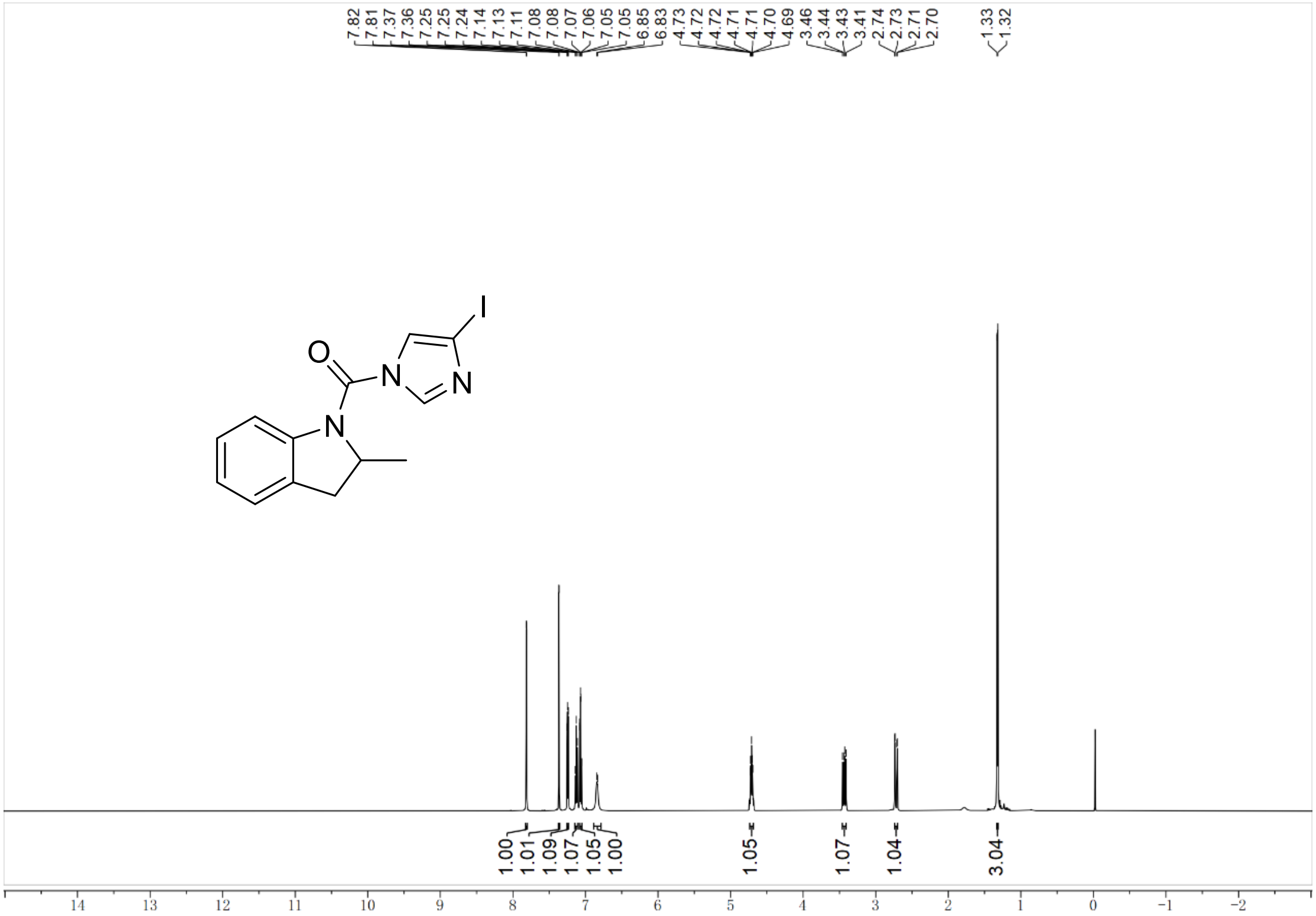

^{1}H NMR spectrum of compound DK20

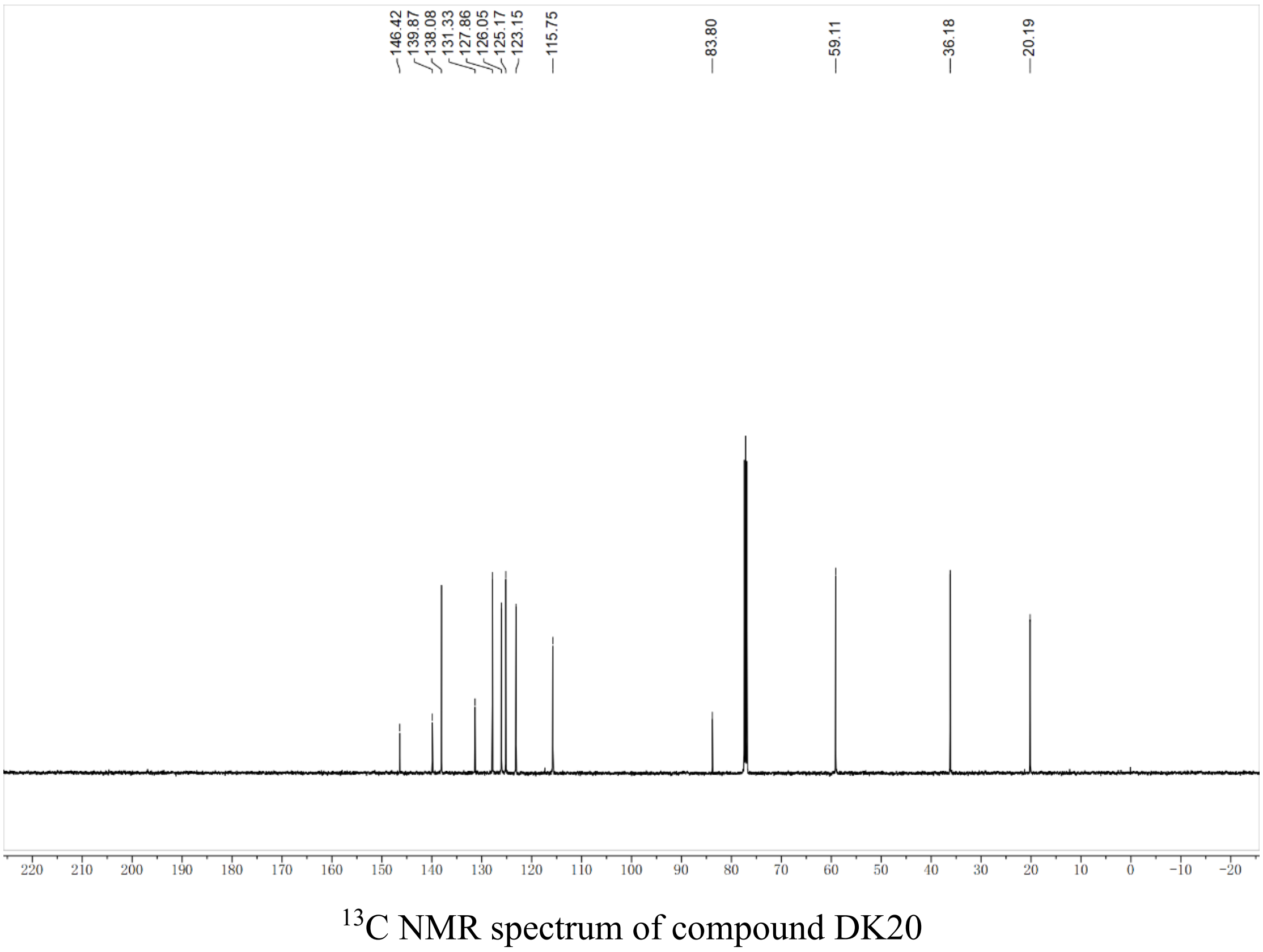

^{13}C NMR spectrum of compound DK20

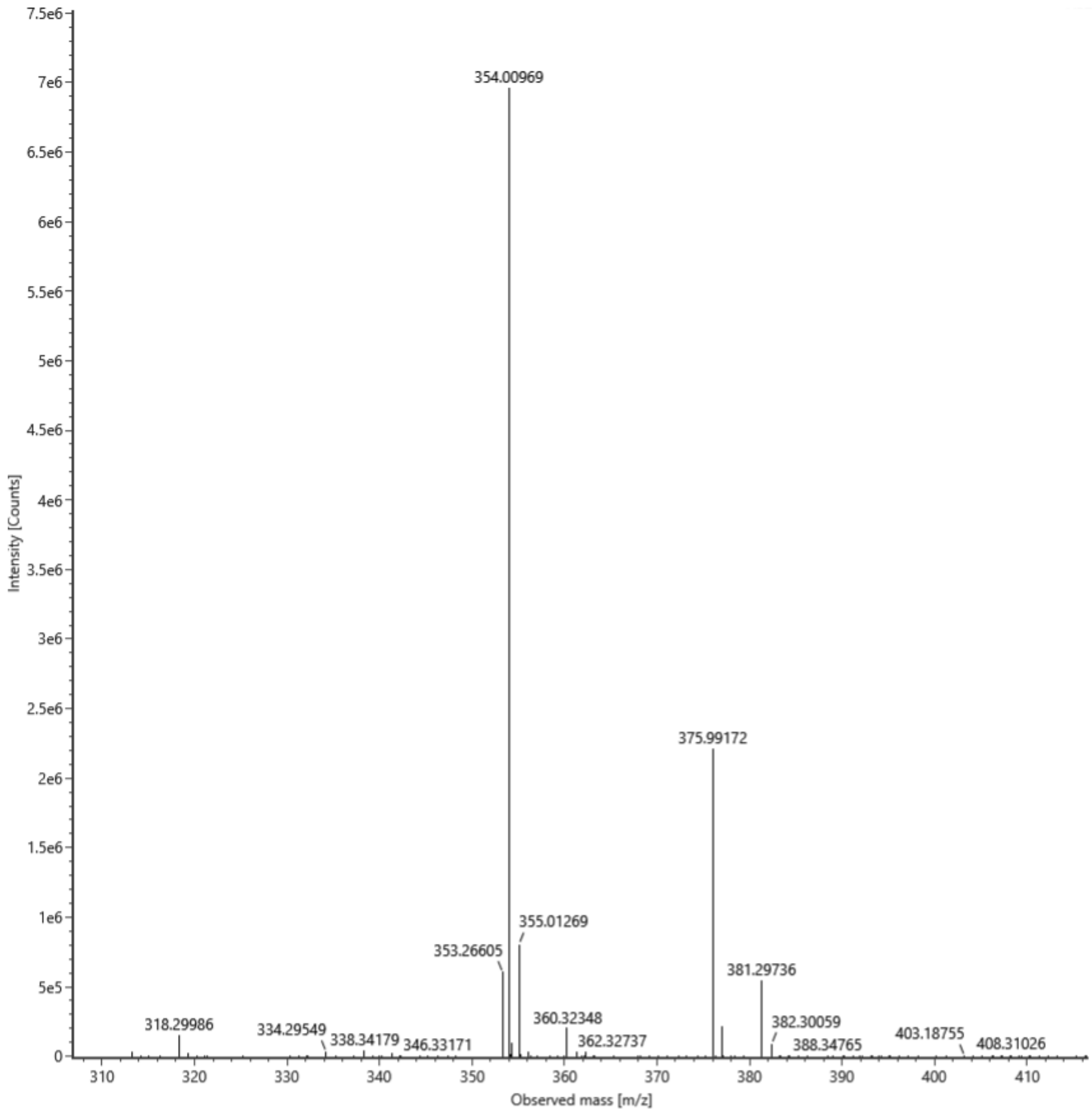

HRMS Spectrum of compound DK20

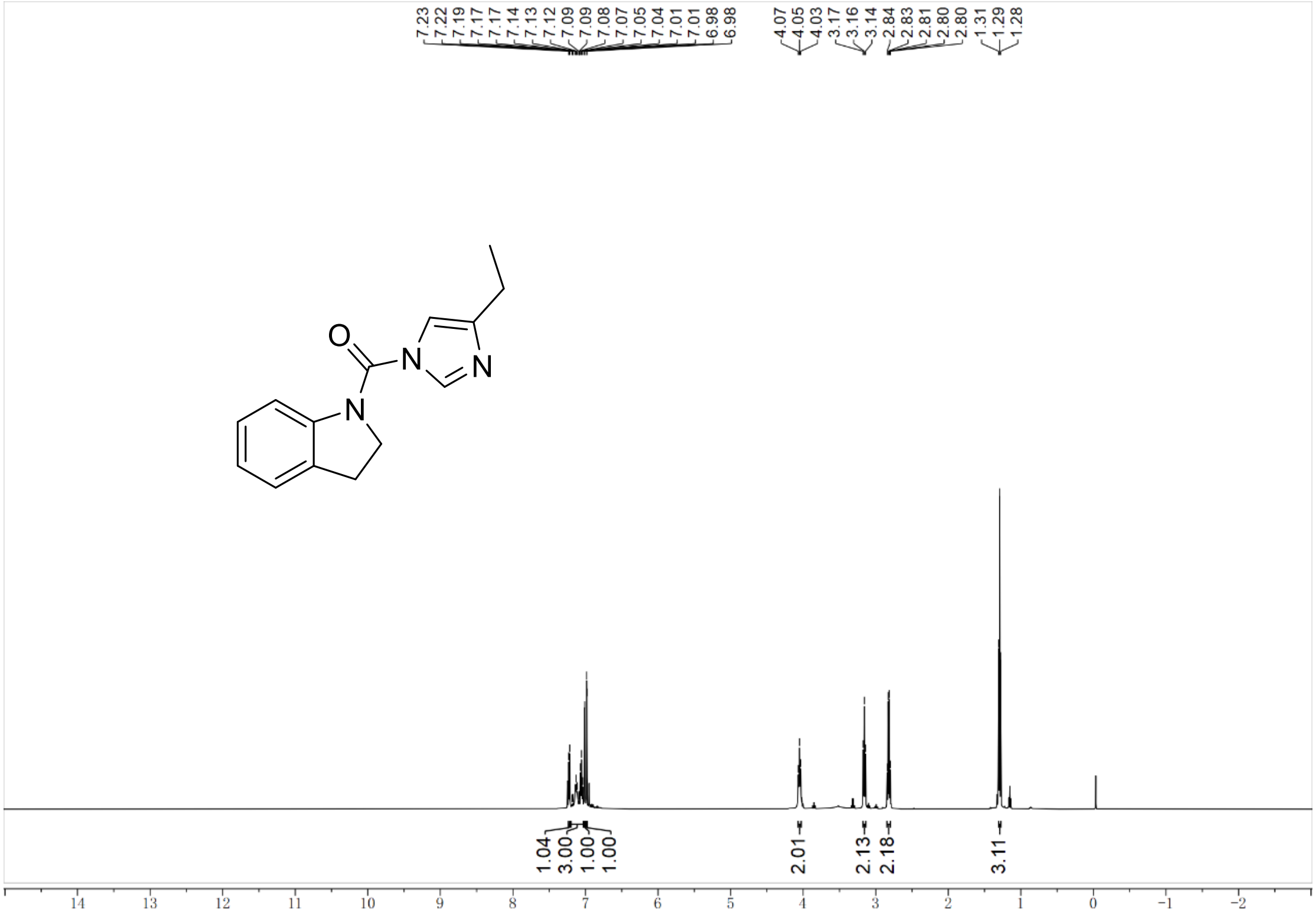

^{1}H NMR spectrum of compound DK21

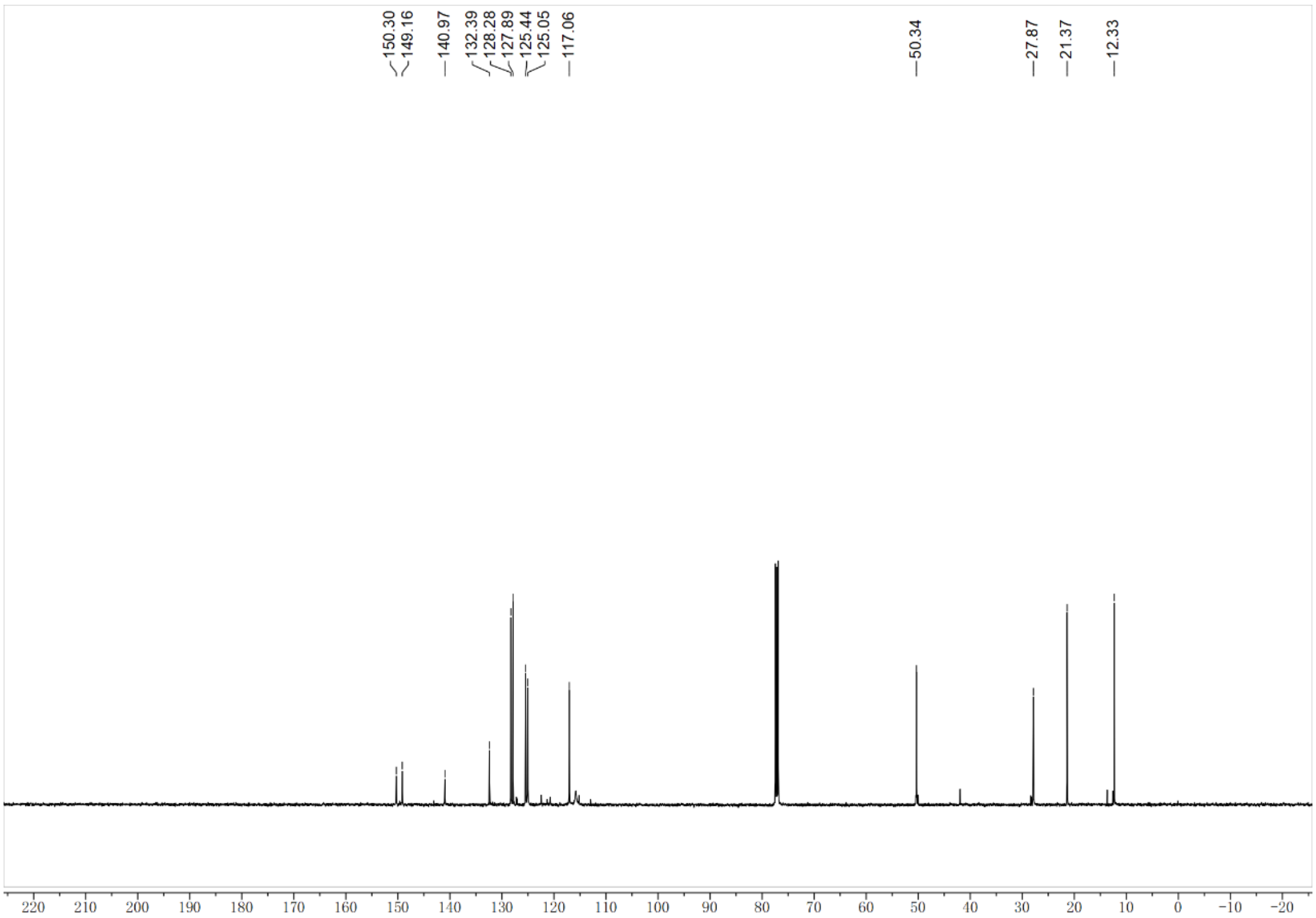

^{13}C NMR spectrum of compound DK21

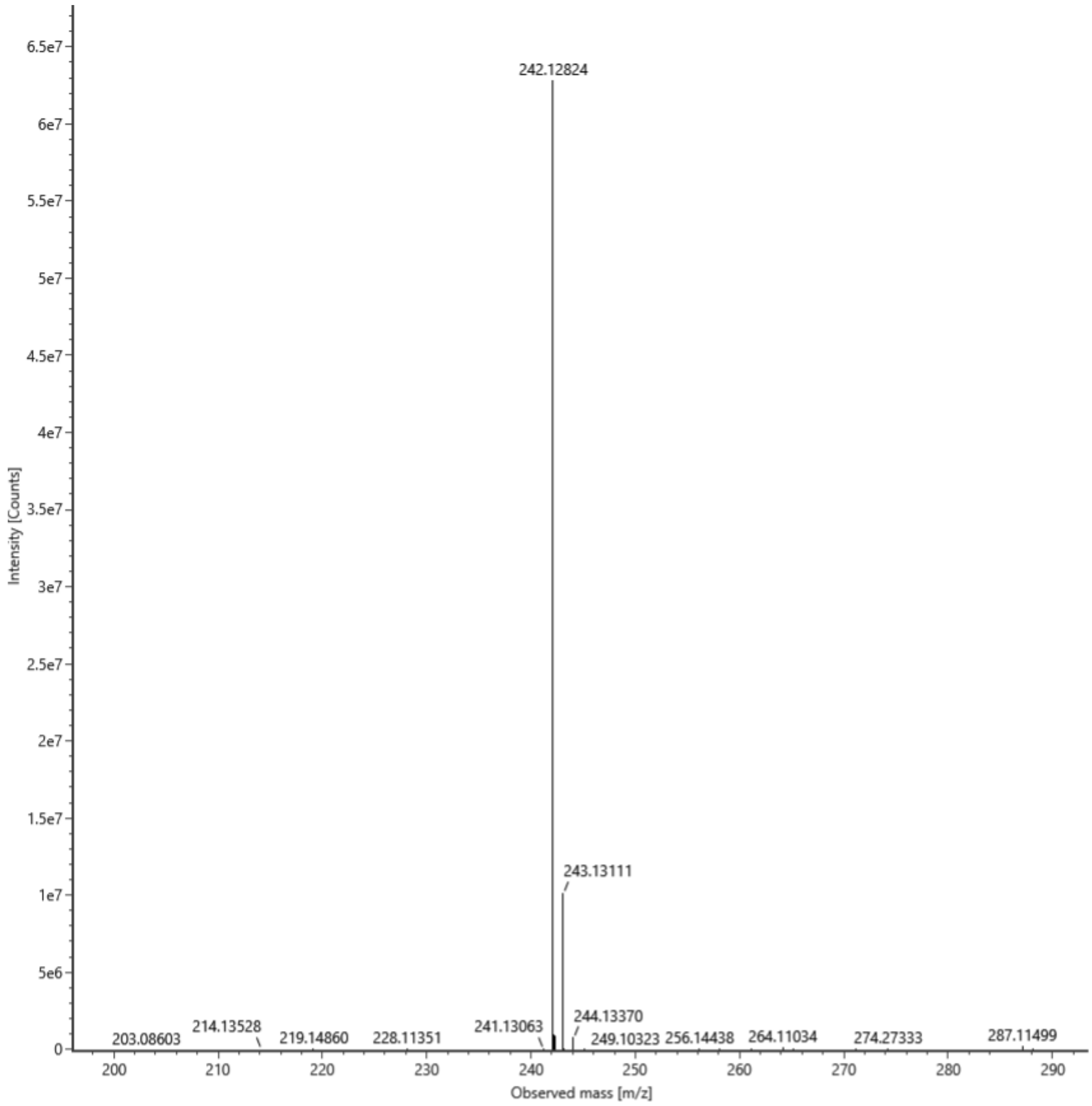

HRMS Spectrum of compound DK21

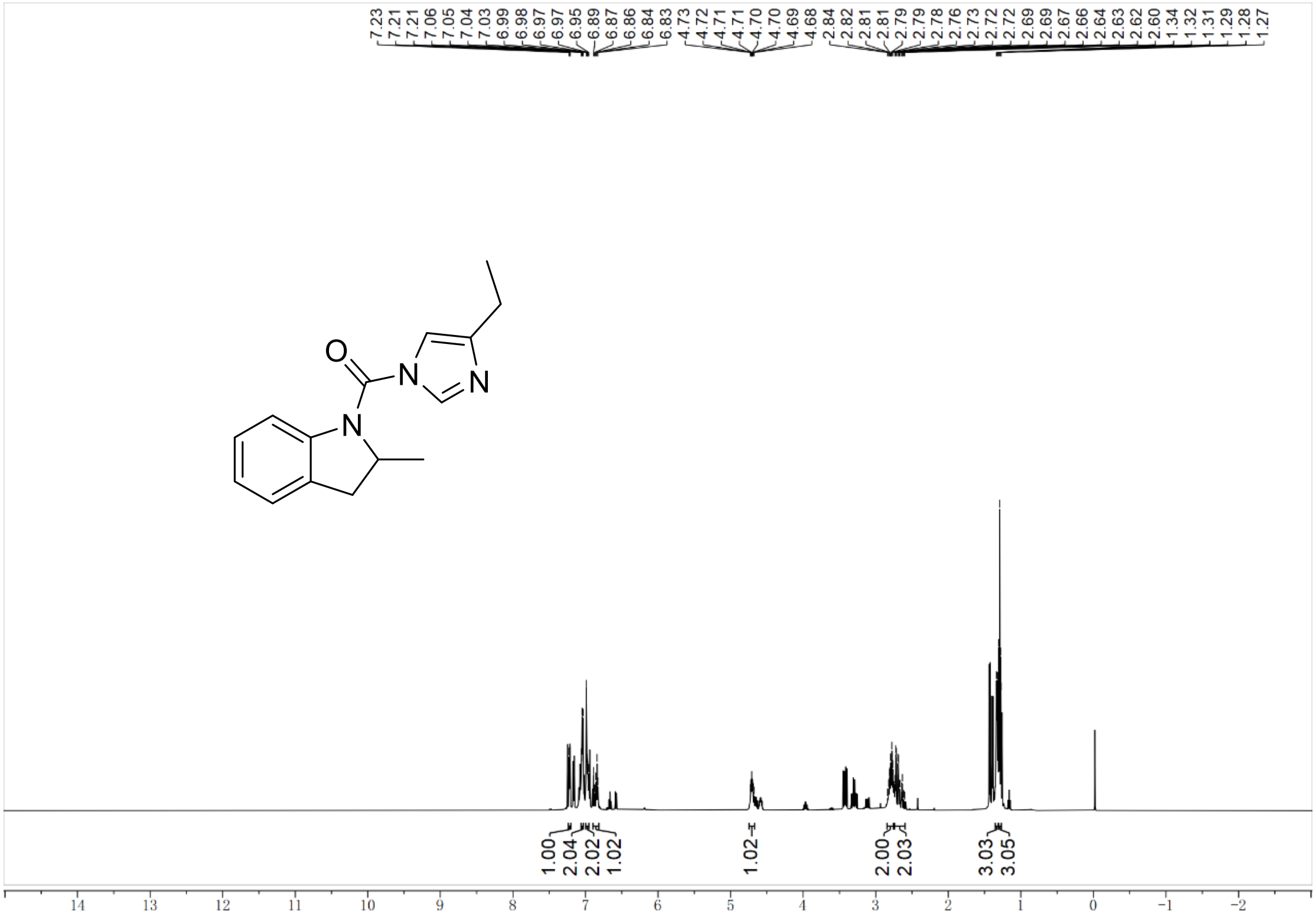

^{1}H NMR spectrum of compound DK22

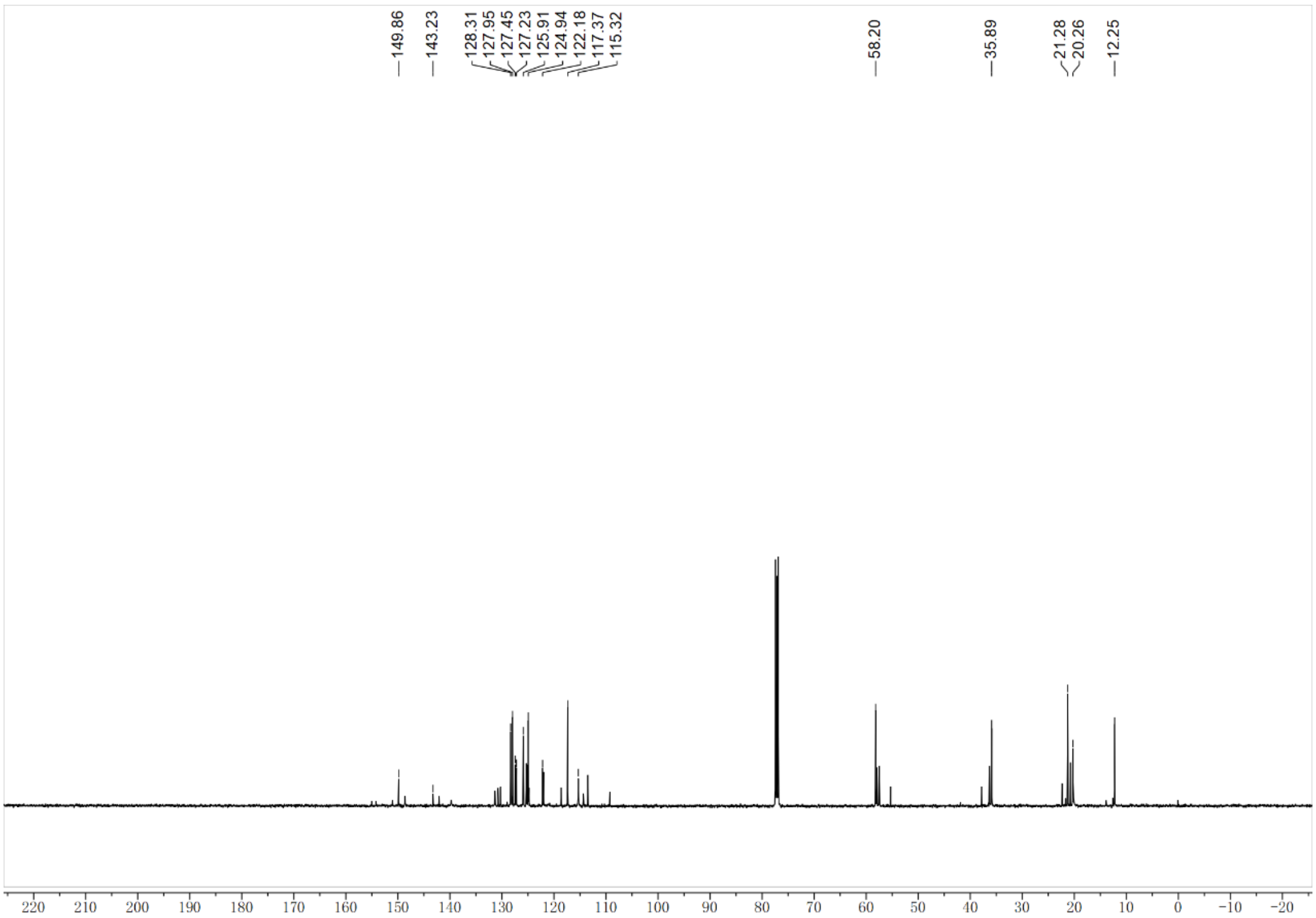

^{13}C NMR spectrum of compound DK22

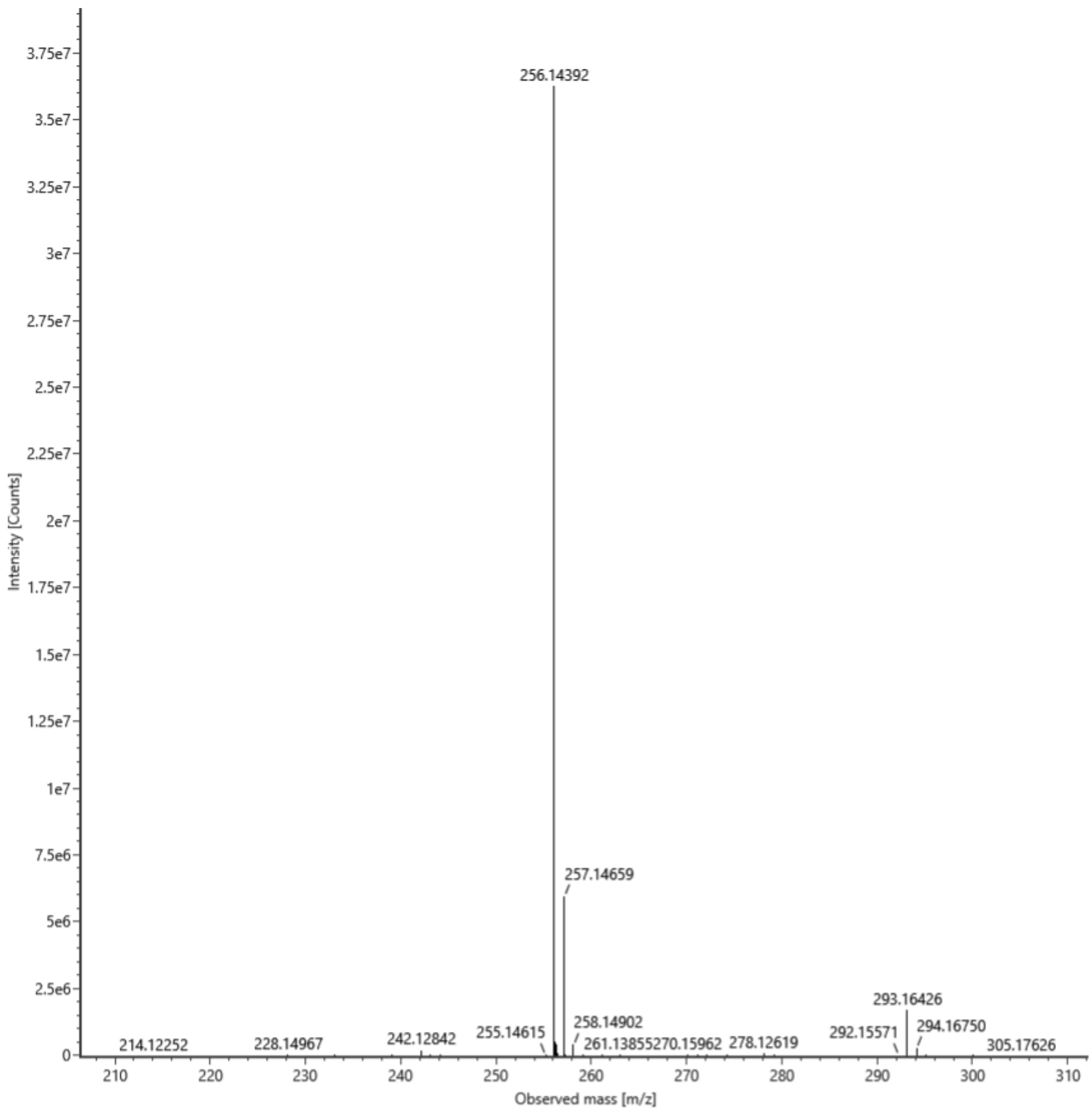

HRMS Spectrum of compound DK22

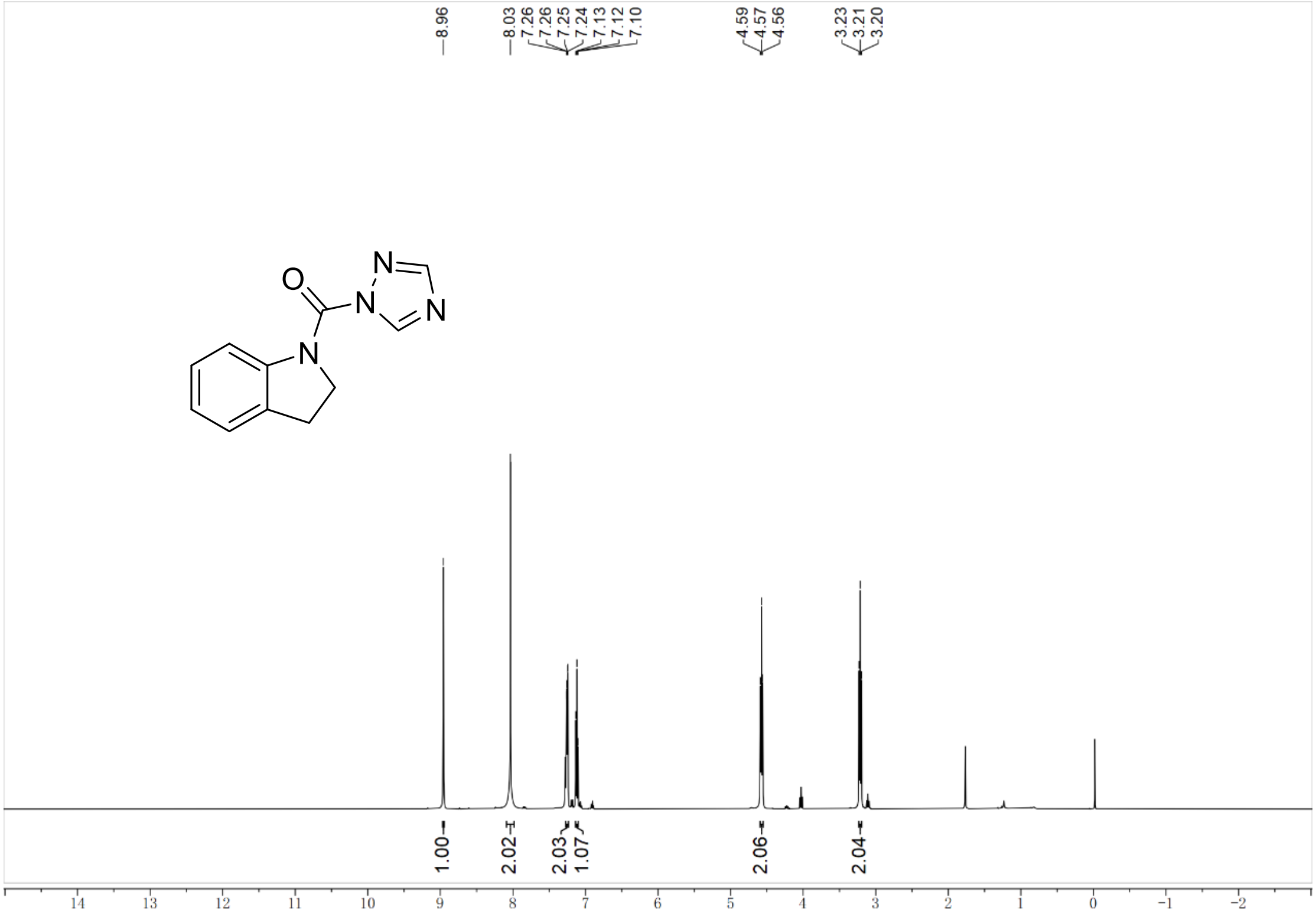

^{1}H NMR spectrum of compound DK23

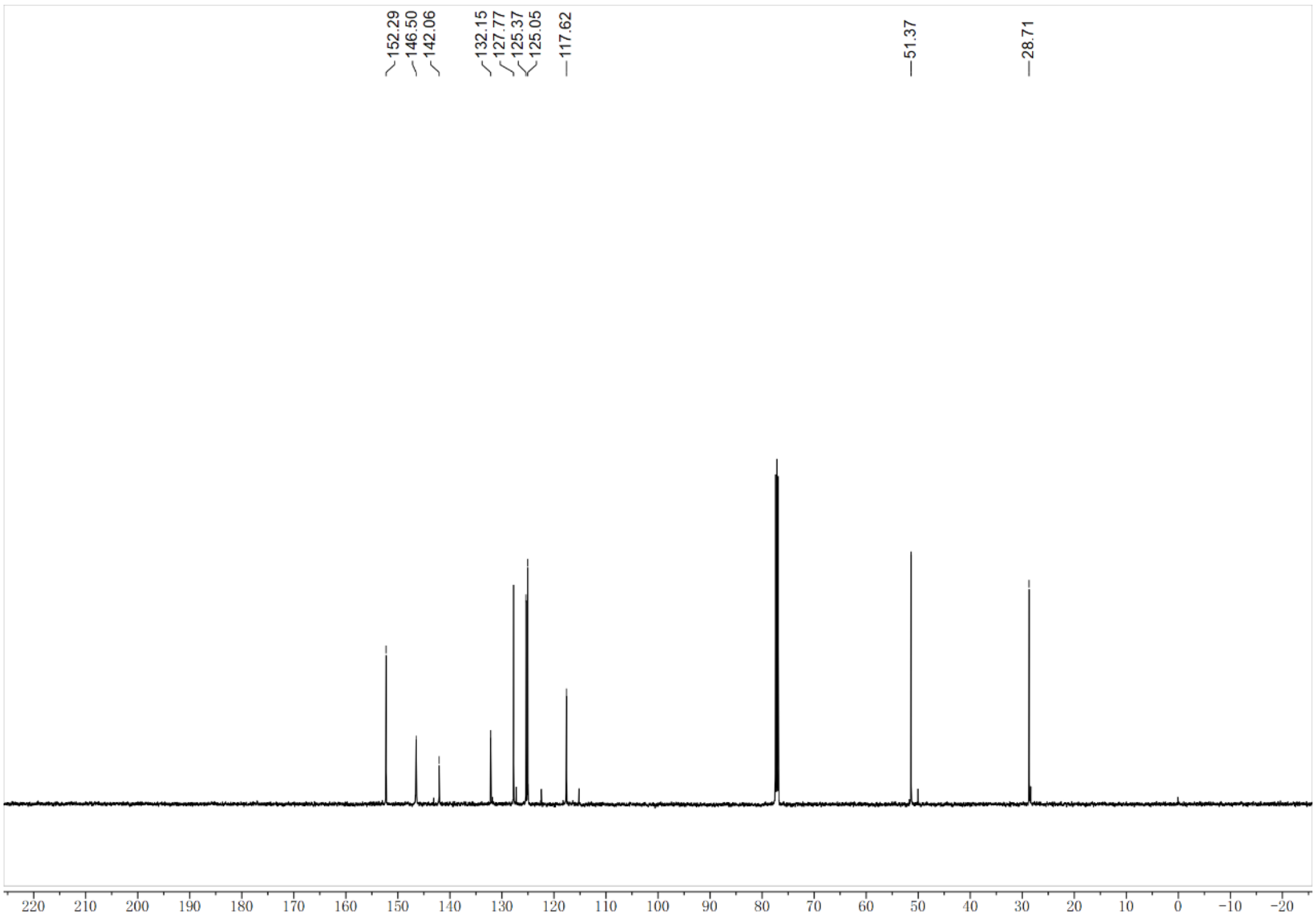

^{13}C NMR spectrum of compound DK23

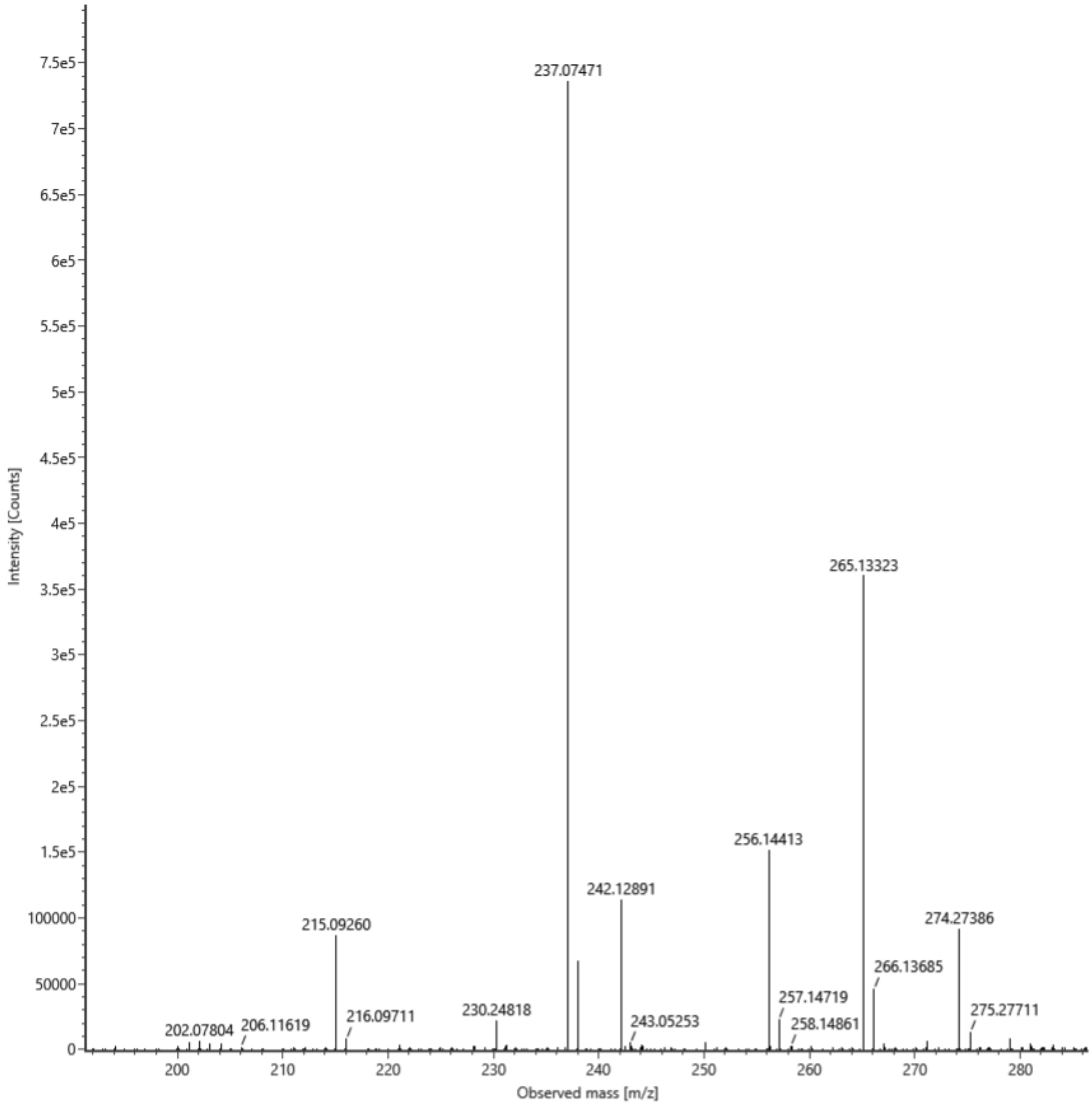

HRMS Spectrum of compound DK23

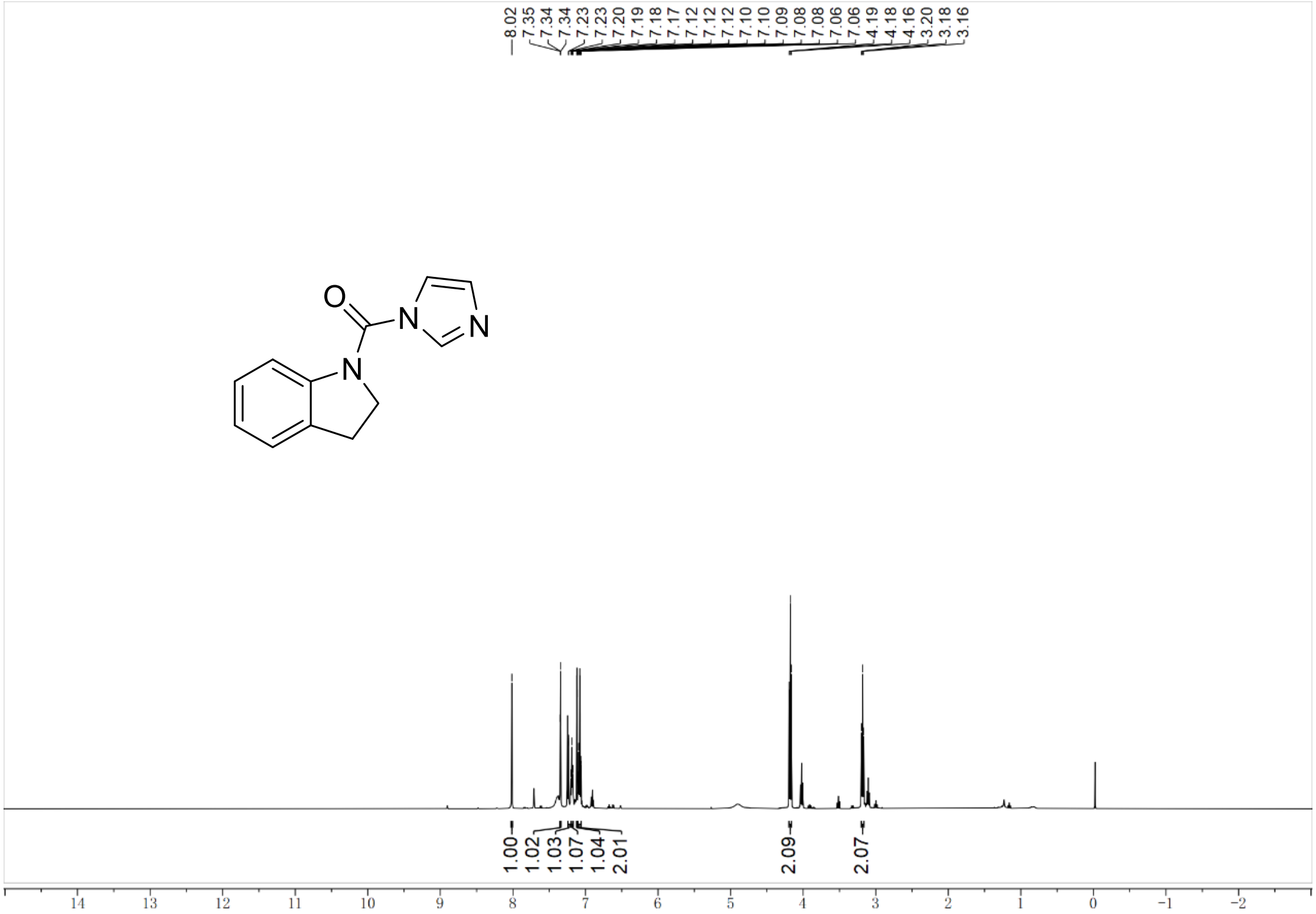

^{1}H NMR spectrum of compound DK24

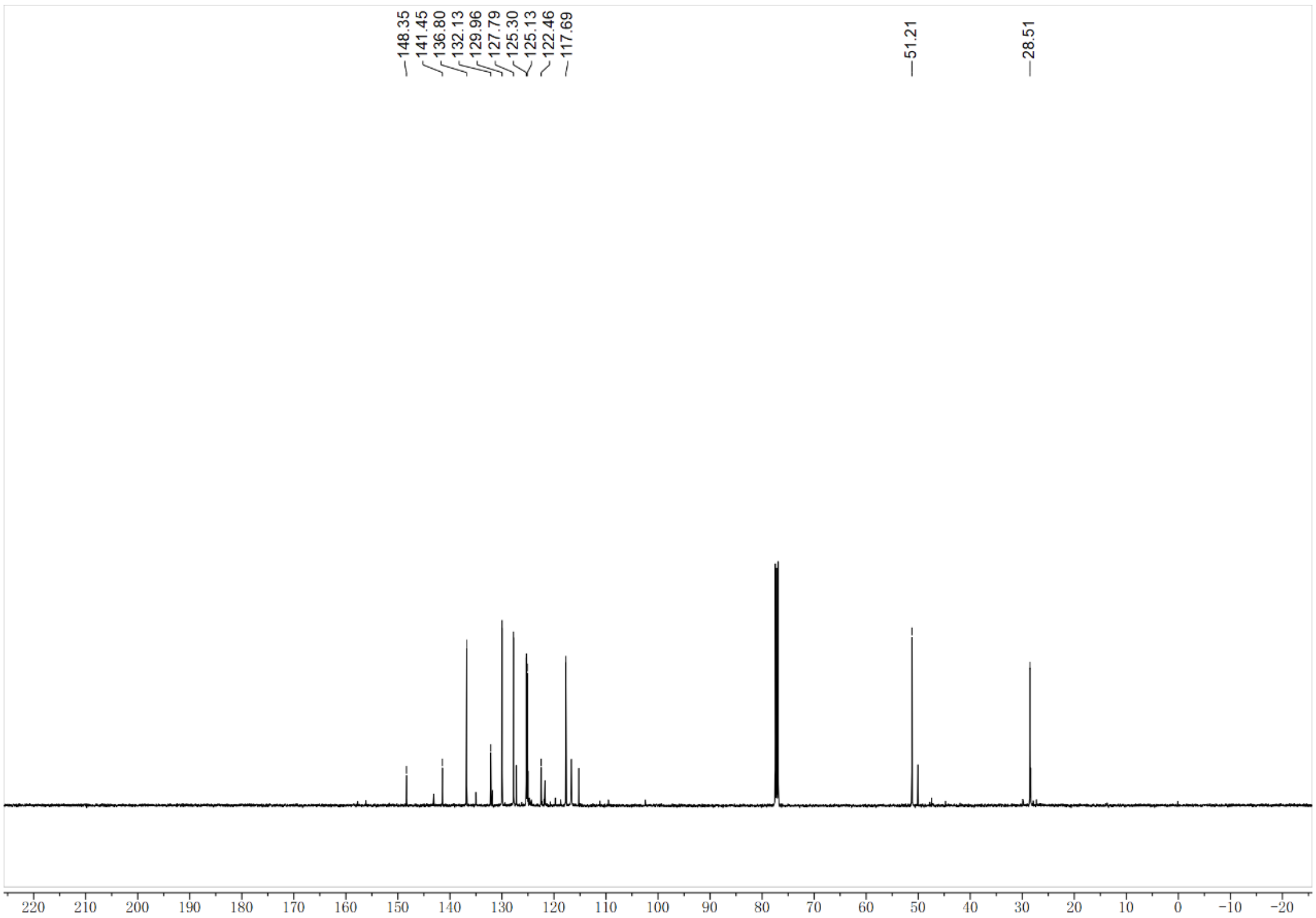

^{13}C NMR spectrum of compound DK24

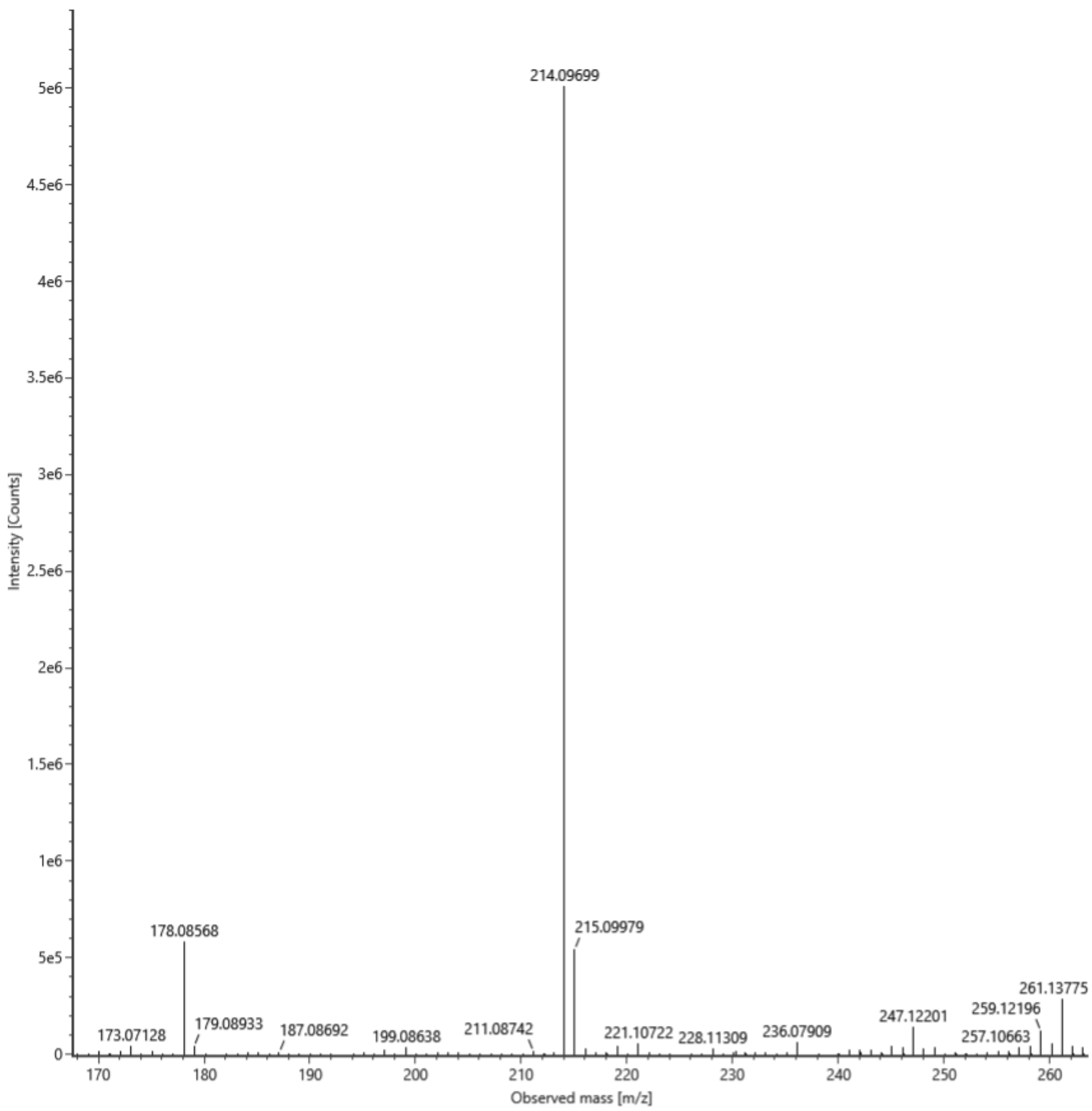

HRMS Spectrum of compound DK24

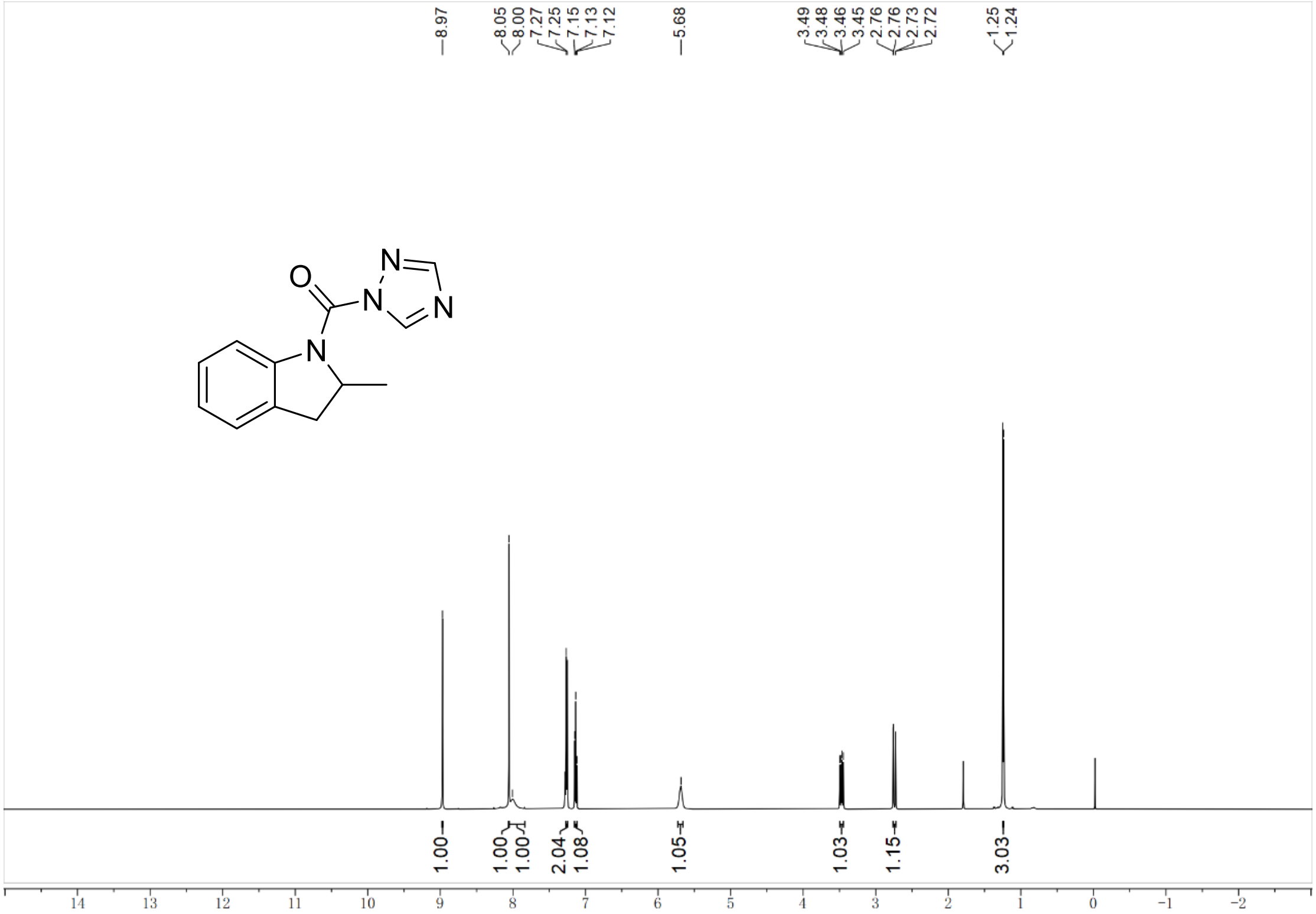

^{1}H NMR spectrum of compound DK25

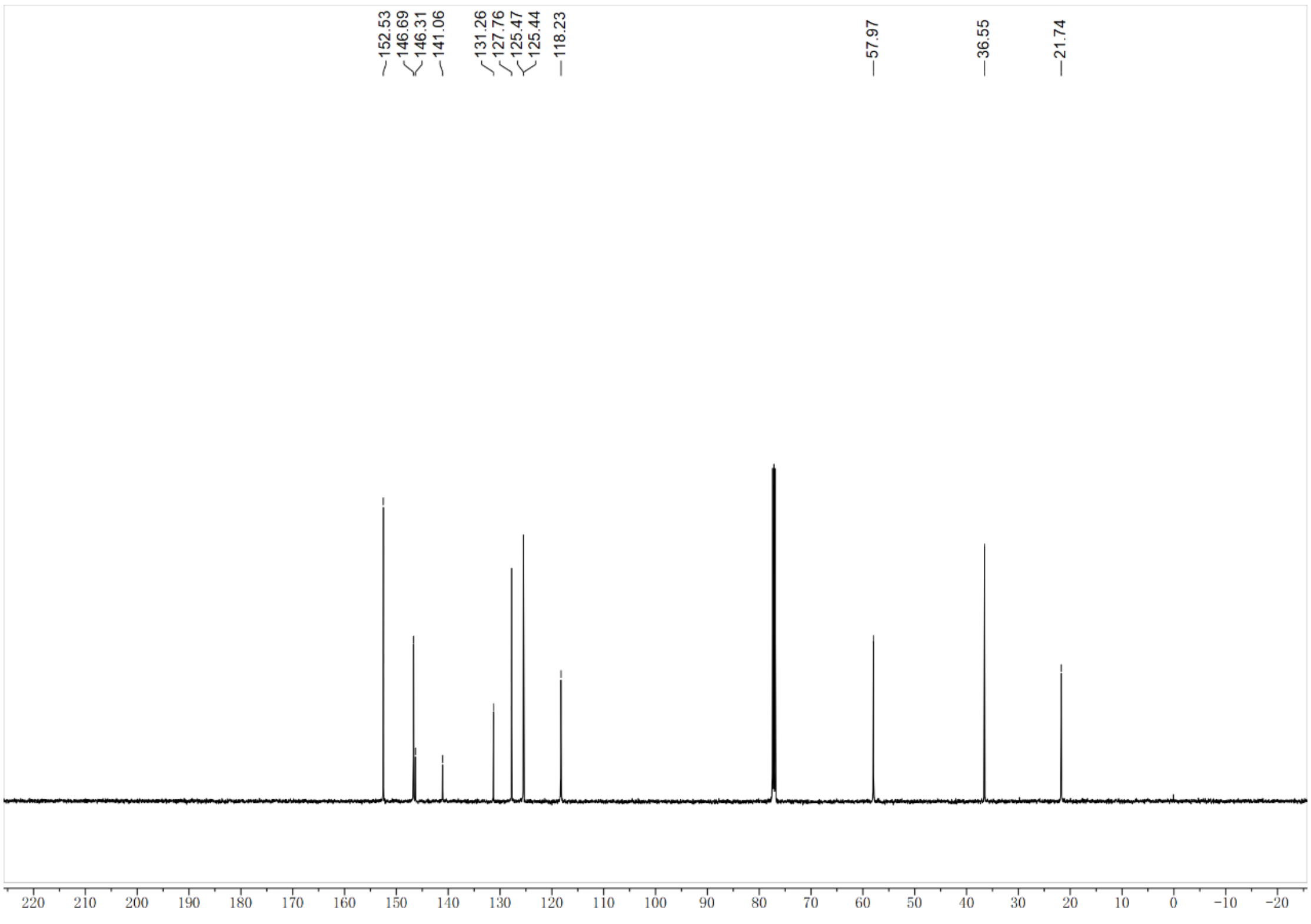

^{13}C NMR spectrum of compound DK25

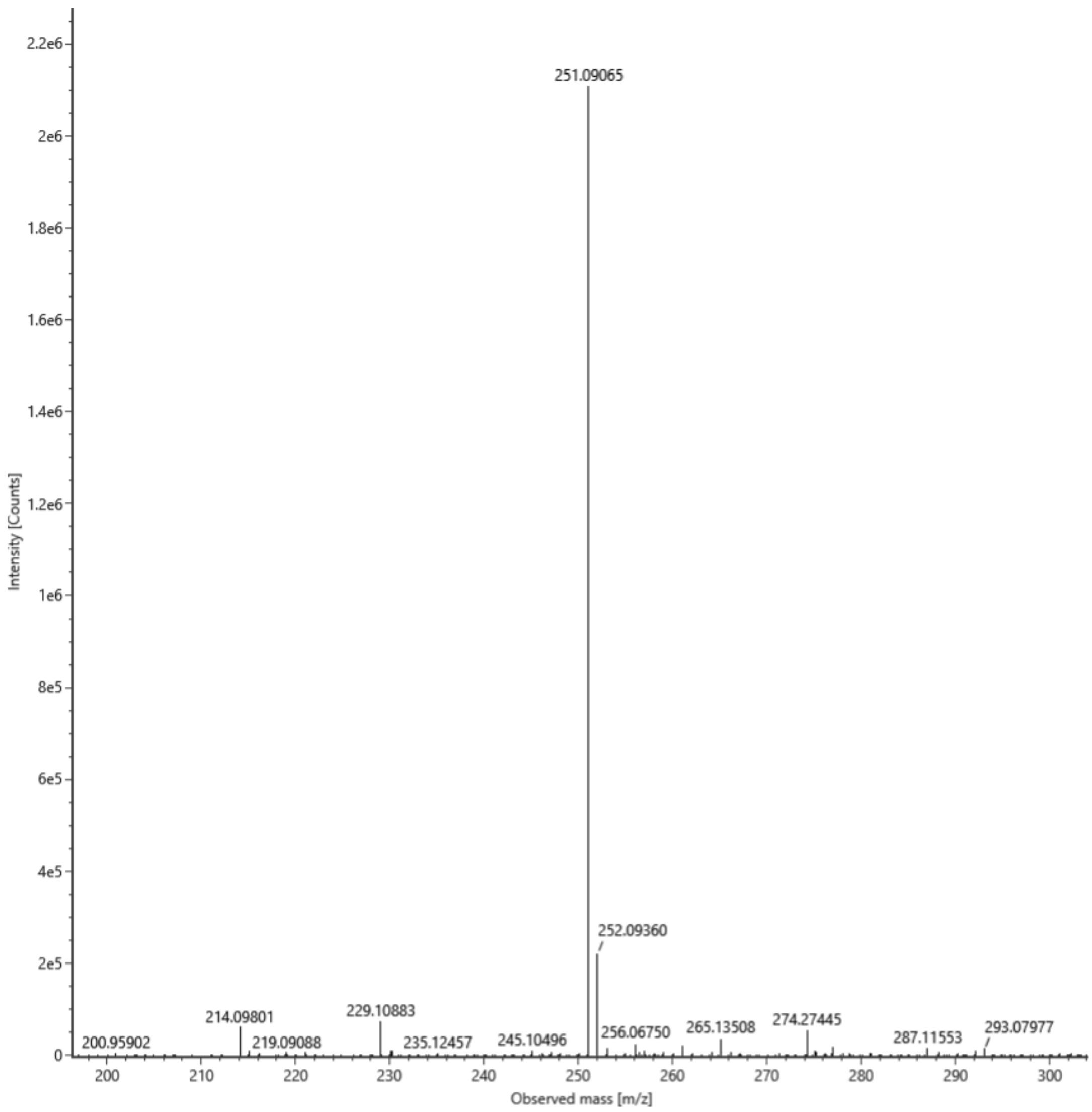

HRMS Spectrum of compound DK25

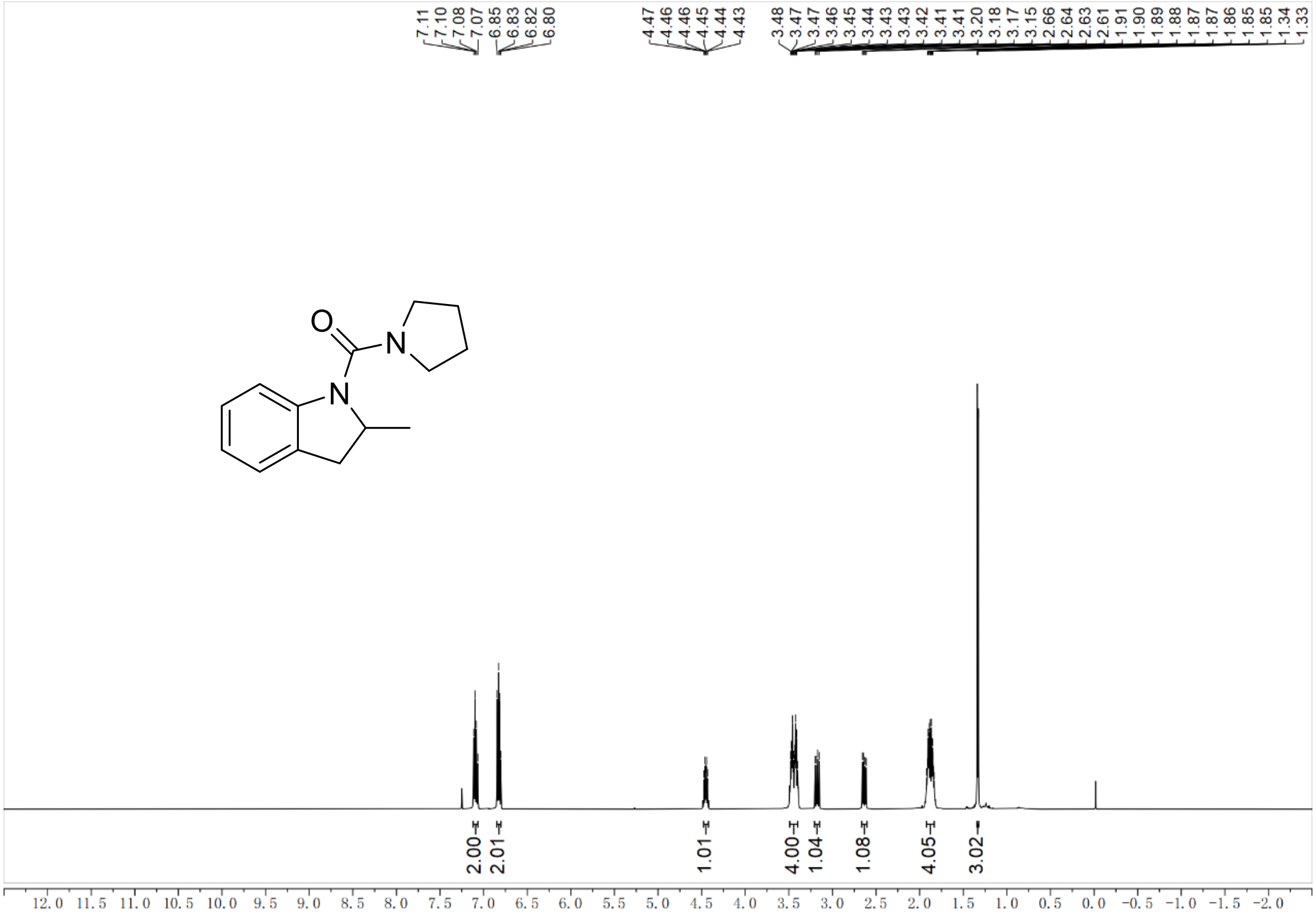

^{1}H NMR spectrum of compound DK26

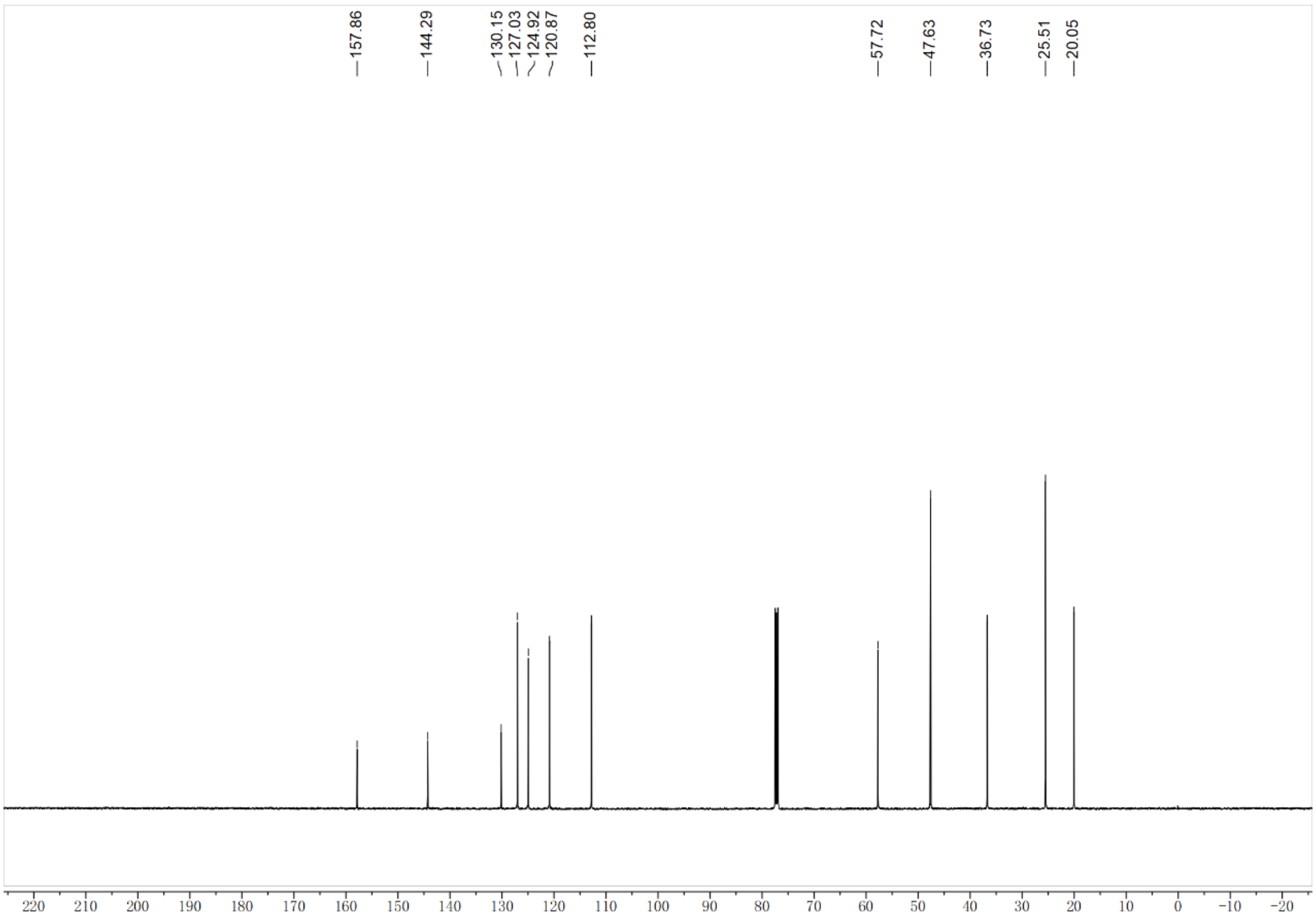

^{13}C NMR spectrum of compound DK26

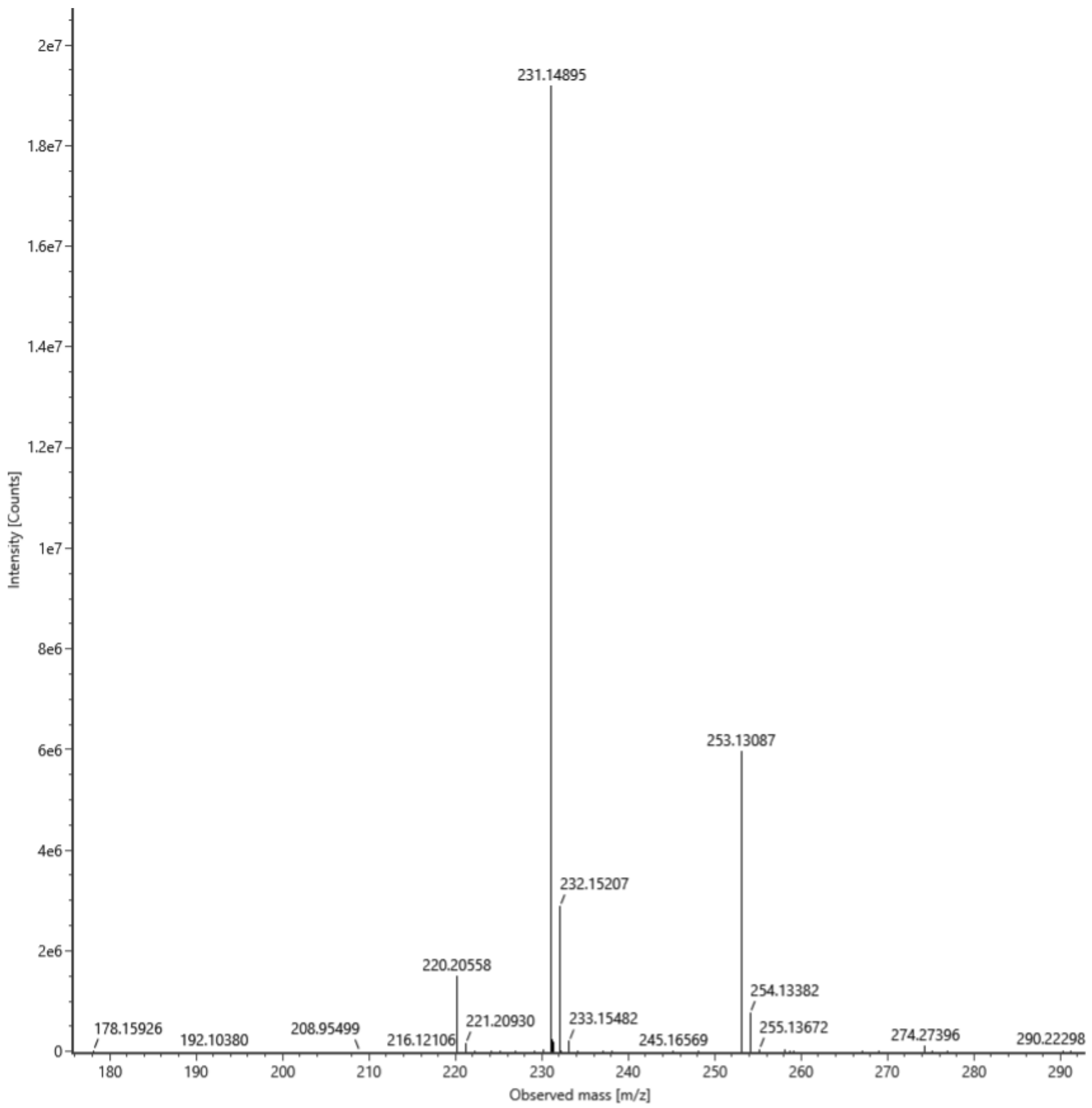

HRMS Spectrum of compound DK26

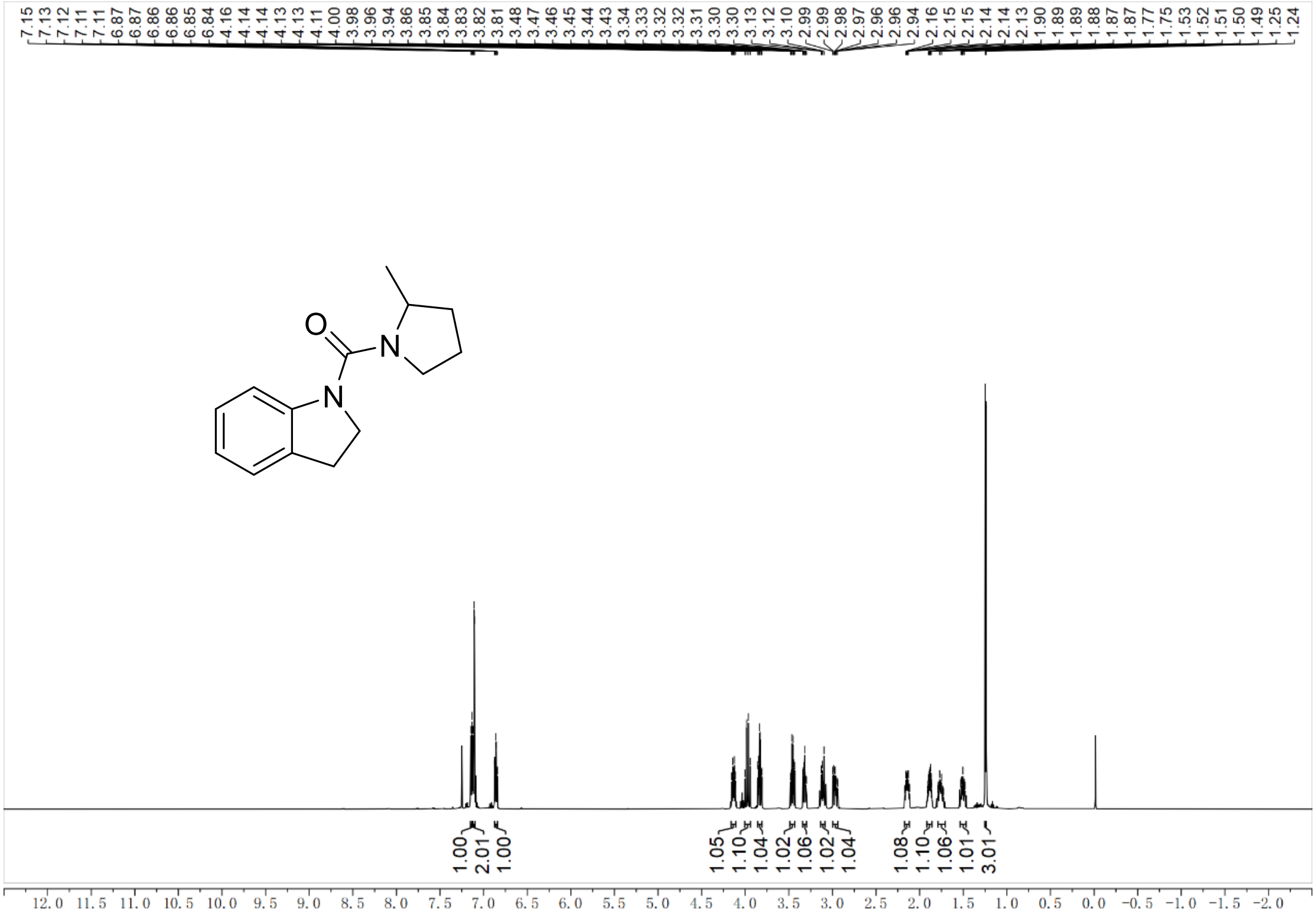

^{1}H NMR spectrum of compound DK27

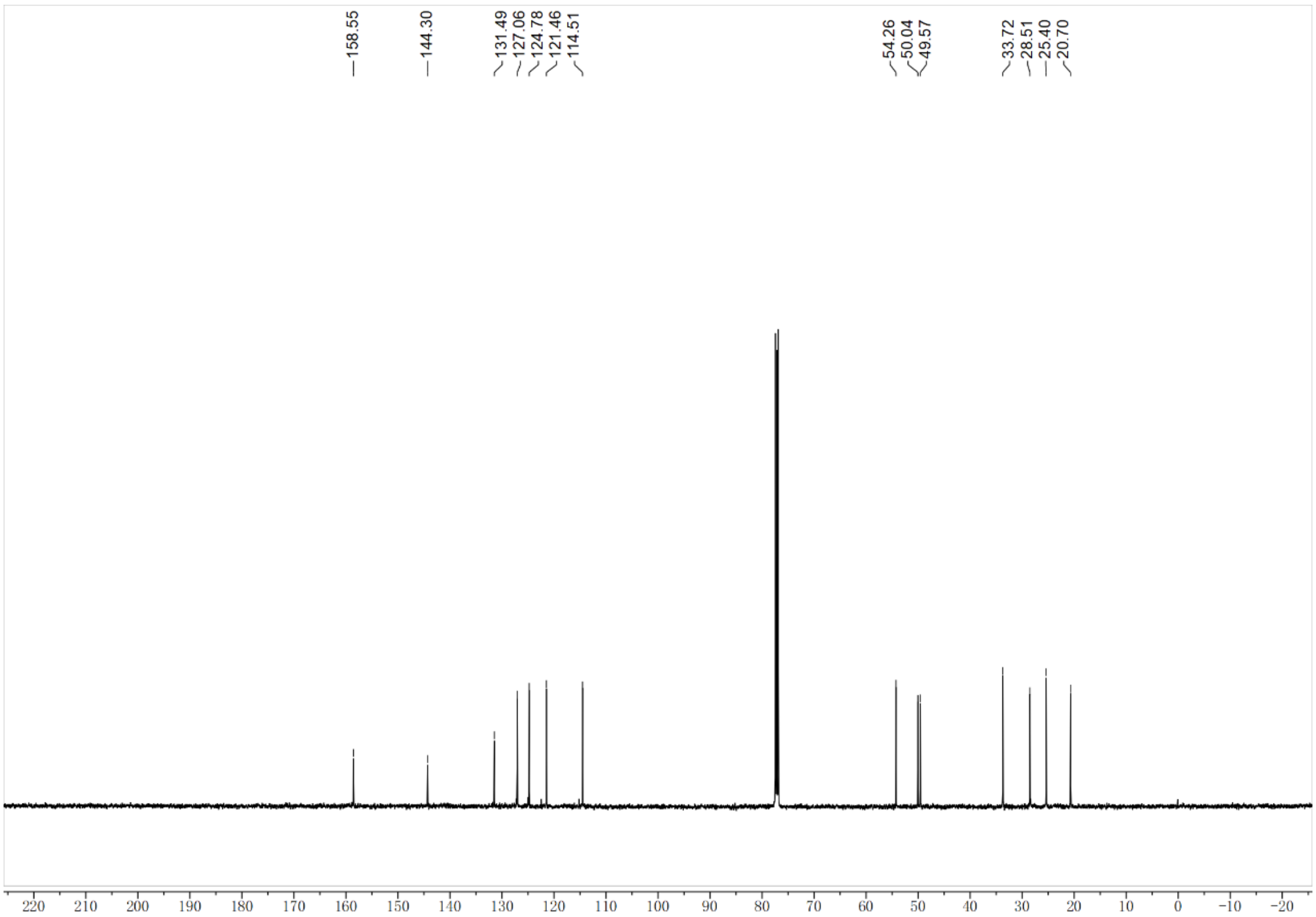

^{13}C NMR spectrum of compound DK27

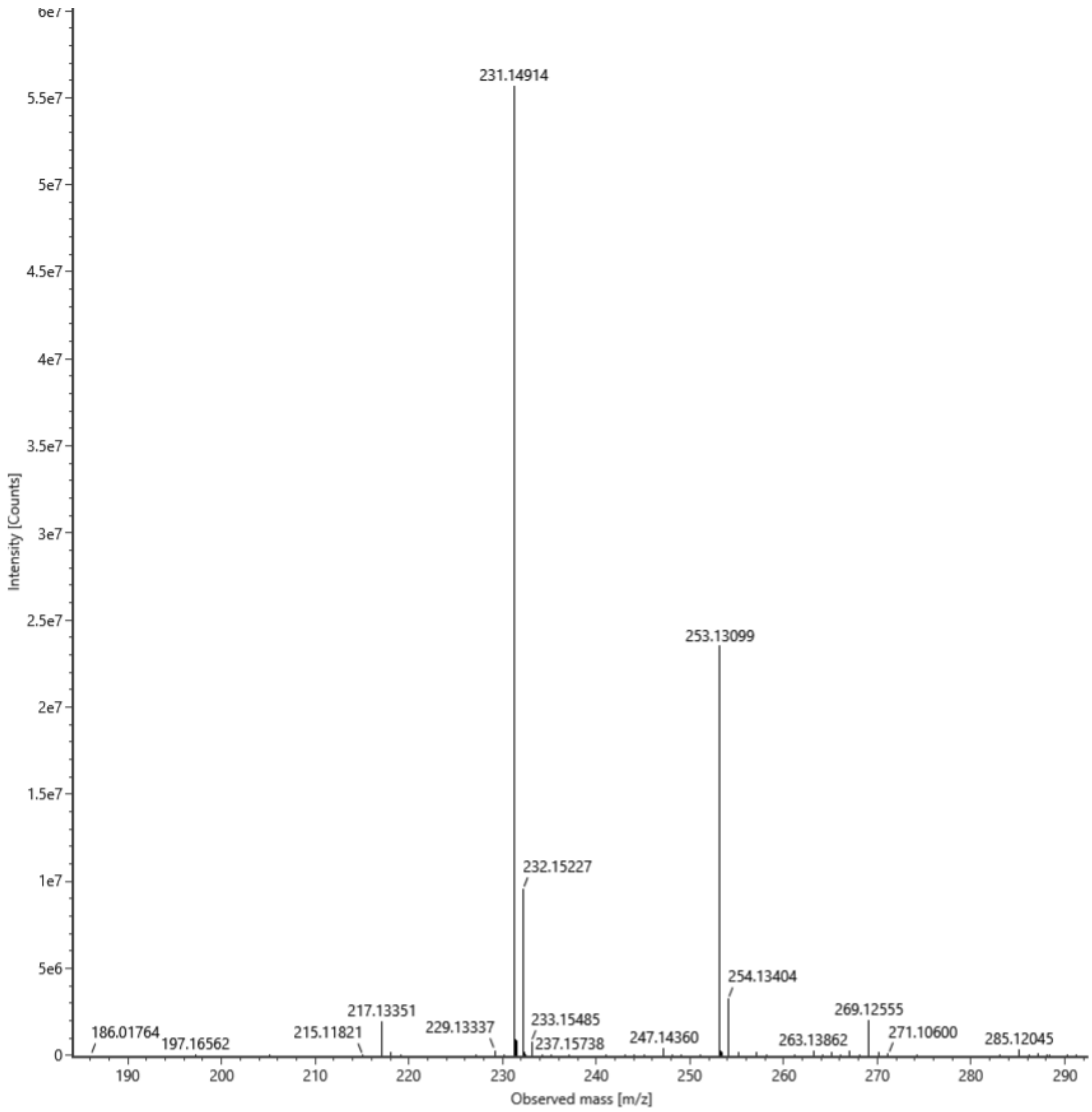

HRMS Spectrum of compound DK27

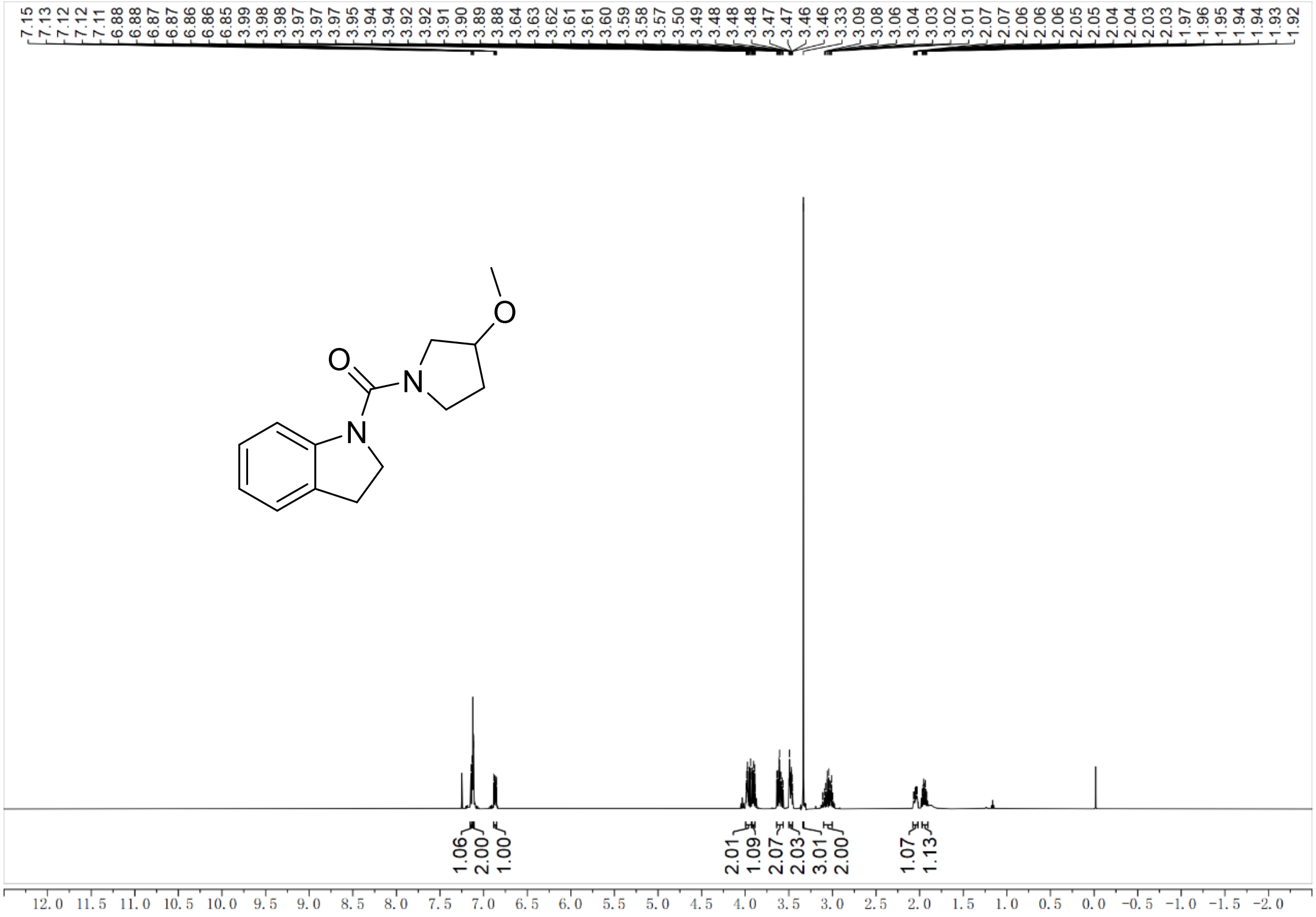

^{1}H NMR spectrum of compound DK28

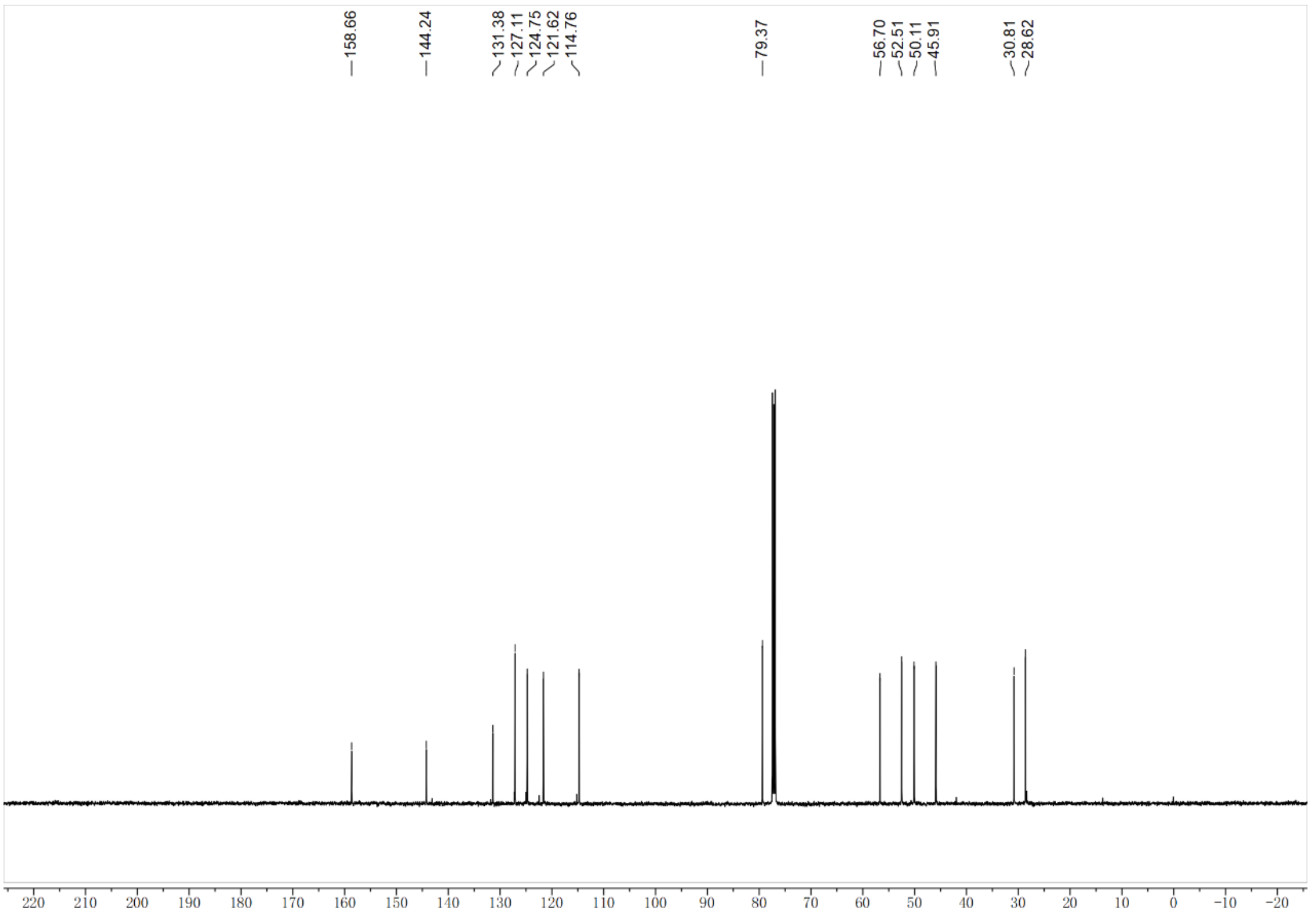

^{13}C NMR spectrum of compound DK28

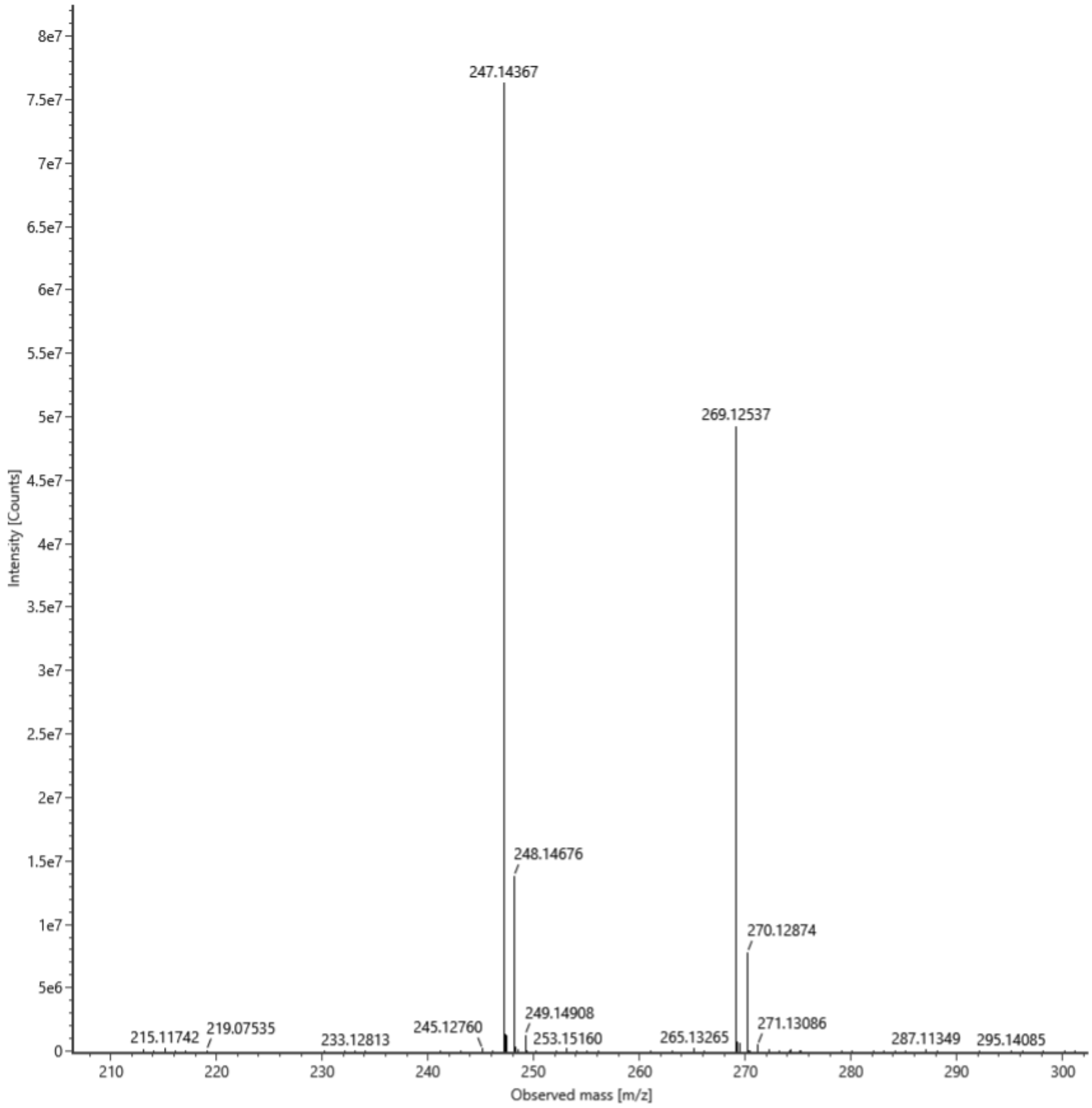

HRMS Spectrum of compound DK28

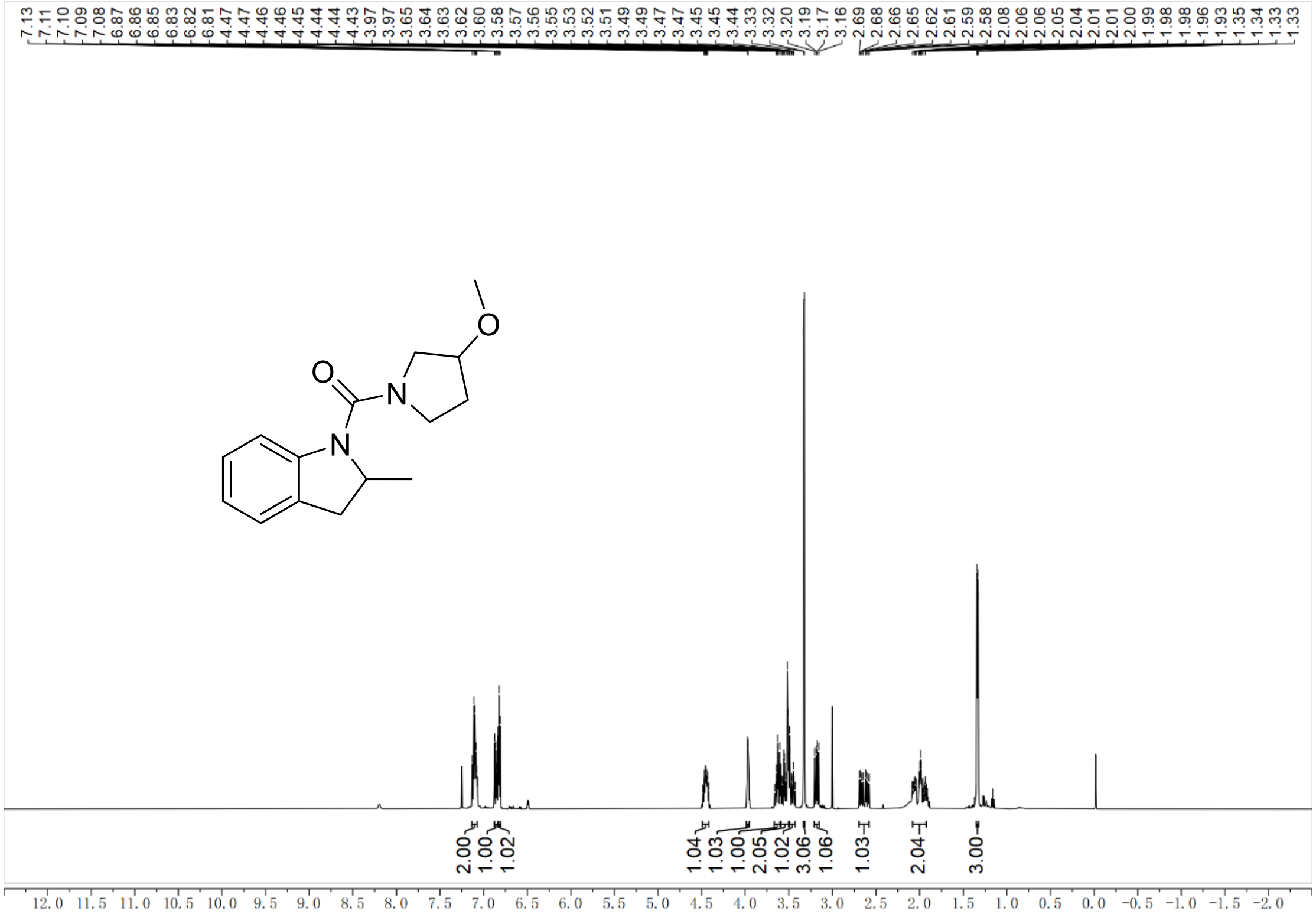

^{1}H NMR spectrum of compound DK29

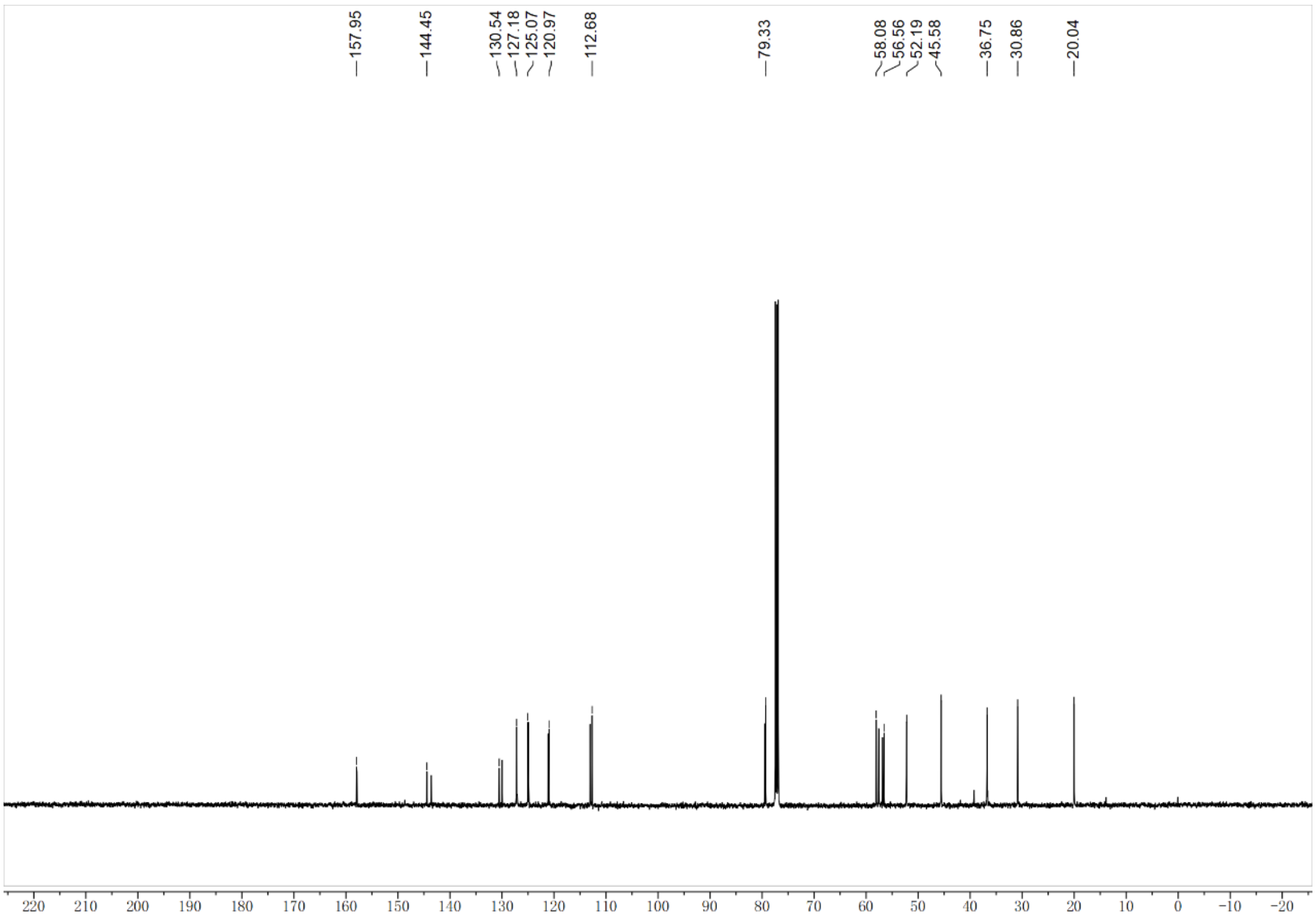

^{13}C NMR spectrum of compound DK29

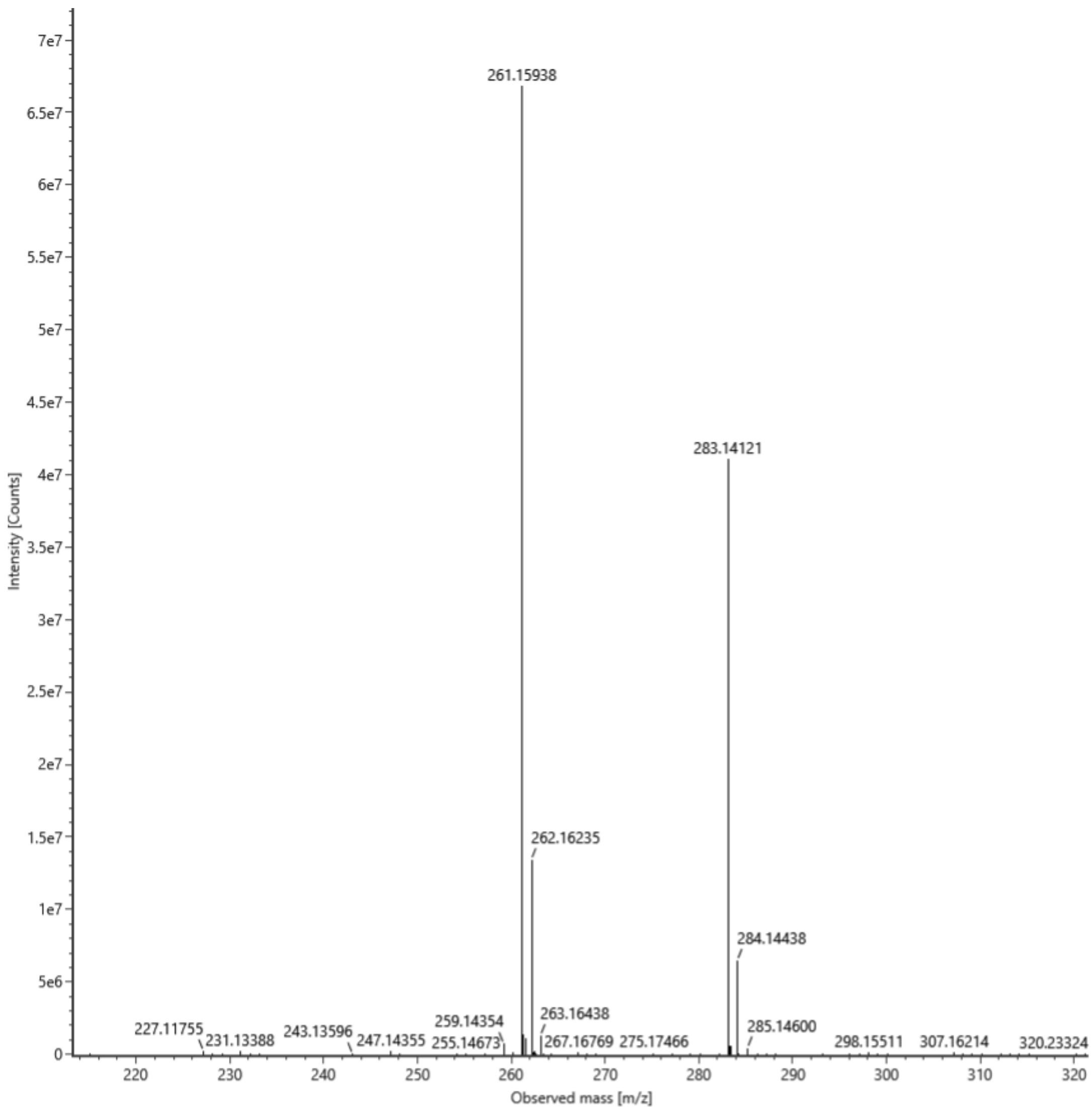

HRMS Spectrum of compound DK29

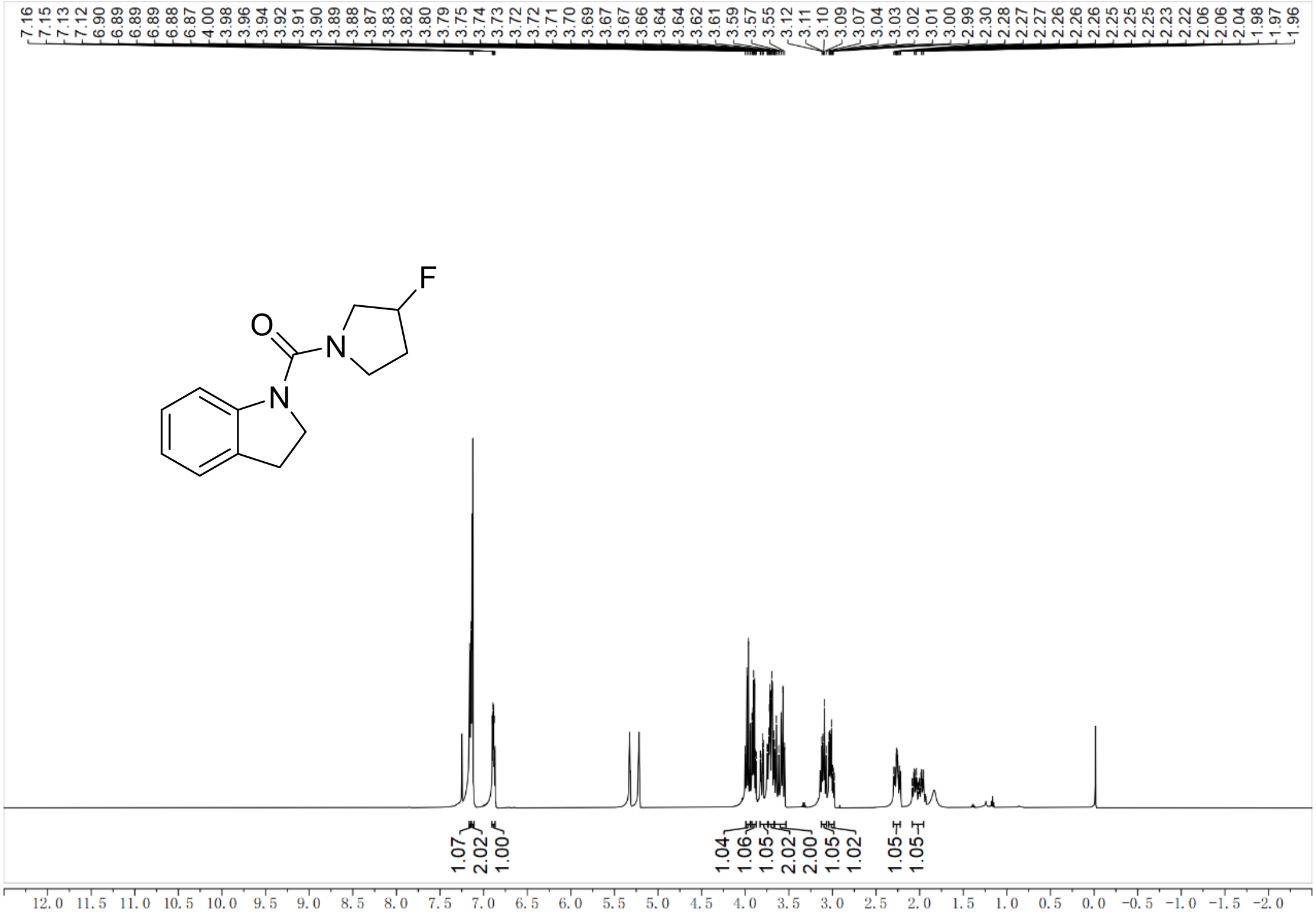

^{1}H NMR spectrum of compound DK30

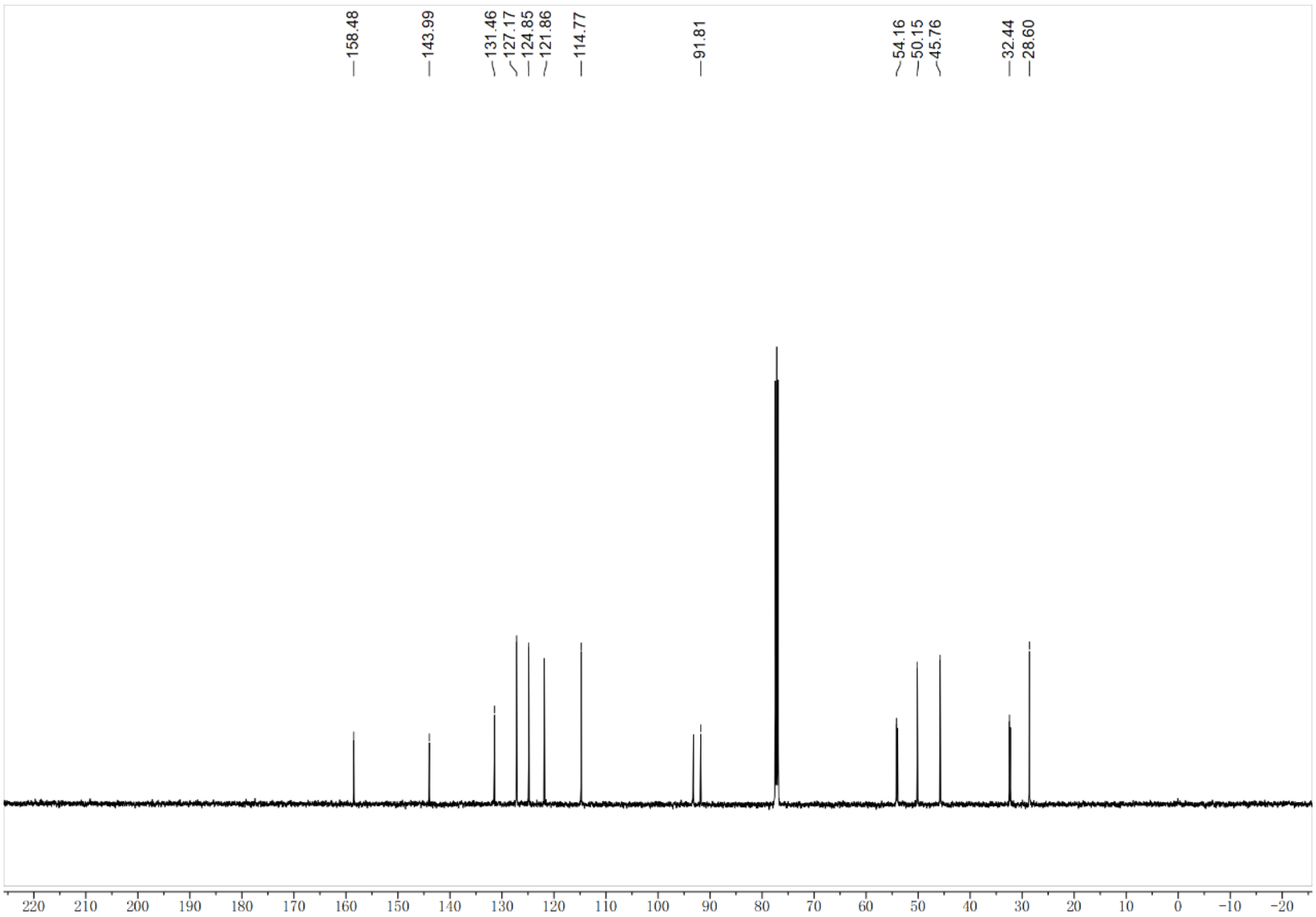

^{13}C NMR spectrum of compound DK30

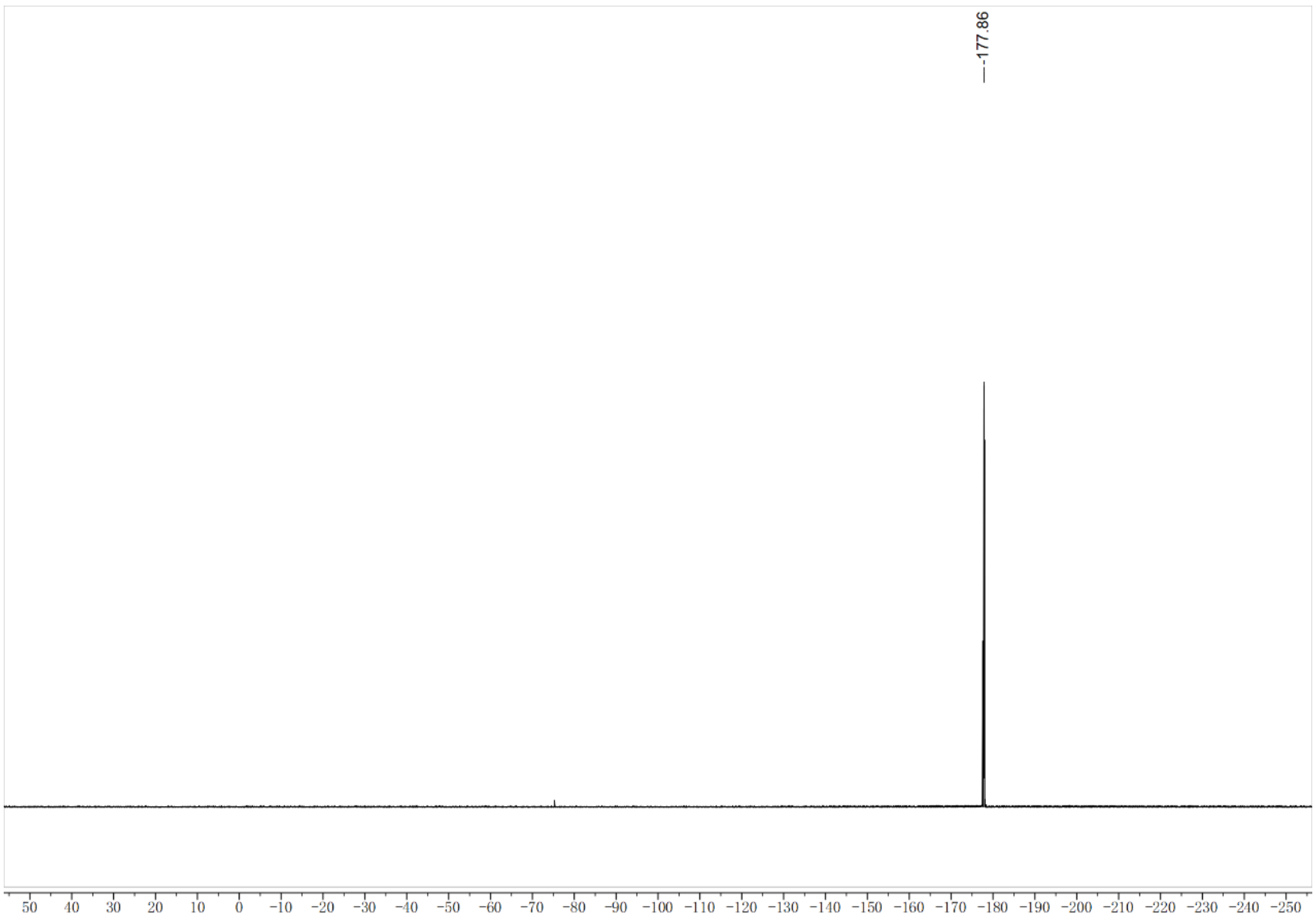

^{19}F NMR spectrum of compound DK30

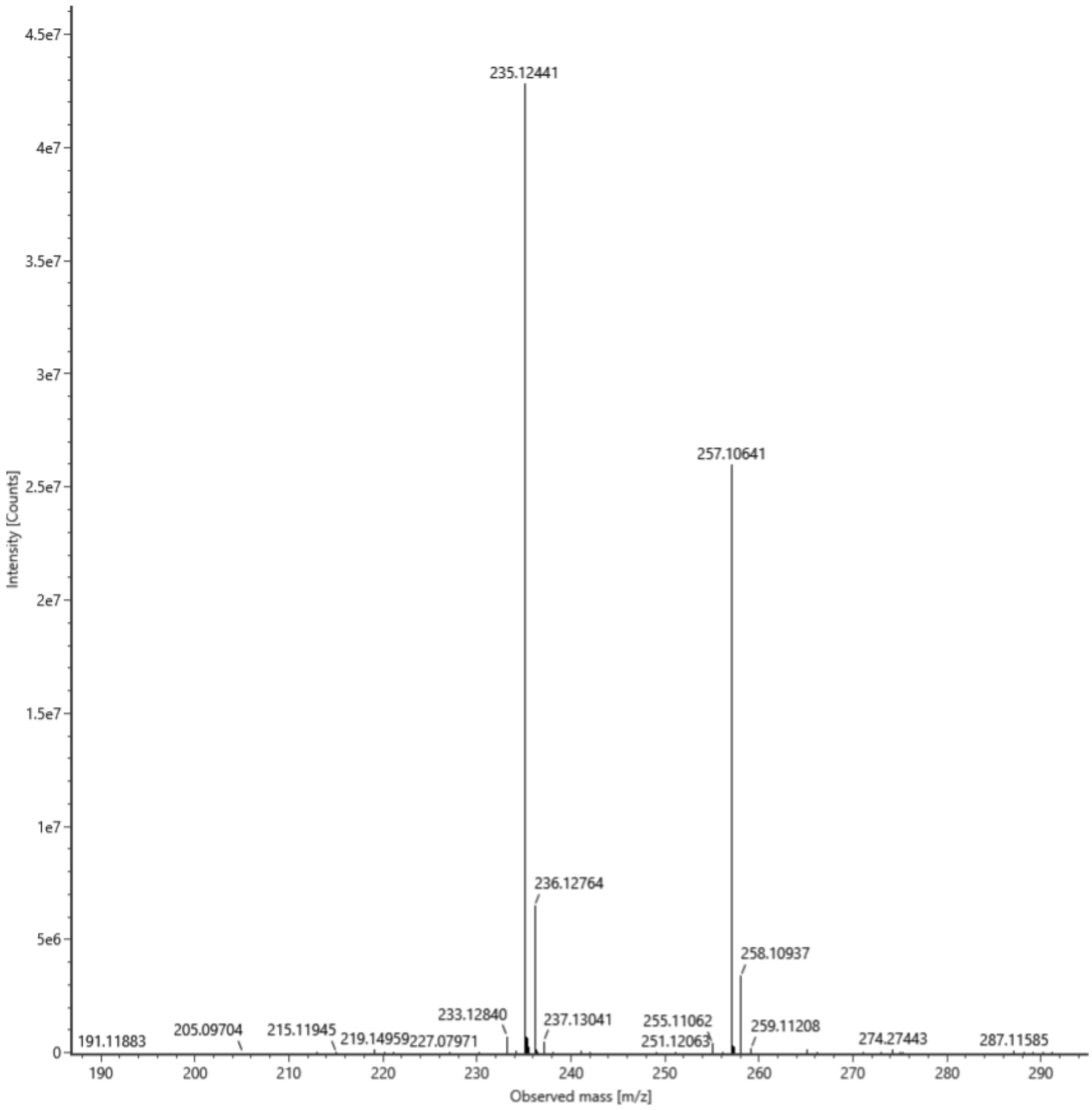

HRMS Spectrum of compound DK30

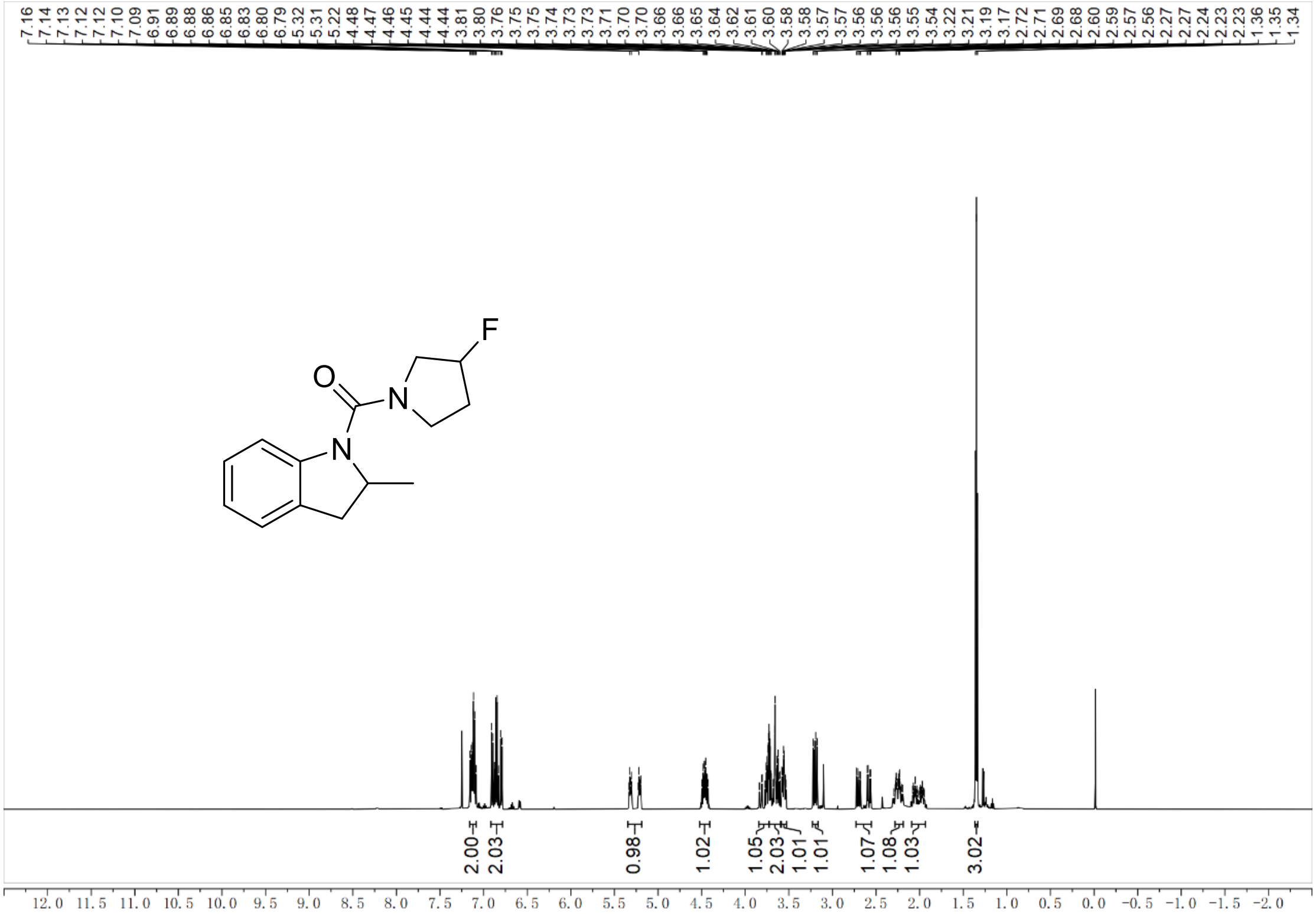

^{1}H NMR spectrum of compound DK31

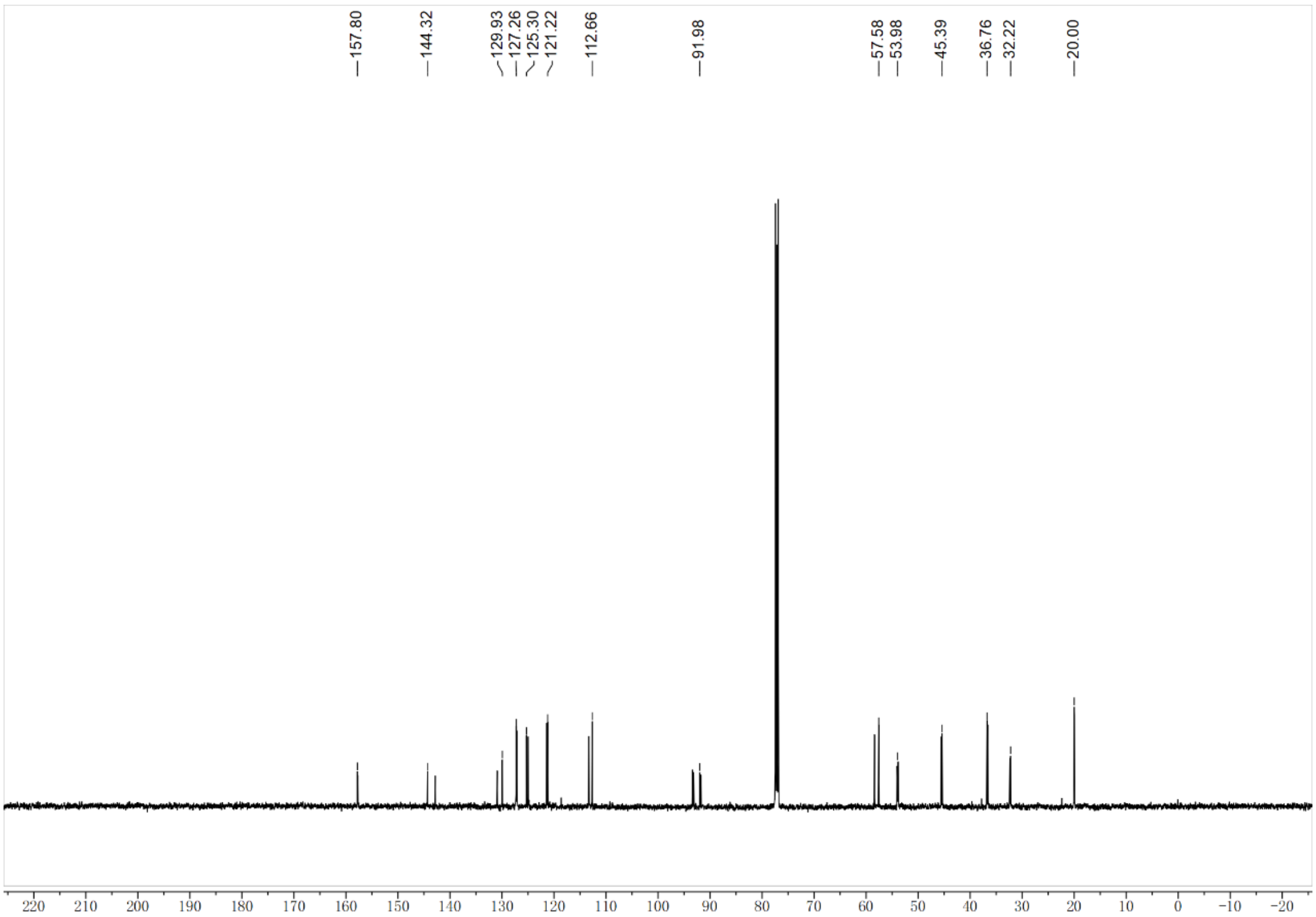

^{13}C NMR spectrum of compound DK31

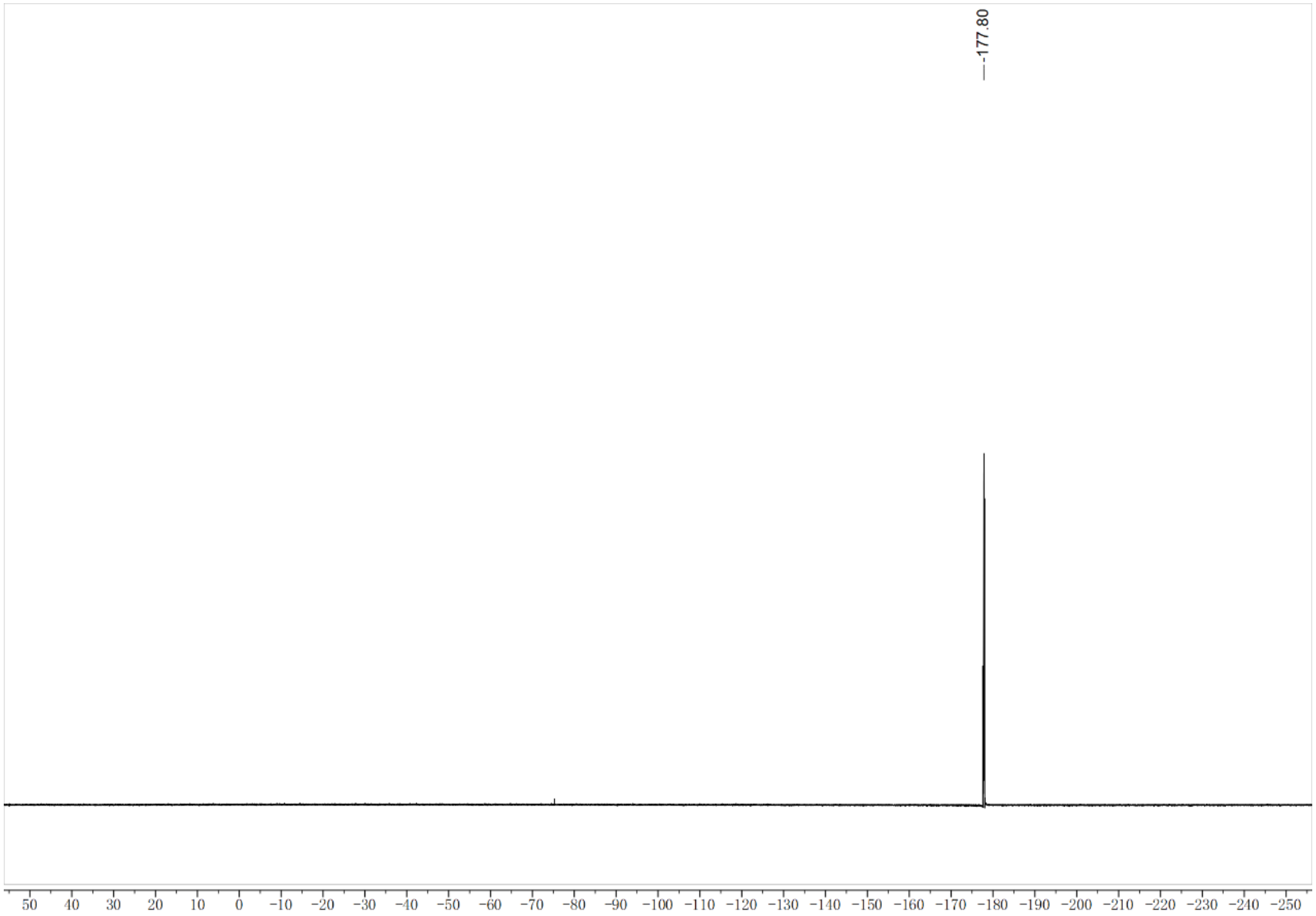

^{19}F NMR spectrum of compound DK31

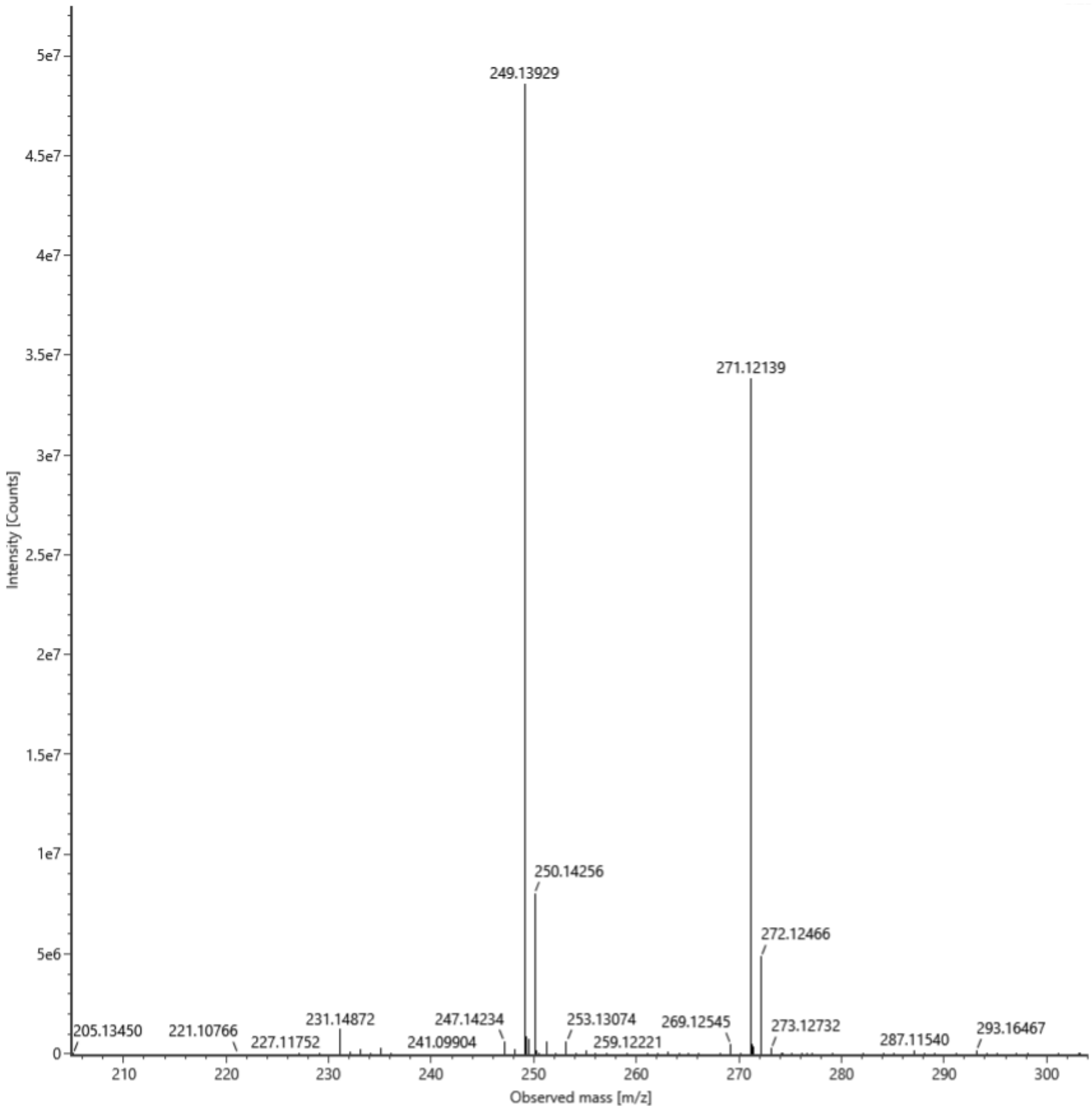

HRMS Spectrum of compound DK31

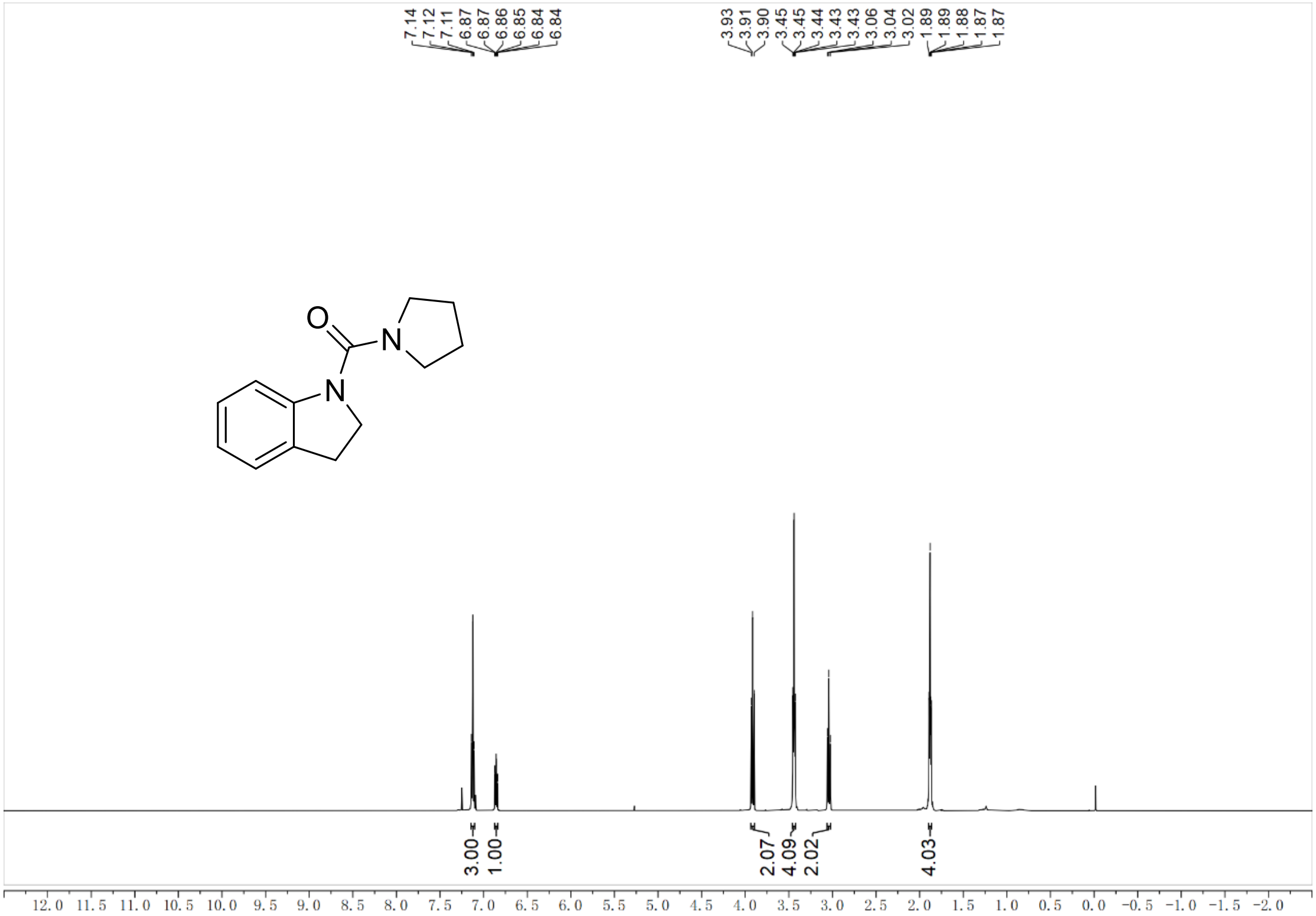

^{1}H NMR spectrum of compound DK32

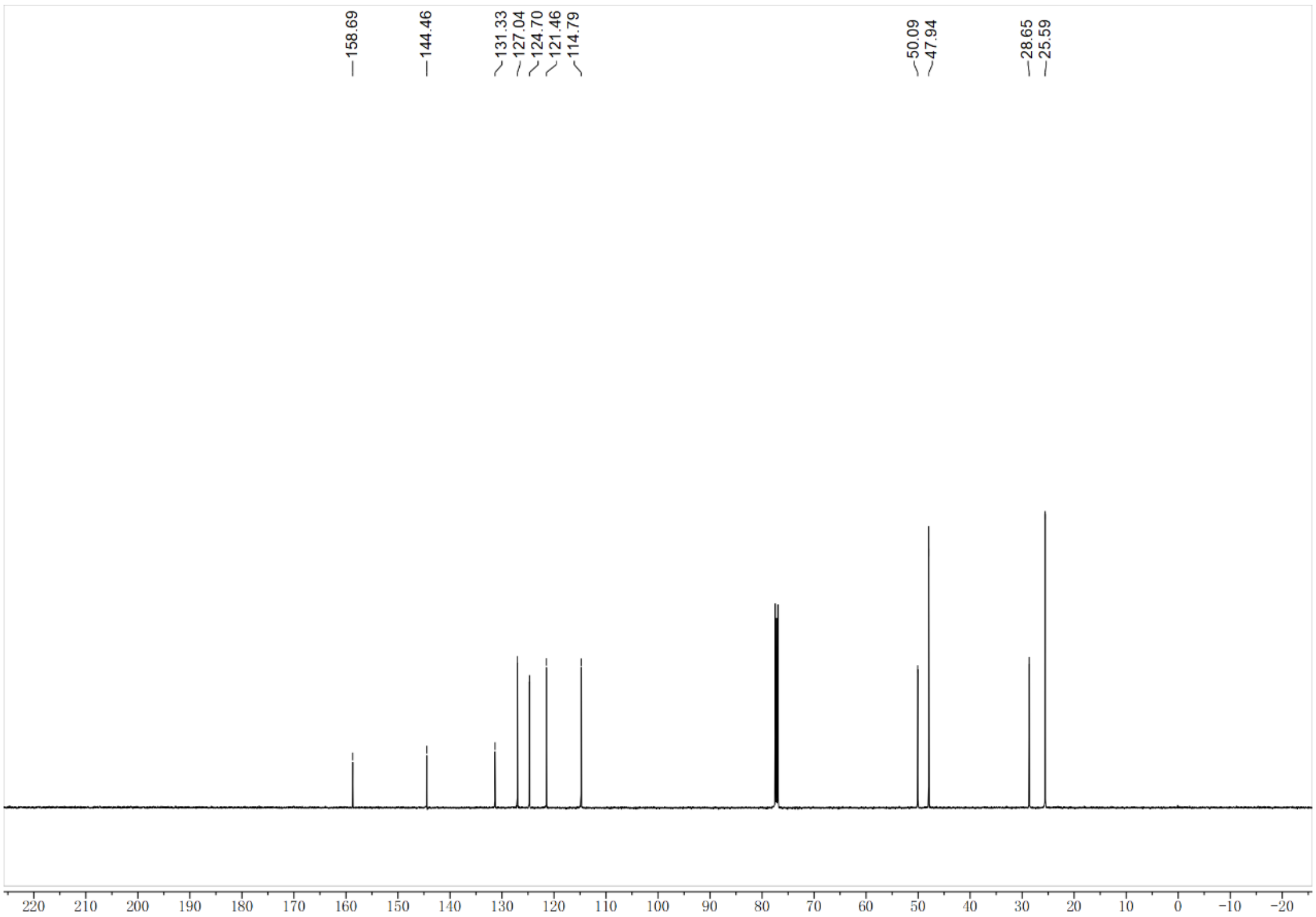

^{13}C NMR spectrum of compound DK32

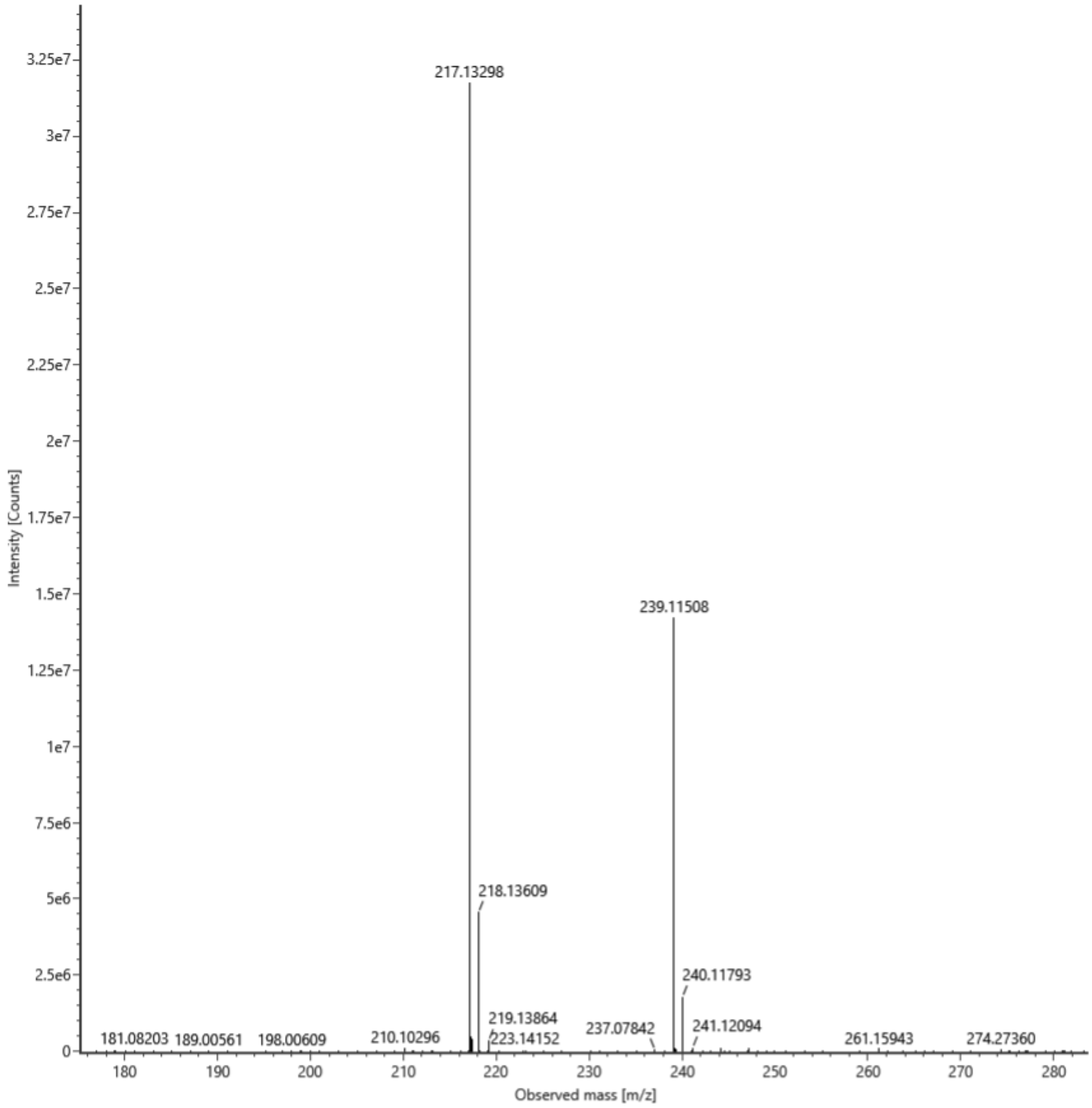

HRMS Spectrum of compound DK32

3. Effects of DK1-DK32 on rice plant height and dry weight

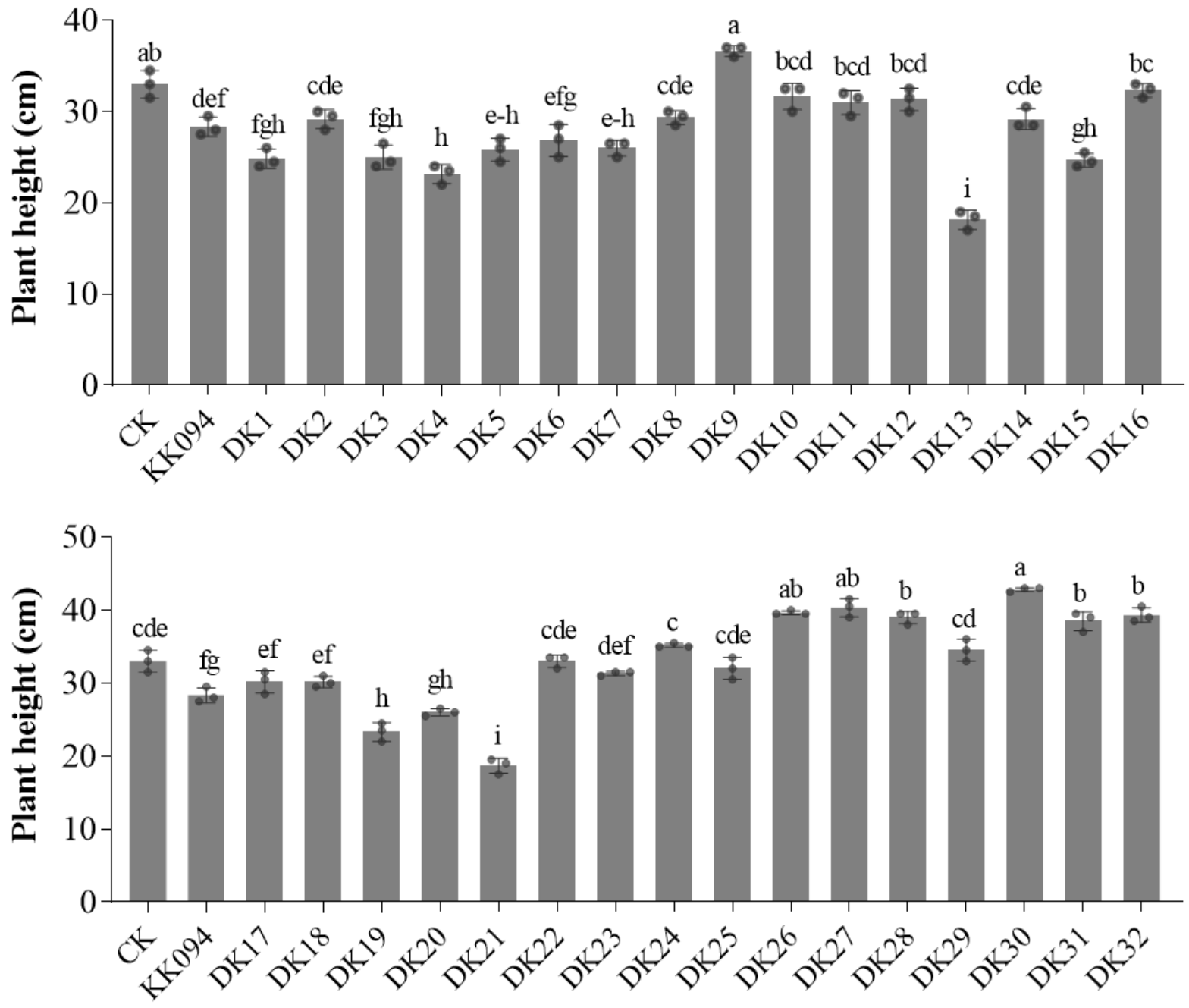

Supplementary Fig. 1 Effect of DK1-DK32 on rice plant height at 10 μM. The experiment was repeated three times showing similar results, with two samples per replicate. Error bars represent the mean ± standard deviation (SD). Statistical significance among means was determined using one-way ANOVA followed by Tukey's multiple comparison test. Different letters indicate statistically significant differences (n=3, $p < 0.05$). Specifically, for DK6, the significance levels compared with CK and KK094 were $p = 0.0001$ and $p = 0.9725$, respectively. Source data are provided as a Source Data file.

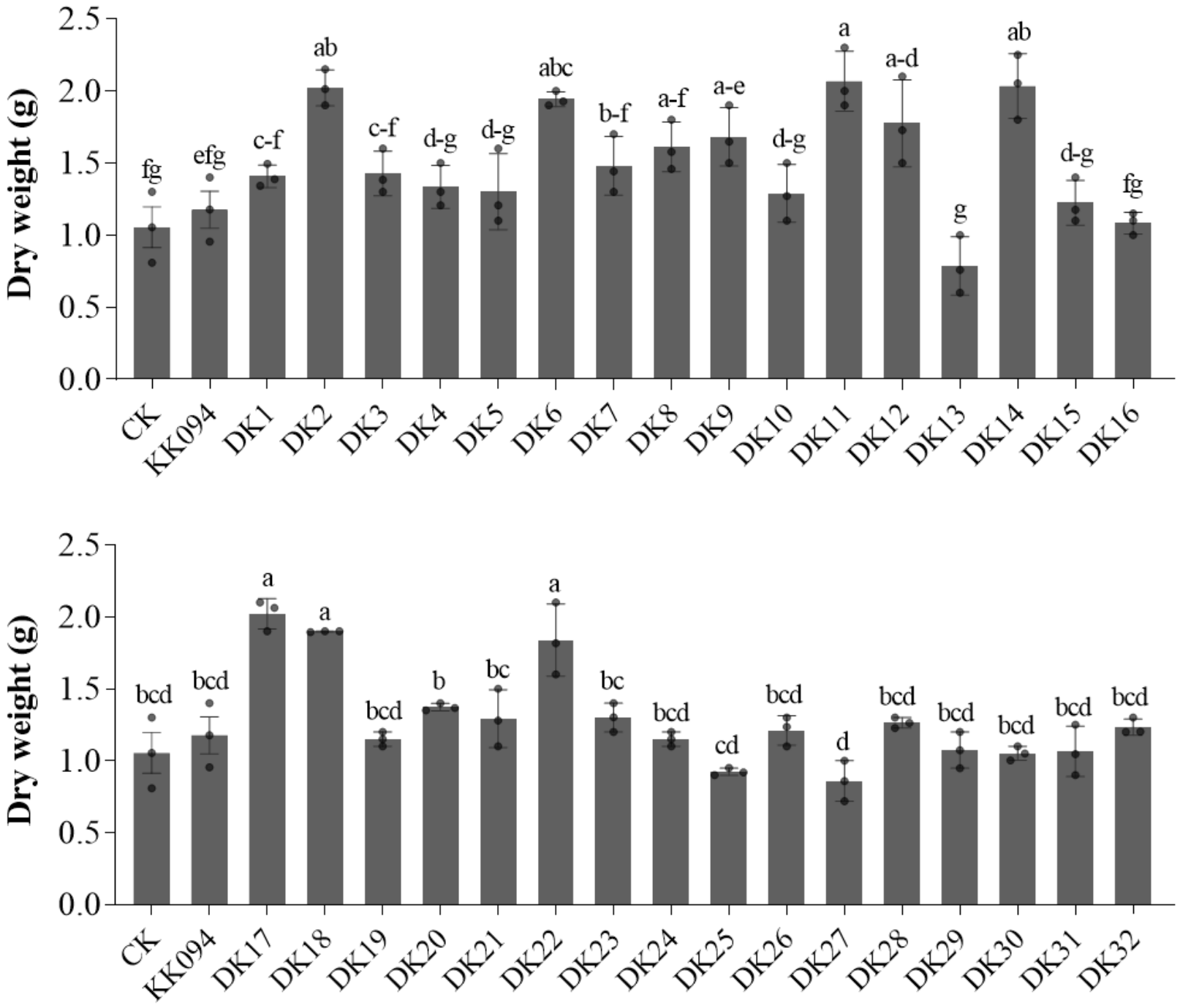

Supplementary Fig. 2 Effect of DK1-DK32 on rice dry weight at 10 µM. The experiment was repeated three times showing similar results, with two samples per replicate. Error bars represent the mean ± standard deviation (SD). Statistical signific ance among means was determined using one-way ANOVA followed by Tukey's multiple comparison test. Different letters indicate statistically significant differences (n=3, $p < 0.05$). Specifically, for DK6, the significance levels compared with CK and KK094 were $p = 0.0002$ and $p = 0.0023$, respectively. Source data are provided as a Source Data file.

4. Rice tillering-promoting activity of DK6 at different concentrations

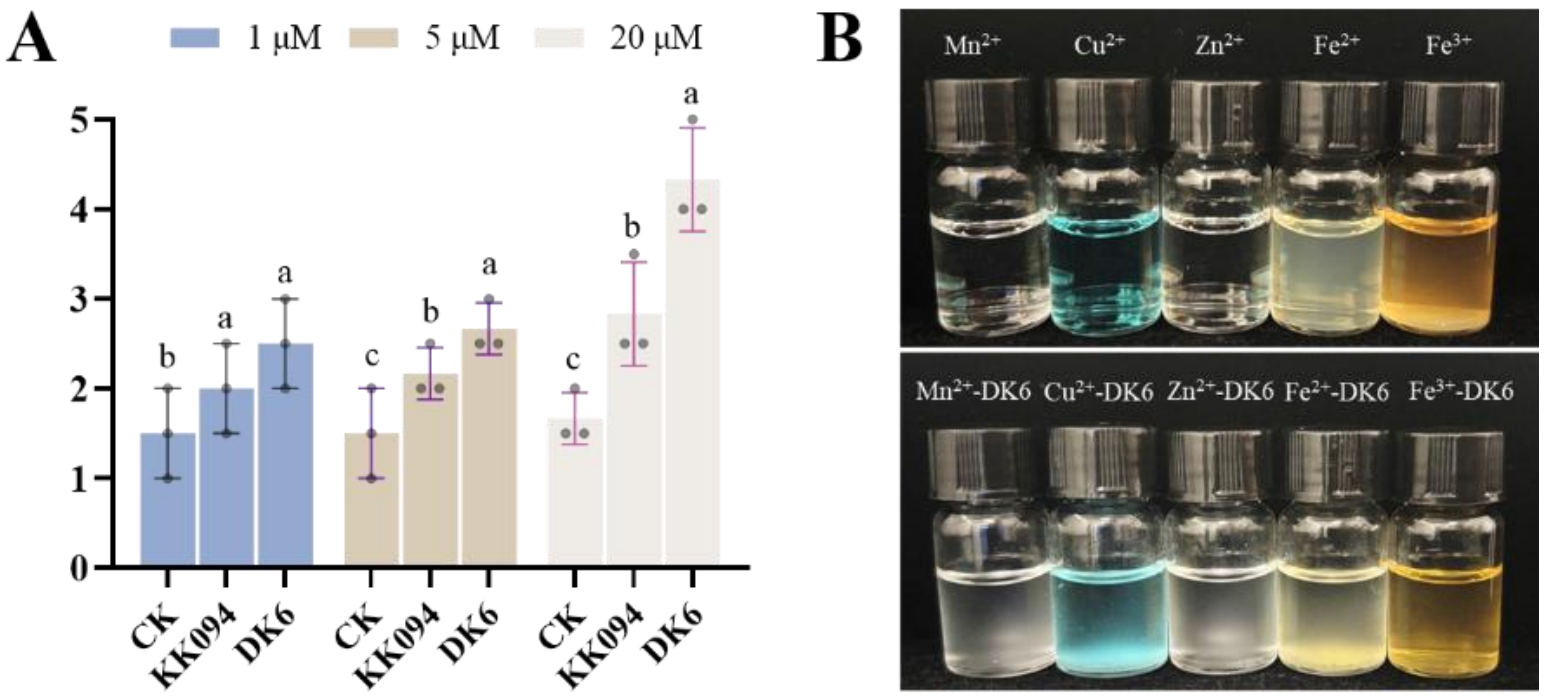

Supplementary Fig. 3 (A) The tillering-promoting activity of compound DK6 on rice at different concentrations and (B) the situation after compound DK6 is mixed with different metal ion solutions. For (A), error bars represent the mean ± standard deviation (SD), The experiment was repeated three times showing similar results (n=3), with two samples per replicate. Statistical significance among means was determined using one-way ANOVA followed by Tukey's multiple comparison test. Different letters indicate statistically significant differences ($p < 0.05$). Specifically, for DK6, compared with CK, the p-values at 1, 5, and 20 μM were 0.1089, 0.0202, and 0.0015, respectively; and compared with KK094, they were 0.4827, 0.3000, and 0.0242, respectively. Source data are provided as a Source Data file.

5. Digital photos of CMCS-DK6 swelling

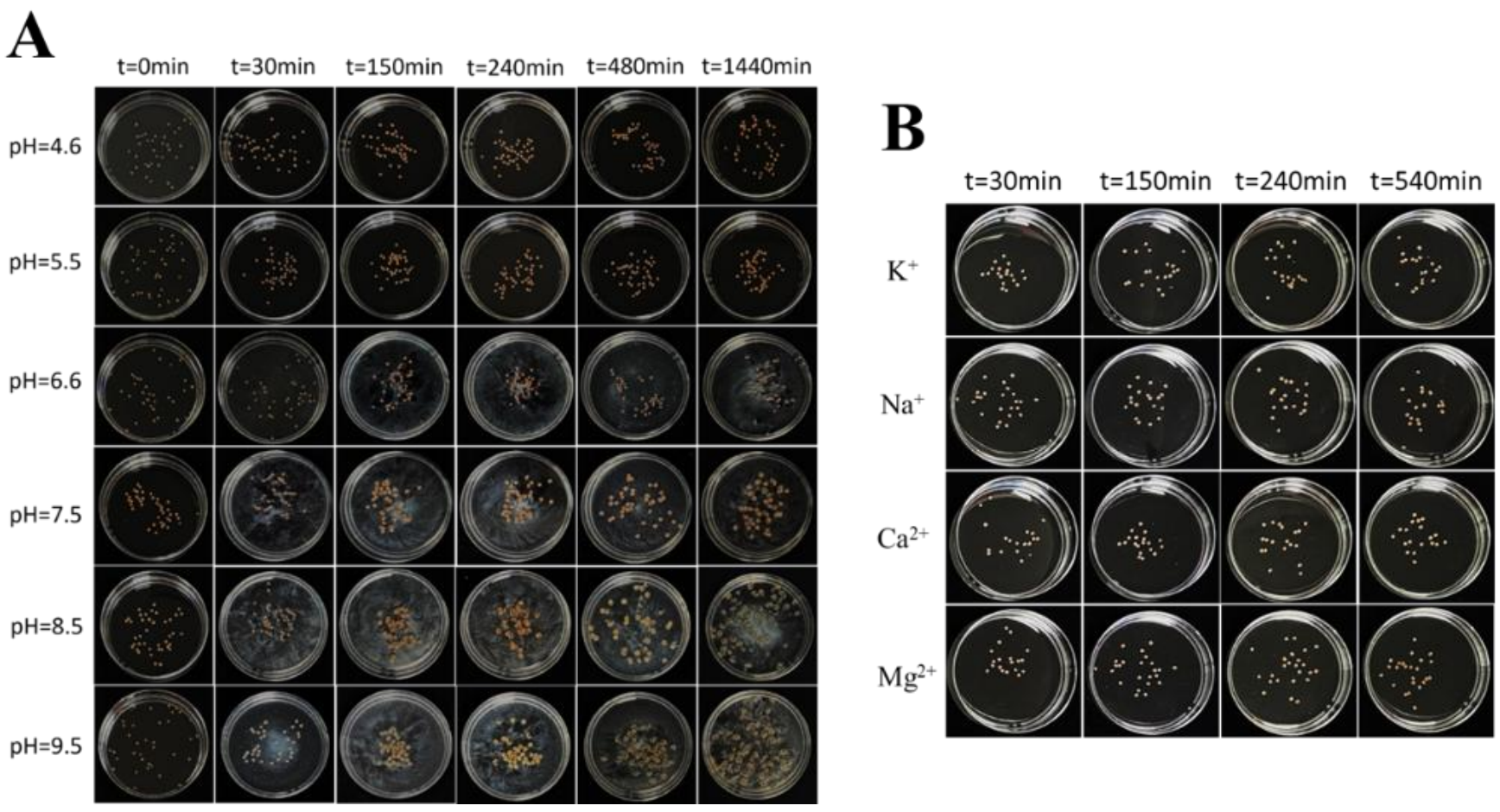

Supplementary Fig. 4 (A) Digital photos of CMCS-DK6 swelling in different pH solutions and (B) Digital photos of CMCS-DK6 swelling in different ionic solutions. The experiment was repeated three times showing similar results (n=3).

6. pH-dependent swelling reasonable mechanism of CMCS-DK6 hydrogel

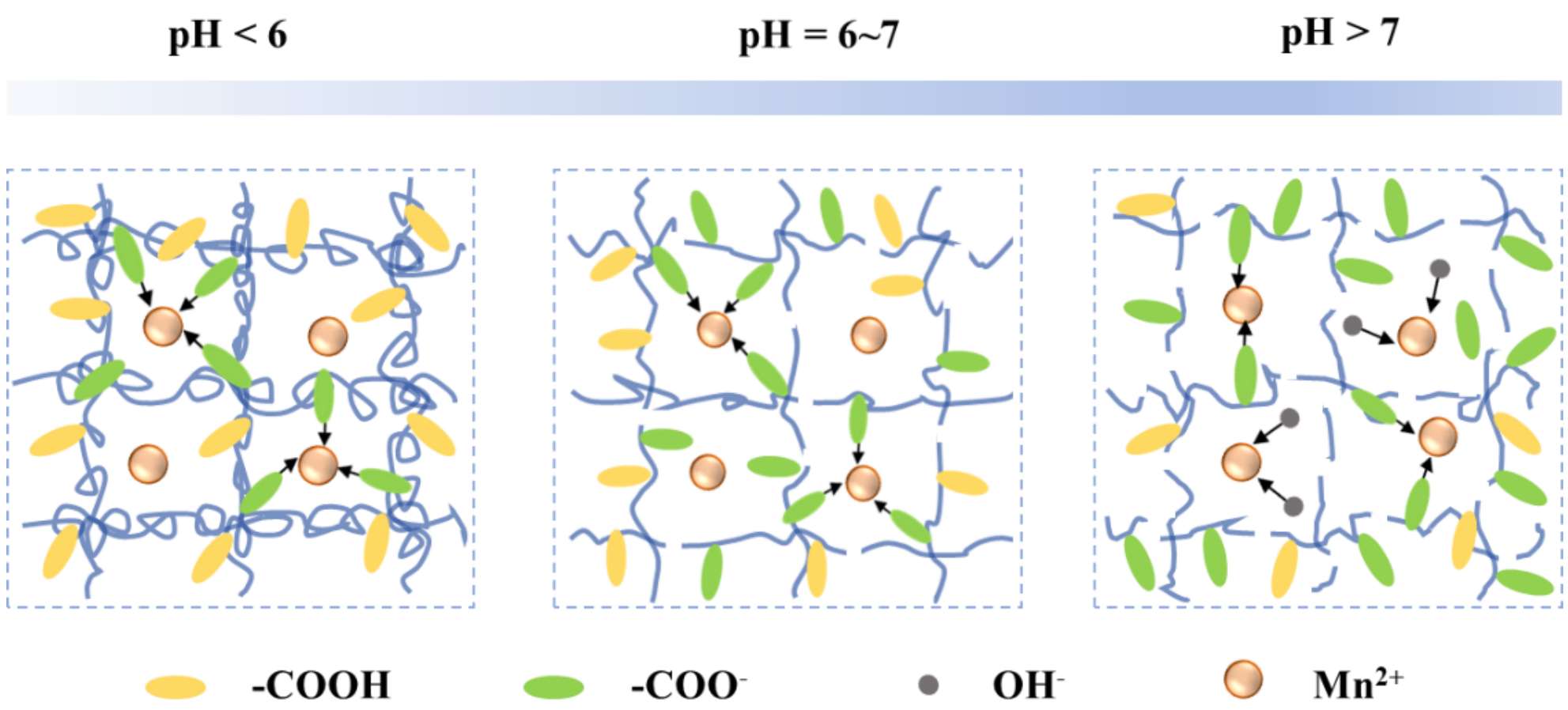

Supplementary Fig. 5 Reasonable mechanism of swelling behavior of CMCS-DK6 hydrogel in response to different pH.

7. Cumulative release and kinetics of CMCS-DK6 hydrogel in different soils

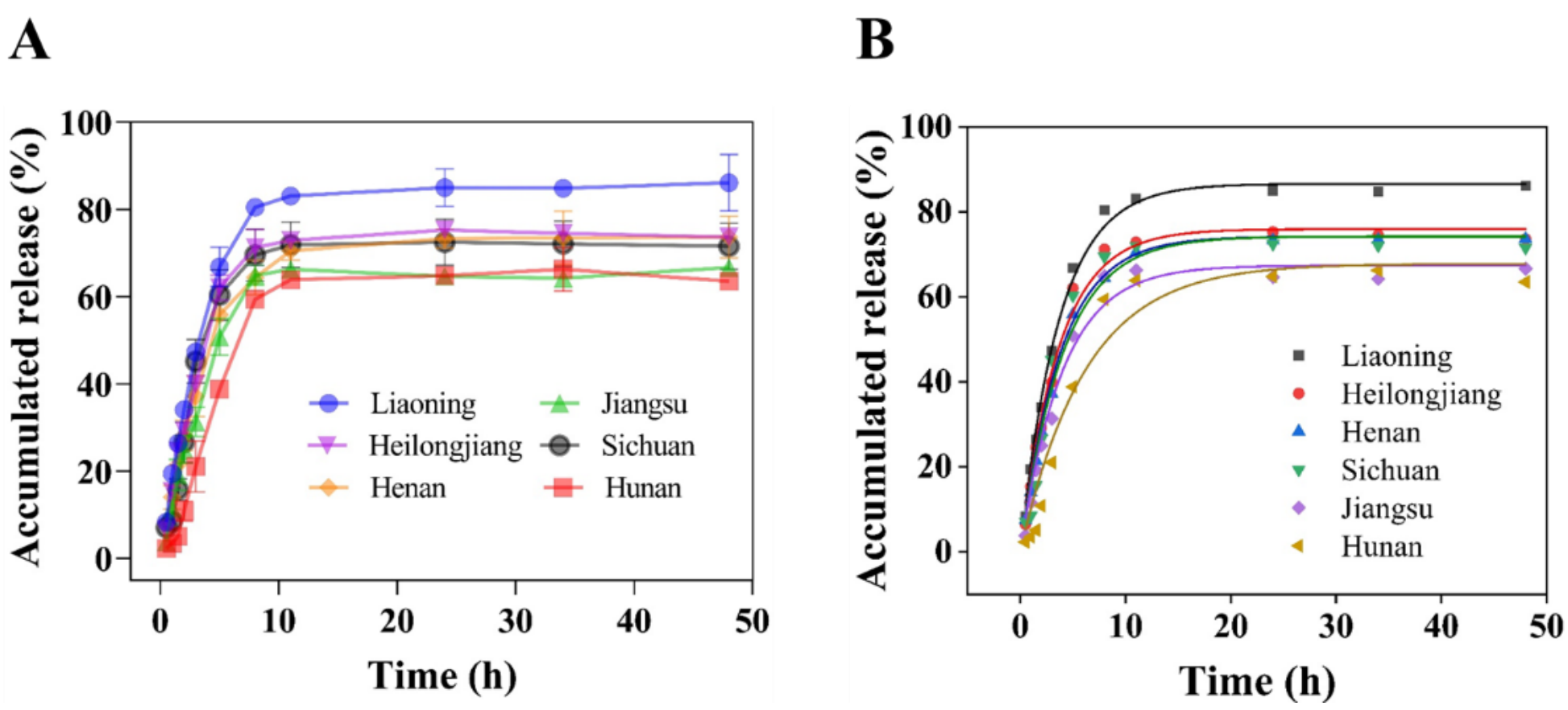

Supplementary Fig. 6 (A) Cumulative release profile of DK6 from the CMCS-DK6 hydrogel at different soil. (B) Fitting of the DK6 release profiles from CMCS-DK6 at different soil using a first-order kinetic model. Error bars represent the mean ± standard deviation (SD). The experiment was repeated three times showing similar results (n=3).

8. Determination of rice growth in different soils

Oryza sativa was used as the test plant, seeds were surface-sterilized by soaking in sodium hypochlorite solution. Six different soils were placed in equal amounts into planting pots. The soil in each pot was treated with various concentrations of CMCS-DK6 hydrogel sustained-release granules, including a blank control. The active ingredient of DK6 was applied at 20 mg and 40 mg per pot. Pre-germinated seeds (6 seeds per pot) were sown. All treatments were grown at 30 ± 1 °C, and rice growth was assessed after 45 days. The experiment was repeated three times.

9. Rice growth in different soils

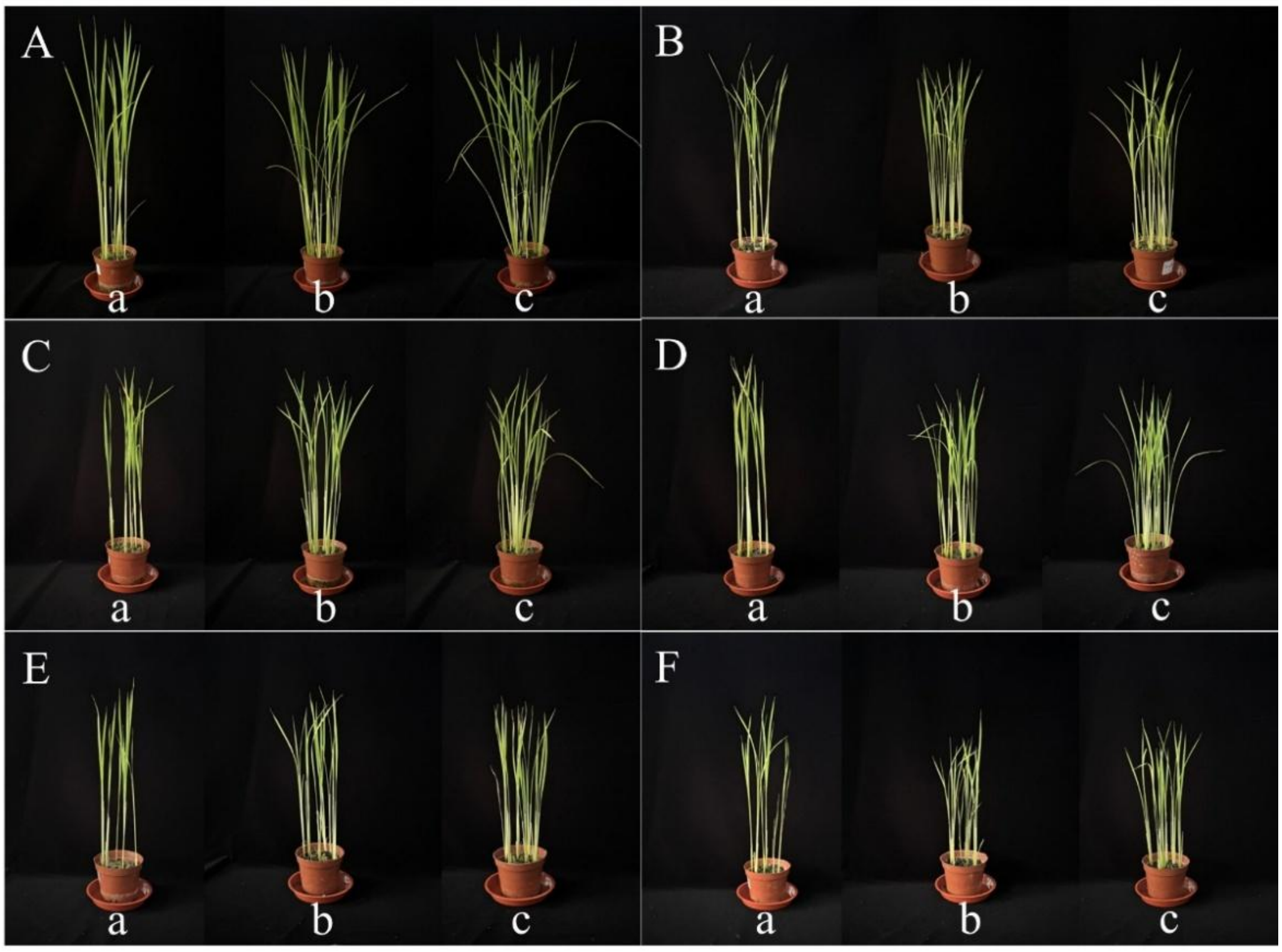

Supplementary Fig. 7 Rice growth observed after 45 days of cultivation in soils from Hunan (A), Jiangsu (B), Sichuan (C), Heilongjiang (D), Henan (E), and Liaoning (F), supplemented with different concentrations of CMCS-DK6 (a: 0 mg, b: 20 mg, c: 40 mg). The experiment was repeated three times showing similar results (n=3).

10.Kinetic fitting of cumulative release of CMCS-DK6 in different pH and soil

Supplementary Table 1 The release results of CMCS-DK6 hydrogel at different pH values and the correlation coefficient R^2 after fitting different models

pH	Zero-order		First-order		Higuchi		Ritger-Peppas		
	k	R^2	*k*	R^2	*k*	R^2	*k*	*n*	R^2
5.5	0.05730	0.75025	0.00172	0.91577	2.69648	0.88579	0.71757	0.65583	0.83889
6.6	0.06228	0.74622	0.00177	0.91691	2.93694	0.88550	0.82554	0.64850	0.83866
7.5	0.05708	0.67603	0.00301	0.97629	2.77602	0.86325	2.75079	0.49008	0.85698
8.5	0.04615	0.47244	0.00697	0.97597	2.37481	0.68627	12.18913	0.30189	0.77746

Supplementary Table 2 The release results of CMCS-DK6 hydrogel at different soil and the correlation coefficient R^2 after fitting different models

Different soil	Zero-order		First-order		Higuchi		Ritger-Peppas		
	k	R^2	*k*	R^2	*k*	R^2	*k*	*n*	R^2
Liaoning	1.35178	0.45546	0.26942	0.99420	12.03887	0.67403	32.62346	0.29042	0.77124
Heilongjiang	1.16659	0.42060	0.27262	0.98665	10.50267	0.64186	28.75341	0.28843	0.74285
Henan	1.20988	0.49574	0.24554	0.99591	10.66306	0.71246	26.34009	0.30069	0.79750
Sichuan	1.17055	0.39207	0.26203	0.96242	10.59969	0.60978	26.82838	0.30067	0.70296
Jiangsu	1.08527	0.43120	0.24848	0.97643	9.71728	0.64851	23.78849	0.30658	0.73609
Hunan	1.30942	0.50426	0.16105	0.94130	11.47235	0.71420	16.52492	0.40170	0.74435

11. Construction and purification of *At*D14 protein expression vector

Construction of AtD14 protein expression vector: Total RNA was extracted from wild-type Arabidopsis thaliana and reverse-transcribed into cDNA. PCR amplification was performed using primers containing the target vector sequences. The amplified product was confirmed by agarose gel electrophoresis, purified, and recovered. The pET28a vector was double-digested with EcoRI and XhoI, and the target gene fragment was ligated into the vector. The recombinant plasmid was transformed into E. coli DE3 pLys competent cells and verified by PCR and sequencing. Primer sequences:

F: CCGGAATTCATGAGTCAACACAACATC

R: CCGCTCGAGAAGTCACCGAGGAAGAGC

Expression and purification of AtD14 protein: A single colony of the expression strain was inoculated into 5 mL LB medium containing kanamycin (1:1000 dilution) and cultured at 37°C, 180 rpm for 8-10 h. The culture was then transferred to 150 mL LB medium with kanamycin and incubated at 180 rpm for an additional 1-2 h. Protein expression was induced with 0.2 mM IPTG at 18°C, 180 rpm overnight. Cells were harvested by centrifugation at 2390 g for 10 min, and the pellet was resuspended in 1 mL binding buffer. The suspension was sonicated on ice for 20 min (30% power, 2 s on/3 s off) until clear. After centrifugation at 12,000 g for 15 min at 4°C, the supernatant was collected. A column was loaded with 600-700 µL Ni-NTA resin, washed with water and binding buffer (twice each). The supernatant was passed through the column, followed by two washes with 10 mL binding buffer. The bound protein was eluted sequentially with 1 mL each of elution buffers containing 50 mM, 100 mM, and 150 mM imidazole, collecting 1-2 drops per fraction to monitor protein concentration. The target protein fractions were pooled. The column was washed twice with binding buffer, once with water, and stored in 20% ethanol (reusable for 2-3 times). The purified protein was analyzed by SDS-PAGE for quality and concentration determination.

12.Hydrogel nutrient function

This study used *Oryza sativa* as the test material, seeds were surface-sterilized by soaking in sodium hypochlorite solution. Equal amounts of nutrient soil were placed into planting pots, and the soil was treated with DK6 or CMCS-DK6 hydrogel slow-release granules, with a blank control included. Each pot received 20 mg of DK6 active ingredient and was sown with six seeds. All pots were placed in a growth chamber under conditions of (30 ± 1)°C with a 16 h light/8 h dark photoperiod. The day when rice seeds in all treatments germinated was recorded as day 1. On day 5 after emergence, seedlings were carefully removed from the soil, rinsed, and blotted dry with filter paper. Fresh weight and shoot height were then measured to evaluate the effects of the hydrogel granules on rice seedling growth.

13.Photographs of rice with different treatment

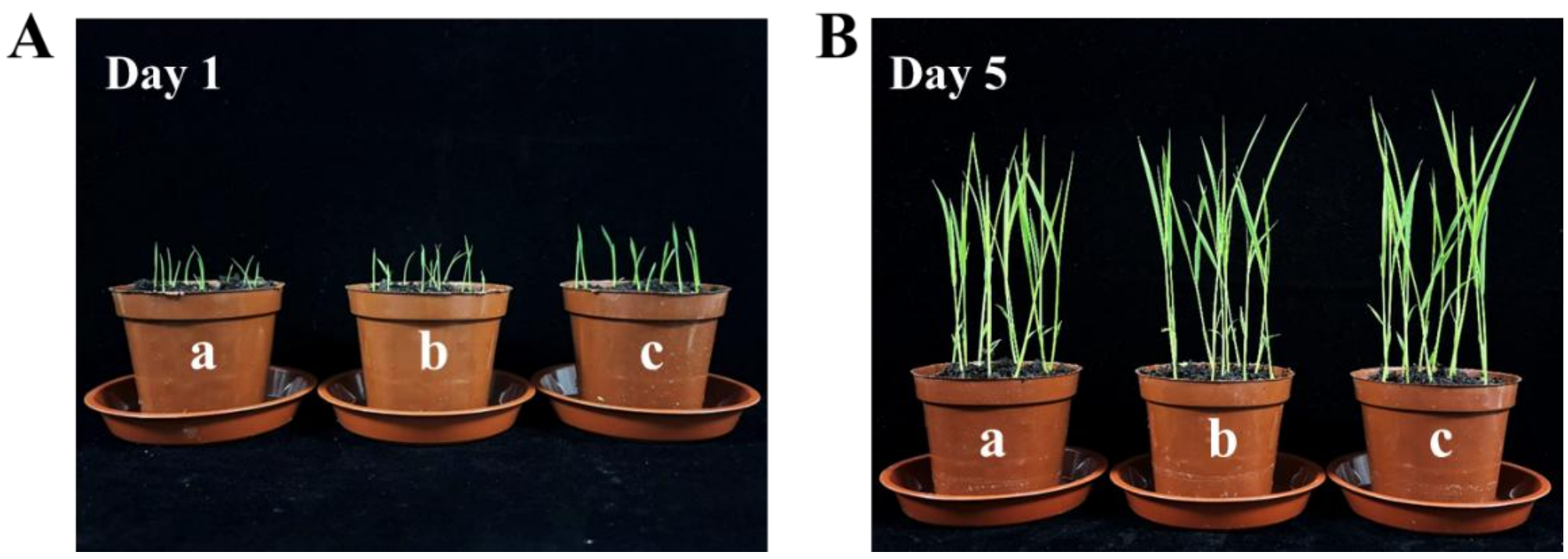

Supplementary Fig. 8 Photographs of rice with different treatment on the 1st and 5th days (a: CK, b: DK6, c: CMCS-DK6). The experiment was repeated three times showing similar results (n=3).

14.Primer information of the RT-qPCR assay

Supplementary Table 3 Primer information of the RT-qPCR assay

Genes	Forward sequence (5′-3′)	Reverse sequence (5′-3′)
*Os*D53	AAGAGTCGCCAAGCAGTTCA	AAGCAACCTTGCAGTTGTGGA
*Os*D14	GGACACGCATCCCTCTACTG	GGCCTGAGAAGAGGAGGGTA
*Os*PIN1B	CCCTCTTGCCTCATCTGTGT	GGTAGAGGCACACGAACTCC
*Os*MYB2	TGGACTCCTAGCTCCCAGAC	TGACGTCGGAGCTTTGCTTT
Ubiquitin	CAAGATGATCTGCCGCAAATGC	TTTAACCAGTCCATGAACCCG
