# Supplementary material for: pH-responsive manganese carboxymethyl chitosan hydrogel releases pesticide to promote rice tillering and suppress root-parasitic weeds
Source: Nat Commun. 2026 Oct 9;17:11467. doi: 10.1038/s41467-026-78320-3 (PMC13739920; doi:10.1038/s41467-026-78320-3)
Supplement: Supplementary file 2 [file 41467_2026_78320_MOESM2_ESM.pdf]

Description of additional Supplementary files

Supplementary Data 1 genes analysis of significant difference between DK6 and DMSO (3h)

Supplementary Data 2 genes analysis of significant difference between DK6 and DMSO (6h)

Supplementary Data 3 genes analysis of significant difference between DK6 and DMSO (12h)

Supplementary Data 4 Go enrichment analysis was performed for the significantly changed genes (6h)

Supplementary Data 5 Kegg enrichment analysis was performed for the significantly changed genes (6h)
