# Supplementary material for: pH-responsive manganese carboxymethyl chitosan hydrogel releases pesticide to promote rice tillering and suppress root-parasitic weeds
Source: Nat Commun. 2026 Oct 9;17:11467. doi: 10.1038/s41467-026-78320-3 (PMC13739920; doi:10.1038/s41467-026-78320-3)
Supplement: Supplementary file 4 [file 41467_2026_78320_MOESM9_ESM.pdf]

nature portfolio

Peer Review File

pH-Responsive Manganese Carboxymethyl Chitosan Hydrogel Releases Pesticide to Promote Rice Tillering and Suppress Root-Parasitic Weeds

Corresponding Author: Professor Weiming Tan

This file contains all reviewer reports in order by version, followed by all author rebuttals in order by version.

Attachments originally included by the reviewers as part of their assessment can be found at the end of this file.

Version 0:

Reviewer comments:

Reviewer #1

(Remarks to the Author)
This study combines strigolactone (SL) signaling pathway inhibitors with pH-responsive manganese-coordinated carboxymethyl chitosan hydrogels to achieve the dual functions of "promoting rice tillering & suppressing root parasitic weeds". The design concept is novel and closely aligned with agricultural production needs, providing a new strategy for sustainable weed control and crop architecture improvement with significant application potential. However, certain aspects of the experimental design still need to be enhanced.

Comments:
1. It is recommended to supplement direct comparative data on release efficiency, duration of efficacy, and cost between this hydrogel and existing pesticide delivery systems (e.g., starch-based and alginate-based hydrogels) in the introduction, so as to further highlight its technical advantages.

2. The study verified the successful loading of DK6 through characterizations such as SEM and XRD, but failed to clarify the correlation between the hydrogel's pore structure (pore size and porosity) and drug release rate. It is recommended to supplement BET specific surface area and pore size distribution data, and conduct drug release experiments on hydrogels with different crosslinking densities (by adjusting Mn^{2+} concentration). This will help establish a quantitative relationship between "microstructure and release kinetics", thereby deepening the scientific validity of the pH-responsive release mechanism.

3. The in vitro release experiments were based on PBS buffer, which differs significantly from the actual soil environment characterized by complex pH, diverse ionic composition, and complex microbial communities. It is recommended to supplement release kinetics and stability data in simulated farmland soils (with different pH values and textures), as well as small-scale field trials (e.g., rice pot experiments combined with Striga infestation simulation). This will verify the effectiveness and duration of the hydrogel in real agricultural scenarios.

4. Transcriptomic analysis revealed that DK6 regulates the strigolactone (SL) signaling pathway and hormone networks, yet the expression changes of key genes have not been validated by independent experiments. It is recommended to verify the expression levels of core genes (e.g., D14, D53, OsMYB2, PIN1) at different treatment time points via quantitative real-time polymerase chain reaction (qPCR).

5. In some experiments, the manuscript mentions "a minimum of three independent replicates", but certain experiments (e.g., tiller number counting, weed germination rate determination) lack the presentation of coefficient of variation or data distribution characteristics. It is recommended to supplement the specific description of statistical grouping methods, and add error bars and sample size labels to key figures (such as release curves and tiller number comparison graphs), so as to improve data readability and rigor.

6. The statistical annotation of some experimental data is ambiguous. For example, the lowercase letters (r,s, m-o, etc.)

corresponding to the IC50 values in Table 1 need to clearly state the grouping basis in the table note (e.g., "Different lowercase letters indicate significant differences between groups at $p<0.05$, based on Duncan's multiple range test"), so as to improve data readability.

7. In the abstract, it is clearly stated that "CMCS-DK6 strongly inhibits the germination of parasitic weeds, especially Striga hermonthica (IC50=1.72 μM)". However, in the Results and Discussion as well as the Methods sections, Striga asiatica is used as the main test object in the weed germination experiments, leading to an inconsistency in the species name. It is recommended to unify the species name of the test object. If two Striga species are involved simultaneously, the corresponding IC50 data and experimental details for S. hermonthica should be supplemented to avoid confusion.

8. In the "Loading Content and Encapsulation Efficiency" section of the Methods, there is a spelling error in "hrdrogel granules" in Equations (1) and (2). It should be corrected to "hydrogel granules" to ensure the rigor of the expression.

9. There is a typesetting issue with Lines 27-338 in the Support Information, please correct it.

Reviewer #2

(Remarks to the Author)
This manuscript presents a thorough and interdisciplinary study combining strigolactone inhibition with polysaccharide-based hydrogel delivery and multiscale mechanistic analyses. While the work is technically strong and has clear potential impact, improvements in statistical rigor, literature contextualization, and data interpretation are necessary to strengthen the conclusions and align the manuscript with the standards of a high-impact journal.
1. Throughout the manuscript, quantitative data are presented without sufficient statistical analysis, which limits the ability to assess the robustness and reproducibility of the reported effects. While several figures report mean values, the manuscript does not consistently specify:
• the number of biological replicates (n),
• whether replicates are biological or technical,
• the statistical tests employed,
• the criteria for statistical significance (e.g., p-values, confidence intervals).

Error bars are shown in some figures but missing in some such as Figure 5, but it is not clearly stated whether these represent standard deviation (SD), standard error of the mean (SEM), or confidence intervals. Claims such as "significantly increased," "strongly inhibited," or "markedly enhanced" are used throughout the text, yet formal statistical evidence supporting these statements is not consistently provided.

2. The manuscript frames the CMCS-Mn2+ hydrogel as an innovative delivery platform, but does not sufficiently situate this approach within the rapidly expanding literature on smart and stimuli-responsive agro-delivery systems. Recent studies have demonstrated environmentally responsive nanomaterials capable of controlled and sustained delivery of plant growth regulators, enhancement of early plant development, modulation of plant architecture under abiotic stress conditions.
Recent studies on smart nanocarriers for agriculture that could be referred to:
• https://pubs.acs.org/doi/10.1021/acsnano.5c14264
• https://doi.org/10.1002/cplu.202400457
• https://pubs.acs.org/doi/10.1021/acsami.4c11981
• https://pubs.acs.org/doi/10.1021/acs.nanolett.2c04506

3. The Mn^{2+}-mediated coordination strategy is effective, but its design rationale would benefit from broader contextualization. A brief acknowledgment of prior metal-coordinated delivery systems used to achieve tunable stability and environmentally responsive release would strengthen the materials design framework.

4. While the pH-dependent swelling and release behavior is clearly demonstrated, its agronomic relevance is not sufficiently discussed. Linking pH responsiveness to realistic soil and rhizosphere conditions would strengthen the translational relevance of the study.
5. The manuscript repeatedly claims sustainability, low environmental impact, and precision delivery but does not include life-cycle assessment (LCA) or green metrics studies.
6. No consideration of long-term environmental fate of Mn^{2+} crosslinked hydrogels in soil.
7. The manuscript presents limited biocompatibility data based on early rice seedling growth, indicating no apparent phytotoxic effects at the tested concentration. However, broader biocompatibility or environmental safety assessments of the designed material are not addressed at the specific tested concentration.
8. Ensure consistent use of terminology (e.g., “smart hydrogel,” “stimuli-responsive hydrogel,” “pH-responsive hydrogel”).
9. Briefly rationalize why the release kinetics follow a first-order model in the context of polymer swelling and diffusion.
10. Acknowledge limitations related to field-scale translation, such as soil heterogeneity and long-term stability.
11. Some figure captions are generally too brief and do not provide sufficient methodological or statistical context for the figures to be interpreted independently. All quantitative data should be presented as mean ± SD or SEM, with the type of error clearly and consistently specified across all figures.
12. Standardize fonts, font sizes, and line thicknesses across all figures. Use a consistent color scheme for recurring conditions (e.g., DK6, CMCS-DK6, controls) throughout the manuscript. Increase the size and clarity of axis labels, legends, and annotations to ensure readability at print scale.

Reviewer #3

(Remarks to the Author)
After careful evaluation of the manuscript titled "pH-Responsive Manganese-Based Carboxymethyl Chitosan Hydrogel: Promoting Rice Tillering and Suppressing Root Parasitic Weeds", we find that the study designs an intelligent carboxymethyl chitosan (CMCS)-based hydrogel delivery system for a novel strigolactone (SL) inhibitor DK6, achieving pH-responsive release and demonstrating excellent performance in promoting rice tillering and suppressing root parasitic weeds. The research spans chemical synthesis, materials science, plant physiology, and computational biology, exhibiting strong innovation and potential application value. However, several aspects could be improved. The following comments are intended to enhance the rigor, completeness, and readability of the article.

1. In the weed germination inhibition assay, a control group with blank hydrogel (without DK6) was not included. This omission makes it impossible to rule out potential effects of the CMCS-Mn carrier itself on germination. It is recommended to supplement the experimental results with a blank hydrogel group.
2. The manuscript only evaluates the safety of the hydrogel on rice seedlings and does not address its impact on non-target organisms such as zebrafish. It is recommended to supplement with a simple experiment assessing the effects of the blank hydrogel at different concentrations on zebrafish to preliminarily evaluate its environmental safety.
3. The authors selected CMCS as the carrier based on its biocompatibility and modifiability but did not explain why manganese ions were preferentially chosen as the crosslinking agent. A brief justification should be added.
4. Given that manganese (Mn) is an essential micronutrient, the release of Mn^{2+} from the hydrogel may confer an additional growth-promoting effect on plants. It is recommended to briefly address this point in the Discussion section.
5. In Figure 1A, "X=C or N" could be revised to "X=C or N, Y= N" or simply removed. Furthermore, in the METHODS section, Equation 8 is followed directly by Equation 10. Please check whether Equation 9 is missing or if the numbering is incorrect.
6. The heading "Design, Synthesis, and Identification of Small Molecule DK6" in the RESULTS AND DISCUSSION section is incorrectly formatted. Additionally, would it be more appropriate to present the bioactivity results in the order of compounds DK1 to DK32?
7. In the METHODS section, "Synthesis of Target Compounds DK1-DK33." is mentioned, but compound DK33 is not referred to anywhere else in the manuscript. Please check and revise accordingly. Also, in the "Seed Germination Assay" subsection, the citation for reference 15 is incorrectly formatted and should be corrected.
8. Abbreviations should be defined upon first use (e.g., FT-IR). Some sections use abbreviations directly without definition. Additionally, there are minor grammatical errors and unclear expressions, such as "tetrahydropsyrole ring," which should be standardized to "tetrahydropyrrole ring."
9. The sterilization protocol for parasitic weed seeds involves treatment with 1% NaClO for 2 minutes, followed by 75% ethanol for 2 minutes. Compared to some established methods, this treatment duration is relatively short. Please provide justification or cite relevant literature to support the effectiveness of this protocol.
10. Please specify the instrument or tool used to control the droplet size of the emulsion during hydrogel preparation to support the reproducibility of the experiment.
11. Figure 6: The germination inhibition assay for parasitic weeds should specify whether a standalone GR24 treatment group was included, and at what concentrations it was applied.
12. The explanation of the pH-responsive mechanism of the hydrogel is clear but could be enhanced by adding a schematic diagram in the Supporting Information illustrating the competition between COOH ionization and Mn^{2+} coordination.
13. What was the rationale for selecting 6 hours as the key time point for GO/KEGG analysis?
14. Please meticulously check the formatting of all references throughout the manuscript to ensure full compliance with the journal's requirements.

Reviewer #4

(Remarks to the Author)
Dear Authors,
It was a pleasure reading your manuscript; I hope my comments are useful and help improve your manuscript. Please let me know if anything is not clear.

The sentence "Indole derivatives exhibit potential for plant growth regulation due to their ability to mimic peptide conformations and reversibly bind target enzymes" is a very close paraphrase of reference 46, which is not a primary source. I would suggest providing more context using primary articles to better understand why indole derivatives have good potential. Reversibly binding target enzymes is a property many non-indole derivatives also have.

The initial experiment evaluating the tillering-promoting activity of synthesized compounds in the presence of GR24 appears to be lacking a GR24-only control. Also, the figure legend states compounds were used at a concentration of 10 µM, while the text says 80 µM. Which one is it? What was the concentration of GR24 used for co-treatments? What is CK- should it be spelled out on the legend? It seems to me these are two separate experiments, one is germination inhibition while the other is tillering evaluation. It is unclear if this is true and should be better specified. How were IC50s calculated using a single dose rather than an inhibitor titration? The methods section talks about this, but the figures do not reflect a titration. Furthermore, Figure S3A shows 3 different concentrations were used in the tillering assay (1, 5, and 15 µM); it would not be possible/reliable to obtain the IC50s reported in the text using only these concentrations.

Were other varieties of Striga (i.e., hermonthica) tested in the germination suppression assay? What about a non-Striga control expressing the targeted receptor/its absence to make sure the reason for germination was its engagement?

There is a typo in the subtitle "Release Hehavior of CMCS-DK6 Hydrogel Delivery System"

The encapsulation capacity seems rather low; this is fine, as it is an exploratory article, but it would be valuable if potential approaches to improve this were included in the discussion.

The explanation of the hydrogel being more efficacious at inhibiting parasitic germination than DK6 alone "hydrogel matrix likely enhances DK6 bioactivity, potentially due to its excellent biocompatibility and sustained-release properties, which stabilize DK6 interaction with weed seeds and prolong its duration of action" seems too vague to me. Often, free drugs can be more active, due to exposure, pharmacokinetics, etc., so I am wary of this explanation. The figure legend lacks enough explanation to understand properly how this experiment (Fig. 6) was conducted. Unclear if proper controls (hydrogel only, GR24, what is the baseline of germination in the absence of compound (100%?)) were included. Also, it is unclear what the 0 µM treatment in Figure 6A means, but visually, it looks like more seedlings are germinating in the CMCS-DK6 treatment than on the DK6-only treatment. Does this not contradict the proposal that CMCS enhances activity?

"hydrogel matrix likely enhances DK6 bioactivity, potentially due to its excellent biocompatibility and sustained-release properties, which stabilize DK6 interaction with weed seeds and prolong its duration of action" - the data shown on graph 6E does not agree with this statement. It is also unclear what the small a, b, and c on top of each bar represent.

"Based on the established mechanism that SL inhibits hypocotyl elongation in a MAX2-dependent manner, we hypothesized that DK6 might function as an SL signaling inhibitor" this section could benefit from a reference.

For the hypocotyl elongation assays: Graph 7C shows GR24-treated seedlings had a hypocotyl of ~0.7 cm, while DK6-treated seedlings at 5 µM (7A) had a length of 0.8 cm. When the 2 are combined in Graph 7B, the length is 0.6 cm. However the claim in the text is "In the presence of GR24 (3 µM), both DK6 and KK094 effectively reversed the GR24-induced suppression of hypocotyl elongation". This is not supported by the data shown. The a/b labels on the graphs are again quite confusing.

"In summary, DK6 likely acts by specifically inhibiting SL signal transduction, thereby relieving its suppressive effect on hypocotyl elongation, in a MAX2-dependent manner" I would not conclude this from the data presented. Additionally, no direct test against a SL receptor was performed, so the conclusion that DK6 acts by specifically inhibiting SL signal transduction seems far-fetched.

The statement "the dual anchoring provided by a hydrogen bond with SER-97 (2.7 Å) and hydrophobic interactions with VAL-144 and PHE-195 is insufficient to match the stability of DK6" is unclear. KK094 is a validated ligand, saying that the interactions it forms with certain residues are no match for those interactions predicted for DK6 is a bit confusing.

The figures for DK6 docking are too small or low quality for me to provide an informed opionion on them (characters are distorted). PDB code 7SNU is talked about in the data availability section, but no reference to that paper was made.

Some residues in the text have an unclear numbering format, i.e., PHE-A28 or VAL-A219. What does the character preceding the number mean?

I am unconvinced a binding difference of 0.2 kcal/mol is truly reflective of a "significantly stronger binder with superior binding affinity". The score difference is higher for OsD14, however the parasitic receptor ShHTL7 shows pretty much identical binding energies, so it is unclear why DK6 performs better than KK094. Is it possible it is not actually binding to ShHTL7 but rather to a different receptor?

Graphs 8B and 8C show that the Rg and SASA parameters for all 4 systems evaluated are pretty much identical. However, the RMSD fluctuation shown on 8A does appear to be higher for ShHTL7-KK094 than all the other systems. Are these 3 graphs from the same simulations? Also, there is no mention in the text or methods of what residue entities were analyzed to quantify RMSD, and the visualized trajectories are not included, so it is impossible to assess what 8A is actually plotting. Furthermore, the text claims DK6 forms more H-bonds with the 2 proteins, but the graphs in 8E do not show that, except for the L7-KK094 system. If the claim is that DK6 presents less fluctuation within the pocket, why would there be more interactions at different points in the simulation, instead of more stability (like that one shown in the ShHTL7-DK6 system)? I would encourage the authors to discuss their findings in the context of previous literature using MD simulations for Striga inhibitors and chemical discovery.

Most of the section titled "Analysis of SL Pathway and Genes Related to Rice Tillering" is hypothetical and relies on the assumption that DK6 directly interacts with a strigolactone receptor in a parasitic plant. I would suggest removing phrases such as "providing molecular-level confirmation of the pharmacological activity of DK6 as an inhibitor of the SL signaling pathway" unless direct evidence is presented. Framing it as a discussion and further directions would be adequate.

The claim "Furthermore, this study reveals a novel mechanism by which DK6 regulates tillering through interference with a multi-hormone network" is not substantiated by the data. Elucidating this proposed novel mechanism would necessitate doing controlled experiments to test this proposal.

"Thus, this work not only confirms the pharmacological function of DK6 as an SL signaling inhibitor at the molecular level, but also elucidates its complex role in modulating rice tillering via a multi-hormone network, providing a new theoretical

foundation for improving plant architecture in crops." No direct evidence of engagement with an SL receptor is presented, so this claim is not subtantiated. Also, there is no reference to previous studies proposing molecular targeting of Striga receptors to design potent and specific antagonists, however they say that this study provides a new theoretical foundation for a related purpose; this claim seems to be too ambitious.

"Mechanistic studies revealed that DK6 stably binds to the OsD14 and ShHTL7 receptors with higher binding affinity than KK094" It should be made clear that, aside from docking, no experiments show direct engagement with OsD14 and ShHTL7 in this study.

It would be great, as a proof of concept, to show how well the proposed hydrogel works on actual soil conditions that resemble the fields. Or at least comment on this expectation in the discussion.

Version 1:

Reviewer comments:

Reviewer #2

(Remarks to the Author)
The manuscript is recommended for publication as all our concerns were addressed

Reviewer #3

(Remarks to the Author)
The authors have resolved the raised issues, and the current version of the manuscript can be accepted.

Reviewer #4

(Remarks to the Author)
Thank you for providing your responses to my comments. Most of them have been addressed, but a few considerable outstanding points remain.
1. In their rebuttal, the authors explain that compound DK6 was tested at 1, 5, 15 μM to be able to obtain the 2.15 μM IC50; however, there is no mention of these concentrations in the methods or main text. On page 2, the target compounds were said to have been tested at 5-80 μM. This would not allow the obtention of such a low IC50.
2. The statement "Molecular docking is a key technique for elucidating ligand–receptor interactions and quantifying binding affinity." Is not correct; yes, docking is used to predict ligand binding to a protein pocket; using it to quantify binding affinity is not something serious biochemical/enzymatic studies perform.
3. The authors claim binding/structural studies of AtD14 binding to DK6 are reflective of the mechanism SL receptors OsD14 and ShHTL7 would follow. Nevertheless, Fig. 7E shows no docking on AtD14, and instead shows OsD14 and ShHTL7. It is claimed that DK6 has a "binding advantage" in ShHTL7 of 0.2 kcal/mol; this is within the range of error, and I do not agree with that claim. It is also unclear how those values were calculated as none of the figures contain them. If the point is to show the binding mode between 2 highly similar receptors (OsD14 and ShHTL7) is similar, the docking figures presented do not represent that. The compound adopts completely different orientations in the 2 pockets, and the catalytic residues interact with different/opposite atoms of the small molecule. Overall, my conclusion is that the MD/docking section does not support the claims that DK6 acts primarily through ShHTL7 and is a better inhibitor of parasitic SL receptors than KK094.
4. While I partially understand the authors did not have OsD14/ShHTL7 protein and they were not able to generate knockdown lines of these receptors (even though other groups have generated both recombinant protein and plant lines), the lack of correlation with docking makes the whole argument weaker. The only claim fully supported in this part of the study, using MST, is that DK6 appears to have a lower Kd than KK094 in an Arabidopsis SL receptor.
5. The reference cited for PDB entry code 7SNU is incorrect, please add the correct primary reference.
6. The authors claim that new qPCR experiments are helpful to reach the conclusion "this study reveals a novel mechanism by which DK6 regulates tillering through interference with a multi-hormone network." This is not my field of expertise, and I am not certain these experiments fully support the conclusion. Again, the lack of data that shows direct engagement with the proposed parasitic receptors is the main problem here. However, the statements included in the previous version were toned down and might be appropriate considering the experimental evidence provided.
7. A lot of the original questions were answered through MST; however, there is little to no information to show protein purification (aside from the methods section, which include a few phrases clearly copied from a generic recombinant protein purification protocol). There is also no explanation as to how the protein was fluorescently labeled or the settings used for thermal fluorescence. No explanation related to the number of replicates (if any) for the MST experiment or any statistics related to it.

Open Access This Peer Review File is licensed under a Creative Commons Attribution 4.0 International License, which permits use, sharing, adaptation, distribution and reproduction in any medium or format, as long as you give appropriate credit to the original author(s) and the source, provide a link to the Creative Commons license, and indicate if changes were made.
In cases where reviewers are anonymous, credit should be given to 'Anonymous Referee' and the source.
The images or other third party material in this Peer Review File are included in the article's Creative Commons license, unless indicated otherwise in a credit line to the material. If material is not included in the article's Creative Commons license and your intended use is not permitted by statutory regulation or exceeds the permitted use, you will need to obtain permission directly from the copyright holder.
To view a copy of this license, visit https://creativecommons.org/licenses/by/4.0/

REVIEWER COMMENTS

Reviewer #1 (Remarks to the Author):

This study combines strigolactone (SL) signaling pathway inhibitors with pH-responsive manganese-coordinated carboxymethyl chitosan hydrogels to achieve the dual functions of "promoting rice tillering & suppressing root parasitic weeds". The design concept is novel and closely aligned with agricultural production needs, providing a new strategy for sustainable weed control and crop architecture improvement with significant application potential. However, certain aspects of the experimental design still need to be enhanced.

Comments:

1. It is recommended to supplement direct comparative data on release efficiency, duration of efficacy, and cost between this hydrogel and existing pesticide delivery systems (e.g., starch-based and alginate-based hydrogels) in the introduction, so as to further highlight its technical advantages.

Our response: Thank you very much for your valuable suggestion. We fully agree that explicitly highlighting these points will more clearly and convincingly articulate the potential applicative value of this study.

Compared with starch-based and alginate-based hydrogels, our Mn^{2+}-coordinated carboxymethyl chitosan (CMCS) system offers several advantages for pesticide delivery. First, the Mn^{2+} coordination bonds enhance the gel network strength, overcoming the burst release issue of starch-based hydrogels and the poor encapsulation efficiency of hydrophobic drugs often seen with alginate-based systems. In this study, the CMCS-DK6 hydrogel achieved a high drug loading of 26.9% and an encapsulation efficiency of 79.6% for the SL inhibitor DK6, markedly outperforming most conventional systems. Second, the Mn^{2+} coordination bonds impart a sharp pH-responsive behavior, with a cumulative release of 95.7% within 24 h under alkaline conditions (pH 8.5–9.5), enabling on-demand release; traditional hydrogels typically have a narrow responsive range or require additional modifications. Moreover, Mn^{2+} serves both as a crosslinking agent and a micronutrient, providing manganese supplementation while avoiding toxic crosslinkers such as glutaraldehyde, thereby improving environmental safety. Collectively, the CMCS-Mn^{2+} system outperforms conventional polysaccharide-based hydrogels in terms of drug loading efficiency, responsive release, and multifunctional integration, offering a superior solution for the delivery of hydrophobic pesticide molecules.

Following your suggestion, we will add the following text at the end of the Introduction (before the last paragraph) in the revised manuscript: "Although natural polysaccharide-based hydrogels are promising for pesticide delivery, their performance varies. Mn^{2+} coordination bonds reinforce polysaccharide hydrogels, overcoming burst release, low efficiency, rapid degradation, and poor mechanical strength of starch-based and alginate/cassava systems[35,36,50]. Under alkaline conditions, OH^- competitively disrupts coordination bonds, thereby enabling on-demand release—unlike conventional polysaccharide hydrogels, which have a narrow pH response and require chemical modification[45,51,52]. Mn^{2+} serves both as a crosslinker and a micronutrient, avoiding toxic crosslinkers and achieving delivery-nutrient synergy. The Mn^{2+}-CMCS hydrogel seems to be low in cost, easy to prepare, fairly quick to respond, capable of sustained release, and reasonably multifunctional."

We believe that adding this paragraph will help readers better understand the rationale for choosing the Mn^{2+}-CMCS hydrogel. We will carefully implement these revisions in the revised manuscript. Once again, we sincerely thank you for your insightful comments, which are crucial for improving the quality of our paper.

35. Bai, C. et al. Starch-based hydrogel loading with carbendazim for controlled-release and water absorption. *Carbohydr. Polym*. **125**, 376-383 (2015).

36. Yan, D. D., Pan, Y. F., Cai, P. X. & Xiao, H. N. Redox/pH dual-responsive sodium alginate/cassava starch composite hydrogel beads for slow release of insecticides. *React. Funct. Polym*. **215**, 106389 (2025).

45. Xu, C. L. et al. Multifunctional manganese-based carboxymethyl chitosan hydrogels for pH-triggered pesticide release and enhanced fungicidal activity. *Carbohydr. Polym*. **262**, 117933 (2021).

50. Venkatachalam, S. K., Senthilkumar, D., Duraisamy, N., Sheela, T. & Moovendhan, M. Advancements in polysaccharide-based carriers for eco-friendly fungicide delivery. *Carbohydr. Polym. Technol. Appl*. **12**, 101026 (2025).

51. Zhang, Z. et al. Research progress of a pesticide polymer-controlled release system based on polysaccharides. *Polymers* **15**, 2810 (2023).

52. Huang, L. J. et al. Preparation and properties of modified cassava starch slow-release hydrogel with acid and alkali resistance. *Ind. Crops Prod*. **206**, 117699 (2023).

2. The study verified the successful loading of DK6 through characterizations such as SEM and XRD, but failed to clarify the correlation between the hydrogel's pore structure (pore size and porosity) and drug release rate. It is recommended to supplement BET specific surface area and pore size distribution data, and conduct drug release experiments on hydrogels with different crosslinking densities (by adjusting Mn2+ concentration). This will help establish a quantitative relationship between "microstructure and release kinetics", thereby deepening the scientific validity of the pH-responsive release mechanism.

Our response: Thank you very much for your valuable suggestion. We agree on the importance of establishing a quantitative relationship between microstructure and release kinetics, and we respond as follows.

Regarding BET surface area and pore size distribution measurements. Conventional nitrogen-adsorption BET is mainly applicable to rigid, dry materials. Hydrogels, however, are soft and hydrophilic in their swollen state, and the drying process inevitably leads to irreversible network collapse; thus, the pore parameters measured in the “dry” state cannot accurately reflect the true swollen structure. Nevertheless, we performed the test, but the results were unsatisfactory (BET report is shown below), likely due to drying-induced collapse or the inherently non-rigid, amorphous nature of the material.

CMCS-Mn: Summary Report

Summary Report	
Surface Area	
BET Surface Area:	-10.6836 m²/g
Horvath-Kawazoe	
Maximum pore volume at p/p° = 0.000000000:	0.000000 cm³/g
Median pore width:	0.0000 nm
Pass/Fail	
S A:Single-point BET:	Failed: Value < lowest allowed 204.0000 by 204.0000
S A:Single-point BET:	
S A:Multi-point BET:	Failed: Value < lowest allowed 210.0000 by 220.6836
S A:Multi-point BET:	
P S:Average pore diameter:	Failed: Value < lowest allowed 10.0000 by 10.0000
P S:Average pore diameter:	
P V:Adsorption Total: - 0.99800000 Relative Pressure	Failed: Value < lowest allowed 0.540000 by 0.540000
P V:Adsorption Total: - 0.99800000 Relative Pressure	

CMCS-Mn CMCS-DK6 +

CMCS-Mn: BET Report and BET Surface Area Plot

BET Report		
BET surface area:	-10.6836 ± 0.0654 m²/g	
Slope:	-0.408249 ± 0.002486 g/cm³ STP	
Y-intercept:	0.000840 ± 0.000207 g/cm³ STP	
C:	-484.721739	
Qm:	-2.4545 cm³/g STP	
Correlation coefficient:	-0.9999629	
Molecular cross-sectional area:	0.1620 nm²	
Relative Pressure (p/p°)	Quantity Adsorbed (cm³/g STP)	1/[Q(p°/p - 1)]
0.034502084	-2.678728289	-0.013340292
0.068982373	-2.724760226	-0.027192675
0.091922846	-2.765003429	-0.036610447
0.11486911	-2.811171108	-0.046164536

BET Surface Area Plot			
mn : Micro+Meso		Not Fitted	
Relative Pressure (p/p°)	1/[Q(p°/p - 1)]	Relative Pressure (p/p°)	1/[Q(p°/p - 1)]
0.034502084	-0.013340292	0.137847661	-0.055974695
0.068982373	-0.027192675	0.159710842	-0.065781604
0.091922846	-0.036610447	0.179190543	-0.074279619
0.11486911	-0.046164536	0.199184759	-0.083319697
		0.219451689	-0.093339426
		0.269176405	-0.119044673
		0.319177782	-0.147899877

CMCS-Mn CMCS-DK6 +

CMCS-DK6: Summary Report

Summary Report	
Surface Area	
BET Surface Area:	-50.5637 m²/g
BJH Adsorption cumulative surface area of pores between 1.7000 nm and 300.0000 nm diameter:	5.0091 m²/g
BJH Desorption cumulative surface area of pores between 1.7000 nm and 300.0000 nm diameter:	3.3809 m²/g
Pore Volume	
Single point adsorption total pore volume of pores less than 192.7178 nm diameter at p/p° = 0.989960708:	0.130596 cm³/g
BJH Adsorption cumulative volume of pores between 1.7000 nm and 300.0000 nm diameter:	0.130597 cm³/g
BJH Desorption cumulative volume of pores between 1.7000 nm and 300.0000 nm diameter:	0.109903 cm³/g
Pore Size	
Adsorption average pore diameter (4V/A by BET):	-10.3312 nm
BJH Adsorption average pore diameter (4V/A):	104.2877 nm
BJH Desorption average pore diameter (4V/A):	130.0283 nm
Horvath-Kawazoe	
Maximum pore volume at p/p° = 0.000000000:	0.000000 cm³/g
Median pore width:	-0.0000 nm
Pass/Fail	
S A:Single-point BET:	Failed: Value < lowest allowed 204.0000 by 204.0000
S A:Single-point BET:	
S A:Multi-point BET:	Failed: Value < lowest allowed 210.0000 by 260.5637
S A:Multi-point BET:	
P S:Average pore diameter:	Failed: Value < lowest allowed 10.0000 by 20.3312
P S:Average pore diameter:	
P V:Adsorption Total: - 0.99800000 Relative Pressure	Failed: Value < lowest allowed 0.540000 by 0.409404
P V:Adsorption Total: - 0.99800000 Relative Pressure	

CMCS-Mn CMCS-DK6 +

CMCS-DK6: BET Report and BET Surface Area Plot

BET Report		
BET surface area:	-50.5637 ± 0.4848 m²/g	
Slope:	-0.086479 ± 0.000822 g/cm³ STP	
Y-intercept:	0.000398 ± 0.000070 g/cm³ STP	
C:	-216.386701	
Qm:	-11.6169 cm³/g STP	
Correlation coefficient:	-0.9999096	
Molecular cross-sectional area:	0.1620 nm²	
Relative Pressure (p/p°)	Quantity Adsorbed (cm³/g STP)	1/[Q(p°/p - 1)]
0.052971621	-13.28109154	-0.00421159
0.069122828	-13.36213293	-0.00555717
0.092058472	-13.46192311	-0.0075318
0.115085483	-13.57487814	-0.00958039

BET Surface Area Plot			
dk6 : Micro+Meso		Not Fitted	
Relative Pressure (p/p°)	1/[Q(p°/p - 1)]	Relative Pressure (p/p°)	1/[Q(p°/p - 1)]
0.052971621	-0.004211594	0.137971802	-0.011693441
0.069122828	-0.005557165	0.159328357	-0.013742683
0.092058472	-0.0075318	0.179217893	-0.015716399
0.115085483	-0.009580392	0.199170063	-0.017766126
		0.219095913	-0.019886178
		0.268955487	-0.025731187
		0.3189217	-0.032339074

CMCS-Mn CMCS-DK6 +

Regarding the choice of Mn^{2+} concentration. The use of 0.2 M Mn^{2+} was determined through comprehensive optimization of sphericity, drug loading, and encapsulation efficiency, consistent with literature reports. Systematically varying the Mn^{2+} concentration would simultaneously affect crosslinking density, swelling ratio, and drug loading, making it difficult to attribute differences in release rates solely to pore structure. Therefore, this study focused on the optimized system with a single crosslinking density (0.2 M Mn^{2+}), highlighting the driving mechanisms of DK6 release under different pH conditions through increased swelling and dynamic changes in coordination bonds. We thank you again for your thoughtful review.

3. The in vitro release experiments were based on PBS buffer, which differs significantly from the actual soil environment characterized by complex pH, diverse ionic composition, and complex microbial communities. It is

recommended to supplement release kinetics and stability data in simulated farmland soils (with different pH values and textures), as well as small-scale field trials (e.g., rice pot experiments combined with Striga infestation simulation). This will verify the effectiveness and duration of the hydrogel in real agricultural scenarios.

Our response: Thank you for your valuable comments. Your point regarding the discrepancy between in vitro release experiments and actual soil conditions is highly insightful and has provided important guidance for improving the completeness and practical relevance of our study. In response, we have conducted additional experiments and revised the manuscript accordingly. Our detailed responses are as follows.

First, we performed release kinetics and stability experiments in paddy soils. Soils from six typical rice-growing regions (Hunan, Jiangsu, Sichuan, Henan, Heilongjiang, and Liaoning) were collected, and the cumulative release rate of CMCS-DK6 hydrogel over 48 hours was measured and fitted to kinetic models. The cumulative release rates ranged from 63.5% to 86.1% across different soils, all reaching effective levels, and the release behavior followed a first-order kinetic model (R^2 =0.94-0.99). Although soil buffering and adsorption partially attenuated the pH-responsive regulation, the hydrogel still achieved effective and controllable release. These results have been added to the “Release Behavior of CMCS-DK6 Hydrogel Delivery System” section of the revised manuscript (see Supplementary Fig. 6 and Table 1), with a brief discussion in the main text. In addition, we conducted a 45-day rice cultivation experiment in the same six soils with varying concentrations of CMCS-DK6. The results showed that CMCS-DK6 promoted rice tillering to varying degrees in all soils (see Supplementary Fig. 7), further validating its bioactivity and persistence in real soil environments. We have also noted in the manuscript that future studies should quantify the influence of soil physicochemical properties on release kinetics to optimize application strategies.

Regarding your suggestion to perform rice pot experiments simulating *Striga asiatica* infestation, we fully agree on its importance for validating field efficacy. However, due to seasonal availability of Striga asiatica seeds and the long time required to establish a stable and reproducible infestation system (including seed preconditioning and optimization of host-parasite co-culture), we were unable to complete this experiment within the revision period. We have included this experiment in our future work plan and will prioritize it once conditions permit, in order to comprehensively evaluate the protective effect and duration of efficacy of the CMCS-DK6 hydrogel under parasitic weed infestation. We kindly ask for your understanding. Thank you again for your constructive comments.

4. Transcriptomic analysis revealed that DK6 regulates the strigolactone (SL) signaling pathway and hormone networks, yet the expression changes of key genes have not been validated by independent experiments. It is recommended to verify the expression levels of core genes (e.g., D14, D53, OsMYB2, PIN1) at different treatment time points via quantitative real-time polymerase chain reaction (qPCR).

Our response: Thank you very much for your valuable suggestions, which are of great significance for further improving our study.

In our transcriptomic analysis, we included three time points (3, 6, and 12 h) after DK6 treatment. Among these, the 6 h time point yielded the highest number of differentially expressed genes (3,718), including 1,691 unique genes—far more than at the other time points. This suggests that 6 h represents a critical window for DK6-induced transcriptional regulation, with the strongest temporal association with subsequent phenotypic formation. Therefore, we selected the 6 h samples for GO and KEGG enrichment analyses to focus on early changes in key metabolic and signaling pathways. Based on your suggestion, we have additionally performed RT-qPCR experiments to validate the expression changes of *Os*D53, *Os*D14, *Os*PIN1B, and *Os*MYB2 in rice treated with DK6 for 6 h, further supporting the observations from the transcriptome analysis. We will include the relevant experimental data in the revised manuscript, add a new “RT-qPCR” subsection in the Methods section to describe the experiments, and systematically discuss the validation results in the Results and Discussion section (see manuscript-track change). Once again, thank you for your valuable comments. We will carefully revise and improve the manuscript accordingly.

5. In some experiments, the manuscript mentions "a minimum of three independent replicates", but certain experiments (e.g., tiller number counting, weed germination rate determination) lack the presentation of coefficient of variation or data distribution characteristics. It is recommended to supplement the specific description of statistical grouping methods, and add error bars and sample size labels to key figures (such as release curves and tiller number comparison graphs), so as to improve data readability and rigor.

Our response: Thank you very much for your thorough review and valuable suggestions. We fully agree on the

importance of clearly presenting data variability and statistical information. Accordingly, we will add the following in the revised manuscript: In the “Statistical Analysis” subsection of the Methods, we will clarify that all experiments were performed with at least three biological replicates, data are presented as mean ± standard deviation (SD), and one-way ANOVA with Tukey's multiple comparison test was used, with statistically significant differences ($p < 0.05$) indicated by different letters. In addition, we will add error bars and indicate sample sizes (n) in all key figures, and explain the meaning of the letters in the figure legends. We thank you again for your input, which will significantly improve the rigor and clarity of our manuscript.

6. The statistical annotation of some experimental data is ambiguous. For example, the lowercase letters (r,s, m-o, etc.) corresponding to the IC50 values in Table 1 need to clearly state the grouping basis in the table note (e.g., "Different lowercase letters indicate significant differences between groups at p<0.05, based on Duncan's multiple range test"), so as to improve data readability.

Our response: Thank you very much for your rigorous correction. We fully agree that the meaning of the lowercase letters should be clearly indicated in the table footnote. Table 1 and the associated data analyses were performed using Tukey's multiple comparison test. We will add the following annotation: “Statistical significance among means was determined using one-way ANOVA followed by Tukey's multiple comparison test. Different letters indicate statistically significant differences ($p < 0.05$)” We have also provided a unified explanation for the statistical annotations in all figures and tables throughout the manuscript. Thank you again for your assistance.

7. In the abstract, it is clearly stated that "CMCS-DK6 strongly inhibits the germination of parasitic weeds, especially Striga hermonthica (IC50=1.72 μM)". However, in the Results and Discussion as well as the Methods sections, Striga asiatica is used as the main test object in the weed germination experiments, leading to an inconsistency in the species name. It is recommended to unify the species name of the test object. If two Striga species are involved simultaneously, the corresponding IC50 data and experimental details for S. hermonthica should be supplemented to avoid confusion.

Our response: Thank you very much for your thorough review and correction. We confirm that the species used in this study is *Striga asiatica*, and the appearance of *Striga hermonthica* in the abstract was a clerical error, for which we sincerely apologize. We will immediately correct it to *Striga asiatica*. We thank you again for your rigorous guidance, which has helped us avoid this important mistake.

8. In the "Loading Content and Encapsulation Efficiency" section of the Methods, there is a spelling error in "hrdrogel granules" in Equations (1) and (2). It should be corrected to "hydrogel granules" to ensure the rigor of the expression.

Our response: Thank you for your careful correction. The term “hrdrogel granules” was indeed a typo, and the correct spelling should be “hydrogel granules”. We have corrected it in equations (1) and (2) in the “Loading content and encapsulation efficiency” section and have reviewed the entire manuscript to ensure consistency. Thank you again for your assistance.

9. There is a typesetting issue with Lines 27-338 in the Support Information, please correct it.

Our response: Thank you very much for carefully reviewing the Supplementary of our manuscript and for pointing out the formatting issues.

We have now carefully checked the content of the Supplementary and thoroughly reviewed and reformatted this section to ensure it is compliant and clearly readable. We apologize again for any inconvenience caused by our oversight, and sincerely thank you for your rigorous and professional work, which has greatly helped us improve the quality of our manuscript.

Reviewer #2 (Remarks to the Author):

This manuscript presents a thorough and interdisciplinary study combining strigolactone inhibition with polysaccharide-based hydrogel delivery and multiscale mechanistic analyses. While the work is technically strong and has clear potential impact, improvements in statistical rigor, literature contextualization, and data interpretation are necessary to strengthen the conclusions and align the manuscript with the standards of a high-impact journal.

1. Throughout the manuscript, quantitative data are presented without sufficient statistical analysis, which limits the ability to assess the robustness and reproducibility of the reported effects. While several figures report mean values, the manuscript does not consistently specify:

• the number of biological replicates (n),
• whether replicates are biological or technical,
• the statistical tests employed,
• the criteria for statistical significance (e.g., p-values, confidence intervals).

Error bars are shown in some figures but missing in some such as Figure 5, but it is not clearly stated whether these represent standard deviation (SD), standard error of the mean (SEM), or confidence intervals. Claims such as "significantly increased," "strongly inhibited," or "markedly enhanced" are used throughout the text, yet formal statistical evidence supporting these statements is not consistently provided.

Our response: Thank you for your thorough review and valuable comments. We agree that the quantitative statistical analysis needs to be more comprehensive, which is crucial for the scientific rigor, robustness, and reproducibility of the findings. In response, we have revised the manuscript as follows:

For all quantitative data, we have specified in the figure legends, table footnotes, or main text that the data are derived from three independent biological replicates. Error bars have been added, with the description: "Data are presented as mean ± SD, n = 3." The statistical method used is now clearly indicated: "Statistical significance among means was determined using one-way ANOVA followed by Tukey's multiple comparison test. Different letters indicate statistically significant differences (n = 3, $p < 0.05$)." These details have been explicitly stated in the "Statistical analysis" subsection of the Methods and in the relevant figures/tables.

We believe these revisions have substantially improved the scientific clarity and transparency of the data, allowing readers and future researchers to better assess the reliability and reproducibility of our findings. We greatly appreciate your insightful input.

2. The manuscript frames the CMCS-Mn2+ hydrogel as an innovative delivery platform, but does not sufficiently situate this approach within the rapidly expanding literature on smart and stimuli-responsive agro-delivery systems. Recent studies have demonstrated environmentally responsive nanomaterials capable of controlled and sustained delivery of plant growth regulators, enhancement of early plant development, modulation of plant architecture under abiotic stress conditions.

Recent studies on smart nanocarriers for agriculture that could be referred to:

• https://pubs.acs.org/doi/10.1021/acsnano.5c14264
• https://doi.org/10.1002/cplu.202400457
• https://pubs.acs.org/doi/10.1021/acsami.4c11981
• https://pubs.acs.org/doi/10.1021/acs.nanolett.2c04506

Our response: Thank you for your insightful comments and for sharing the highly relevant literature. Your suggestions are crucial for accurately positioning the novelty of our study. We fully agree that our work should be framed within the broader context of smart agricultural delivery systems, and the provided references have been invaluable in highlighting the rapid progress and diverse strategies in this field.

After careful reading, we recognize that our study aligns well with this cutting-edge area. Accordingly, we have revised the penultimate paragraph of the Introduction as follows (inserted after the sentence "…CMCS hydrogels represent a highly promising functional carrier for controlled pesticide release."): "Metal coordination-based stimuli-responsive delivery systems hold promise for smart agriculture. Recent studies report zinc-phenolic networks (e.g., CAFZn) for controlled release of plant growth regulators[46], iron-tannic acid core-shell structures (Phelm) for smart seed coating[47], and zeolitic imidazolate frameworks (ZIF-8) as nanocarriers for biostimulants and micronutrients while inhibiting Striga germination[48,49]. These systems enable abiotic stress response and seed nano-priming, promoting early development and stress tolerance. In this context, we constructed a Mn^{2+}-crosslinked CMCS hydrogel using the essential micronutrient Mn^{2+} as a crosslinker."

We greatly appreciate your profound insights. The above considerations have been incorporated into our revision, and we believe that the inclusion of these references has substantially enriched the theoretical background and overall quality of our manuscript. Thank you again for your constructive feedback.

46. Aguilar Perez, K. M. et al. Abiotic stress-triggered nanocarriers for seed nanopriming and early-stage plant development. *ACS Nano* **20**, 575-592 (2026).
47. Aguilar Perez, K. M. et al. Tailoring core-shell metal coordination for smart seed coatings in sustainable agriculture. *ACS Appl. Mater. Interfaces* **16,** 65255-65269 (2024).
48. Saleh, N. A. et al. Inhibition of the germination of root parasitic plants by zeolitic imidazolate framework-8. *ChemPlusChem* **90**, e202400457 (2025).
49. Aguilar Perez, K. M. et al. Biomimetic mineralization for smart biostimulant delivery and crop micronutrients fortification. *Nano Lett*. **23**, 4732-4740 (2023).

3. The Mn^{2+}-mediated coordination strategy is effective, but its design rationale would benefit from broader contextualization. A brief acknowledgment of prior metal-coordinated delivery systems used to achieve tunable stability and environmentally responsive release would strengthen the materials design framework.

Our response: Thank you very much for your insightful and constructive comments. We fully agree that connecting our Mn^{2+}-coordination strategy to the broader context of metal-coordination-based delivery systems will significantly strengthen the theoretical framework of our material design. Accordingly, we have revised the "Preparation of CMCS-DK6 hydrogel" section as follows:

The original text: "In this study, CMCS hydrogel, known for its excellent solubility, biocompatibility, and biodegradability, was selected as a carrier to encapsulate the hydrophobic compound DK6, thereby constructing a CMCS-DK6 hydrogel delivery system. This approach aims to enhance its solubility, water absorption, and water retention capabilities, thus improving its drug delivery performance (Fig. 3A)." has been revised to: "Recent studies using systems such as Zn-phenolic networks, Fe-tannic acid shells, and ZIF-8 have achieved controlled release of kinetin, seed coating, modulation of signaling molecules, and co-delivery of nutrients, demonstrating the potential of metal coordination chemistry in constructing tunable agricultural delivery platforms. Building on this, we selected the essential plant micronutrient Mn^{2+} to coordinate with CMCS—a soluble, biocompatible, and degradable polymer—to encapsulate the hydrophobic compound DK6, forming a CMCS-DK6 hydrogel delivery system aimed at improving solubility, water absorption, and retention, thereby enhancing drug delivery performance (Fig. 3A)."

We believe that these revisions, together with the added content in the Introduction, firmly anchor the design rationale of our Mn^{2+}-coordination strategy within a broader academic context, reflecting both the inheritance and advancement of existing knowledge and highlighting the unique innovation of our work in achieving the dual objectives of "delivery" and "nutritional synergy." Thank you again for your valuable comments, which have greatly improved our manuscript.

4. While the pH-dependent swelling and release behavior is clearly demonstrated, its agronomic relevance is not sufficiently discussed. Linking pH responsiveness to realistic soil and rhizosphere conditions would strengthen the translational relevance of the study.

Our response: Thank you very much for your valuable feedback. We agree that while the pH-dependent swelling and release behavior of the hydrogel was clearly demonstrated, the discussion of its agronomic relevance was insufficient. This criticism is well taken and has significantly improved the translational value of our study.

In response to your suggestion, we collected typical paddy soils from six major rice-producing regions in China (Hunan, Jiangsu, Sichuan, Henan, Heilongjiang, and Liaoning), which differ in pH and texture. We measured the 48-h cumulative release profiles of CMCS-DK6 in these soils. The results (Fig. 6A and Table 1, Supplementary) show that the cumulative release rates ranged from 63.5% to 86.1%, and the release kinetics followed a first-order model ($R^2 = 0.94$-0.99) in all soils. Compared with the PBS buffer system, the buffering and adsorption effects in actual soil solutions moderately attenuated the pH-mediated control; nevertheless, the hydrogel achieved effective release in all tested soils. We have also noted in the manuscript that future studies should quantify the influence of soil physicochemical properties on release kinetics to optimize application strategies. Furthermore, when CMCS-DK6 was added to the same six soils at various concentrations and rice was grown for 45 days, rice tillering was promoted to different extents in each soil (Fig. 7, Supplementary), further validating its agronomic effectiveness under diverse field-relevant conditions.

We have added the above data and discussion to the "Release Behavior of CMCS-DK6 Hydrogel Delivery System" section in the revised manuscript, and we have explicitly noted that future work should quantify the influence of soil physicochemical properties on release kinetics. These complementary data clearly demonstrate that, despite the greater complexity of real soil environments compared to buffer solutions, CMCS-DK6 hydrogel can achieve effective and kinetically controllable release in soils of varying pH and texture, which translates into consistent tillering promotion. This strengthens the logical link between pH responsiveness and soil conditions and highlights the agronomic translational potential of the material. We thank the reviewer again for this valuable suggestion.

5. The manuscript repeatedly claims sustainability, low environmental impact, and precision delivery but does not include life-cycle assessment (LCA) or green metrics studies.

Our response: Thank you very much for your valuable comments, which have helped us to more rigorously examine the statements regarding the environmental impact of our manuscript. We fully agree that our initial discussion of "sustainability" and "low environmental impact" was primarily based on the natural origin, biodegradability, and low toxicity of CMCS and Mn^{2+}, and indeed lacked a full life cycle assessment (LCA) or systematic green metrics studies.

Although the present study has mainly focused on the construction, characterization, and bioactivity validation of the materials, in response to your concerns about environmental safety, we have subsequently performed acute toxicity tests of CMCS-Mn and CMCS-DK6 on zebrafish to preliminarily assess their impact on non-target aquatic organisms. The results showed that CMCS-Mn did not cause obvious acute toxicity to zebrafish at concentrations of 20, 15, and 10 μg/mL. For CMCS-DK6, the survival rate of zebrafish was 86.7% at 15 μg/mL, and no obvious acute toxicity was observed at 10 μg/mL. According to the standard toxicity classification (LC_{50} > 10 μg/mL indicates low toxicity), the CMCS-Mn and CMCS-DK6 hydrogels exhibited limited toxicity and only mild effects upon acute exposure in zebrafish. However, if a comprehensive assessment of their environmental safety is required, further and more systematic ecotoxicological studies are still needed in the future. We will present these results in the main text in a new subsection entitled "Zebrafish Acute Toxicity Analysis", along with the methodological description in a new "Zebrafish Acute Toxicity Assay" subsection.

Although these preliminary data support a low ecotoxicity potential, a comprehensive sustainability assessment (e.g., LCA, effects on soil microorganisms, residual dynamics, etc.) is necessary prior to future field applications. We have included these evaluations in our follow-up research plans. At the same time, we will appropriately moderate the statements regarding "sustainability", "low environmental impact", and "precision delivery" in the manuscript to make them more objective and avoid overstatement.

Once again, we sincerely thank you for your valuable feedback, which is of great significance for further improving our research. The revised content will be highlighted in the revised manuscript for your review.

6. No consideration of long-term environmental fate of Mn^{2+} crosslinked hydrogels in soil.

Our response: Thank you for raising this important question. The long-term environmental fate of the Mn^{2+}-crosslinked carboxymethyl chitosan hydrogel in soil is indeed a key scientific issue.

In this study, Mn^{2+} was selected as the crosslinking ion primarily because of its biological function as an essential micronutrient for plants, as well as its favorable crosslinking performance and drug loading efficiency in the CMCS system. We preliminarily propose that, as a natural polymer derivative, CMCS can be gradually degraded by microorganisms in soil, and the released Mn^{2+} may be taken up by crops, thereby reducing the risk of environmental residues.

However, we have not yet systematically evaluated the migration, transformation, and accumulation behavior of Mn^{2+} in soil, nor its long-term effects on soil microorganisms and ecosystems. We acknowledge this as a limitation of the current study. In future work, we will focus on the degradation kinetics of the hydrogel, the transport and transformation patterns of Mn^{2+} in the soil–crop system, and its impact on microbial community structure and function. We also plan to conduct field trials to systematically assess the ecological safety of this material, providing a more solid scientific basis for its environmental friendliness. We thank the reviewer again for this valuable comment.

7. The manuscript presents limited biocompatibility data based on early rice seedling growth, indicating no apparent phytotoxic effects at the tested concentration. However, broader biocompatibility or environmental safety assessments of the designed material are not addressed at the specific tested concentration.

Our response: We greatly appreciate your interest in our work and your constructive comments. We fully recognize the importance of the issue you raised and have accordingly supplemented the manuscript with additional experimental data as suggested.

In the revised manuscript, we have included acute toxicity tests of CMCS-Mn (blank hydrogel) and CMCS-DK6 (drug-loaded hydrogel) on zebrafish to more comprehensively evaluate their biocompatibility and environmental safety. Zebrafish, as a globally recognized model organism, are widely used in aquatic ecotoxicity assessments, and the results provide important insights into the potential ecological risks of the materials in aquatic environments.

The acute toxicity test results showed that after 96 hours of exposure to CMCS-Mn hydrogel (at concentrations of 10, 15, and 20 μg/mL), no mortality or abnormal behavior was observed, with a survival rate of 100%. Following 96 hours of exposure to the drug-loaded hydrogel CMCS-DK6, the survival rate of zebrafish was 86.7% at 15 μg/mL, while no significant abnormalities were observed at 10 μg/mL, indicating that the median lethal concentration (LC_{50}) exceeds 10 μg/mL. According to the toxicity classification criteria specified by the Organisation for Economic Co-operation and Development (OECD) and the Chinese "Guidelines for Environmental Safety Evaluation of Chemical Pesticides" (GB/T 31270.13-2014), this LC_{50} value corresponds to a "low toxicity" rating.

We have presented these results in the main text in a new subsection entitled "Zebrafish Acute Toxicity Analysis", along with the methodological description in a new "Zebrafish Acute Toxicity Assay" subsection. We have also noted that, although the current data support the low toxicity profile and provide an initial assessment of environmental safety, more systematic ecotoxicological studies are still needed to comprehensively evaluate its safety. Once again, we thank you for your valuable comments, which have provided important guidance for further improving the depth and breadth of our study.

8. Ensure consistent use of terminology (e.g., "smart hydrogel," "stimuli-responsive hydrogel," "pH-responsive hydrogel").

Our response: Thank you very much for your valuable comment regarding the inconsistent use of terminology. We have carefully reviewed the entire manuscript and standardized the relevant terms to ensure accuracy and consistency. These revisions include, but are not limited to, changing "stimuli-responsive" to "pH-responsive release" and replacing "smart hydrogel" with "pH-responsive hydrogel."

Throughout the manuscript, we now uniformly use "pH-responsive hydrogel" or "pH-responsive CMCS-DK6 hydrogel" when referring specifically to this hydrogel system. The occasional use of "smart hydrogels" and "stimuli-responsive" in the Introduction has been retained because these refer to general categories rather than our specific material, allowing us to distinguish broad concepts from our particular hydrogel. All changes are marked in the revised manuscript. We thank you again for your careful review and constructive suggestions.

9. Briefly rationalize why the release kinetics follow a first-order model in the context of polymer swelling and diffusion.

Our response: Thank you for your valuable comment. Regarding the reason why the release kinetics of the CMCS-DK6 hydrogel follow the first-order model, we provide a brief explanation based on hydrogel swelling and diffusion theory.

In pH-responsive hydrogel systems, drug release is primarily governed by diffusion driven by a concentration gradient. The first-order model describes a release rate that is proportional to the remaining drug amount. In this study, the release profiles of CMCS-DK6 hydrogels under different pH conditions showed the highest goodness-of-fit to the first-order model (R^2 = 0.91-0.97), indicating that diffusion is the dominant release mechanism. Specifically, under alkaline conditions, the hydrogel network rapidly swells due to enhanced electrostatic repulsion, while Mn^{2+} coordination bonds are partially disrupted by competition from OH^- ions, leading to a loosened polymer network. DK6 molecules can then rapidly diffuse into the medium through hydrated channels. In this process, the drug release rate is linearly dependent on the remaining drug concentration within the gel, which is why the first-order model adequately explains the release behavior of CMCS-DK6 hydrogels under swelling and diffusion. We will further elaborate on this point in the revised manuscript in the "Release Behavior of CMCS-DK6 Hydrogel Delivery System" section by adding relevant descriptions. We hope this explanation addresses your concern. Thank you again for your thorough review.

10. Acknowledge limitations related to field-scale translation, such as soil heterogeneity and long-term stability.
Our response: Thank you for your valuable comment. We fully recognize that soil heterogeneity (e.g., differences in organic matter content, microbial activity, and buffering capacity) and the long-term stability of the formulation under field conditions are important challenges for the scalable application of CMCS-DK6 hydrogels. In this study, we made an initial attempt to address this issue by selecting six typical paddy soils from Hunan, Jiangsu, Sichuan, Henan, Heilongjiang, and Liaoning to evaluate the release behavior of CMCS-DK6 and its effect on rice tillering. The results showed that within 48 h, the cumulative release of CMCS-DK6 in these soils ranged from 63.5% to 86.1%, reaching effective release levels in all cases, and the hydrogel promoted tillering to varying degrees across all tested soils. Notably, compared with the PBS buffer system, buffering and adsorption effects in actual soil solutions indeed attenuated the pH-dependent release profile. Nevertheless, the release kinetics still followed the first-order model (R^2 = 0.94-0.99), indicating that the release rate is proportional to the residual drug amount, providing a theoretical reference for time-based prediction of release behavior in field applications.

We fully agree that the above short-term soil incubation experiments cannot fully capture the complexity of field-scale conditions, including seasonal fluctuations in temperature and humidity, microbial degradation, leaching losses, and the impact of soil spatial heterogeneity on formulation consistency. We have added a clear statement of these limitations in the relevant section of the revised manuscript and noted that future studies should further quantify the influence of soil physicochemical properties on release kinetics and conduct multi-site field trials and long-term stability studies to optimize the application strategy of this hydrogel in real soil environments. Thank you again for your insightful comments, which will guide our future research directions.

11. Some figure captions are generally too brief and do not provide sufficient methodological or statistical context for the figures to be interpreted independently. All quantitative data should be presented as mean ± SD or SEM, with the type of error clearly and consistently specified across all figures.
Our response: Thank you for your valuable comment. We agree that complete figure legends and consistent data presentation are essential for readers to interpret the figures independently. Accordingly, we have carefully revised and supplemented all figure legends throughout the manuscript, explicitly adding necessary experimental details (e.g., number of replicates, statistical tests) and statistical information. In addition, all quantitative data are now uniformly presented as mean ± standard deviation (SD), with the type of error bars and sample size (n) clearly and consistently indicated in each figure and its legend. The specific revisions are highlighted in the revised manuscript. Thank you again for your thorough and constructive feedback.

12. Standardize fonts, font sizes, and line thicknesses across all figures. Use a consistent color scheme for recurring conditions (e.g., DK6, CMCS-DK6, controls) throughout the manuscript. Increase the size and clarity of axis labels, legends, and annotations to ensure readability at print scale.
Our response: Thank you very much for your valuable suggestions regarding the quality of our figures and tables. We fully recognize the importance of consistent formatting and enhanced readability for clearly conveying research findings. In response to your comments, we have systematically optimized all figures and tables throughout the manuscript: we have unified font types, font sizes, and line widths; established a fixed color code for frequently appearing experimental conditions (e.g., DK6, CMCS-DK6, KK094, CK) and applied it consistently across all relevant figures; moderately increased the font size of axis labels and legends, adjusted legend positions to avoid occlusion, and used bold or enlarged fonts for annotations to ensure legibility after printing. All these modifications have been incorporated into the revised manuscript and accompanying figures. We thank you again for your thorough and constructive feedback.

Reviewer #3 (Remarks to the Author):

After careful evaluation of the manuscript titled "pH-Responsive Manganese-Based Carboxymethyl Chitosan Hydrogel: Promoting Rice Tillering and Suppressing Root Parasitic Weeds", we find that the study designs an intelligent carboxymethyl chitosan (CMCS)-based hydrogel delivery system for a novel strigolactone (SL) inhibitor DK6, achieving pH-responsive release and demonstrating excellent performance in promoting rice tillering and suppressing root parasitic weeds. The research spans chemical synthesis, materials science, plant physiology, and computational biology, exhibiting strong innovation and potential application value. However, several aspects could be improved. The following comments are intended to enhance the rigor, completeness, and readability of the article.

1. In the weed germination inhibition assay, a control group with blank hydrogel (without DK6) was not included. This omission makes it impossible to rule out potential effects of the CMCS-Mn carrier itself on germination. It is recommended to supplement the experimental results with a blank hydrogel group.

Our response: Thank you very much for your valuable comment. We fully agree that the absence of a blank hydrogel (CMCS-Mn) control in the weed seed germination inhibition experiments was a critical oversight. In response, we have performed the following additional experiments: blank CMCS-Mn hydrogel groups were included alongside CMCS-DK6, free DK6, and KK094 treatments to evaluate inhibition against three parasitic weed species. The results showed that 100 nM GR24 alone induced germination rates of 67.5%, 95.5%, and 70.9% for *Striga asiatica*, *Phelipanche aegyptiaca*, and *Orobanche cumana*, respectively. Co-treatment of the blank CMCS-Mn hydrogel at various concentrations (80-5 μM) with 100 nM GR24 gave germination rates of 66.5%-67.1%, 93.1%-94.0%, and 69.3%-69.8%, respectively, which were comparable to those of GR24 alone, indicating that the blank hydrogel itself had no significant inhibitory effect on seed germination. We will include these results in the revised manuscript (particularly in the legend of Fig. 6), confirming that the inhibitory activity of CMCS-DK6 primarily stems from the release of DK6. Thank you again for your professional review.

2. The manuscript only evaluates the safety of the hydrogel on rice seedlings and does not address its impact on non-target organisms such as zebrafish. It is recommended to supplement with a simple experiment assessing the effects of the blank hydrogel at different concentrations on zebrafish to preliminarily evaluate its environmental safety.

Our response: We thank you for your careful review and constructive feedback. You pointed out that our current study primarily focuses on the safety evaluation of the target crop (rice), but lacks an assessment of potential effects on non-target organisms such as zebrafish. We fully agree with this valuable comment.

In the present work, we have preliminarily verified the biosafety of the CMCS-DK6 hydrogel on the target crop through rice seed germination and seedling growth assays (Fig. 6H–J). However, as you noted, this does not adequately address its safety to aquatic organisms. Zebrafish, as a widely accepted aquatic model organism, provide a standard and effective approach for evaluating the acute toxicity of chemicals on the aquatic environment.

Therefore, following your suggestion, we have now performed acute toxicity tests of both the blank CMCS-Mn and CMCS-DK6 hydrogels on zebrafish larvae, and we will include these results in the revised manuscript. Our results showed that CMCS-Mn did not cause obvious acute toxicity at 20, 15, and 10 μg/mL. For CMCS-DK6, the survival rate of zebrafish was 86.7% at 15 μg/mL, and no significant acute toxicity was observed at 10 μg/mL. According to the standard toxicity classification (LC_{50} > 10 μg/mL indicates low toxicity), the CMCS-Mn and CMCS-DK6 hydrogels exhibited limited toxicity and only mild effects upon acute exposure in zebrafish. However, if a comprehensive assessment of their environmental safety is required, further and more systematic ecotoxicological studies are still needed in the future. We will present these results in a new subsection "Zebrafish Acute Toxicity Analysis" in the main text, and include a "Zebrafish Acute Toxicity Assay" subsection in the Methods. Additionally, we will note that, while current data support a low-toxicity profile and provide an initial environmental safety assessment, more systematic ecotoxicological studies are warranted in the future.

We believe that adding this non-target biosafety evaluation will enhance the scientific rigor and completeness of our study. Once again, we sincerely thank you for your insightful review, and we look forward to your further guidance.

3. The authors selected CMCS as the carrier based on its biocompatibility and modifiability but did not explain why manganese ions were preferentially chosen as the crosslinking agent. A brief justification should be added.

Our response: Thank you for your valuable comment. Regarding the rationale for selecting Mn^{2+} as the crosslinker for the CMCS hydrogel, we provide the following clarification.

In the "Preparation" section of the original manuscript, we briefly reported a screen of several plant-essential metal ions (Zn^{2+}, Cu^{2+}, Mn^{2+}, Fe^{2+}, Fe^{3+}). Among these, Cu^{2+} and Fe^{2+} formed precipitates when mixed with the DK6-containing emulsion and were unsuitable for the system. Among Zn^{2+}, Mn^{2+}, and Fe^{3+}, which all yielded homogeneous systems, the Mn^{2+}-crosslinked CMCS-DK6 hydrogel exhibited the best drug loading performance (drug loading 26.9%, encapsulation efficiency 79.6%; see Fig. 3C), which was the direct and primary reason for choosing Mn^{2+}. In addition, Mn^{2+} is an essential micronutrient for plants, involved in key physiological processes such as photosynthesis and enzyme activation. Its use avoids the introduction of non-essential or potentially toxic metal ions, aligning with the concept of green agricultural materials. Thus, the selection of Mn^{2+} was a comprehensive decision based on experimental results and biocompatibility. We thank you again for your thorough review.

4. Given that manganese (Mn) is an essential micronutrient, the release of Mn^{2+} from the hydrogel may confer an additional growth-promoting effect on plants. It is recommended to briefly address this point in the Discussion section.

Our response: Thank you for your insightful comment. We agree that as an essential plant micronutrient, Mn^{2+} released from the hydrogel may provide additional benefits for plant growth, and we appreciate your suggestion to briefly address this in the Discussion.

In our study, the CMCS-DK6 hydrogel uses Mn^{2+} as the crosslinking ion, enabling sustained release of both DK6 and Mn^{2+}. Supplementary experiments showed that rice seeds treated with CMCS-DK6 germinated slightly earlier than the control group, and by day 5 post-germination, seedling height and fresh weight were significantly higher in the treatment group (Fig. 9G, H, Supplementary Fig. 8). These results collectively suggest that the Mn^{2+}-based CMCS-DK6 hydrogel may have potential as a crop nutrient supplement (Xu et al., 2021).

Accordingly, we will add a subsection entitled "Nutritional function of hydrogel for rice growth" in the revised manuscript, highlighting that this hydrogel system not only delivers DK6 in a targeted manner but also serves a micronutrient supplementation function. We thank the reviewer again for this valuable suggestion, which will help more comprehensively elucidate the multiple mechanisms by which the CMCS-DK6 hydrogel promotes crop growth.

5. In Figure 1A, "X=C or N" could be revised to "X=C or N, Y= N" or simply removed. Furthermore, in the METHODS section, Equation 8 is followed directly by Equation 10. Please check whether Equation 9 is missing or if the numbering is incorrect.

Our response: Thank you for your thorough review. Regarding the annotation "X=C or N" in Fig. 1A, we have adopted your first suggestion and revised it to "X=C or N, Y=N" to more accurately reflect the atomic differences. As for the inconsistent numbering of equations in the Methods section, upon verification we found that the numbering after Equation 8 was incorrect, and we will renumber all subsequent equations in the revised manuscript. We apologize for these oversights and thank you again for your rigorous correction.

6. The heading "Design, Synthesis, and Identification of Small Molecule DK6" in the RESULTS AND DISCUSSION section is incorrectly formatted. Additionally, would it be more appropriate to present the bioactivity results in the order of compounds DK1 to DK32?

Our response: Thank you very much for your thorough review. We apologize for the minor issue regarding the heading format in the "Results and Discussion" section and will standardize it in the revised manuscript.

Regarding the organization of the bioactivity section, we fully understand the suggestion to present the compounds in the order DK1–DK32. After careful consideration, we chose to first present the overall activity data (Table 1) followed by the structure-activity relationship (SAR) analysis for three main reasons. (1) To highlight the SAR as the main thread, presenting the overall trend first to help readers focus on the contributions of key structural features (e.g., triazole ring, halogen substituents) to activity. (2) To align with the research logic of "design, synthesis, screening, and optimization", providing an overview of activity before delving into the inhibition and tiller-promoting effects of DK6. (3) To avoid redundancy—the physicochemical data for all compounds are fully provided in the Supporting Information, and describing each sequentially in the main text would dilute the core focus. We believe that the current structure more effectively guides readers through the SAR

and transitions smoothly to the subsequent functional material and mechanistic studies. Thank you again for your expert guidance.

7. In the METHODS section, "Synthesis of Target Compounds DK1-DK33." is mentioned, but compound DK33 is not referred to anywhere else in the manuscript. Please check and revise accordingly. Also, in the "Seed Germination Assay" subsection, the citation for reference 15 is incorrectly formatted and should be corrected.
Our response: Thank you very much for your thorough review. We have carefully checked and corrected the two issues in the Methods section. First, "DK33" in "Synthesis of Target Compounds DK1–DK33" was a typo and should be DK1–DK32, this has been corrected accordingly. Second, the citation format of reference [15] in the "Seed Germination Assay" has been adjusted to conform to the journal's style, and other citations have been rechecked. We apologize for these oversights and thank you again for your professional guidance. We sincerely apologize for these inaccuracies resulting from our oversight. Your professional review has significantly improved the accuracy of our manuscript. Thank you for your time and effort, and we look forward to your further guidance.

8. Abbreviations should be defined upon first use (e.g., FT-IR). Some sections use abbreviations directly without definition. Additionally, there are minor grammatical errors and unclear expressions, such as "tetrahydropsyrole ring," which should be standardized to "tetrahydropyrrole ring."
Our response: Thank you very much for your thorough review. Based on your comments, we have made the following corrections: the full name "Fourier transform infrared (FT-IR)" has been added at its first appearance; "SEM" has been changed to "scanning electron microscopy (SEM)"; and the spelling error "tetrahydropsyrole ring" has been corrected to "tetrahydropyrrole ring" throughout the manuscript, with all related terminology rechecked. We apologize for any inconvenience caused by these oversights. Thank you again for your valuable input.

9. The sterilization protocol for parasitic weed seeds involves treatment with 1% NaClO for 2 minutes, followed by 75% ethanol for 2 minutes. Compared to some established methods, this treatment duration is relatively short. Please provide justification or cite relevant literature to support the effectiveness of this protocol.
Our response: Thank you for your comment. The disinfection protocol was not arbitrarily shortened; rather, it was based on a recently published and well-established method. This approach effectively eliminates surface microorganisms while minimizing any potential adverse effects of the disinfectant on seed viability, thereby ensuring the reliability of subsequent germination experiments.

In the reference (Int. J. Mol. Sci. 2023, 24(12), 9967), the "Seed Germination Bioassay" section describes the following procedure: "The seeds were sterilized with 1% NaClO for 2 min, then with 75% ethanol for 2 min, and finally rinsed three times with sterile distilled water." As this method has been published in a reputable journal, it demonstrates the validity of this protocol within the relevant field. We thank the reviewer again for the valuable feedback.

10. Please specify the instrument or tool used to control the droplet size of the emulsion during hydrogel preparation to support the reproducibility of the experiment.
Our response: Thank you very much for your thorough review and insightful comments. Your suggestion regarding droplet size control during hydrogel preparation is critically important for ensuring material uniformity and batch-to-batch reproducibility, and we fully agree. As described in the revised manuscript, the pre-prepared emulsion was transferred into a 10 mL syringe equipped with a needle and then added dropwise into a 0.2 M metal ion solution to form the hydrogels. In addition, we have verified the size uniformity and preparation reproducibility through multiple independent batch experiments and subsequent characterizations. We sincerely appreciate your constructive feedback, which has helped us refine the relevant methodological details in the manuscript.

11. Figure 6: The germination inhibition assay for parasitic weeds should specify whether a standalone GR24 treatment group was included, and at what concentrations it was applied.
Our response: Thank you very much for your comments. In all parasitic weed seed germination inhibition experiments, we have established a complete set of controls.

As shown in Fig.6, treatment with 100 nM GR24 alone was used as the positive control. This concentration, determined based on standard practices in the field and our preliminary experiments, effectively activates the strigolactone signaling pathway and induces seed germination. Under our experimental conditions, 100 nM GR24

induced germination rates of 67.5%, 70.9%, and 95.5% for *Striga asiatica*, *Phelipanche aegyptiaca*, and *Orobanche cumana*, respectively, providing a favorable signaling background for antagonist testing.

In data analysis, the germination rate of this positive control was defined as "100% GR24-induced germination" (i.e., 0% inhibition). The inhibition rate of each test compound was calculated by comparing the reduction in germination of the "test compound + 100 nM GR24" treatment relative to the positive control, thereby accurately assessing the compound's specific ability to antagonize GR24 signaling rather than non-specifically suppressing basal seed viability. Following your suggestion, we have added the above information to the legend of Fig. 6. Once again, we thank you for your valuable input.

12. The explanation of the pH-responsive mechanism of the hydrogel is clear but could be enhanced by adding a schematic diagram in the Supporting Information illustrating the competition between COOH ionization and Mn^{2+} coordination.

Our response: Thank you for your careful reading and valuable suggestions. We fully agree with your perspective. Although the original manuscript already described the pH-responsive behavior of the CMCS-DK6 hydrogel through textual explanations and experimental data on swelling and release, we recognize that a clear schematic would help readers intuitively understand the dynamic molecular processes. Therefore, we plan to include a schematic (Fig. 5) in the Supplementary to visually illustrate the structural evolution and drug release mechanism of the hydrogel network under different pH conditions.

We believe that adding this schematic will make the mechanistic explanations in sections such as "pH-Dependent Swelling of CMCS-DK6 Hydrogel" and "Release Behavior of CMCS-DK6 Hydrogel Delivery System" more complete and accessible. In the revised manuscript, we will cite the schematic accordingly and provide a detailed caption in the Supplementary Information. We thank you again for your insightful suggestion and look forward to your further guidance.

13. What was the rationale for selecting 6 hours as the key time point for GO/KEGG analysis?

Our response: Thank you for your valuable comment regarding the selection of the 6-hour time point for GO/KEGG analysis. We provide the following explanation.

In our transcriptomic analysis, we included three time points (3, 6, and 12 hours) after DK6 treatment to systematically capture dynamic changes in gene expression. Statistical analysis of differentially expressed genes (DEGs) revealed that the 6-hour treatment group exhibited the highest number of DEGs (3,718), significantly more than the 3-hour (2,697) and 12-hour (2,616) groups, indicating that the transcriptional response was most active at this time point. Furthermore, Venn diagram analysis showed that only 825 DEGs were common to all three time points, whereas 1,691 DEGs were unique to the 6-hour time point. These results suggest that 6 hours represents a critical window for DK6-induced transcriptional regulation, with gene expression changes at this time point showing stronger temporal correlation with subsequent phenotypic outcomes.

Therefore, we selected the 6-hour samples for GO and KEGG enrichment analysis to gain deeper insight into the key metabolic and signaling pathways affected by DK6 during the early response. This choice was based on data significance and the stage-specific characteristics of the biological response, rather than overlooking the other time points. We believe it helps clarify the early mechanism of action of DK6. Thank you again for your insightful comment.

14. Please meticulously check the formatting of all references throughout the manuscript to ensure full compliance with the journal's requirements.

Our response: Thank you for your careful review and for highlighting the importance of proper reference formatting. In response to your comment, we have systematically checked and uniformly corrected all references throughout the manuscript to ensure full compliance with the journal's requirements. We apologize again for the few formatting inconsistencies in the initial manuscript and sincerely thank you for your rigorous and thorough review, which has greatly improved the formal quality of our work.

Reviewer #4 (Remarks to the Author):

Dear Authors,

It was a pleasure reading your manuscript; I hope my comments are useful and help improve your manuscript. Please let me know if anything is not clear.

1. The sentence "Indole derivatives exhibit potential for plant growth regulation due to their ability to mimic peptide conformations and reversibly bind target enzymes" is a very close paraphrase of reference 46, which is not a primary source. I would suggest providing more context using primary articles to better understand why indole derivatives have good potential. Reversibly binding target enzymes is a property many non-indole derivatives also have.

Our response: Thank you for your valuable comment. We agree that the original statement—"Indole derivatives exhibit potential for plant growth regulation due to their ability to mimic peptide conformations and reversibly bind target enzymes"—was insufficiently comprehensive. Following your suggestion, we have re-examined the original literature, which indicates that the advantages of indole derivatives include structural modifiability, the ability to mimic the active conformation of endogenous hormones, specific interactions with multiple receptors, and favorable metabolic stability. Accordingly, we have revised the sentence to: "Indole derivatives exhibit plant growth regulation potential, attributed to the structural diversity of their core skeleton, their ability to mimic the active conformations of various endogenous hormones, specific interactions with multiple receptors, and favorable metabolic stability" The corresponding text and reference have been updated in the manuscript. We thank you again for your thorough review.

2. The initial experiment evaluating the tillering-promoting activity of synthesized compounds in the presence of GR24 appears to be lacking a GR24-only control. Also, the figure legend states compounds were used at a concentration of 10 μM, while the text says 80 μM. Which one is it? What was the concentration of GR24 used for co-treatments? What is CK- should it be spelled out on the legend? It seems to me these are two separate experiments, one is germination inhibition while the other is tillering evaluation. It is unclear if this is true and should be better specified. How were IC50s calculated using a single dose rather than an inhibitor titration? The methods section talks about this, but the figures do not reflect a titration. Furthermore, Figure S3A shows 3 different concentrations were used in the tillering assay (1, 5, and 15 μM); it would not be possible/reliable to obtain the IC50s reported in the text using only these concentrations.

Our response: We sincerely thank the reviewer for the valuable comments and careful corrections. We apologize that the description of the relevant experiments in the original manuscript was unclear and incomplete, which may have led to misunderstandings. Based on the suggestions, we have addressed each point as follows:

These two experiments were conducted independently. The first was a germination inhibition assay of root-parasitic weed seeds, designed to evaluate the inhibitory effect of the compounds themselves on seed germination. Therefore, co-treatment with the compound and 100 nM GR24 was applied to simulate a competitive inhibition scenario of strigolactone (SL) signaling. This concentration was chosen based on standard practices in the field and our preliminary results, as it is sufficient to activate the SL signaling pathway in parasitic weed seeds and induce germination, thereby providing a clear baseline for assessing the inhibitory activity of the test compounds. The second experiment was a rice tillering evaluation, aimed at assessing the regulatory effect of the compounds on rice tillering without exogenous GR24. In this case, the CK control referred to an aqueous solution containing the same concentration of DMSO as in the treatment groups (with the final DMSO concentration well below 0.1%).

Regarding the inconsistency in compound concentrations used: in the germination inhibition assay, the initial screening concentration for IC_{50} determination was 80 μM. Subsequently, a concentration gradient (80, 40, 20, 10, and 5 μM) was applied to accurately determine the IC_{50} value. In the rice tillering evaluation, the initial screening concentration was 10 μM, and only the candidate compound DK6 was further tested using a concentration gradient (1, 5, 15 μM).

We apologize that the descriptions in the main text and figure legends were not sufficiently clear, which may have caused confusion. The corresponding figure legends and methods have now been corrected. We greatly appreciate the reviewer's time and professional insights.

3. Were other varieties of Striga (i.e., hermonthica) tested in the germination suppression assay? What about a non-Striga control expressing the targeted receptor/its absence to make sure the reason for germination was its

engagement?
Our response: Thank you for your valuable comments. In this study, the germination inhibition assay was conducted only on *Striga asiatica* and not on other *Striga* species such as *S. hermonthica*. The appearance of *S. hermonthica* in the abstract was a clerical error, for which we apologize. We will correct this in the revised manuscript to ensure accuracy.

Regarding validation of receptor involvement, we fully agree on the importance of using control lines that do not express the target receptor to confirm target engagement. At present, we have not performed control experiments using *Striga* mutants that lack the target receptor (e.g., *Sh*HTL7). To address this, we have performed microscale thermophoresis (MST) to investigate the binding ability of DK6 to SL receptors.

Due to the unavailability of the rice SL receptor *Os*D14 and the *Striga* SL receptor *Sh*HTL7 proteins, we tested the binding of DK6 to the *Arabidopsis* SL receptor *At*D14. The MST results gave a dissociation rate constant (K_d) of 3.72 ± 1.509 μM for DK6 binding to *At*D14, providing direct biochemical evidence for an interaction between DK6 and *At*D14. Coupled with the genetic observation that DK6 activity is abolished in the *max2-1* mutant, we propose that DK6 likely acts through the SL signaling pathway. Given the high sequence homology and conserved binding pocket structures among *At*D14, *Sh*HTL7, and *Os*D14, these data support the notion that DK6 can bind to SL receptors, although direct binding to *Os*D14 and *Sh*HTL7 remains to be confirmed.

Thus, generating receptor knockout/knockdown lines in *Striga* for direct validation is an ideal direction for future studies, and we will consider this suggestion in our subsequent work. We thank you again for your valuable input, which will help us further improve the manuscript.

4. There is a typo in the subtitle "Release Hehavior of CMCS-DK6 Hydrogel Delivery System"
Our response: Thank you very much for your thorough review and for pointing out a spelling error in our manuscript. We apologize for this oversight during proofreading. The subheading "Release Hehavior of CMCS-DK6 Hydrogel Delivery System" indeed contains a typo, the correct spelling should be "Behavior". This error was introduced during the final text processing and regrettably escaped our last check. The subheading has now been corrected to "Release Behavior of CMCS-DK6 Hydrogel Delivery System" in the final version of the manuscript. We have also rechecked the entire text to eliminate any such mistakes. We are grateful for your careful reading and valuable correction, which have significantly helped improve the quality of our work.

5. The encapsulation capacity seems rather low; this is fine, as it is an exploratory article, but it would be valuable if potential approaches to improve this were included in the discussion.
Our response: We sincerely thank the reviewer for the careful assessment and constructive comments. We fully agree that the encapsulation efficiency could be further improved and appreciate the suggestion to include potential optimisation strategies in the Discussion.

In the current study, the CMCS-DK6 hydrogel achieved an encapsulation efficiency of 79.6%, which, while sufficient to support the subsequent biological evaluations, still leaves room for improvement. Accordingly, we have added the following text to the Discussion section of "Preparation of CMCS-DK6 Hydrogel": "Nevertheless, there remains room for further improvement in encapsulation efficiency. Future efforts could focus on optimising emulsification-crosslinking parameters to enhance the loading capacity for small molecules, as well as introducing co-crosslinking systems or multi-responsive architectures to improve the stability of the encapsulated payload, thereby achieving higher drug loading efficiency."

We thank the reviewer again for this valuable suggestion, which we believe has strengthened the completeness of the manuscript.

6. The explanation of the hydrogel being more efficacious at inhibiting parasitic germination than DK6 alone "hydrogel matrix likely enhances DK6 bioactivity, potentially due to its excellent biocompatibility and sustained-release properties, which stabilize DK6 interaction with weed seeds and prolong its duration of action" seems too vague to me. Often, free drugs can be more active, due to exposure, pharmacokinetics, etc., so I am wary of this explanation. The figure legend lacks enough explanation to understand properly how this experiment (Fig. 6) was conducted. Unclear if proper controls (hydrogel only, GR24, what is the baseline of germination in the absence of compound (100%?)) were included. Also, it is unclear what the 0 μM treatment in Figure 6A means, but visually, it looks like more seedlings are germinating in the CMCS-DK6 treatment than on the DK6-only treatment. Does this not contradict the proposal that CMCS enhances activity?

Our response: Thank you for your thorough and constructive comments on Fig. 6 and its interpretation. We have addressed each point as follows and revised the manuscript accordingly.

Ambiguous wording regarding "hydrogel enhancing DK6 bioactivity", we fully agree that free drugs typically show higher apparent activity, and the sustained-release nature of a hydrogel generally does not truly enhance intrinsic activity. Re-examination of the data showed that the IC_{50} of CMCS-DK6 against *Striga asiatica* (1.72 μM) was slightly lower than that of free DK6 (2.15 μM). This modest difference is more likely attributable to the sustained-release effect and protective action of the hydrogel (e.g., reducing degradation or non-specific adsorption), which improve apparent drug bioavailability. We have revised the manuscript accordingly and toned down the claim of "enhanced activity".

Completeness of controls and figure legends, we apologize for this oversight. The positive control was 100 nM GR24 alone (defined as 100% germination and 0% inhibition). This concentration, chosen based on standard practices in the field and preliminary experiments, sufficiently activates the strigolactone signaling pathway in parasitic weed seeds to induce germination, providing a clear benchmark for assessing inhibitory activity. The inhibition rate for each test compound was calculated by comparing the germination reduction of "test compound + 100 nM GR24" with that of the positive control. Co-treatment of the blank hydrogel (CMCS-Mn) with GR24 gave germination rates comparable to GR24 alone, indicating no inhibitory effect. In Fig. 6A, "0 μM" refers to a DK6 or CMCS-DK6 concentration of zero (i.e., only 100 nM GR24). In the rice experiment, the control (CK) was an aqueous solution containing <0.1% DMSO. The figure legends have been substantially expanded in the revised manuscript.

Visual difference in the 0 μM treatment groups in Fig. 6A, upon checking the raw data, this visual difference resulted from normal fluctuations in GR24 induction efficiency and minor errors in data normalization. Statistical analysis revealed no significant difference in germination rates between the two groups. In the revised manuscript, the treatment groups are clearly labeled as "GR24 + DK6" and "GR24 + CMCS-DK6", and the figure legend now includes statistical information indicating that this difference is not statistically significant and does not affect the dose-dependent inhibition trend or the reliability of the IC_{50} values.

Once again, we thank you for your valuable input, which has substantially improved the rigor and transparency of our study.

7. "hydrogel matrix likely enhances DK6 bioactivity, potentially due to its excellent biocompatibility and sustained-release properties, which stabilize DK6 interaction with weed seeds and prolong its duration of action" - the data shown on graph 6E does not agree with this statement. It is also unclear what the small a, b, and c on top of each bar represent.

Our response: Thank you for your careful observation of the data in Fig. 6E and your query regarding the statistical annotations. We respond as follows.

In Fig. 6E, the tiller-promoting effect of CMCS-DK6 on rice at 10 μM was only marginally better than that of free DK6, with only a minor difference. Therefore, our previous statement that "the hydrogel enhances the bioactivity of DK6" was too broad. The core value of the hydrogel lies in improving the water solubility of hydrophobic pesticides, enabling controlled release, and prolonging the effective duration, rather than significantly enhancing intrinsic activity. The slight advantage of CMCS-DK6 in Fig. 6E is more likely attributable to a sustained-release effect that compensates for rapid depletion of DK6, rather than true "activity enhancement." We have revised the relevant statements in the manuscript to indicate that the two treatments show comparable effects, with the modest trend in favor of CMCS-DK6 being explained by its sustained-release property.

We apologize for the omission of statistical annotations in the figure legend. The different lowercase letters (a, b, c, etc.) above the bars indicate significant differences between treatment groups at the $p < 0.05$ level (Tukey's multiple comparison test). We will clearly state in the figure legend: "Statistical significance among means was determined using one-way ANOVA followed by Tukey's multiple comparison test. Different letters indicate statistically significant differences ($p < 0.05$)". We thank you again for your rigorous review.

8. "Based on the established mechanism that SL inhibits hypocotyl elongation in a MAX2-dependent manner, we hypothesized that DK6 might function as an SL signaling inhibitor" this section could benefit from a reference.

Our response: Thank you for your thorough and insightful review. Your suggestion to add a reference supporting the established mechanism that strigolactones inhibit hypocotyl elongation in a MAX2-dependent manner is well taken, as it will enhance the rigor and traceability of our discussion. In the revised manuscript, we will include a

citation of a key study that originally established this pathway (e.g., Umehara et al., Nature 2008) in the relevant section (Analysis of *A. thaliana* Hypocotyl Elongation), thereby providing a more complete justification for our hypothesis. We appreciate your valuable input and will address it in the revision.

9. For the hypocotyl elongation assays: Graph 7C shows GR24-treated seedlings had a hypocotyl of ~0.7 cm, while DK6-treated seedlings at 5 µM (7A) had a length of 0.8 cm. When the 2 are combined in Graph 7B, the length is 0.6 cm. However the claim in the text is "In the presence of GR24 (3 µM), both DK6 and KK094 effectively reversed the GR24-induced suppression of hypocotyl elongation". This is not supported by the data shown. The a/b labels on the graphs are again quite confusing.

Our response: Thank you for your thorough review and valuable comments. Regarding the interpretation of the hypocotyl elongation assay data, we would like to provide the following clarification: Fig. 7B presents results for wild-type *Col-0* treated with a fixed concentration of GR24 (3 µM) in combination with varying concentrations of DK6. The hypocotyl length observed with 0 µM DK6 + GR24 (i.e., GR24 alone) is compared with that of 5 µM DK6 + GR24, which shows an increase in hypocotyl length. This result indicates that DK6 effectively reverses GR24-induced inhibition of hypocotyl elongation, consistent with the description in the main text.

The value of "~0.7 cm for GR24 treatment" mentioned by the reviewer likely corresponds to the GR24 treatment in the *max2-1* mutant background shown in Fig. 7C. As this mutant is insensitive to GR24, its hypocotyl length (~0.7 cm) exceeds that of GR24-treated wild type; however, this value cannot be directly used to assess the reversal effect of DK6 in the wild-type background. The conclusion regarding reversal of inhibition is based on the comparison within the same genetic background (wild type) in Fig. 7B, i.e., 0 µM DK6 + GR24 versus 5 µM DK6 + GR24.

Regarding the a/b lettering on the graphs, these letters denote statistically significant differences among means as determined by one-way ANOVA followed by Tukey's multiple comparison test ($p < 0.05$). Different letters indicate statistically significant differences between treatments, whereas identical letters indicate no significant difference. We will clarify the meaning of this notation in the revised figure legend to avoid ambiguity. We thank you again for your rigorous evaluation and will refine the figure annotations accordingly to ensure clear and precise presentation of the data.

10. "In summary, DK6 likely acts by specifically inhibiting SL signal transduction, thereby relieving its suppressive effect on hypocotyl elongation, in a MAX2-dependent manner" I would not conclude this from the data presented. Additionally, no direct test against a SL receptor was performed, so the conclusion that DK6 acts by specifically inhibiting SL signal transduction seems far-fetched.

Our response: We Thank you very much for your thorough review and constructive comments. We fully agree that our previous conclusion — that "DK6 acts by specifically inhibiting SL signal transduction" — was insufficiently supported by data, particularly lacking direct evidence of SL receptor binding.

To address this, we have now performed microscale thermophoresis (MST) experiments to directly measure the binding affinity between DK6 and the *Arabidopsis* SL receptor *At*D14. The results show that DK6 binds *At*D14 with a dissociation rate constant (K_d) of 3.72 ± 1.509 µM, while the control compound KK094 exhibits a K_d of 10.55 ± 3.508 µM, indicating a strong direct interaction between DK6 and the SL receptor (see revised Fig. 7D and Methods).

Based on this new evidence, we have carefully revised the relevant conclusion in the manuscript. Revised conclusion: "Combining genetic evidence with MST binding assays, DK6 likely disrupts SL signaling through direct binding to the SL receptor *At*D14, thereby relieving hypocotyl elongation suppression in a MAX2-dependent manner."

We thank you again for raising this critical point, which has substantially improved the rigor and scientific validity of our conclusions. We believe the revised manuscript now presents stronger data and more precise claims. We look forward to your further guidance and appreciate your time and expertise.

11. The statement "the dual anchoring provided by a hydrogen bond with SER-97 (2.7 Å) and hydrophobic interactions with VAL-144 and PHE-195 is insufficient to match the stability of DK6" is unclear. KK094 is a validated ligand, saying that the interactions it forms with certain residues are no match for those interactions predicted for DK6 is a bit confusing.

Our response: Thank you very much for your valuable comments. We agree that the original wording was unclear and potentially misleading, and we apologize for this. The sentence could be misinterpreted as unduly dismissing

KK094, a well-validated active SL receptor antagonist whose binding mode on D14 has been established in previous studies. Our computational comparison was intended to objectively quantify the differences between DK6 and KK094 in terms of binding energy, hydrogen bond network, and hydrophobic burial, rather than to question the validity of KK094 as a ligand. To express this more accurately and professionally, we have revised the text accordingly (see manuscript- track change). We have removed the subjective comparative phrasing such as "insufficient to match the stability of DK6" and replaced it with a more objective description of "fewer interactions" and "higher calculated binding energy." Thus, we respect the established role of KK094 as a validated ligand while clearly presenting the advantages of DK6 in the current computational models. We thank you again for your careful review, which has helped improve the rigor and clarity of our manuscript. We hope that these revisions meet with your approval.

12. The figures for DK6 docking are too small or low quality for me to provide an informed opionion on them (characters are distorted). PDB code 7SNU is talked about in the data availability section, but no reference to that paper was made.
Our response: Thank you for your valuable comments. In response to the issues you raised: first, regarding the insufficient resolution of the molecular docking figures, we have replaced them with high-resolution images and also updated other low-resolution images throughout the manuscript. Second, concerning the citation of PDB code 7SNU, the corresponding literature (Xu et al., Nat. Commun. 2018, 9, 3947) has been cited in the "Molecular Docking" subsection of the Methods. To make this clearer, we will also include this reference in the "Data availability" section. We thank you again for your thorough review.

13. Some residues in the text have an unclear numbering format, i.e., PHE-A28 or VAL-A219. What does the character preceding the number mean?
Our response: Thank you for your correction. As you noted, the "A" in "PHE-A28" and "VAL-A219" refers to the chain identifier in the PDB protein structure; however, we inadvertently failed to delete this letter during revision, leading to inconsistent formatting. We apologize for this oversight. We have now removed all chain identifiers from residue numbers throughout the manuscript, standardizing them to "PHE-28", "VAL-219" and so forth, and have verified consistency across the text. Thank you again for your thorough review.

14. I am unconvinced a binding difference of 0.2 kcal/mol is truly reflective of a "significantly stronger binder with superior binding affinity". The score difference is higher for OsD14, however the parasitic receptor ShHTL7 shows pretty much identical binding energies, so it is unclear why DK6 performs better than KK094. Is it possible it is not actually binding to ShHTL7 but rather to a different receptor?
Our response: Thank you for your thoughtful comments. We agree that a docking score difference of only 0.2 kcal/mol alone is insufficient to strongly support the conclusion of "significantly stronger binding affinity". It should be noted that in the original manuscript, docking scores were not used as the primary evidence; rather, we focused on multi-dimensional validation:

Predictions from molecular docking and dynamics simulations: The 100-ns MD simulations showed that, compared to KK094, the DK6–*Sh*HTL7 complex exhibited faster convergence of RMSD with smaller fluctuations, lower RMSF of key residues, and a more stable hydrogen bond network. Dynamic stability is more indicative of true binding behavior than docking scores alone.

Biological functional inference, DK6 effectively inhibits Striga seed germination, and *Sh*HTL7 is one of its key SL receptors. We therefore hypothesize that DK6 may act primarily through this receptor, although involvement of other *Sh*HTL isoforms cannot be excluded.

Additional experimental evidence, in the revised manuscript, we have added microscale thermophoresis (MST) experiments. Since *Sh*HTL7 and *Os*D14 proteins were not available, we measured the binding affinity of DK6 to the *Arabidopsis* *At*D14 receptor (K_d = 3.72 ± 1.51 μM, vs. 10.55 ± 3.51 μM for KK094). Given the high sequence homology and conserved binding pockets among *At*D14, *Sh*HTL7 and *Os*D14, this provides direct experimental evidence that DK6 targets SL receptors.

Taken together, the docking and MD simulations, MST data and bioactivity results indicate that DK6 is a potential modulator of the SL pathway. Nevertheless, we have clearly stated that direct binding of DK6 to *Sh*HTL7 still requires further confirmation (see MST analysis section). To avoid misunderstanding, the revised manuscript has toned down the relevant claims and incorporated these clarifications. We thank you again for your rigorous review.

15. Graphs 8B and 8C show that the Rg and SASA parameters for all 4 systems evaluated are pretty much identical. However, the RMSD fluctuation shown on 8A does appear to be higher for ShHTL7-KK094 than all the other systems. Are these 3 graphs from the same simulations? Also, there is no mention in the text or methods of what residue entities were analyzed to quantify RMSD, and the visualized trajectories are not included, so it is impossible to assess what 8A is actually plotting. Furthermore, the text claims DK6 forms more H-bonds with the 2 proteins, but the graphs in 8E do not show that, except for the L7-KK094 system. If the claim is that DK6 presents less fluctuation within the pocket, why would there be more interactions at different points in the simulation, instead of more stability (like that one shown in the ShHTL7-DK6 system)? I would encourage the authors to discuss their findings in the context of previous literature using MD simulations for Striga inhibitors and chemical discovery.

Our response: Thank you for your thorough review and valuable comments. Regarding Fig.8 and the related molecular dynamics simulations, we provide point-by-point responses below.

In Fig. 8B-C, the Rg and SASA of the *Sh*HTL7 – KK094 complex are similar to those of the DK6 complex, yet its RMSD in Fig. 8A shows larger fluctuations. This is because KK094 binding does not markedly alter the overall protein folding state (as reflected by similar Rg/SASA), but induces greater local conformational drift, leading to increased global RMSD. This observation is consistent with the larger RMSF fluctuations of binding-pocket residues shown in Fig. 8D.

Regarding the object of RMSD analysis, we apologize for the lack of clarity. We would like to clarify that RMSD analysis was performed on the backbone atoms of the entire protein to measure the deviation of the overall complex conformation from the initial structure. As detailed in the Methods section, we used the main-chain atoms of the full amino acid sequence, took the energy-minimized initial structure as the reference, and calculated RMSD values from the simulation trajectories using GROMACS.

In the molecular docking, DK6 formed two hydrogen bonds with OsD14, while KK094 formed one, representing only a small difference; the hydrogen bond analysis (Fig. 8E) also shows minimal difference. For the *Sh*HTL7 system, the numbers of hydrogen bonds formed by DK6 and KK094 were comparable. Therefore, based on the present docking and molecular dynamics results, DK6 exhibits only slightly better dynamic stability metrics, rather than universally stronger hydrogen bonding or higher binding affinity across all evaluated parameters. Although a modest advantage is observed, the overall difference between the two compounds is minor, and the manuscript has been revised accordingly.

Upon receptor binding, DK6 itself shows smaller fluctuations (i.e., a more stable ligand conformation), but this does not imply a reduced frequency of interactions with surrounding residues. On the contrary, because the binding mode is more complementary, DK6 forms more persistent non-bonded interactions (e.g., hydrogen bonds, hydrophobic contacts) with pocket residues, collectively resulting in a more favorable binding energy and lower RMSF. We thank you again for your in-depth review and welcome further discussion to improve the manuscript.

16. Most of the section titled "Analysis of SL Pathway and Genes Related to Rice Tillering" is hypothetical and relies on the assumption that DK6 directly interacts with a strigolactone receptor in a parasitic plant. I would suggest removing phrases such as "providing molecular-level confirmation of the pharmacological activity of DK6 as an inhibitor of the SL signaling pathway" unless direct evidence is presented. Framing it as a discussion and further directions would be adequate.

Our response: Thank you for your rigorous feedback. We fully agree that our original inference regarding direct interaction between DK6 and SL receptors in parasitic plants lacked direct experimental evidence. Accordingly, we have added the following in the revised manuscript: microscale thermophoresis (MST) experiments were performed to determine the binding affinities of DK6 and the control compound KK094 to the *Arabidopsis* SL receptor *At*D14. The results show that DK6 exhibits superior binding affinity, providing molecular evidence that DK6 directly targets an SL receptor. In addition, RT-qPCR validated the expression changes of key genes involved in the SL signaling pathway and hormonal regulatory networks (e.g., *Os*D53, *Os*D14, *Os*PIN1B, *Os*MYB2), which were highly consistent with the transcriptomic data. Furthermore, we have revised statements that were previously too definitive or lacking direct support into more cautious, discussion-oriented language. We thank you again for your valuable input.

17. The claim "Furthermore, this study reveals a novel mechanism by which DK6 regulates tillering through interference with a multi-hormone network" is not substantiated by the data. Elucidating this proposed novel

mechanism would necessitate doing controlled experiments to test this proposal.
Our response: Thank you for your valuable comment. We fully agree that the original statements lacked sufficient data support and were overly speculative. Therefore, we have revised the absolute wording to a more cautious description. In addition, we have added RT-qPCR experiments in the revised manuscript to validate the expression changes of key genes involved in the SL signaling pathway and multi-hormone networks (e.g., *Os*D53, *Os*D14, *Os*PIN1B, *Os*MYB2). The results are highly consistent with the transcriptomic data, providing more solid evidence for the inference that "DK6 may regulate tillering through influencing multi-hormone networks." We thank you again for your correction.

18. "Thus, this work not only confirms the pharmacological function of DK6 as an SL signaling inhibitor at the molecular level, but also elucidates its complex role in modulating rice tillering via a multi-hormone network, providing a new theoretical foundation for improving plant architecture in crops." No direct evidence of engagement with an SL receptor is presented, so this claim is not subtantiated. Also, there is no reference to previous studies proposing molecular targeting of Striga receptors to design potent and specific antagonists, however they say that this study provides a new theoretical foundation for a related purpose; this claim seems to be too ambitious.
Our response: Thank you for your insightful criticism. We fully agree with your assessment and have made substantial revisions accordingly.

First, to further validate the direct interaction between DK6 and SL receptors, we have added microscale thermophoresis (MST) experiments measuring the binding affinity of DK6 and the control compound KK094 to the Arabidopsis SL receptor *At*D14 (Fig. 7D). The results show that DK6 exhibits superior binding to *At*D14 compared to KK094, providing direct molecular evidence that DK6 targets an SL receptor and inhibits its signaling. In addition, we validated the expression changes of SL signalling and other hormone-related genes (e.g., *Os*D53, *Os*D14, *Os*PIN1B, *Os*MYB2) using RT-qPCR, which were highly consistent with the transcriptomic data, further strengthening the reliability of our conclusions (Fig. 9F). Nevertheless, the detailed molecular mechanism still requires further comprehensive investigation.

Second, we have revised the over-definitive statements. Following your suggestion, the original sentence: "Thus, this work not only confirms the pharmacological function of DK6 as an SL signaling inhibitor at the molecular level, but also elucidates its complex role in modulating rice tillering via a multi-hormone network, providing a new theoretical foundation for improving plant architecture in crops." has been changed to a more cautious, evidence-appropriate statement: "Therefore, these expression changes are consistent with the expected effects of DK6 as an antagonist of the SL signalling pathway, providing transcriptome-level insights into its mechanism of action, although the detailed molecular mechanism requires further investigation."

We believe these revisions have made the conclusions more rigorous and objective, avoiding overinterpretation. Thank you again for your valuable input.

19. "Mechanistic studies revealed that DK6 stably binds to the OsD14 and ShHTL7 receptors with higher binding affinity than KK094" It should be made clear that, aside from docking, no experiments show direct engagement with OsD14 and ShHTL7 in this study.
Our response: Thank you for your valuable comments. We agree that molecular docking and dynamics simulations are computational predictions and do not constitute experimental evidence of direct binding. In response to this concern, we have revised the manuscript accordingly.

In this study, we were unable to successfully express and purify the rice *Os*D14 or *Striga Sh*HTL7 receptor proteins, precluding direct binding experiments for these two targets. Following your suggestion, we performed microscale thermophoresis (MST) using the successfully obtained *Arabidopsis* homologous receptor *At*D14 to measure the binding affinities of DK6 and the control compound KK094. The results showed that DK6 binds *At*D14 with a K_d of 3.72 ± 1.509 μM, which is significantly better than that of KK094 (K_d = 10.55 ± 3.508 μM), providing biochemical evidence for the binding capacity of DK6 to *At*D14. Given the high sequence homology and conserved binding pocket architecture between *At*D14 and *Os*D14, this result provides indirect evidence that DK6 may target rice SL receptors. However, we have explicitly noted in the manuscript that direct binding of DK6 to *Os*D14 and *Sh*HTL7 receptors requires further confirmation (see "Microscale Thermophoresis Analysis"). To clearly distinguish computational predictions from experimental evidence, we have revised the conclusion in the manuscript (Manuscript-track change).

The revisions have been marked in the revised manuscript. Thank you again for helping us improve the rigor and credibility of our work.

20. It would be great, as a proof of concept, to show how well the proposed hydrogel works on actual soil conditions that resemble the fields. Or at least comment on this expectation in the discussion.

Our response: Thank you for your valuable feedback. We fully agree that validating the hydrogel's performance in real soils under field-simulated conditions is of great importance for proof-of-concept. In response to your suggestion, we have added the following experimental data to the revised manuscript.

Regarding cumulative release and its kinetics in soils, we selected six paddy soils differing in pH and texture from Hunan, Jiangsu, Sichuan, Henan, Heilongjiang, and Liaoning, and systematically evaluated the 48h release behavior of CMCS-DK6 hydrogel. The results showed that cumulative release rates ranged from 63.5% to 86.1% across the different soils, all reaching effective release levels, with release kinetics following a first-order model (R^2 = 0.94-0.99). Compared with the PBS buffer system, the buffering and adsorption effects in actual soil solutions moderately attenuated the pH-dependent release profile (Fig. 6 and Table 1, Supplementary). Furthermore, when rice was grown for 45 days in the same six soils supplemented with different concentrations of CMCS-DK6, the hydrogel promoted rice tillering to varying extents (Fig. 7, Supplementary). In addition, it was clearly pointed out that the impact of soil physical and chemical properties on release kinetics needs to be further quantified in the future to optimize the application strategy of hydrogels. These additional results further confirm the efficacy of the CMCS-DK6 hydrogel in real soil environments and provide direct experimental support for its potential field application. The relevant findings have been incorporated into the "Release Behavior of CMCS-DK6 Hydrogel Delivery System" section of the revised manuscript.

We thank the reviewer again for the constructive comments, which have substantially improved the completeness and applied value of our study.

REVIEWER COMMENTS

Dear Professor Tan,

Thank you again for submitting your revised manuscript "pH-Responsive Manganese-Based Carboxymethyl Chitosan Hydrogel Triggers Pesticide Release: Promoting Rice Tillering and Suppressing Root Parasitic Weeds" to Nature Communications. We have now received reports from the reviewers who evaluated the original version. Due to the unavailability of reviewer#1, we got the assessment from reviewer#4 instead and they are overall satisfied with your revision pertaining to reviewer#1's comments (see attachment). On the basis of their comments (copied below), we have decided to invite an additional revision of your work.

You will see that, while the reviewers find that your revisions improved the manuscript, some important points remain to be addressed. Please revise your manuscript, addressing all the remaining issues raised by the reviewers.

Our response:

Dear Dr. Narasimhan,

Thank you very much for your kind letter and for facilitating the re-review process. We truly appreciate the time and effort of the reviewers, and we are grateful that Reviewer #4 found our revisions satisfactory. We have carefully considered all remaining comments and concerns raised by the reviewers and have thoroughly revised the manuscript accordingly. A detailed point-by-point response is enclosed. We believe that the revised version now meets the standards for publication in ***Nature Communications***.

Reviewer #1 (Remarks to the Author):

1. I believe this point is fully addressed in the introduction now.

Our response: We are grateful for your positive feedback on this point.

2. Material pore structure is not my area of expertise, but it looks like the authors addressed the reviewer's comments via a Brunauer-Emmett-Teller test, which failed due to the dry nature of the materials tested.

Our response: Thank you very much for your valuable comments. We previously performed BET analysis on the hydrogel, but the data were not usable. The reason is that the vacuum heating degassing pretreatment required for BET measurement forcibly removed the internal water from the hydrogel, causing the polymer network to shrink/collapse upon loss of swelling support. Consequently, the obtained adsorption isotherms could not reflect the true pore parameters of the material, rendering the data of no practical reference value. This outcome reflects a conflict between the testing method and the material characteristics, rather than an operational error.

3. The authors performed a real-life experiment showing rice growth in the presence of hydrogel containing DK6 to support the patterns previously described in PBS are consistent in soil. Nevertheless, there are no replicates to this experiment. Also, the test in line 620 of the supplemental document indicates 20 and 40 mg of the "active ingredient" were added per pot, while the figure caption (line 627) shows 20 and 40 μM of gel-containing DK6 were used. Which one is correct? Also, no methods for these experiments were included to the methods section.

Our response: Thank you for pointing out these issues. The soil pot experiment was conducted with three independent replicates ($n = 3$), and this information has been added to the Methods section. The unit in the legend of Supplementary Fig. 7 was incorrectly given as 20 and 40 μM. The correct doses are 20 and 40 mg of the active ingredient DK6 per pot, and this has been corrected in the revised Supplementary. The detailed experimental procedure for the soil pot assay is provided in the Supplementary Methods section under "Determination of rice growth in different soils." To avoid ambiguity, a citation to this Supplementary Methods section has also been added to the revised manuscript.

4. Addressed by including new RT-qPCR experiments.

Our response: Thank you for confirming that this point has been addressed.

5. It appears most experiments that required replicates/statistical analyses now include them. However, it is unclear if the new MST and soil experiments were performed more than once.

Our response: Thank you for pointing out this lack of clarity. Both the MST and soil experiments were performed in triplicate ($n = 3$). The corresponding descriptions have been added to the manuscript.

6. Figures showing statistical significance now include an annotation to describe their meaning.

Our response: Thank you for acknowledging this improvement.

7. Species name corrected.

Our response: Thank you for confirming this correction.

8. Spelling error corrected.

Our response: Thank you for confirming this correction.

9. Readability of Supplementary document is now improved.

Our response: Thank you for acknowledging this improvement.

We sincerely thank you for carefully evaluating our revised manuscript and responses, particularly in the absence of the original Reviewer #1. We greatly appreciate your time, effort, and constructive assessment, which have helped us further improve the clarity and rigor of the manuscript.

Reviewer #2 (Remarks to the Author):

The manuscript is recommended for publication as all our concerns were addressed

Reviewer #3 (Remarks to the Author):

The authors have resolved the raised issues, and the current version of the manuscript can be accepted.

Reviewer #4 (Remarks to the Author):

1. In their rebuttal, the authors explain that compound DK6 was tested at 1, 5, 15 μM to be able to obtain the 2.15 μM IC50; however, there is no mention of these concentrations in the methods or main text. On page 2, the target compounds were said to have been tested at 5-80 μM. This would not allow the obtention of such a low IC50.

Our response: We thank you for carefully scrutinizing this issue and for emphasizing the importance of data rigor. Regarding the concentration ranges: the 1, 5, and 15 μM treatments were used for rice tillering activity (Supplementary Fig. 3A), whereas the seed germination inhibition assay (Table 1) employed 5–80 μM, these are independent experiments. We apologize for any ambiguity in our previous response.

You correctly noted that fitting an IC_{50} of 2.15 μM from data starting at 5 μM is methodologically questionable. To address it, we performed additional germination inhibition assays using *Striga asiatica* seeds co-treated with 100 nM GR24. DK6, CMCS-DK6, DK1, and DK7 were tested at additional lower concentrations of 0.3125, 0.625, 1.25, 2.5, and 5 μM, with at least three independent replicates. The recalculated IC_{50} value for DK6 was 2.25 μM, which is consistent with the original estimate and supports the reliability of our conclusion.

Accordingly, we have revised the manuscript by updating the IC_{50} values in Table 1, revising Fig. 6B, specifying in the table and figure legends that the concentration ranges for selected compounds were extended to 0.3125–80 μM, adding the additional concentration ranges to the Methods section, and correcting the corresponding values in the Results section.

We appreciate your constructive comment, which helped improve the completeness and rigor of the data.

2. The statement "Molecular docking is a key technique for elucidating ligand–receptor interactions and quantifying binding affinity." Is not correct; yes, docking is used to predict ligand binding to a protein pocket; using it to quantify binding affinity is not something serious biochemical/enzymatic studies perform.

Our response: We apologize for the inaccurate wording in the original statement. Accordingly, we have revised the sentence to: "Molecular docking is a valuable computational method for predicting ligand–receptor binding modes and elucidating key intermolecular interactions."

3. The authors claim binding/structural studies of AtD14 binding to DK6 are reflective of the mechanism SL receptors OsD14 and ShHTL7 would follow. Nevertheless, Fig. 7E shows no docking on AtD14, and instead shows OsD14 and ShHTL7. It is claimed that DK6 has a "binding advantage" in ShHTL7 of 0.2 kcal/mol; this is

within the range of error, and I do not agree with that claim. It is also unclear how those values were calculated as none of the figures contain them. If the point is to show the binding mode between 2 highly similar receptors (OsD14 and ShHTL7) is similar, the docking figures presented do not represent that. The compound adopts completely different orientations in the 2 pockets, and the catalytic residues interact with different/opposite atoms of the small molecule. Overall, my conclusion is that the MD/docking section does not support the claims that DK6 acts primarily through ShHTL7 and is a better inhibitor of parasitic SL receptors than KK094.

Our response: We thank you for your detailed and constructive comments, which helped us identify several overstatements and clarify the limitations of the computational analysis. We have revised this section accordingly.

First, we agree that the *At*D14 binding results should not be directly extrapolated to *Os*D14 or *Sh*HTL7. In the revised manuscript, we have further clarified that the direct binding of DK6 to *Os*D14 and *Sh*HTL7 remains to be experimentally validated, and we have removed or revised wording that could imply such extrapolation.

Second, we agree that the 0.2 kcal/mol difference in docking scores is within the expected uncertainty of docking calculations and should not be interpreted as evidence of a binding advantage. We have therefore removed the claim that DK6 has a "binding advantage" in *Sh*HTL7 and revised the discussion to focus only on qualitative differences in predicted binding modes.

Third, we acknowledge that the current MD/docking results are insufficient to conclude that DK6 acts primarily through *Sh*HTL7 or that it is a better inhibitor of parasitic SL receptors than KK094. We have revised the manuscript to clearly distinguish experimentally supported findings from computational predictions and have tempered the related conclusions accordingly.

We appreciate your rigorous evaluation, which has improved the clarity and accuracy of this section.

4. While I partially understand the authors did not have OsD14/ShHTL7 protein and they were not able to generate knockdown lines of these receptors (even though other groups have generated both recombinant protein and plant lines), the lack of correlation with docking makes the whole argument weaker. The only claim fully supported in this part of the study, using MST, is that DK6 appears to have a lower Kd than KK094 in an Arabidopsis SL receptor.

Our response: We thank you for your careful reading and constructive comments. We fully agree that direct binding experiments are essential to validate molecular docking predictions, and we candidly acknowledge that the current study only confirms DK6 binding to *At*D14, lacking direct data for *Os*D14 and *Sh*HTL7, this is indeed a limitation of our work.

Because recombinant *Os*D14 and *Sh*HTL7 proteins were not available to us, we chose *At*D14 as a representative surrogate based on the high sequence and binding-pocket conservation among the three receptors. The results at least demonstrate that DK6 can directly interact with a member of the SL receptor family, with higher affinity than the positive control KK094, thereby providing initial biochemical evidence for targeting SL receptors by DK6.

Regarding the relationship between docking and MST, we recognise that they represent different levels of evidence. In the manuscript, docking and MD simulations are used solely as predictive tools to offer a possible structural-biology framework for interpreting DK6 activity, without substituting for direct binding assays, and they are clearly labelled as predictions throughout the text.

We plan to add the following limitation statement in the Discussion section, and we will endeavour to purify *Os*D14 and *Sh*HTL7 proteins or contact collaborating groups to obtain them for future direct binding studies: "It should be noted that while MST analysis confirmed the direct binding of DK6 to *At*D14, direct binding assays with *Os*D14 and *Sh*HTL7 recombinant proteins were not performed in the current study due to the limited availability of these proteins. Given the high sequence and structural conservation of the SL receptor family, the binding affinity observed with *At*D14 supports the notion that DK6 likely interacts with other SL receptors as well. However, direct biochemical evidence for DK6 binding to *Os*D14 and *Sh*HTL7, as well as genetic evidence from corresponding knockout lines, will be essential to fully validate the molecular mechanism and will be pursued in future investigations."

We appreciate your professional guidance, which helped us clarify the hierarchy of evidence and avoid overinterpretation of the computational results.

5. The reference cited for PDB entry code 7SNU is incorrect, please add the correct primary reference.

Our response: Thank you for pointing out the error in our references. We sincerely apologise for this oversight. Upon verification, the correct reference for 7SNU is: Arellano-Saab, A. et al. A novel strigolactone receptor antagonist provides insights into the structural inhibition, conditioning, and germination of the crop parasite Striga. *J. Biol. Chem*. **298**, 101734 (2022). We have now updated this as reference [73] in the revised manuscript for your verification.

6. The authors claim that new qPCR experiments are helpful to reach the conclusion "this study reveals a novel mechanism by which DK6 regulates tillering through interference with a multi-hormone network." This is not my field of expertise, and I am not certain these experiments fully support the conclusion. Again, the lack of data that shows direct engagement with the proposed parasitic receptors is the main problem here. However, the statements included in the previous version were toned down and might be appropriate considering the experimental evidence provided.

Our response: Thank you for your further review of the revised manuscript. We agree that qPCR alone is insufficient to establish a complete regulatory mechanism. Our conclusions are primarily based on GO/KEGG enrichment of differentially expressed genes, known literature on SL-hormone crosstalk, and qPCR validation of key gene expression trends, with qPCR serving mainly to verify data reliability. We have now clearly stated in the manuscript that these findings represent only "transcriptional regulatory clues." Accordingly, we have revised the definitive statement "Furthermore, this study reveals a novel mechanism by which DK6 regulates tillering through interference with a multi-hormone network." to a more cautious phrasing: "Furthermore, our transcriptomic analysis suggests that DK6 may cooperatively regulate rice tillering with other hormone-related genes." to better reflect the strength of evidence.

Regarding the MST experiments, we acknowledge that we have only validated the binding of DK6 to *At*D14, and direct binding data for *Os*D14 and *Sh*HTL7 are not yet available. We have explicitly noted this limitation in the Discussion and used more tempered language to describe the predictive and referential value of molecular docking. We are actively working on purifying the relevant proteins or seeking collaborative support to obtain direct evidence. We appreciate your pointing out this issue and are encouraged that you find the current version appropriately adjusted and acceptable. Thank you again for your understanding and guidance.

7. A lot of the original questions were answered through MST; however, there is little to no information to show protein purification (aside from the methods section, which include a few phrases clearly copied from a generic recombinant protein purification protocol). There is also no explanation as to how the protein was fluorescently labeled or the settings used for thermal fluorescence. No explanation related to the number of replicates (if any) for the MST experiment or any statistics related to it.

Our response: Thank you for your constructive comments on the MST experimental methods. We fully agree with your suggestions regarding protein purification details, fluorescent labeling, instrument settings, replicate numbers, and statistical data, and we will address each point.

First, regarding protein purification, we confirm that the expression and purification of *At*D14 were previously described in Supplementary section 11 ("Construction and purification of *At*D14 protein expression vector"), including expression strain (E. coli DE3 pLysS), induction (0.2 mM IPTG, 18 °C overnight), lysis (sonication on ice, 30% power, 2 s on/3 s off), Ni-NTA affinity chromatography with stepwise elution (50, 100, and 150 mM imidazole), and SDS-PAGE quality validation. We will explicitly direct readers to this section in the revised Methods.

Second, we will revise and expand the fluorescence labeling and instrument description as follows: *Binding affinities of compounds DK6/KK094 to AtD14 were determined using microscale thermophoresis (MST) on a Monolith NT.115 instrument (NanoTemper Technologies). AtD14 was fluorescently labeled with the Monolith NT Protein Labeling Kit RED-NHS (2nd Generation). Test compounds were serially diluted and mixed with an equal volume of labeled AtD14 at 25 °C in the dark for 5 min. Samples were loaded into standard treated capillaries*

and measured on the Monolith NT.115 according to the preset program, with three replicates. Data were analyzed by nonlinear fitting using MO.Affinity Analysis software to obtain dissociation constants (K_d).

Third, we confirm that the MST experiment was performed in three replicates, and the fitted Kd values have been previously provided in the Source Data file.

We sincerely thank you for your careful and rigorous evaluation of our revised manuscript. Your comments have been particularly valuable in helping us improve the precision and rigor of our wording, better distinguish experimentally supported conclusions from computational predictions, and avoid overinterpretation of the available data. We also appreciate your constructive suggestions regarding methodological clarity, including the MST experimental details, statistical descriptions, and reference accuracy. These comments have substantially improved the scientific clarity, transparency, and reliability of the manuscript.

Editorial Note: Comments from Reviewer#4 mediating Reviewer#1 in Version 1.

Reviewer #1 rebuttal comments

Following a point-by-point structure as in the original Reviewer #1's comments.

1. I believe this point is fully addressed in the introduction now.
2. Material pore structure is not my area of expertise, but it looks like the authors addressed the reviewer's comments via a Brunauer-Emmett-Teller test, which failed due to the dry nature of the materials tested.
3. The authors performed a real-life experiment showing rice growth in the presence of hydrogel containing DK6 to support the patterns previously described in PBS are consistent in soil. Nevertheless, there are no replicates to this experiment. Also, the test in line 620 of the supplemental document indicates 20 and 40 mg of the "active ingredient" were added per pot, while the figure caption (line 627) shows 20 and 40 μM of gel-containing DK6 were used. Which one is correct? Also, no methods for these experiments were included to the methods section.
4. Addressed by including new RT-qPCR experiments.
5. It appears most experiments that required replicates/statistical analyses now include them. However, it is unclear if the new MST and soil experiments were performed more than once.
6. Figures showing statistical significance now include an annotation to describe their meaning.
7. Species name corrected.
8. Spelling error corrected.
9. Readability of Supplementary document is now improved.
